# Alkaline‐Earth Metal Mediated Benzene‐to‐Biphenyl Coupling

**DOI:** 10.1002/anie.202212463

**Published:** 2022-12-14

**Authors:** Jonathan Mai, Michael Morasch, Dawid Jędrzkiewicz, Jens Langer, Bastian Rösch, Sjoerd Harder

**Affiliations:** ^1^ Inorganic and Organometallic Chemistry Universität Erlangen-Nürnberg Egerlandstrasse 1 91058 Erlangen Germany

**Keywords:** Alkaline-Earth Metal, Cross-Coupling, DFT Calculations, Low-Valent, Mechanochemistry

## Abstract

Complex [(^DIPeP^BDI)Ca]_2_(C_6_H_6_), with a C_6_H_6_^2−^ dianion bridging two Ca^2+^ ions, reacts with benzene to yield [(^DIPeP^BDI)Ca]_2_(biphenyl) with a bridging biphenyl^2−^ dianion (^DIPeP^BDI=HC[C(Me)N‐DIPeP]_2_; DIPeP=2,6‐CH(Et)_2_‐phenyl). The biphenyl complex was also prepared by reacting [(^DIPeP^BDI)Ca]_2_(C_6_H_6_) with biphenyl or by reduction of [(^DIPeP^BDI)CaI]_2_ with KC_8_ in presence of biphenyl. Benzene‐benzene coupling was also observed when the deep purple product of ball‐milling [(^DIPP^BDI)CaI(THF)]_2_ with K/KI was extracted with benzene (DIPP=2,6‐CH(Me)_2_‐phenyl) giving crystalline [(^DIPP^BDI)Ca(THF)]_2_(biphenyl) (52 % yield). Reduction of [(^DIPeP^BDI)SrI]_2_ with KC_8_ gave highly labile [(^DIPeP^BDI)Sr]_2_(C_6_H_6_) as a black powder (61 % yield) which reacts rapidly and selectively with benzene to [(^DIPeP^BDI)Sr]_2_(biphenyl). DFT calculations show that the most likely route for biphenyl formation is a pathway in which the C_6_H_6_^2−^ dianion attacks neutral benzene. This is facilitated by metal‐benzene coordination.

## Graphical abstract

Attempts to isolate low‐valent (BDI)Ae‐Ae(BDI) complexes led to reduction of the benzene solvent and formation of (BDI)Ae‐(C_6_H_6_)‐Ae(BDI); BDI=β‐diketiminate ligand, Ae=Ca or Sr. Reaction of bridging C_6_H_6_^2−^ with benzene gave (biphenyl)^2−^ complexes. Ball‐milling is an efficient synthetic tool for this dehydrogenative benzene‐benzene coupling. Calculations suggest attack of C_6_H_6_^2−^ at benzene. This is facilitated by Ae^2+^⋅⋅⋅benzene coordination.

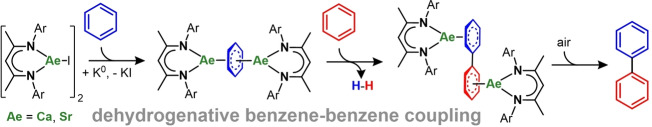

Since biphenyl is a frequently applied building block in pharmaceuticals and fine‐chemicals, there is wide interest in aryl‐aryl coupling (Scheme 1).[^1^, ^2^, ^3^] Classical preparative routes, like the century old Cu‐mediated Ullmann coupling,^[4]^ have largely been replaced by numerous Pd‐catalyzed pathways.^[2]^ From an industrial point of view, nowadays the most popular Suzuki coupling is state‐of‐the‐art.^[5]^ These methods rely on aryl halide feedstocks, mostly using heavier bromide or iodide substrates.

**Scheme 1:**
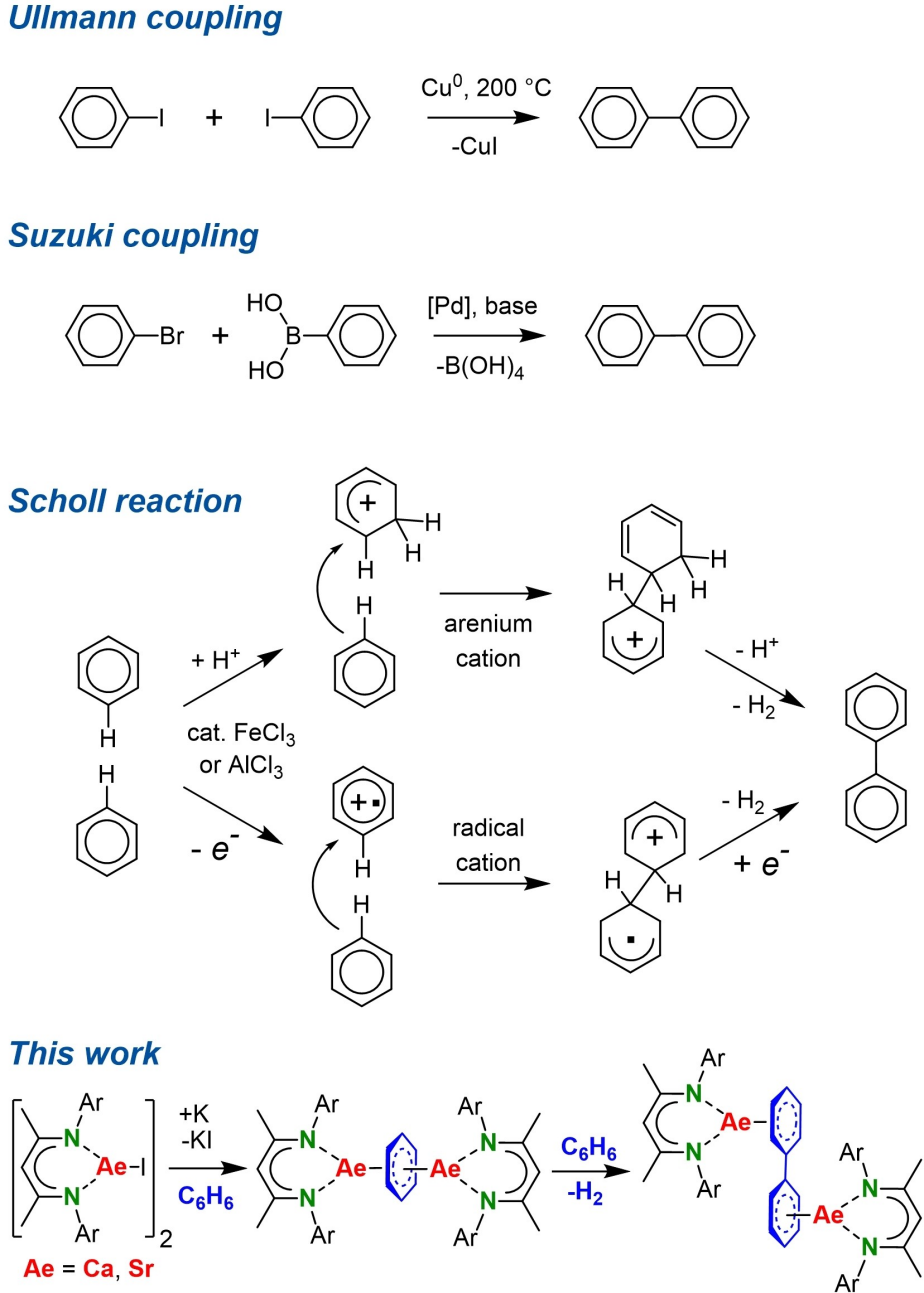
Biphenyl formation.

The more sustainable approach to biaryl formation is the direct dehydrogenative coupling of arene C−H bonds which, given the fact that C−H bonds are almost as strong as the rather inert C−F bond,^[6]^ is a challenging process. While present research activities for direct arene‐arene coupling focus on Pd catalysis,[^7^, ^8^, ^9^, ^10^, ^11^] the classical Scholl reaction^[12]^ using simple Lewis‐acidic catalysts like AlCl_3_ or FeCl_3_ is an attractive alternative (Scheme 1).[^13^, ^14^] However, apart from the need of forcing reaction conditions (>+100 °C), it has limitations like the requirement of electron‐poor arene substrates or intramolecular forced proximity of the C−H bonds. Arene‐arene coupling in the Scholl process proceeds through a cationic intermediate which is either arenium or radical in nature.^[14]^ The remaining alternative of radical anion coupling is rare.^[15]^ In contrast to anionic pyridine‐pyridine coupling,^[16]^ there are only scarce examples for benzene‐benzene coupling via an anionic intermediate. Benzene does not react with alkali metals but cocondensation of K, Rb or Cs with benzene resulted in C_6_H_6_⋅^−^ salts and radical coupling to (biphenyl)^2−^ and H_2_.^[17]^ Similarly unique is benzene‐benzene coupling between the graphite layers of KC_8_, a peculiar reaction that only takes place in presence of benzene and THF,^[18]^ even resulting in formation of larger benzene polymers.^[19]^ Herein we introduce the direct dehydrogenative coupling of benzene by low‐valent alkaline‐earth (Ae) metal intermediates and propose a mechanism through a unique complex with a C_6_H_6_^2−^ dianion.

Previously reported attempts to isolate a low‐valent Ca^I^ complex with the bulky β‐diketiminate ligand ^DIPeP^BDI led to reduction of the aromatic solvent and isolation of black crystals of [(^DIPeP^BDI)Ca]_2_(C_6_H_6_) (**I**),^[20]^ a paramagnetic complex with a bridging C_6_H_6_^2−^ dianion (Scheme 2a) (^DIPeP^BDI=HC[C(Me)N‐DIPeP]_2_; DIPeP=2,6‐CH(Et)_2_‐phenyl). A solution of **I** in hexane decomposed at room temperature very slowly to **II**, benzene and H_2_. In this reaction the C_6_H_6_^2−^ moiety acts as a 2*e* ^−^ donor, reducing the BDI anion to a dianionic *N,C*‐chelating ligand. We now found that changing the solvent to benzene led to slow formation of [(^DIPeP^BDI)Ca]_2_(biphenyl) (**1**) as the main decomposition product, as characterized by ^1^H NMR (Figure S34, S35). Further proof for biphenyl formation was obtained by addition of THF, which led to red crystals of the THF adduct (**1**‐THF) (crystal structure: Figure 1a). Other side‐products detected by ^1^H NMR are [(^DIPeP^BDI)Ca(μ‐H)]_2_ and (^DIPeP^BDI)_2_Ca (product ratios are dependent on the decomposition temperature; Figures S34–S36). Formation of **1** could also be achieved by reduction of biphenyl with the Ca^I^ synthon [(^DIPeP^BDI)Ca]_2_(C_6_H_6_) (**I**) or, even simpler, by in situ reduction of [(^DIPeP^BDI)Ca(μ‐I)]_2_ with KC_8_ in the presence of biphenyl (Scheme 2a).

**Scheme 2:**
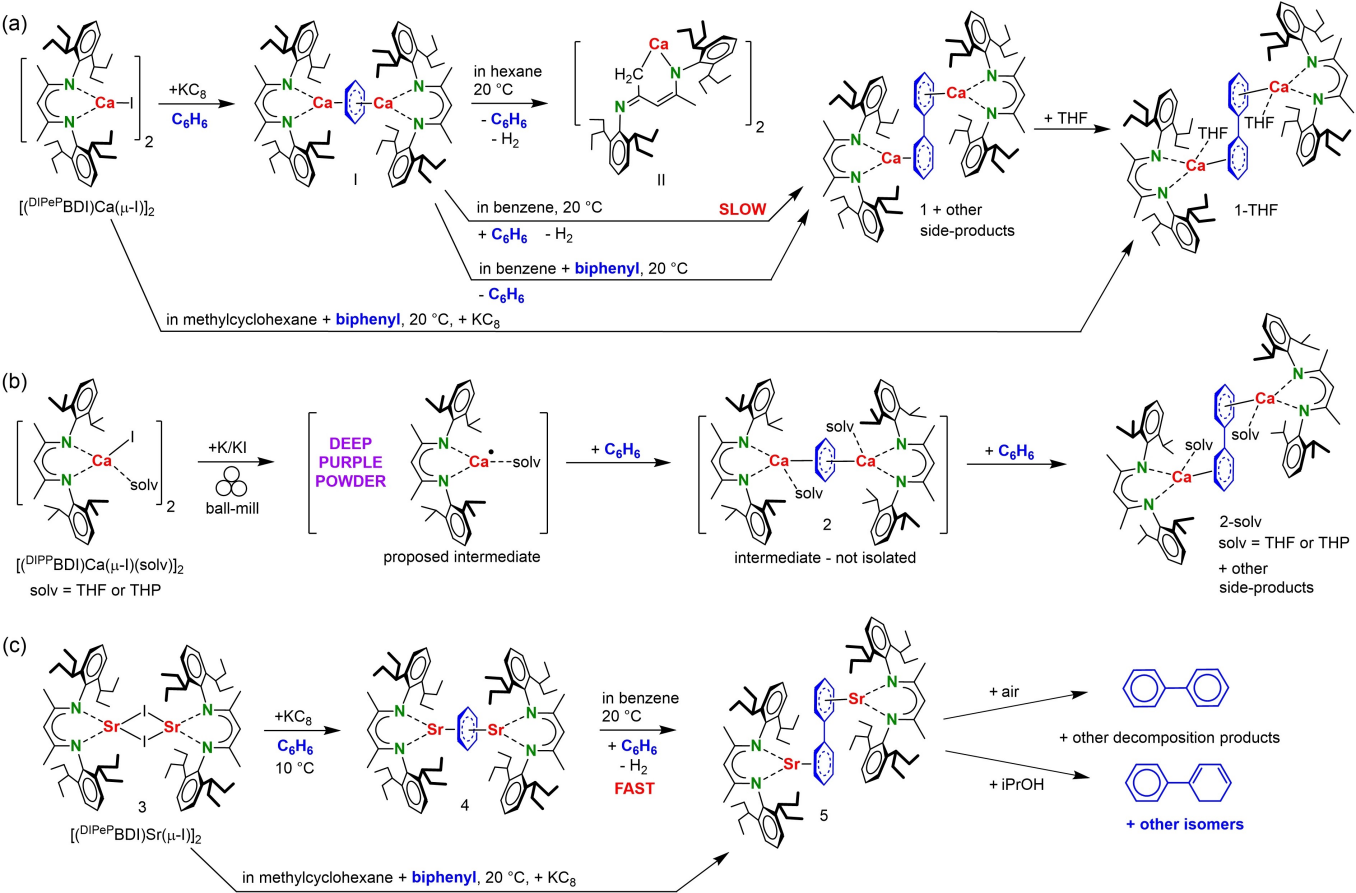
Syntheses of alkaline‐earth metal biphenyl complexes.

**Figure 1:**
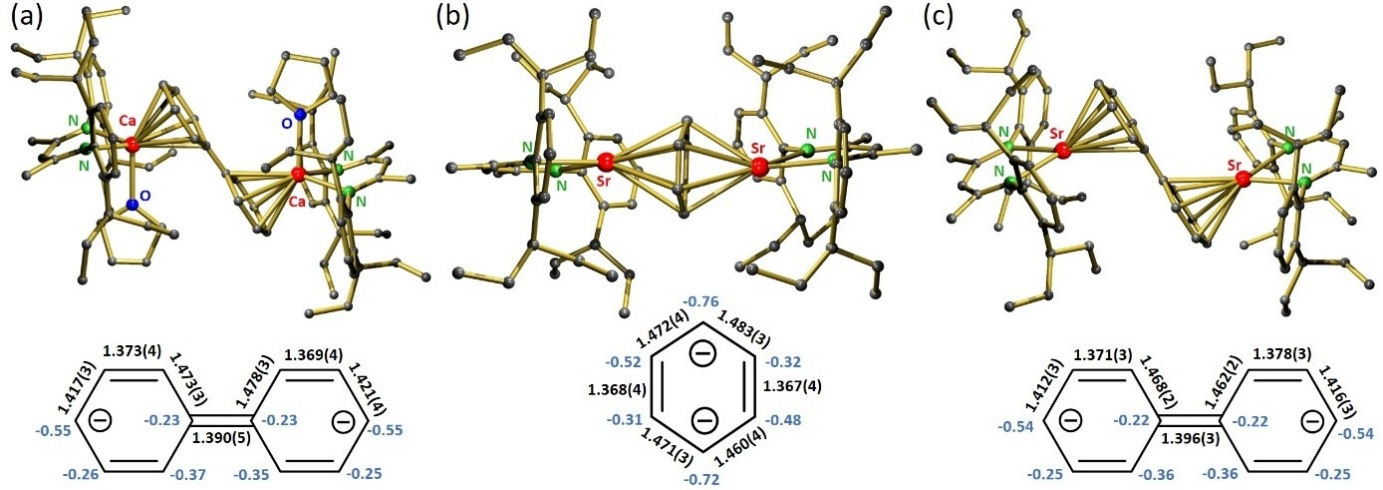
Crystal structures of a)  [(^DIPeP^BDI)Ca(THF)]_2_(biphenyl) (**1**‐THF)), b) [(^DIPeP^BDI)Sr]_2_(C_6_H_6_) (**4**) and c) [(^DIPeP^BDI)Sr]_2_(biphenyl) (**5**). The inset shows C−C bond lengths in black [Å] and NPA charges on C in blue.

An important requirement for isolation of the Ca^I^ synthon [(^DIPeP^BDI)Ca]_2_(C_6_H_6_) (**I**) is the very bulky DIPeP‐substituent in the BDI ligand. Reduction of a precursor with the smaller, more generally used DIPP‐substituent, [(^DIPP^BDI)Ca(μ‐I)(THF)]_2_, led mainly to formation of homoleptic (^DIPP^BDI)_2_Ca and other unidentified decomposition products (DIPP=2,6‐CH(Me)_2_‐phenyl). However, using ball‐milling changed the outcome of this reduction. We recently introduced ball‐milling to low‐valent Ae metal chemistry.[^21^, ^22^] The advantage of this technique is that the reactive radicals formed after reduction are partially “frozen” in the solid‐state. Similar as previously shown for formation of (BDI)Mg⋅ radicals,^[22]^ reduction of [(^DIPP^BDI)Ca(μ‐I)(THF)]_2_ with K/KI in the ball‐mill led to a deep purple powder. Extraction of the expected radical [(^DIPP^BDI)Ca⋅(THF)] (Scheme 2b) with benzene led to immediate formation of a dark‐red solution from which dark‐brown crystals of [(^DIPP^BDI)Ca(THF)]_2_(biphenyl) (**2**‐THF) separated in 52 % yield. During this process, the red color of the mother liquor rapidly faded to yellow. ^1^H NMR showed several decomposition products among which also the previously reported dimers [(^DIPP^BDI)Ca(μ‐H)(THF)]_2_ and [(^DIPP^BDI)Ca(μ‐Ph)]_2_ (Figure S30).[^23^, ^24^] A similar complex with a tetrahydropyran (THP) ligand was isolated in 54 % yield and could be structurally characterized (Figure S53).

Due to diminished steric protection by the ^DIPP^BDI ligand, any attempt to crystallize the presumed intermediate with a bridging C_6_H_6_^2−^ dianion failed. However, cold extraction gave a black powder with a ^1^H NMR spectrum reminiscent to that of paramagnetic **I** (Figure S8). This species decomposed rapidly under crystallization of the biphenyl complex **2**‐THF (Figure S29). We therefore presume that the intermediate with the bridging C_6_H_6_^2−^ dianion plays a pivotal role in biphenyl formation.

The intriguing reactivity of these Ca complexes with bridging C_6_H_6_^2−^ moieties, motivated the synthesis of the analogue Sr^I^ synthon [(^DIPeP^BDI)Sr]_2_(C_6_H_6_) (**4**; Scheme 2c) which due to the much higher reactivity and more difficult to control Schlenk equilibria of Sr complexes is challenging. The room temperature reduction of [(^DIPeP^BDI)Sr(μ‐I)]_2_ (**3**) in benzene did not lead to **4** but unselectively gave various decomposition products. However, after performing the reaction just above the melting point of the solvent (10 °C) and removal of benzene by freeze‐drying at −15 °C, [(^DIPeP^BDI)Sr]_2_(C_6_H_6_) (**4**) could be extracted with cold pentane in form of an essentially pure pitch‐black powder (61 % yield). We anticipate that this recently introduced benzene freeze‐drying method^[25]^ could generally become a key to the isolation of thermally labile complexes. Crystallization from pentane gave black crystals of **4** which are even at −20 °C only of limited stability. ^1^H NMR in cyclohexane‐*d*_12_ indicates that **4** is also paramagnetic (Figure S22). Like [(^DIPeP^BDI)Ca]_2_(C_6_H_6_) (**I**) is a synthon for hitherto unknown (BDI)Ca‐Ca(BDI) complexes, [(^DIPeP^BDI)Sr]_2_(C_6_H_6_) (**4**) could be considered as a synthon for a hitherto unknown Sr^I^ complex.

A C_6_D_6_ solution of **4** rapidly changed color from black to red‐brown under formation of the biphenyl complex [(^DIPeP^BDI)Sr]_2_(biphenyl) (**5**). In contrast to the slow and unselective reaction of **I** with benzene, reaction of **4** with C_6_D_6_ to **5** is highly selective (Figure S38). Alternatively, **5** can be obtained by reduction of [(^DIPeP^BDI)Sr(μ‐I)]_2_ with KC_8_ in methylcyclohexane in the presence of biphenyl. The poor yield of 13 % crystalline **5** is due to decomposition during crystallization, reflecting the low stability of these Sr complexes.

The centrosymmetric crystal structure of **1**‐THF shows a biphenyl^2−^ moiety that is bridging two (^DIPeP^BDI)Ca^+^⋅(THF) units (Figure 1a). The coplanar rings indicate extensive charge delocalization. The central C−C bond of 1.390(6) Å is considerably shorter than the C−C bond in biphenyl (1.507 Å)^[26]^ suggesting a quinoid structure with central C=C bond character. This is supported by the long‐short‐long C−C bond alteration in the rings, typical for a resonance structure with negative charges at the remote *para*‐C atoms. This is in agreement with the NPA charges (B3PW91/def2tzvp//def2svp) which are highest in these positions (Figure 1a). The total charge on the biphenyl unit (−1.74) indicates a Ca^II^ complex which is confirmed by a high positive charge on Ca (+1.77). The Ca−C bond lengths vary from 2.679(3) to 2.829(2) Å. The shortest Ca−C contacts are to the most electron‐rich *para*‐C atoms. Crystal structures of the biphenyl complexes [(^DIPP^BDI)Ca(solv)]_2_(biphenyl) (**2**‐solv; solv=THF or THP) show similar features.

The crystal structure of [(^DIPeP^BDI)Sr]_2_(C_6_H_6_) (**4**) (Figure 1b), with no crystallographic symmetry, shows a slightly puckered C_6_H_6_^2−^ dianion in a flattened boat form (max. C−C−C−C torsion angle: 9.2(1)°). The Sr−C distances are in the range of 2.718(3) to 2.952(3) Å. NPA charges on the C_6_H_6_^2−^ ring (−1.65) and on the Sr atoms (+1.76) are in agreement with a Sr^II^ complex. The bond lengths in the C_6_H_6_^2−^ ring indicate that **4** is in a singlet state with negative charges on C atoms in *para*‐position (at least in the crystal). A triplet state features a C_6_H_6_^2−^ ring with equal C−C bonds and has been calculated to be only 1–3 kcal mol^−1^ more stable (Figures S58, S59). Similar observations have been made for [(^DIPeP^BDI)Ca]_2_(C_6_H_6_) (**I**).^[20]^

The crystal structure of [(^DIPeP^BDI)Sr]_2_(biphenyl) (**5**) (Figure 1c) shows quinoid features comparable to those in **1**‐THF. The geometries and charge distribution of the bridging biphenyl^2−^ dianions are similar (charge on biphenyl: −1.76, charge on Sr: +1.79). The Sr−C distances vary from 2.787(2) to 2.947(2) Å. The quinoid structure of the biphenyl^2−^ dianion gives rise to a strong upfield shift of its proton NMR signals (Figures S38–S39). While reaction of **5** with *i*PrOH led to protonation of the biphenyl^2−^ moiety, oxidation with air gave biphenyl. We observed similar dual reactivity for a Ca‐bridged stilbene^2−^ dianion.^[27]^

The herein described dehydrogenative coupling of benzene is a highly unusual synthetic route to biphenyl. The far majority of inverse sandwich complexes of type M‐(C_6_H_6_)‐M react like electron donors, eliminating aromatic benzene.^[28]^ We recently reported protonation of a Mg‐bridged C_6_H_6_^2−^ dianion to give cyclohexadiene.^[29]^ Arnold and co‐workers reported the first functionalization of the C_6_H_6_^2−^ dianion by dehydrogenative C−B coupling with a borane (R_2_BH) to give Ph‐BR_2_ and H_2_.^[30]^ While boranes are highly electrophilic, the herein described nucleophilic attack of C_6_H_6_^2−^ at electron‐rich, aromatic C_6_H_6_ is unexpected and unique in inverse sandwich chemistry.^[31]^ It fits, however, with the recent observations that heavier Ae^2+^ metals cations (Ae=Ca, Sr) can facilitate such unusual nucleophilic substitutions at aromatic rings.[^32^, ^33^] Such processes are especially fast for the larger Sr metal.^[33]^

Following observations may shine a light on the mechanism of the dehydrogenative benzene coupling. (1) Complexes with bridging C_6_H_6_^2−^ dianions decompose in benzene to give the biphenyl complexes and are therefore likely intermediates. (2) There is experimental evidence that the bridging C_6_H_6_^2−^ dianions exchange with C_6_D_6_ (Figure S31). (3) Apart from biphenyl complexes, also side‐products like dimers with bridging hydrides and/or bridging Ph groups have been detected. These may point to benzene C−H activation by oxidative addition. The relative quantities of these side products are variable and depend on the reaction conditions (Figures S30, S34–S37). (4) Decomposition of [(^DIPeP^BDI)Sr]_2_(C_6_H_6_) (**4**) is in C_6_D_6_ much faster and more selective than in normal benzene (Figures S38–S40), illustrating an inverse isotope effect. GC‐MS analysis shows that **4** reacts in C_6_H_6_ to C_6_H_5_−C_6_H_5_ but in reaction with C_6_D_6_ mainly fully deuterated biphenyl was obtained. This shows that C_6_H_6_/C_6_D_6_ exchange is a very fast first step.

A preliminary DFT study evaluated two different mechanisms for a model system in which due to size limitations the DIPeP‐substituents have been replaced with smaller DIPP‐substituents (Scheme 3) or Ph‐substituents (Figure S60).

**Scheme 3:**
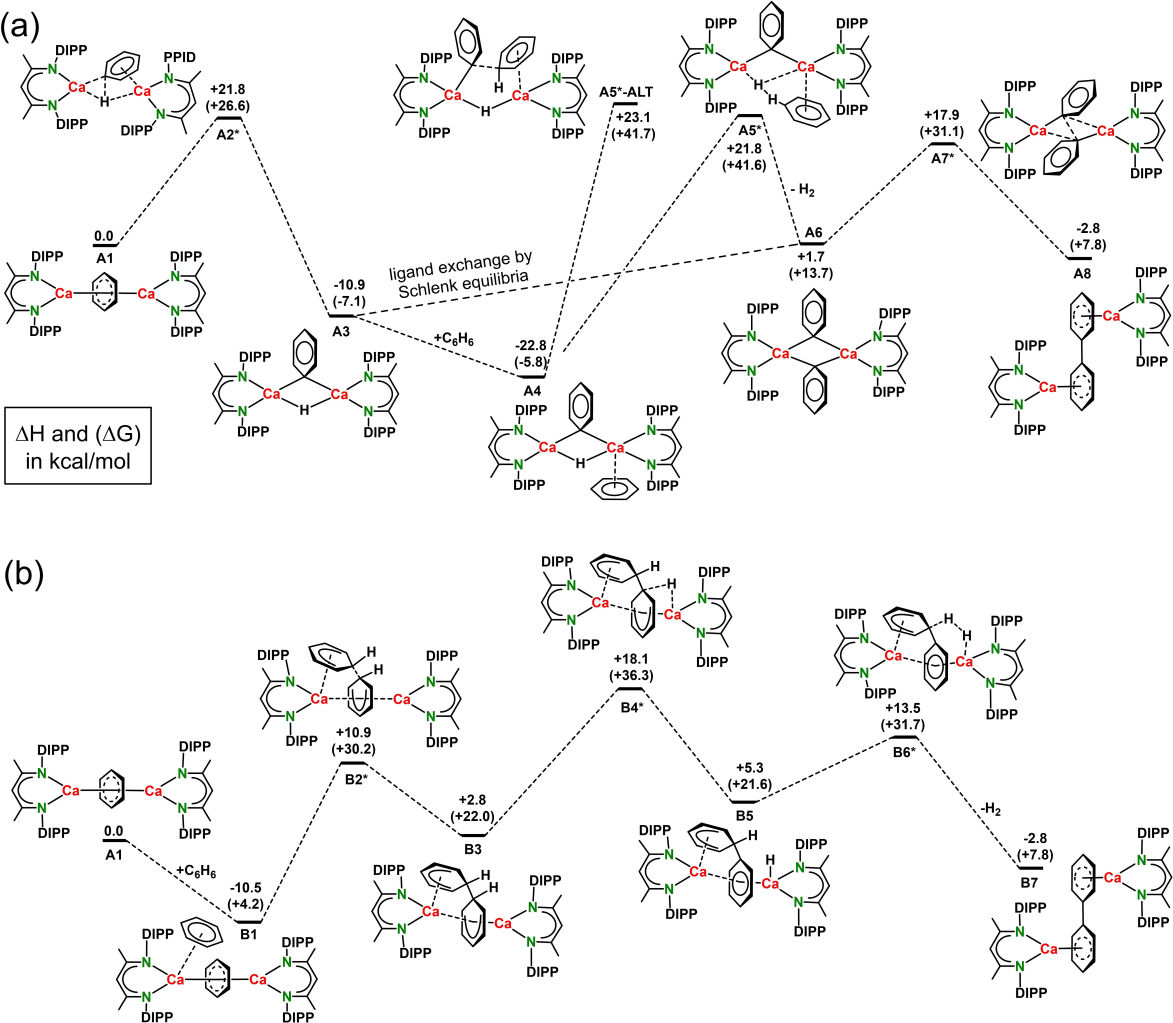
Energy profiles for benzene‐benzene coupling calculated at the B3PW91/def2tzvp//def2svp level of theory for a model system with ^DIPP^BDI ligands. Δ*H* in kcal mol^−1^. Between brackets: Δ*G*(298 K) in kcal mol^−1^. a) Pathway A via Ph^−^−Ph^−^ coupling. b) Pathway B via C_6_H_6_^2−^→benzene attack.

Pathway A starts with cleavage of the benzene C−H bond (Scheme 3a; **A1**‐A2*‐**A3**). The formation of the mixed (μ‐H,μ‐Ph)‐dimer **A3** is exothermic by Δ*H*=−10.9 kcal mol^−1^ and has a barrier of +21.8 kcal mol^−1^. A similar but much slower C−H bond cleavage has also been observed for the analogue Mg complex [(^Ar^BDI)Mg]_2_(C_6_H_6_).[^29^, ^34^] Benzene coordination (**A3**‐**A4**) is slightly exothermic but subsequent C−C coupling by direct attack of Ph^−^ at benzene (**A4**‐A5*‐ALT) is with a barrier of +45.9 kcal mol^−1^ highly endothermic. Alternatively, the H^−^ anion could deprotonate benzene. The transition state for this conversion (A5*) is also very high (the activation energy is 44.6 kcal mol^−1^). It should, however, be noticed that **A6** may also be obtained from **A3** by ligand distribution via a Schlenk equilibrium. The next transition state A7* is unusual in the sense that it represents nucleophilic attack of a Ph^−^ anion at a Ph^−^ anion. This can be envisioned by a side‐way approach leading to HOMO–LUMO interaction. Although unconventional, there is precedence for a comparable C−C coupling of two acetylide anions.^[35]^ The calculated energy barrier of +16.2 kcal mol^−1^ is the lowest along this pathway. The total reaction is only slightly exothermic by −2.8 kcal mol^−1^.

Like in pathway A, the starting point for route B is the complex with the bridging C_6_H_6_^2−^ anion (**A1**); Scheme 3b. Benzene complexation and subsequent C_6_H_6_^2−^→benzene attack (B2*) needs an activation enthalpy of Δ*H*=+21.4 kcal mol^−1^. The C−C coupling product **B3** can be considered as a double Meisenheimer anion. A similar dianion with potassium has been isolated previously from a K‐crown ether‐benzene mixture by Lappert and co‐workers.^[36]^ The transition state for C−C coupling (B2*, Scheme 3b) is comparable to that recently calculated for benzene coupling in a Li(benzene)_2_^−^ sandwich.^[37]^ The bridging dianion in complex **B3** could lose a hydride (B4*) requiring an activation enthalpy of Δ*H*=+15.3 kcal mol^−1^. After relatively facile elimination of H_2_ (**B5**‐B6*: +8.2 kcal mol^−1^) the final biphenyl product **B7** is formed.

With a highest barrier of +21.4 kcal mol^−1^, route B seems more favorable than route A. This is in agreement with the recent isolation of [(^DIPP^BDI)Ca(μ‐Ph)]_2_ which upon heating in C_6_D_6_ did not show any evidence for biphenyl formation.^[24]^ However, the start of pathway A, i.e. C−H bond cleavage, may be responsible for formation of the observed side‐products with Ph^−^ or H^−^ anions.

This preliminary experimental and theoretical study suggests that benzene‐benzene coupling indeed could start from a C_6_H_6_^2−^ complex that reacts with a neutral benzene ligand following pathway B. It has been shown previously[^32^, ^33^] that heavier Ae metal cations like Ca^2+^ and Sr^2+^ can facilitate such unusual nucleophilic substitutions at aromatic benzene by Ae^2+^⋅⋅⋅benzene coordination.[^38^, ^39^] Such processes are especially fast for complexes with the larger Sr^2+^ cation.^[33]^ Ball‐milling is an attractive new approach to this chemistry. Our investigations demonstrate that [(BDI)Ca]_2_(C_6_H_6_) and [(BDI)Sr]_2_(C_6_H_6_) are not just synthons for Ca^I^ and Sr^I^, enriching the field of low‐valent Ae metal chemistry,[^40^, ^41^, ^42^] but also starting compounds for the functionalization of benzene. Further progress in this chemistry will be published in due course.

**Electronic Supporting Information available**: Experimental details, NMR spectra, crystallographic details^[43]^ including ORTEP plots, XYZ coordinates for calculated structures.

## Conflict of interest

The authors declare no conflict of interest.

## Supporting information

As a service to our authors and readers, this journal provides supporting information supplied by the authors. Such materials are peer reviewed and may be re‐organized for online delivery, but are not copy‐edited or typeset. Technical support issues arising from supporting information (other than missing files) should be addressed to the authors.

Supporting Information

## Data Availability

The data that support the findings of this study are available in the Supporting Information of this article.
