# Supplementary material for: Alkaline‐Earth Metal Mediated Benzene‐to‐Biphenyl Coupling
Source: Angew Chem Int Ed Engl. 2022 Dec 14;62(3):e202212463. doi: 10.1002/anie.202212463 (PMC10107259; doi:10.1002/anie.202212463)
Supplement: Supplementary file 1 — Supporting Information [file ANIE-62-0-s001.pdf]

Angewandte Chemie
Eine Zeitschrift der Gesellschaft Deutscher Chemiker

Supporting Information

Alkaline-Earth Metal Mediated Benzene-to-Biphenyl Coupling

*J. Mai, M. Morasch, D. Jędrzkiewicz, J. Langer, B. Rösch, S. Harder**

Supporting Information

Table of Contents

1. Materials and methods S2
2. Complex syntheses S4
3. Spectroscopic data S9
3.1. NMR characterization..S9
3.2. NMR reactions S24
4. GC-MS Data S36
5. Crystal structure determinations S44
6. DFT calculation S51
7. References S105

1. Materials and methods

All experiments were conducted in dry glassware under an inert nitrogen atmosphere by applying standard Schlenk techniques or gloveboxes (MBraun) using freshly dried and degassed solvents. All solvents were degassed with nitrogen, dried over activated aluminum oxide (Innovative Technology, Pure Solv 400-4-MD, Solvent Purification System), and then stored under inert atmosphere over molecular sieves (3 Å) unless noted otherwise. Deuterated benzene (C_6D_6) and cylcohexane-d_{12} were purchased from Sigma Aldrich or Deutero GmbH, degassed and dried over molecular sieves (3 Å). 2,6-CH(Et_2)-aniline was synthesized according to a slightly modified literature procedure.[S1] The following compounds were synthesized according to literature procedures: (^{DIPeP}BDI)H,[S2] [(^{DIPeP}BDI)Ca(μ-I)]$_2$,[S3] [(^{DIPeP}BDI)Ca]$_2$(C_6H_6),[S3] [(^{DIPP}BDI)Ca(μ-I)(THF)]$_2$.[S4] KN($SiMe_3$)$_2$ was purchased from Sigma-Aldrich (95%) and was used without further purification. Biphenyl (Sigma Aldrich, >99%) were obtained commercially, sublimed under reduced pressure, and stored under an N_2 atmosphere.

NMR spectra were measured on Bruker Avance III HD 400 MHz and Bruker Avance III HD 600 MHz spectrometers. Chemical shifts (δ) are denoted in ppm (parts per million), coupling constants in Hz (Hertz). For describing signal multiplicities common abbreviations are used: s (singlet), d (doublet), t (triplet), q (quartet), p (quintet), sept (septet), m (multiplet) and br (broad). Spectra were referenced to the solvent residual signal. Assignments of resonance signals in the ^{1}H and ^{13}C{^{1}H} NMR spectra were made based on two-dimensional NMR correlation (HSQC, HMBC, COSY) experiments. Elemental analysis was performed with an Hekatech Eurovector EA3000 analyzer.

GC/MS measurements were performed on a Thermo Scientific™ Trace™ 1310 gas chromatography system (carrier gas Helium) with detection by a Thermo Scientic™ ISQ™ LT Single Quadrupole mass spectrometer. A Phenomenex® ZebronTMZB-5 column of the dimensions 0.25mm x 30m with a film thickness of 0.25µm was used. The samples (1 µl) were injected with an Instant Connect-SSL Module in the S3 split mode (Injector Temperature: 280 °C, split ratio 0.9, carrier gas flow 1.2 mL/min). Temperature programs were started at 40 °C (hold 1 minute) followed by heating ramps, optimized for ideal separation, ending at 330° C (hold 5 minutes). Conditions for mass spectrometry: ion source temperature 280 °C, ionizing energy (70 eV), mass range 20-500 (m/z). The molecular identity was confirmed by comparison with entries in the NIST/EPA/NIH mass spectral library (version 2.2, built June 10, 2014) and available literature.[S5]

The ball-mill used was an ULTRA-TURRAX® Tube Drive P control from IKA. Mechanochemical reactions were performed at room temperature in 20 mL polypropylene vessel with three stainless steel balls (diameter: 5 mm, weight: 0.52 g, type: AISI 304) without temperature control.

All crystal structures have been measured on a SuperNova (Agilent) diffractometer with dual Cu and Mo microfocus sources and an Atlas S2 detector. Crystallographic data have been deposited with the Cambridge Crystallographic Data Centre as supplementary publication numbers: CCDC 2202205 [(^{DIPeP}BDI)Ca(THF)]$_2$(biphenyl) (**1**-THF), 2202206 [(^{DIPP}BDI)Ca(THF)]$_2$(biphenyl) (**2**-THF), 2202207 [(^{DIPP}BDI)Ca(THP)]$_2$(biphenyl) (**2**-THP), 2202208 [(^{DIPeP}BDI)Sr(μ-I)]$_2$ (**3**), 2202209 [(^{DIPeP}BDI)Sr]$_2$(C_6H_6) (**4**) and 2202210 [(^{DIPeP}BDI)Sr]$_2$(biphenyl) (**5**).

2. Complex syntheses

Synthesis of [(^{DIPeP}BDI)Ca(THF)]$_2$(biphenyl) (1-THF)

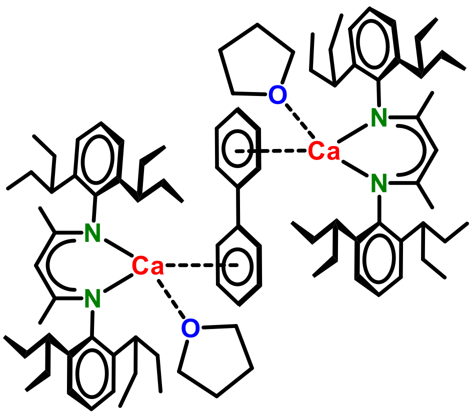

Method A: In a J. Young-Tube [(^{DIPeP}BDI)Ca]$_2$(C_6H_6) (15.1 mg, 0.012 mmol) was dissolved in C_6D_6 (550 µL). The reaction progress was monitored *via* ^{1}H NMR spectroscopy. After stirring the dark brown reaction mixture at room temperature overnight [(^{DIPeP}BDI)Ca]$_2$(biphenyl) is formed, which is confirmed by ^{1}H NMR (Figure S34), as well as by mass spectrometry, where biphenyl is observable (Figure **S49**). Due to formation of other side-products and the high solubility of all complexes, it was not possible to separate the product from the mixture. It was, however, synthesized by two independent methods (**B** and **C**).

Method B: In a J. Young-Tube [(^{DIPeP}BDI)Ca]$_2$(C_6H_6) (19.5 mg, 0.016 mmol) and biphenyl (2.47 mg, 0.016 mmol) were mixed in C_6D_6 (550 µL). The dark brown reaction mixture was stirred at room temperature and a color change to red brown was observed. The reaction progress was monitored *via* ^{1}H NMR spectroscopy, full conversion was achieved after 7 days. The solvent was removed under vacuum and the remaining solid was stripped with pentane (1 x 1 mL). The raw product is relatively pure (88% of [(^{DIPeP}BDI)Ca]$_2$(biphenyl) was formed: Figure S33). The resulting solid was dissolved in pentane (300 µL) and THF (2.6 µL, 0.032 mmol). Crystals were grown from this mixture at -20 °C after 3 days. The red brown crystals [(^{DIPeP}BDI)Ca(THF)]$_2$(biphenyl) were isolated, washed with cold pentane (-20 °C, 1 x 0.5 mL) and dried under vacuum. Yield: 8.00 mg (0.0056 mmol, 35%). The low yield is due to the very good pentane-solubility of the product.

Method C: [(^{DIPeP}BDI)Ca(μ-I)]$_2$ (300 mg, 0.431 mmol), KC_8 (348 mg, 2.58 mmol) and biphenyl (33.1 mg, 0.215 mmol) were suspended in methylcyclohexane (8 mL) and stirred at room temperature for 7 days. The mixture was filtered, and the red brown solution was evaporated to dryness. The raw product is relatively pure (Figure S32). The crude product was stripped with pentane (1 x 1.5 mL), dissolved in pentane (500 µL), and filtered. Tetrahydrofuran (35.0 µL, 0.431 mmol) was slowly added to the mother liquor. Leaving it standing overnight at -20 °C gave red brown crystals of [(^{DIPeP}BDI)Ca(THF)]$_2$(biphenyl) suitable for X-ray diffraction analysis. Crystals were isolated by decantation, washed with cold pentane (-20 °C, 1 x 0.5 mL) and dried *in vacuo*. Yield: 52 mg (0.036 mmol, 17%). The low yield is due to the very good pentane-solubility of the product.

^{1}**H NMR** (C_6D_6, 600.13 MHz, 298 K): δ = 7.03 – 7.01 (m, 12H, C**H**-arom), 4H, C**H**-biphenyl), 4.82 (s, 2H, C**H**-backbone), 3.98 – 3.94 (m, 8H, THF α-C**H₂**), 3.71 (br s, 4H, C**H**-biphenyl), 3.03 (br s, 8H, C**H**), 1.75 – 1.70 (m, 8H, C**H₂**), 1.70 (s, 12H, C**H₃**-backbone), 1.68 – 1.61 (m, 24H, C**H₂**), 1.55 – 1.53 (m, 8H, THF β-C**H₂**), 0.95 (t, $^{3}J_{HH}$ = 7.3 Hz, 24H, C**H₃**), 0.84 (t, $^{3}J_{HH}$ = 7.4 Hz, 24H, C**H₃**) ppm.

^{13}**C NMR** (C_6D_6, 151 MHz, 298 K): δ = 165.0 (**C**N-backbone), 148.2 (**C**-arom), 137.2 (**C**-arom), 124.9 (**C**-arom), 121.5 (**C**-arom), 92.3 (**C**H-backbone), 67.9 (THF α-**C**H_2), 39.3 (**C**H), 25.4 (THF β-**C**H_2), 24.8 (**C**H_2), 24.4 (**C**H_2), 23.9 (**C**H_3-backbone), 9.9 (**C**H_3), 9.6 (**C**H_3) ppm. The signals of biphenyl are not visible.

Elemental analysis Calculated for $C_{94}H_{140}Ca_2N_4O_2$ (M = 1438.34 g/mol): C 78.50, H 9.81, N 3.90 %. Found: C 78.31, H 9.83, N 3.94 %.

Synthesis of [(^{DIPP}BDI)Ca(THF)]$_2$(biphenyl) (2-THF)

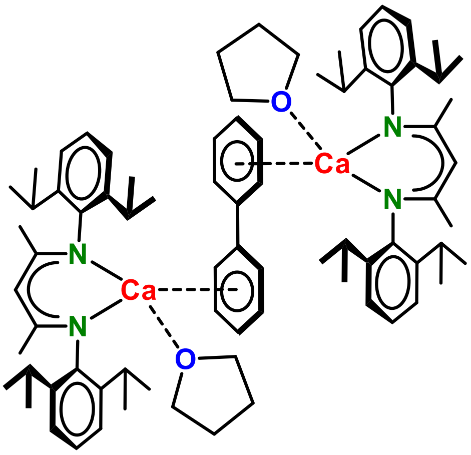

[(^{DIPP}BDI)Ca(μ-I)(THF)]$_2$ (0.131 g, 0.100 mmol) and K/KI (5% w/w, 0.468 g, 0.300 mmol, 3 eq. K) were placed in a vessel equipped with 3 stainless steel balls and ground over 60 minutes with ball-mill speed 1800 rpm (30 Hz). The resulting deep purple powder was extracted with benzene (2 x 3 mL). The dark red extract was left at room temperature over 1 h to give brownish black block-like crystals suitable for X-ray diffraction analysis. The crystals which are highly insoluble in aromatic solvents were isolated by decantation, washed with benzene (3 x 5 mL) and dried *in vacuo.* Yield: 63.0 mg (0.052 mmol, 52%).

^{1}**H NMR** (C_6D_6, 600.13 MHz, 298 K): δ = 7.11 – 7.05 (m, 12H, Ar**H**), 4.95 (br s, 4H, C**H**-biphenyl), 4.87 (s, 2H, C**H**-backbone), 3.78 (br s, 4H, C**H**-biphenyl), 3.65 – 3.52 (m, 8H, THF α-C**H₂**), 3.38 – 3.19 (m, 8H, C**H**), 1.75 (s, 12H, C**H₃**-backbone), 1.46 – 1.39 (m, 8H, THF β-C**H₂**), 1.26 (d, ^{3}J = 6.9 Hz, 24H, C**H₃**), 1.22 (d, ^{3}J = 6.9 Hz, 12H, C**H₃**) ppm.

^{13}**C NMR:** Due to the very low solubility, no ^{13}C NMR characterization was possible.

Elemental analysis for $C_{78}H_{108}N_4O_2Ca_2$ (M = 1213.90 g/mol): C 77.18; H 8.97; N 4.62 %. Found: C 77.07; H 9.53; N 4.69 %. Due to the extreme air-sensitivity of the complex, this represents the best CHN analysis we can provide.

Synthesis of [(^{DIPP}BDI)Ca(μ-I)(THP)]$_2$

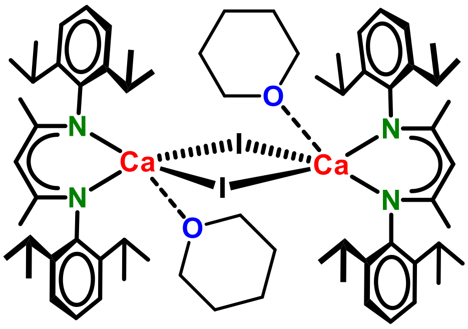

[(^{DIPP}BDI)Ca(μ-I)(THF)]$_2$ (1.97 g, 1.50 mmol) was dissolved in THP (20 mL) and dried under *vacuo* at 30 °C for 2 h, then dissolved again in THP (20 mL) and dried under *vacuo* at 30 °C for 6 h resulting in [(^{DIPP}BDI)Ca(μ-I)(THP)]$_2$ as white powder in quantitative yield (2.01 g, 1.50 mmol, 100%).

^{1}**H NMR** (C_6D_6, 600.13 MHz, 298K): δ = 7.15 – 7.11 (m, 12H, Ar**H**), 4.82 (s, 2H, C**H**-backbone), 3.74 – 3.63 (m, 8H, THP α-C**H$_2$**), 3.26 (sept, ^{3}J = 6.9 Hz, 8H, C**H**), 1.67 (s, 12H, C**H$_3$**-backbone), 1.39 (d, ^{3}J = 6.9 Hz, 24H, C**H$_3$**), 1.31 – 1.26 (m, 8H, THP β-C**H$_2$**), 1.24 (d, ^{3}J = 6.9 Hz, 24H, C**H$_3$**), 1.21 – 1.13 (m, 4H, THP γ-C**H$_2$**) ppm.

^{13}**C NMR** (C_6D_6, 151 MHz, 298K): δ = 166.4 (**C**N-backbone), 146.5 (**C**-arom), 142.0 (**C**-arom), 124.8 (**C**-arom), 124.0 (**C**-arom), 94.7 (**C**H-backbone), 70.2 (THP α-**C**H$_2$), 28.6 (**C**H), 25.9 (**C**H$_3$), 25.8 (THP β-**C**H$_2$), 25.0 (**C**H$_3$), 24.8 (**C**H$_3$), 22.7 (THP γ-**C**H$_2$) ppm.

Elemental analysis Calculated for $C_{68}H_{102}N_4O_2Ca_2I_2$ (M = 1341.56 g/mol): C 60.88; H 7.66; N 4.18 %. Found: C 60.58; H 7.76; N 4.10 %.

Synthesis of [(^{DIPP}BDI)Ca(THP)]$_2$(biphenyl) (2-THP)

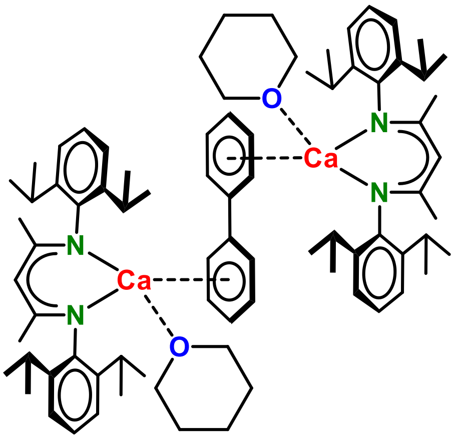

[(^{DIPP}BDI)Ca(μ-I)(THP)]$_2$ (0.134 g, 0.100 mmol) and K/KI (5% w/w, 0.468 g, 0.300 mmol, 3 eq. K) were placed in a vessel equipped with 3 stainless steel balls and ground over 90 minutes with ball-mill speed 1800 rpm (30 Hz). The resulting dark purple powder was extracted with benzene (2 x 3 mL). The dark red extract was left at room temperature over 1 h to give brownish black block-like crystals suitable for X-ray diffraction analysis. The crystals which are highly insoluble in aromatic solvents were isolated by decantation, washed with benzene (3 x 5 mL) and dried *in vacuo*. Yield: 67.0 mg (0.054 mmol, 54%).

NMR: Due to the very low solubility, no NMR characterization was possible.

Elemental analysis for $C_{80}H_{112}N_4O_2Ca_2$ (M = 1241.96 g/mol, calculated with one co-crystallized molecule of benzene): C 78.25; H 9.01; N 4.24 %. Found: C 78.90; H 9.09; N 4.42 %. Due to the extreme air-sensitivity of the complex, this represents the best CHN analysis we can provide.

Synthesis of [(^{DIPeP}BDI)Sr(μ-I)]$_2$ (3)

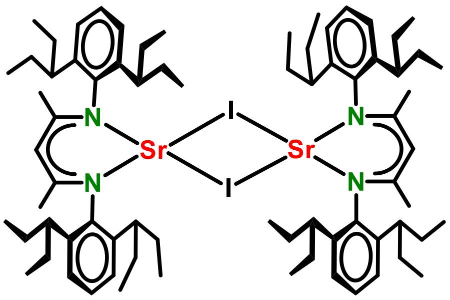

$KN(SiMe_3)_2$ (1.21 g, 6.05 mmol) and (^{DIPeP}BDI)H (3.00 g, 5.65 mmol) were dissolved in hexane (30 mL) and stirred overnight at 65 °C. After cooling to room temperature, (^{DIPeP}BDI)K was isolated as a white solid *via* centrifugation and dried *in vacuo* at 45 °C. Subsequently, SrI_2 (2.13 g, 6.22 mmol) and Et_2O (40 mL) were added. The suspension was stirred overnight at room temperature and all volatiles were removed *in vacuo*. The solid residue was extracted with hexane (2 x 30 mL) to afford a brown solution. After the solvent was evaporated, the solid residue was dried *in vacuo* to afford the product as a beige powder (3.73 g, 2.51 mmol, 89%).

^{1}H NMR (C_6D_6, 600.13 MHz, 298 K): δ = 7.13 – 7.08 (m, 12H, C**H**-arom), 4.83 (s, 2H, C**H**-backbone), 2.69 (p, $^3J_{HH}$ = 6.2 Hz, 8H, C**H**), 1.75 (s, 12H, C**H$_3$**-backbone), 1.72 – 1.60 (m, 32H, C**H$_2$**), 1.03 (t, $^3J_{HH}$ = 7.3 Hz, 24H, C**H$_3$**), 0.83 (t, $^3J_{HH}$ = 7.4 Hz, 24H, C**H$_3$**) ppm.

^{13}C NMR (C_6D_6, 151 MHz, 298 K): δ = 164.1 (**C**N-backbone), 145.7 (**C**-arom), 138.7 (**C**-arom), 125.7 (**C**-arom), 123.7 (**C**-arom), 93.8 (**C**H-backbone), 41.8 (**C**H), 28.7 (**C**H$_2$), 25.8 (**C**H$_2$), 24.1 (**C**H$_3$-backbone), 13.3 (**C**H$_3$), 11.2 (**C**H$_3$) ppm.

Elemental analysis calculated for $C_{74}H_{114}I_2N_4Sr_2$ (M = 1488.80 g/mol): C 59.70, H 7.72, N 3.76 %. Found: C 60.06, H 7.88, N 3.80 %.

Synthesis of [(^{DIPeP}BDI)Sr]$_2$(C_6H_6) (4)

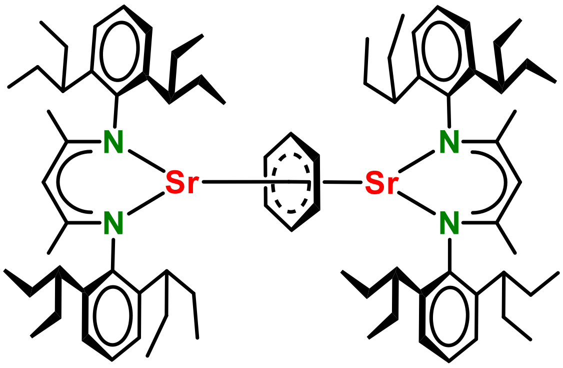

[(^{DIPeP}BDI)Sr(μ-I)]$_2$ (200 mg, 0.134 mmol) and KC_8 (103 mg, 0.763 mmol) were suspended in benzene (2 mL) and cooled to 10 °C. After stirring overnight, the reaction mixture was cooled to -15 °C and the frozen solvent sublimated *in vacuo* to afford a black solid residue. The residue was allowed to reach room temperature and was extracted with cold pentane (4 mL). The solvent was removed *in vacuo* and the solid residue stripped with pentane (4 mL) to afford an essentially pure fine black powder (61% yield). Subsequently, the black solid was dissolved in pentane (700 µL), filtrated and cooled to -20 °C. Crystals suitable for X-ray diffraction analysis were obtained after two weeks at this temperature. The crystals were isolated by decantation of the supernatant and washed with cold pentane (1 mL) and briefly dried *in vacuo* to afford [(^{DIPeP}BDI)Sr]$_2$(C_6H_6) as pitch-black crystals. Crystallized yield: 44.0 mg

(0.0351 mmol, 26%). The low yield is due to the very good pentane-solubility of the product. Crystals are highly air-sensitive but also decompose within weeks when stored under nitrogen in the freezer of a glovebox (-20 °C).

^{1}H NMR: The complex is paramagnetic which leads to signal broadening and the resonances could not be assigned (Figure S22).

Elemental analysis calculated for $C_{80}H_{120}N_4Sr_2$ (M = 1313.11 g/mol): C 73.18, H 9.21, N 4.27 %. Found: C 73.56, H 9.17, N 4.50 %.

Synthesis of [(^{DIPeP}BDI)Sr]$_2$(biphenyl) (5)

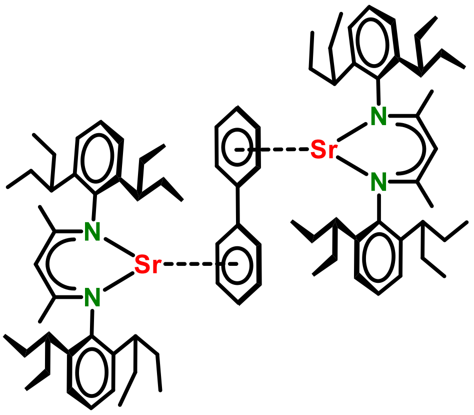

Method A: [(^{DIPeP}BDI)Sr(μ-I)]$_2$ (200 mg, 0.134 mmol), biphenyl (21.0 mg, 0.136 mmol) and KC_8 (108 mg, 0.804 mmol) were suspended in methylcyclohexane (3 mL) and stirred at room temperature. After 2 days, the reaction mixture was filtered, and the solvent removed *in vacuo*. The dark brown solid residue was stripped with pentane (2 x 3 mL) to afford a dark-brown powder, which was dissolved in pentane (700 µL), filtered, and slowly cooled to -20 °C. After one week black crystals of [(^{DIPeP}BDI)Sr]$_2$(biphenyl) suitable for X-ray diffraction analysis were obtained. The crystals were isolated by decantation of the supernatant, washed with cold pentane (1 mL), and dried briefly *in vacuo.* Yield: 25.0 mg (0.018 mmol, 13%).

Method B: [(^{DIPeP}BDI)Sr]$_2$(C_6H_6) (15.0 mg, 0.010 mmol) was dissolved in C_6D_6 (2.75 mL) and the black reaction mixture was stirred for 10 minutes. An immediate color change from black to dark-brown was observed and selective, nearly quantitative, conversion to [(^{DIPeP}BDI)Sr]$_2$(biphenyl) was confirmed *via* ^{1}H NMR spectroscopy (see Figure S38-S39).

^{1}H NMR (C_6D_6, 600.13 MHz, 298 K): δ = 6.97 (s, 12H, C**H**-arom), 5.01 (s, 2H, C**H**-backbone), 4.83 (br s, 4H, C**H**-biphenyl), 3.14 (br s, 6H, C**H**-biphenyl), 2.88 – 2.86 (m, 8H, C**H**), 1.90 (s, 12H, C**H$_3$**-backbone), 1.73 – 1.60 (m, 24H, C**H$_2$**), 1.48 – 1.43 (m, 8H, C**H$_2$**), 1.04 (t, $^{3}J_{HH}$ = 7.3 Hz, 24H, C**H$_3$**), 0.84 (t, $^{3}J_{HH}$ = 7.5 Hz, 24H, C**H$_3$**) ppm.

^{13}C NMR (C_6D_6, 151 MHz, 298 K): δ = 163.9 (**C**N-backbone), 147.0 (**C**-arom), 138.8 (**C**-arom), 125.4 (**C**-arom), 123.0 (**C**-arom), 93.7 (**C**H-backbone), 41.7 (**C**H), 28.5 (**C**H$_2$), 25.5 (**C**H$_2$), 24.1 (**C**H$_3$-backbone), 12.4 (**C**H$_3$), 11.2 (**C**H$_3$) ppm. The signals of biphenyl are not visible.

Elemental analysis calculated for $C_{86}H_{124}N_4Sr_2$ + co-crystallized 1.2 eq. pentane (C_5H_{12}) (M = 1475.79 g/mol): C 74.79, H 9.38, N 3.83 %. Found: C 75.10, H 9.21, N 3.87 %.

3. Spectroscopic data

3.1. NMR characterization

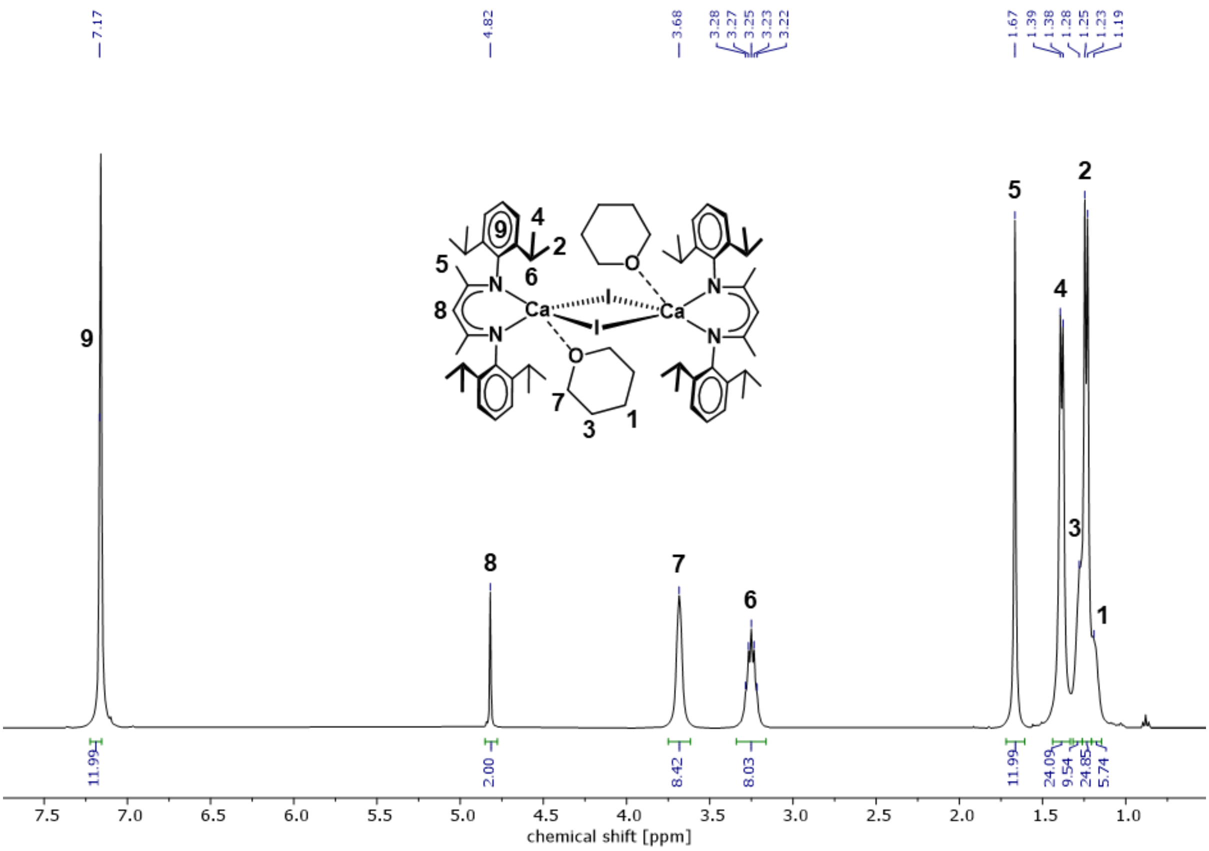

Figure S1. ^{1}H NMR (600.13 MHz, 298 K, C_6D_6) of [(^{DIPP}BDI)Ca(μ-I)(THP)]$_2$.

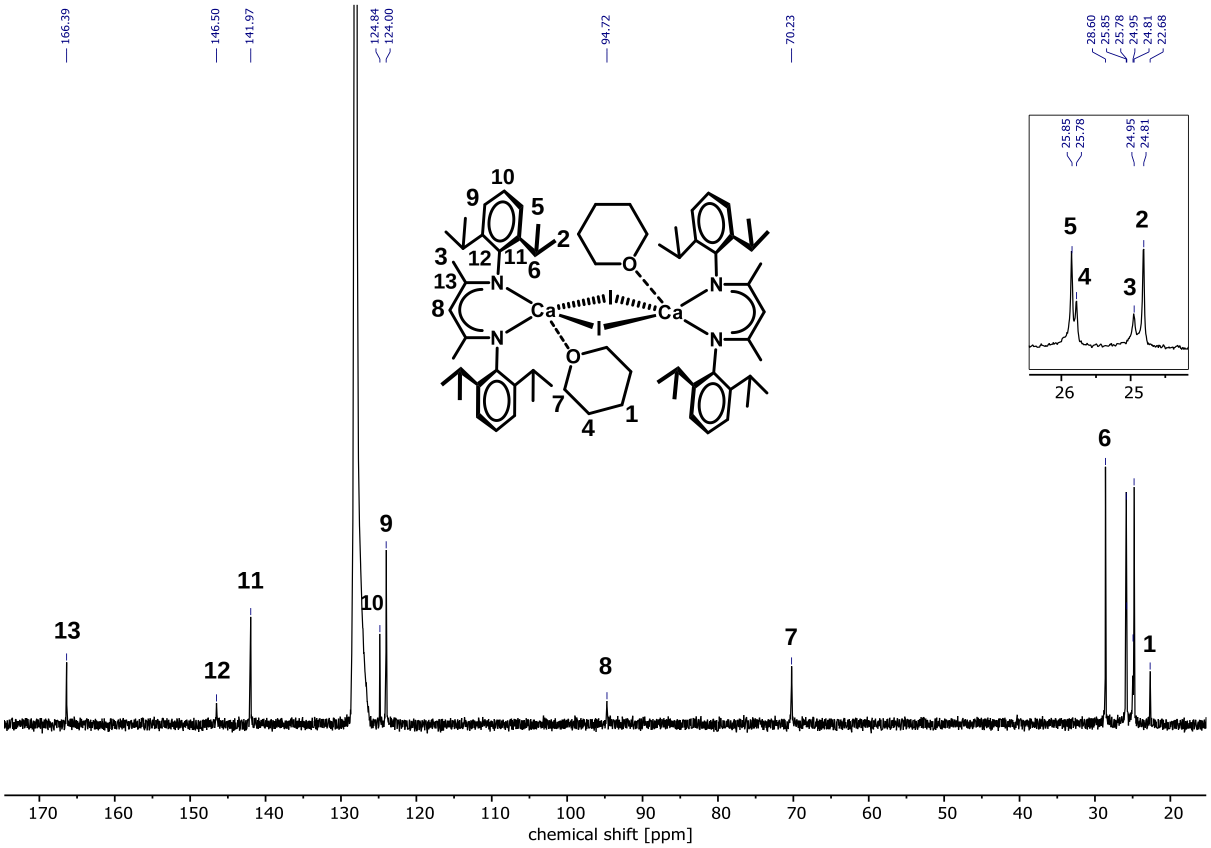

Figure S2. ^{13}C NMR (151 MHz, 298 K, C_6D_6) of [(^{DIPP}BDI)Ca(μ-I)(THP)]$_2$.

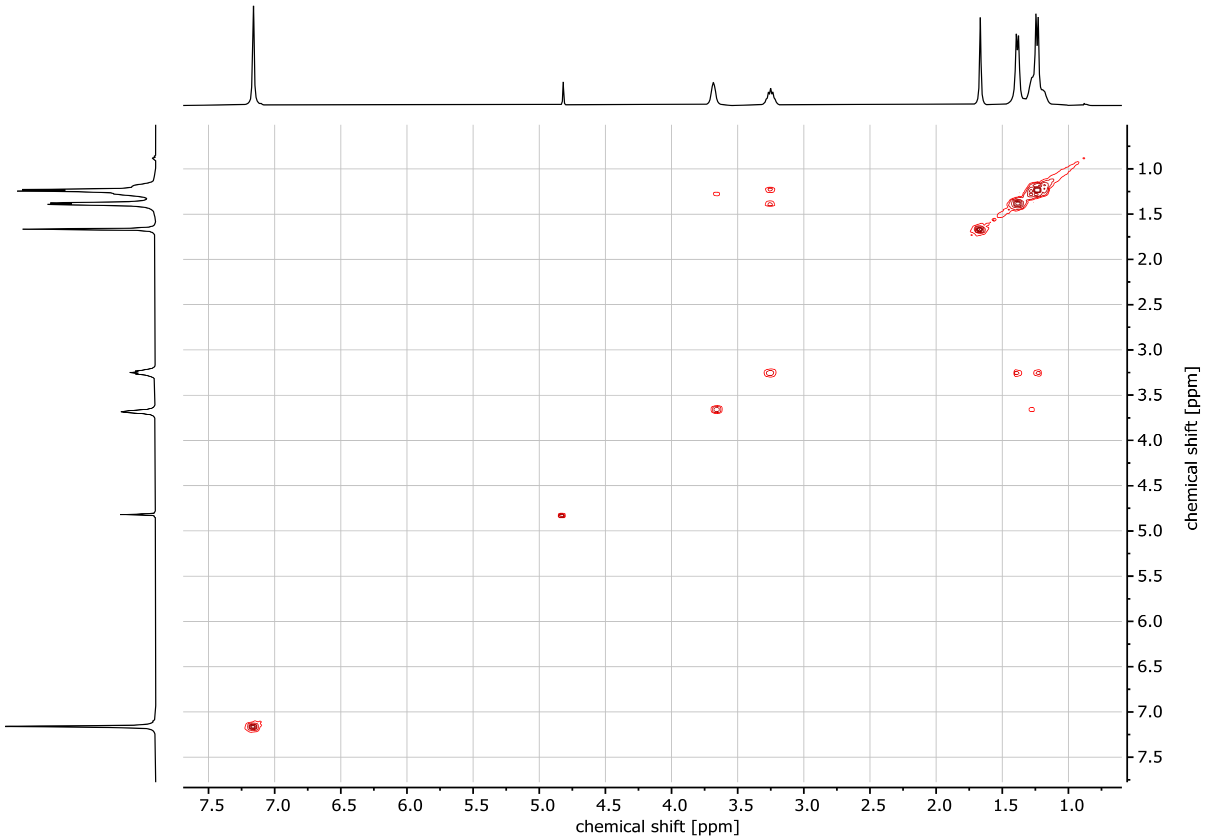

Figure S3. 2D-COSY NMR spectrum (600.13 MHz, 298 K, C_6D_6) of [(^{DIPP}BDI)Ca(μ-I)(THP)]$_2$.

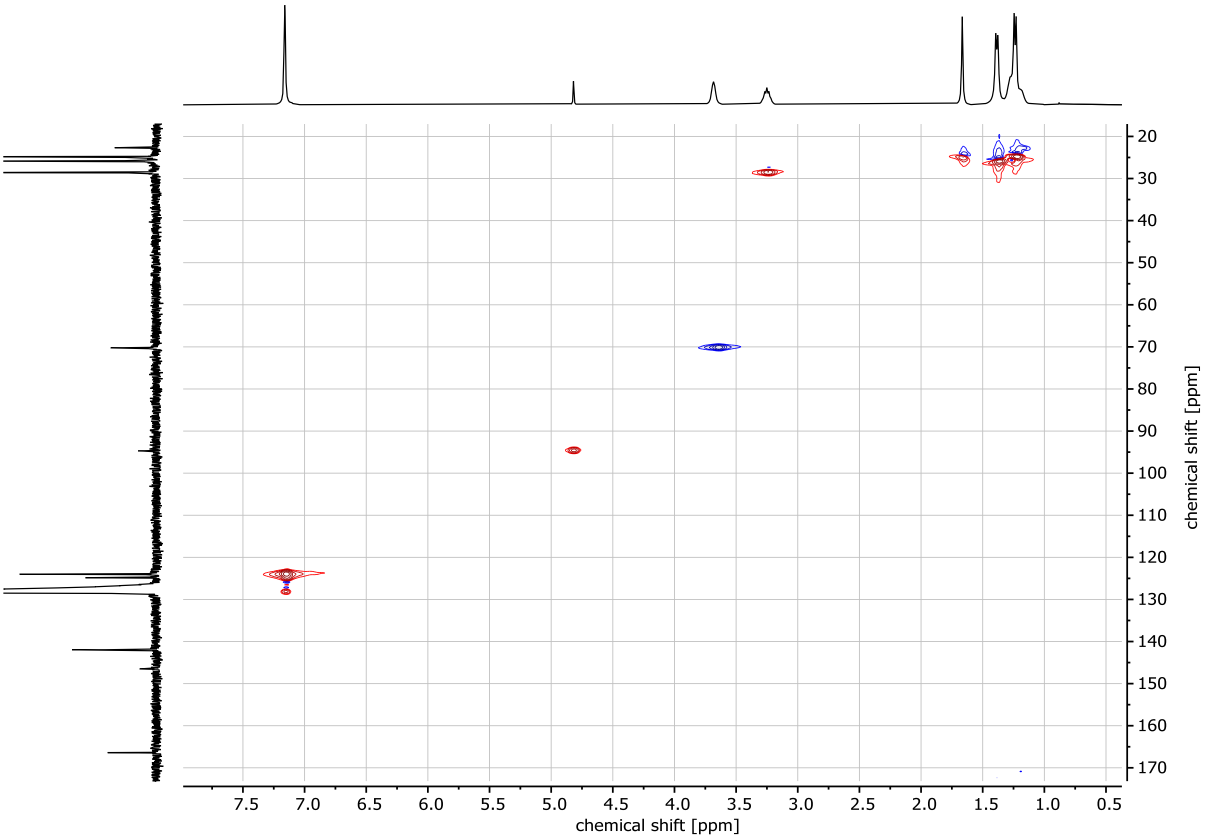

Figure S4. 2D-HSQC NMR spectrum of [(^{DIPP}BDI)Ca(μ-I)(THP)]$_2$.

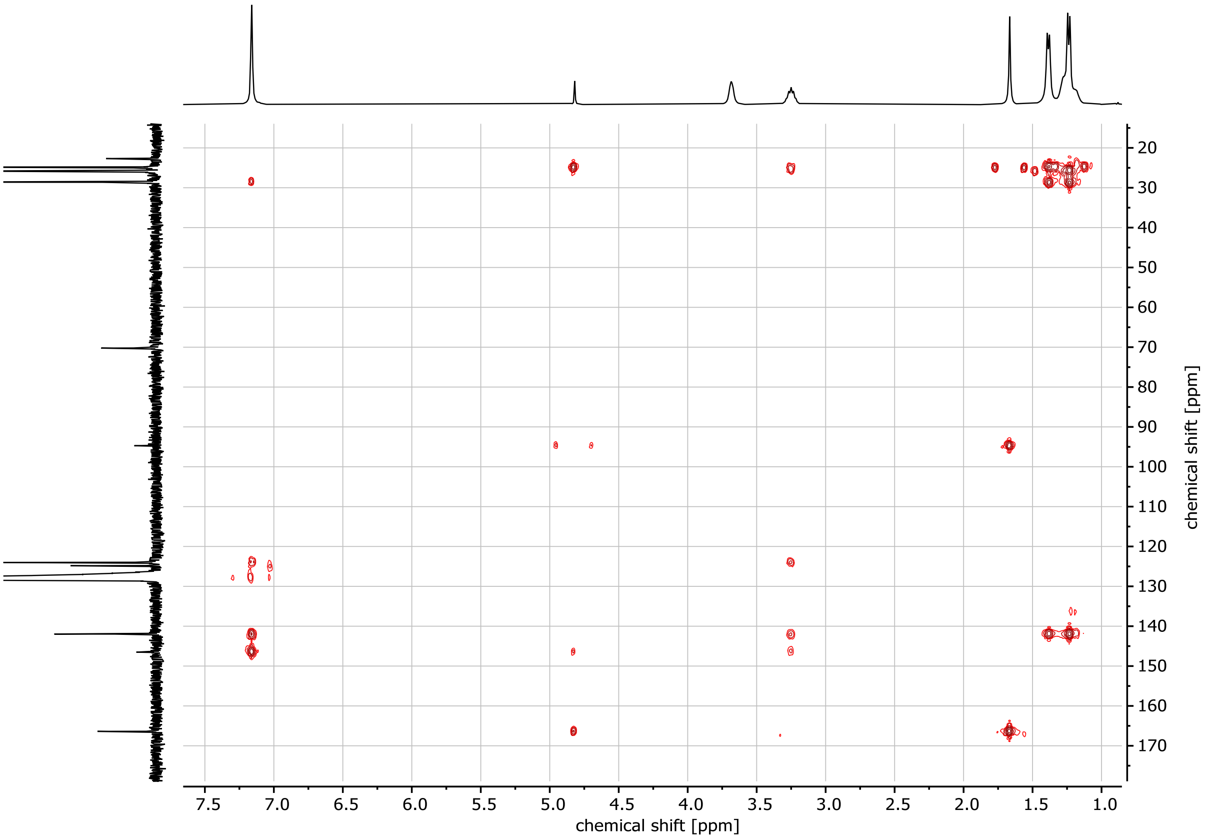

Figure S5. 2D-HMBC NMR spectrum of [(^{DIPP}BDI)Ca(μ-I)(THP)]$_2$.

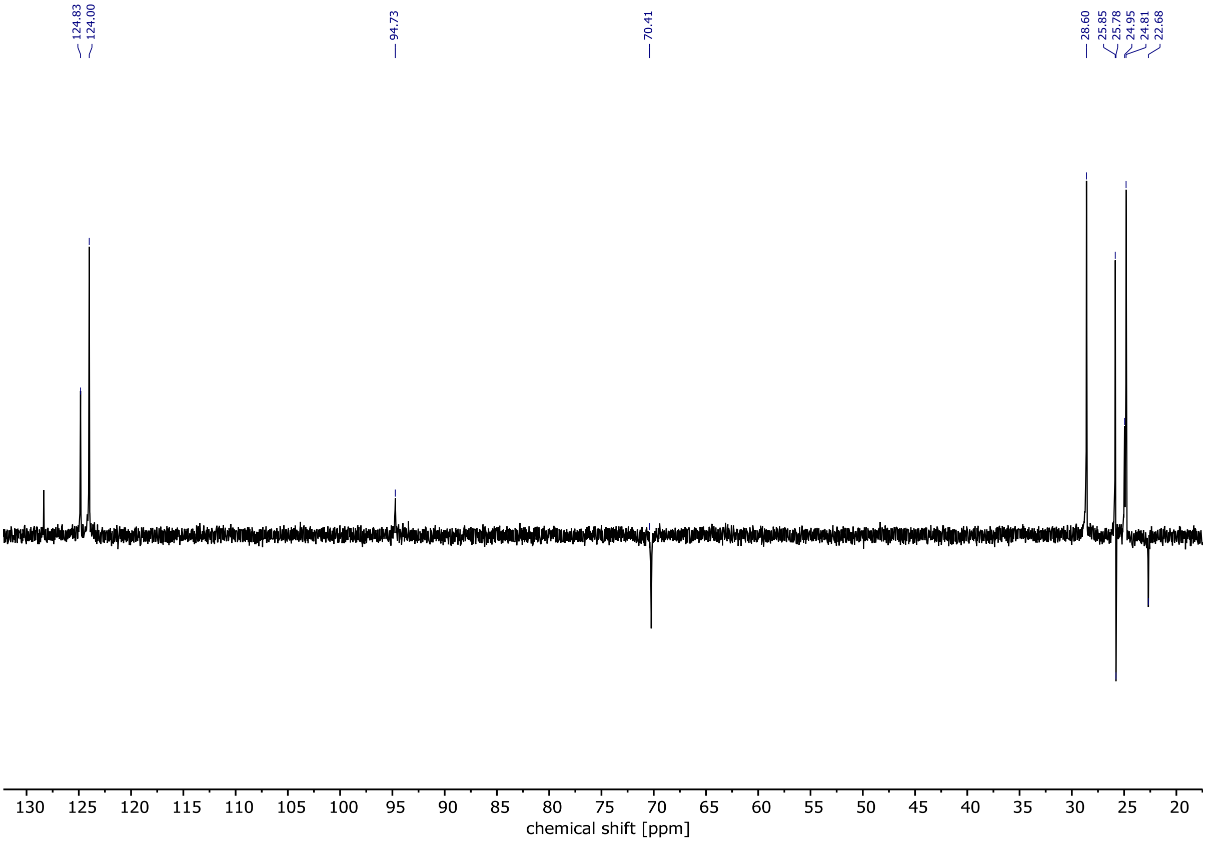

Figure S6. ^{13}C (DEPT 135) NMR spectrum of $[(^{DIPP}BDI)Ca(\mu\text{-}I)(THP)]_2$.

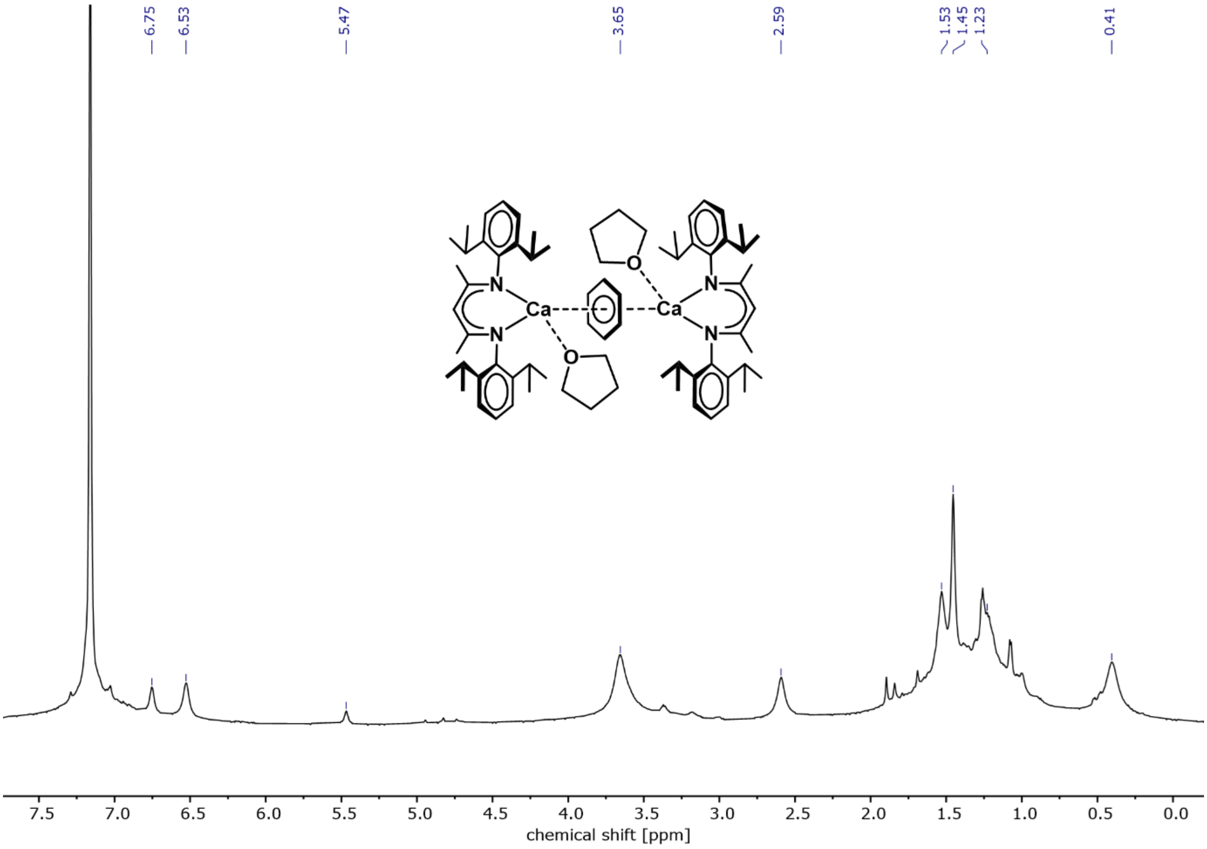

Figure S7. ^{1}H NMR (600.13 MHz, 298 K, C_6D_6) of the intermediate $[(^{DIPP}BDI)Ca(THF)]_2(C_6H_6)$. Due to paramagnetic behavior and very broad signals, no assignment was possible.

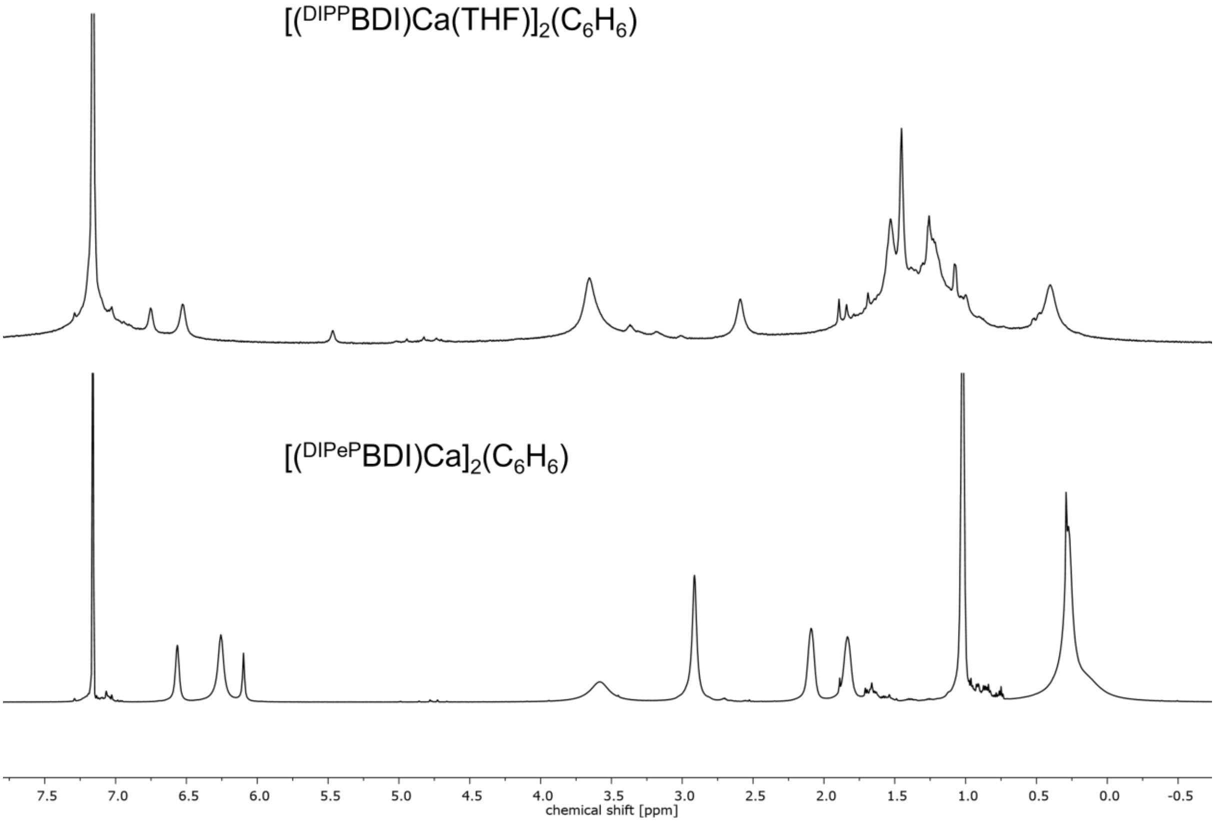

Figure S8. ^{1}H NMR (600.13 MHz, 298 K, C_6D_6) comparison of intermediate [(^{DIPP}BDI)Ca(THF)]$_2$(C_6H_6) and the previously reported complex [(^{DIPeP}BDI)Ca]$_2$(C_6H_6).[S3]

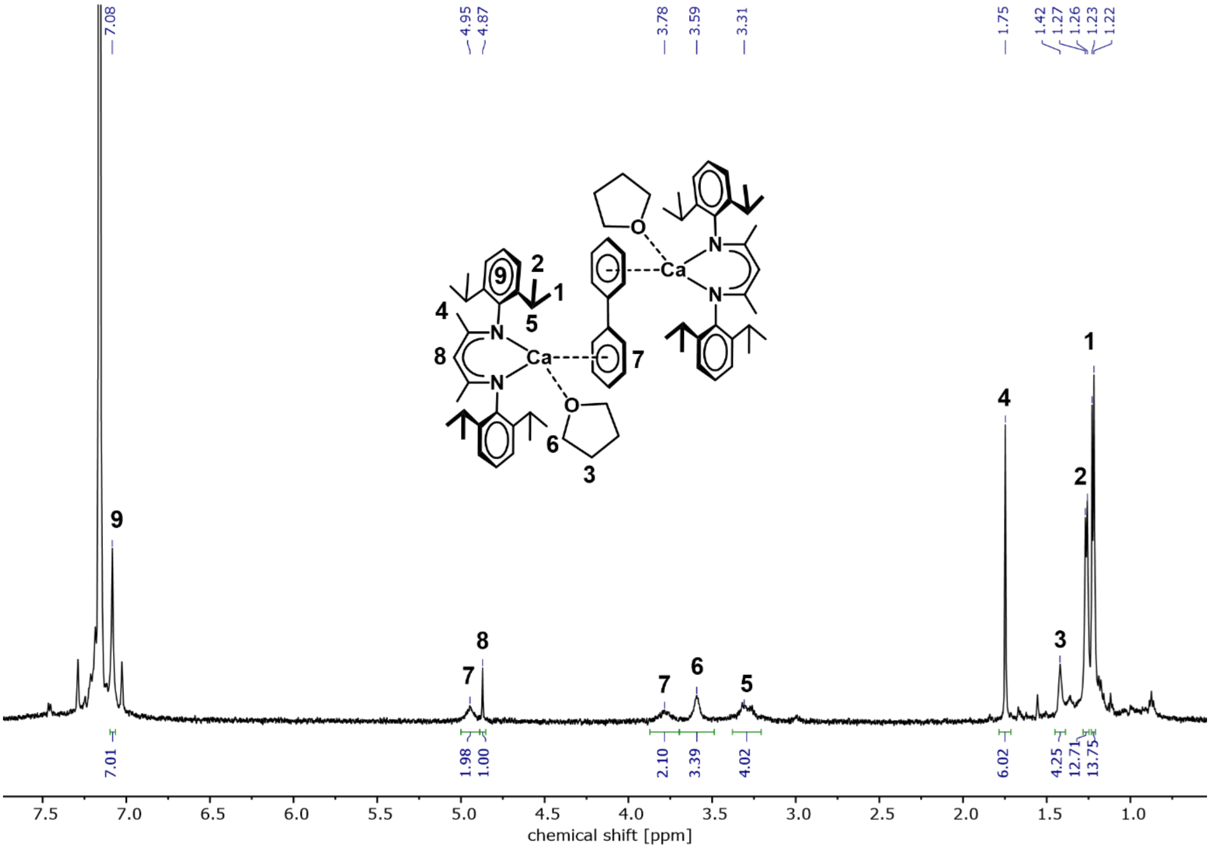

Figure S9. ^{1}H NMR (600.13 MHz, 298 K, C_6D_6) of [(^{DIPP}BDI)Ca(THF)]$_2$(biphenyl). Due to low solubility no further NMR characterization was possible.

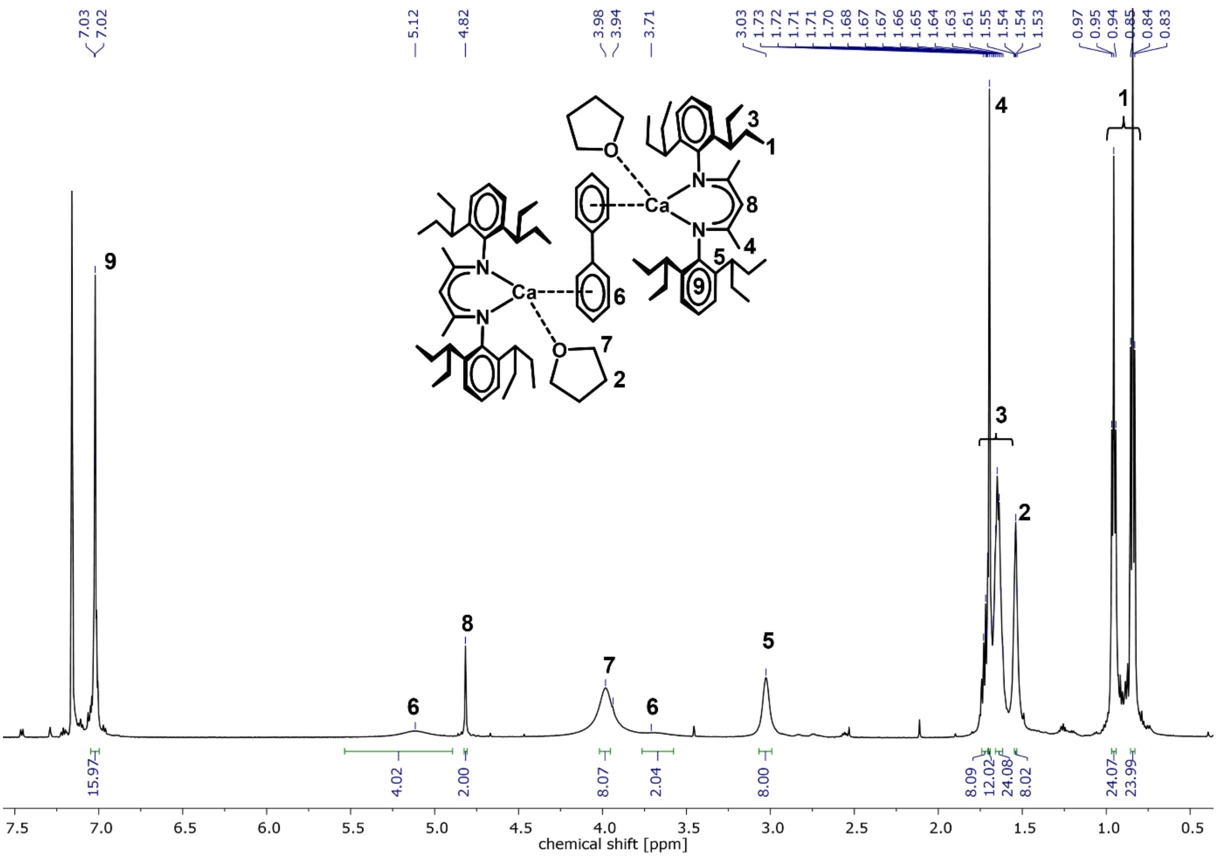

Figure S10. ^{1}H NMR (600.13 MHz, 298 K, C_6D_6) of [(^{DIPeP}BDI)Ca(THF)]$_2$(biphenyl).

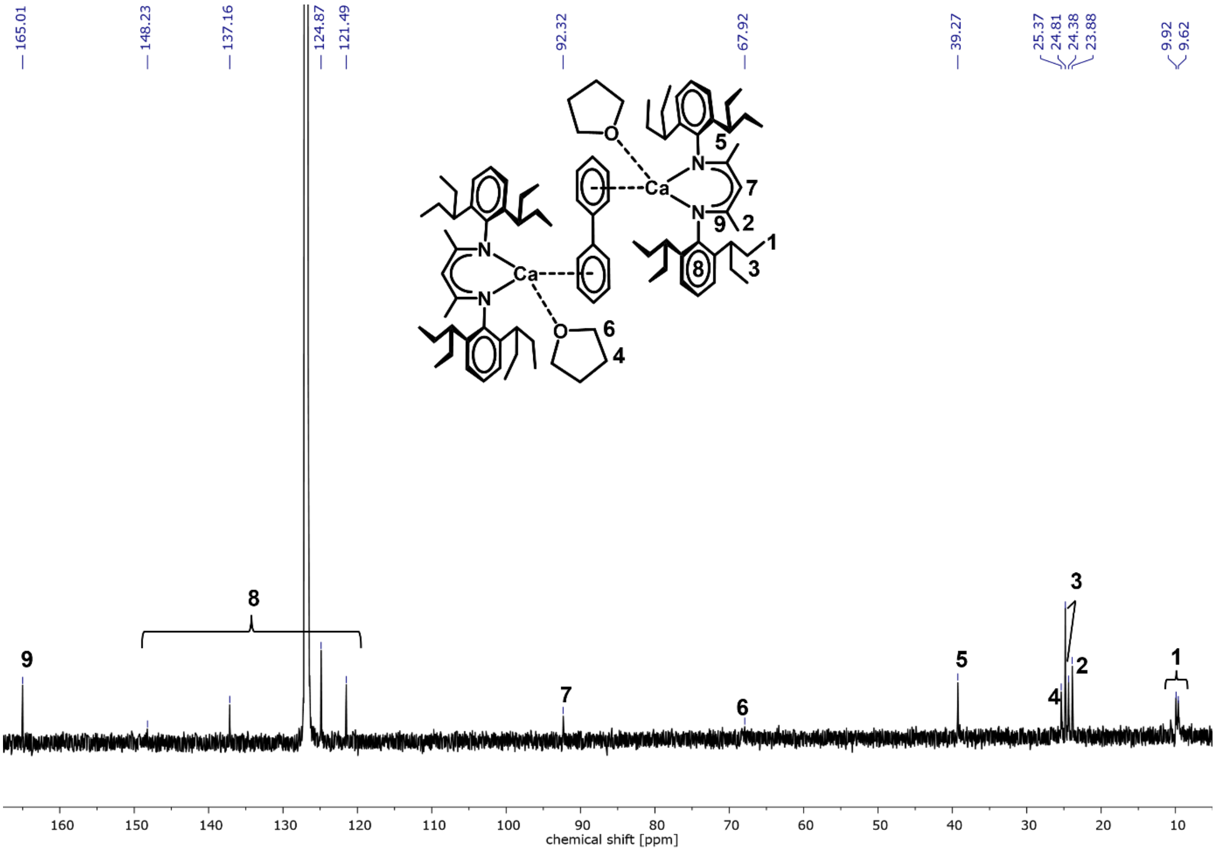

Figure S11. ^{13}C NMR (151 MHz, 298 K, C_6D_6) of [(^{DIPeP}BDI)Ca(THF)]$_2$(biphenyl). Biphenyl signals not visible.

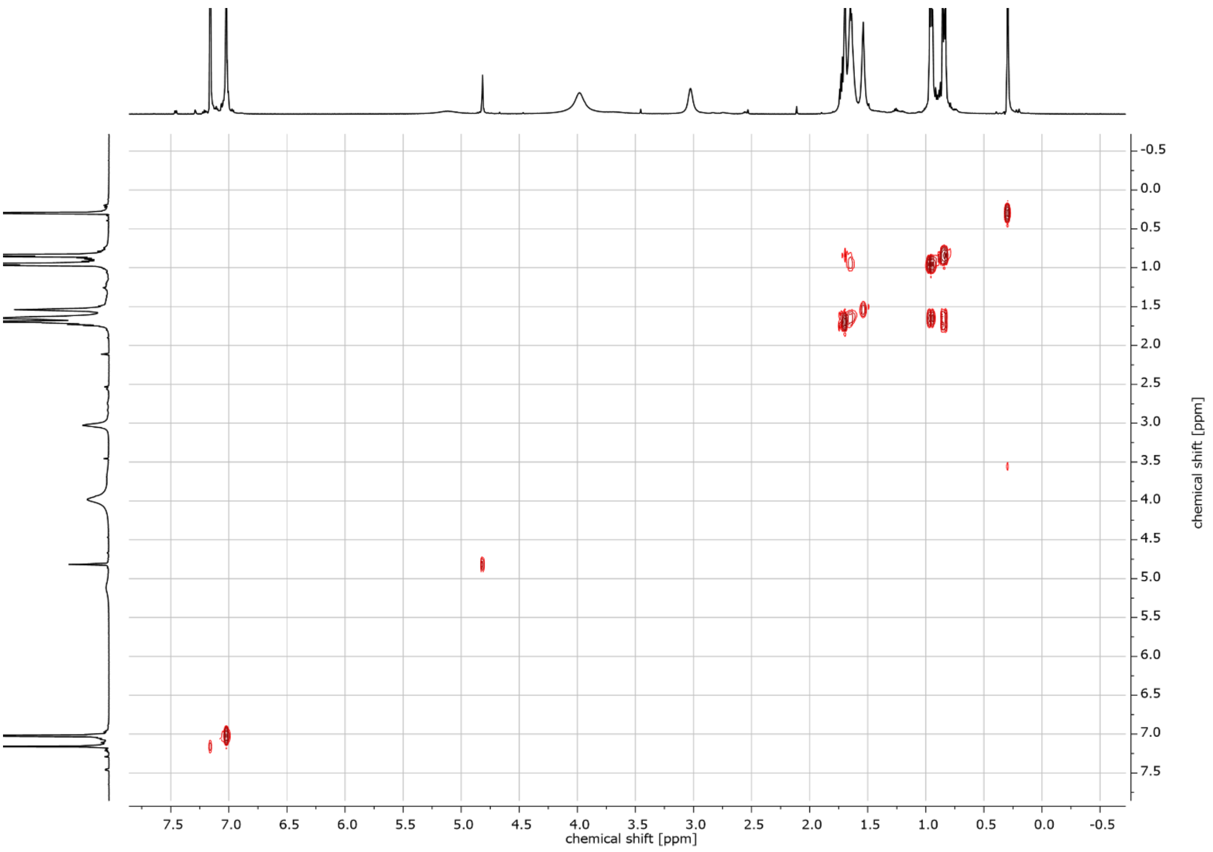

Figure S12. 2D-COSY NMR spectrum (600.13 MHz, 298 K, C_6D_6) of [(^{DIPeP}BDI)Ca(THF)]$_2$(biphenyl).

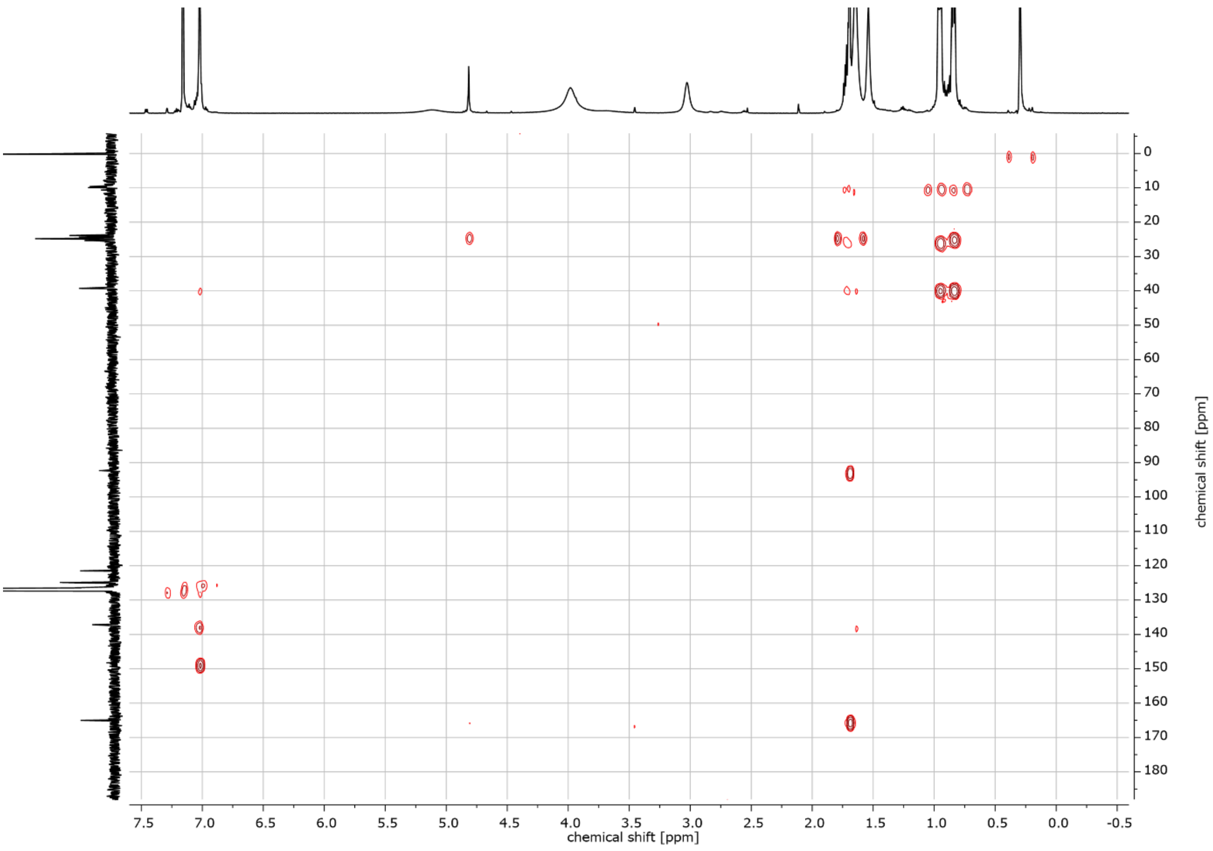

Figure S13. 2D-HMBC NMR spectrum of [(^{DIPeP}BDI)Ca(THF)]$_2$(biphenyl).

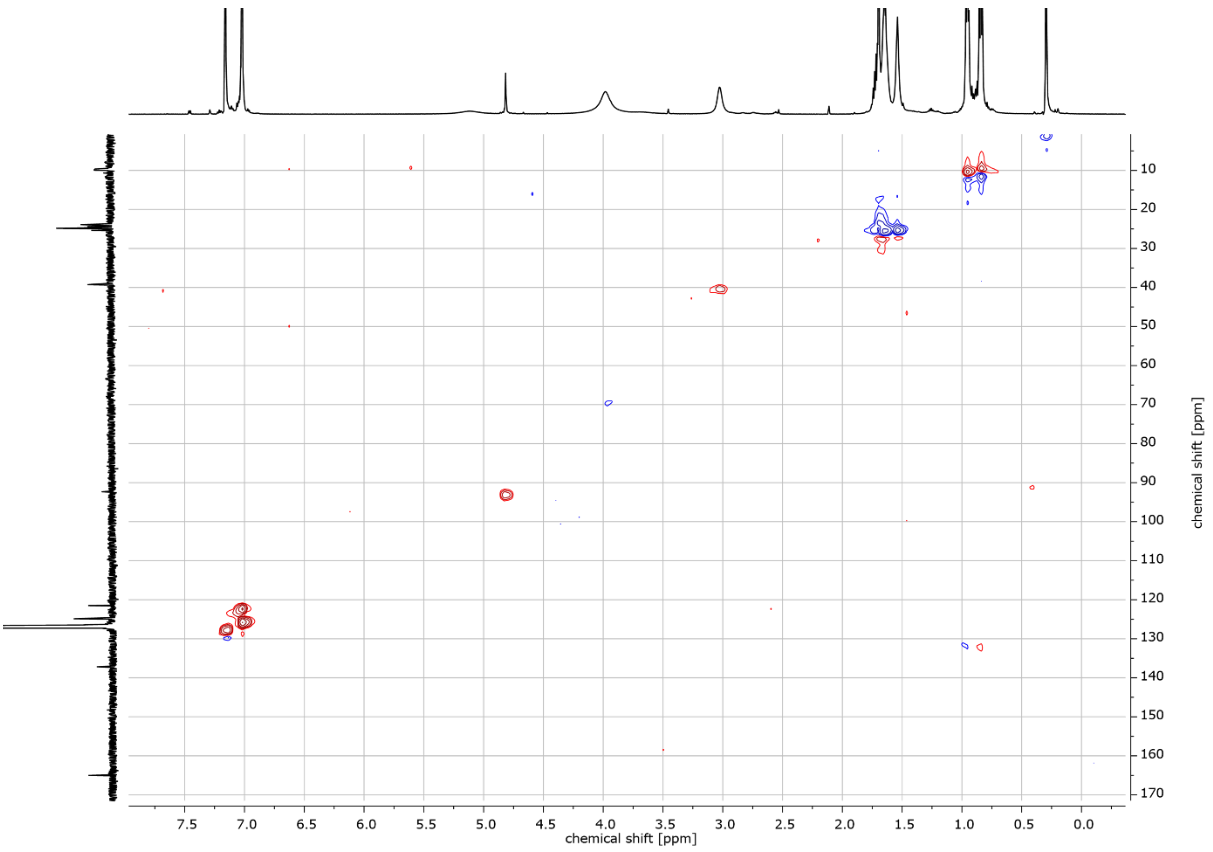

Figure S14. 2D-HSQC NMR spectrum of [(^{DIPeP}BDI)Ca(THF)]$_2$(biphenyl).

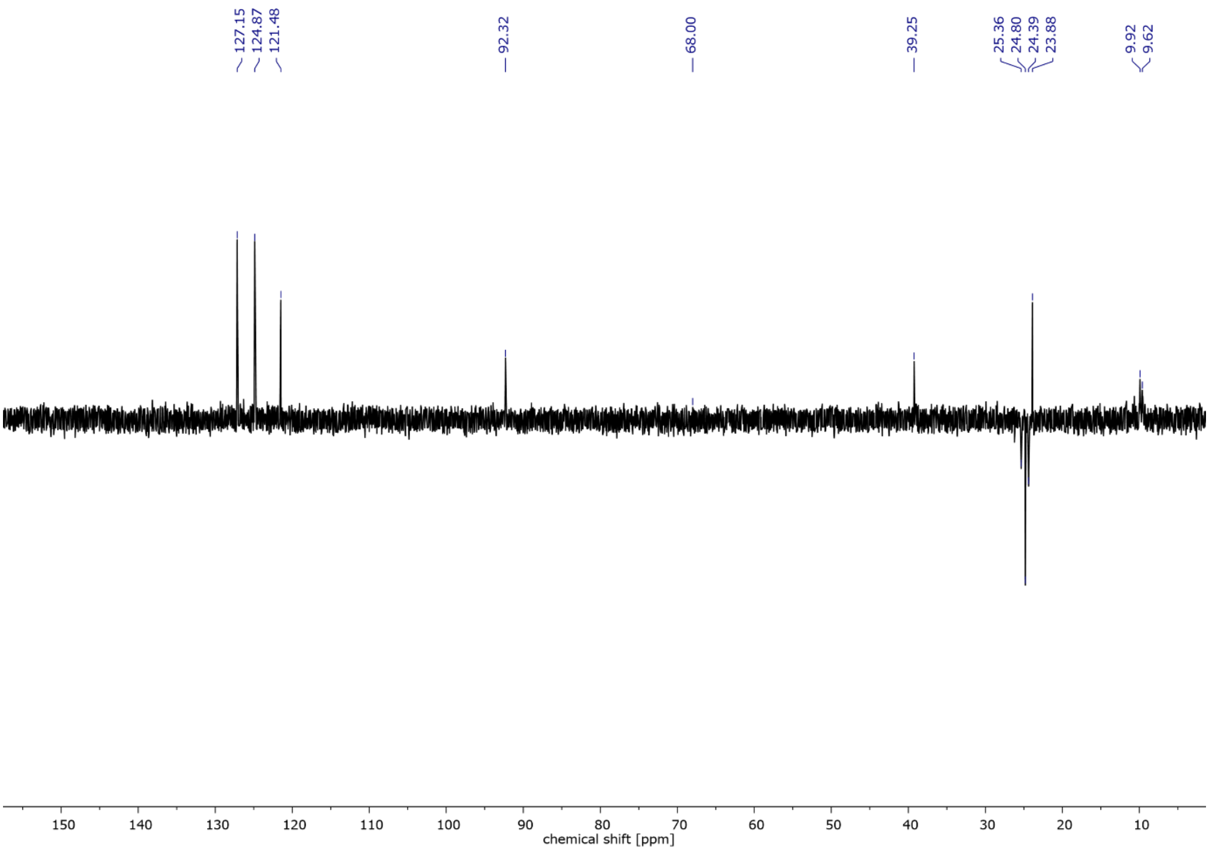

Figure S15. ^{13}C(DEPT 135) NMR spectrum of [(^{DIPeP}BDI)Ca(THF)]$_2$(biphenyl).

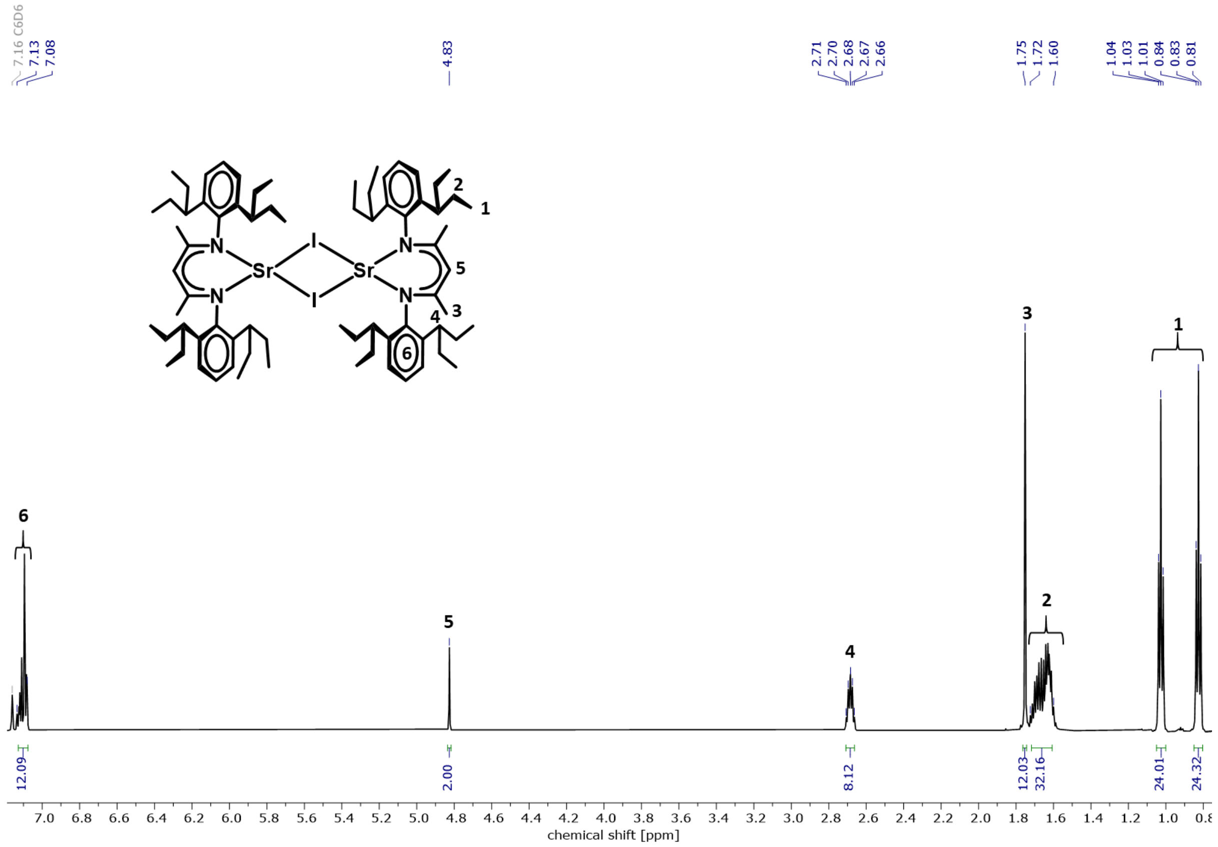

Figure S16. ^{1}H NMR (600.13 MHz, 298 K, C_6D_6) of [(^{DIPeP}BDI)Sr(μ-I)]$_2$.

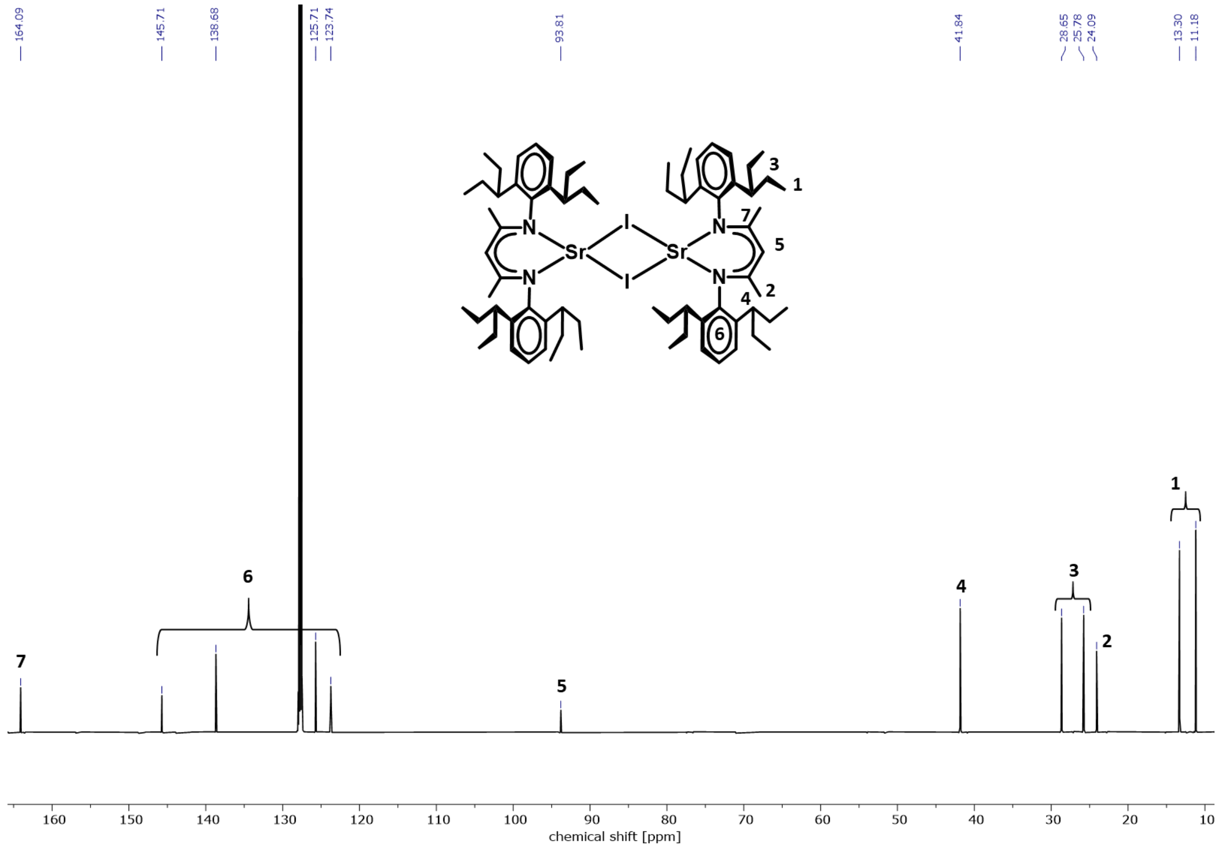

Figure S17. ^{13}C NMR (151 MHz, 298 K, C_6D_6) of [(^{DIPeP}BDI)Sr(μ-I)]$_2$.

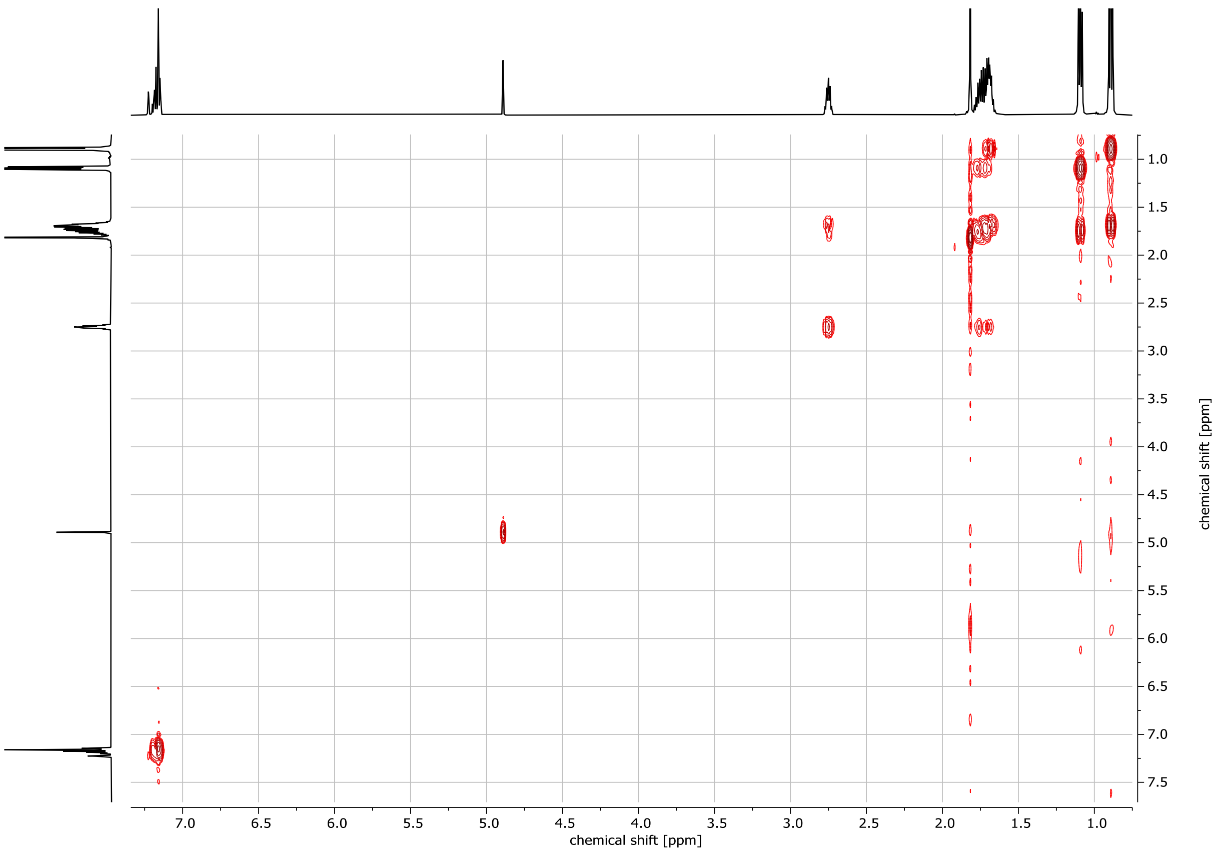

Figure S18. 2D-COSY NMR spectrum (600.13 MHz, 298 K, C_6D_6) of [(^{DIPeP}BDI)Sr(μ-I)]$_2$.

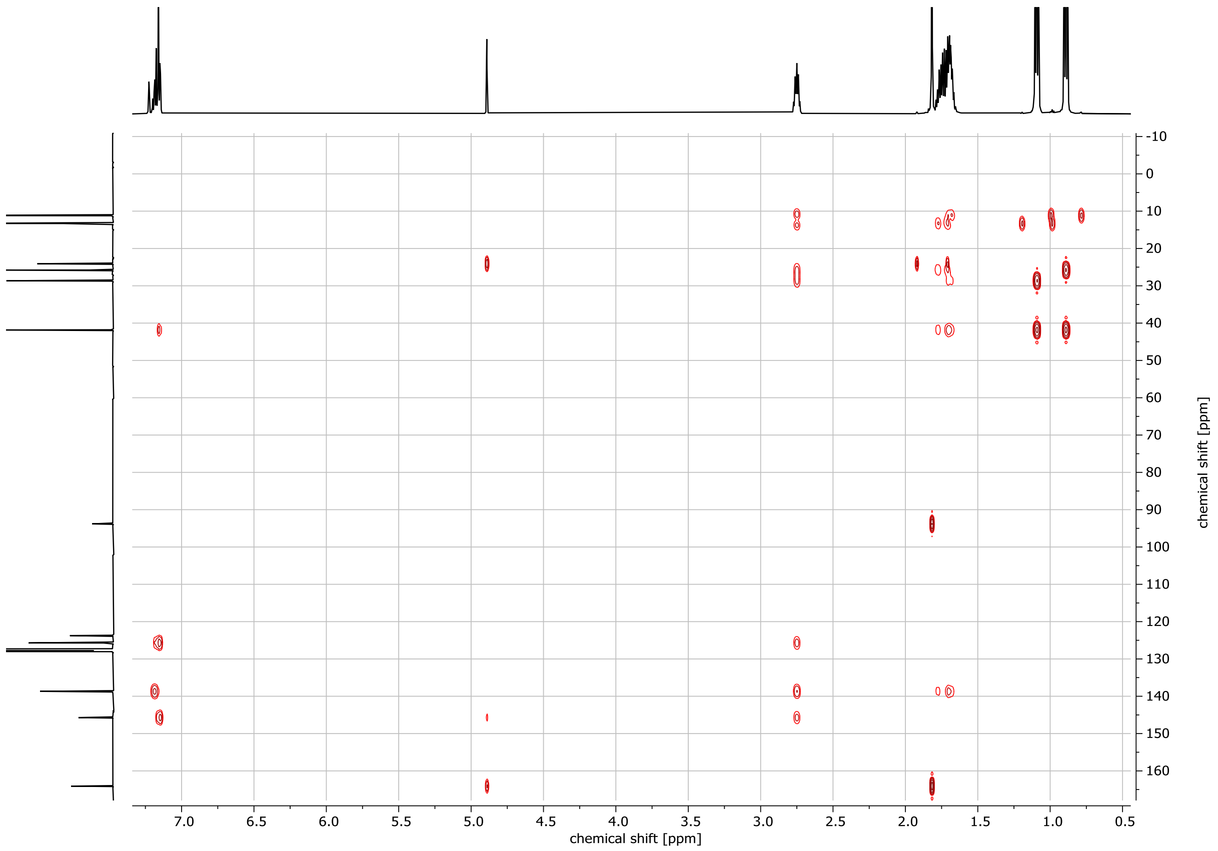

Figure S19. 2D-HMBC NMR spectrum of [(^{DIPeP}BDI)Sr(μ-I)]$_2$.

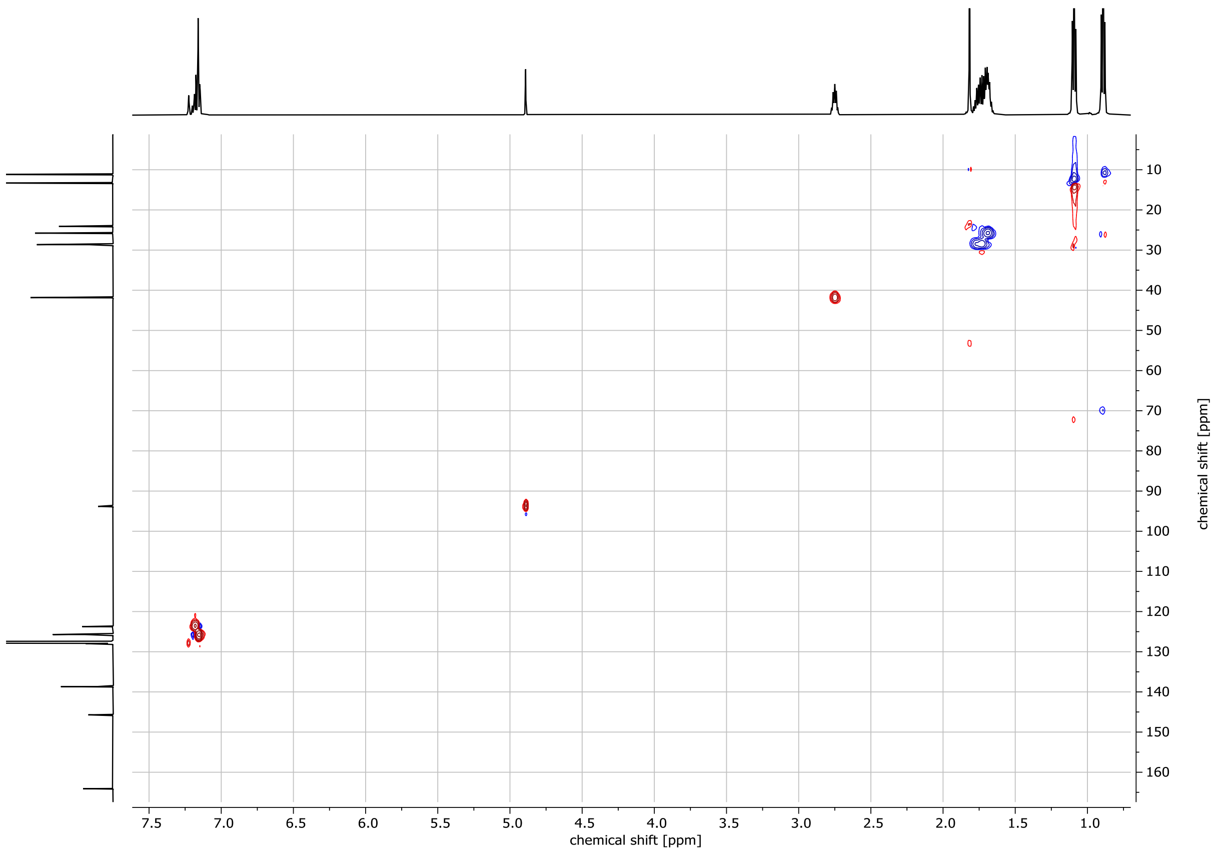

Figure S20. 2D-HSQC NMR spectrum of [(^{DIPeP}BDI)Sr(μ-I)]$_2$.

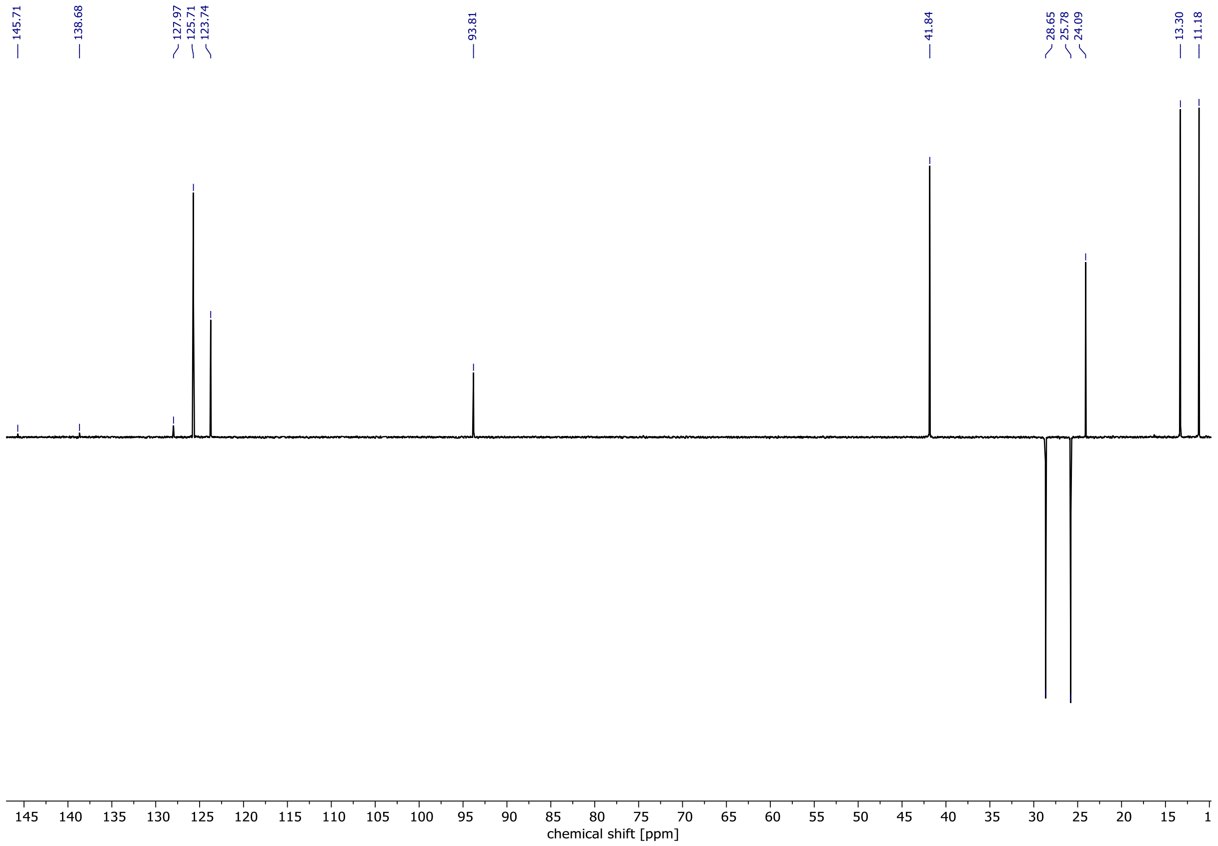

Figure S21. ^{13}C(DEPT 135) NMR (150.92 MHz, 298 K, C_6D_6) of [(^{DIPeP}BDI)Sr(μ-I)]$_2$.

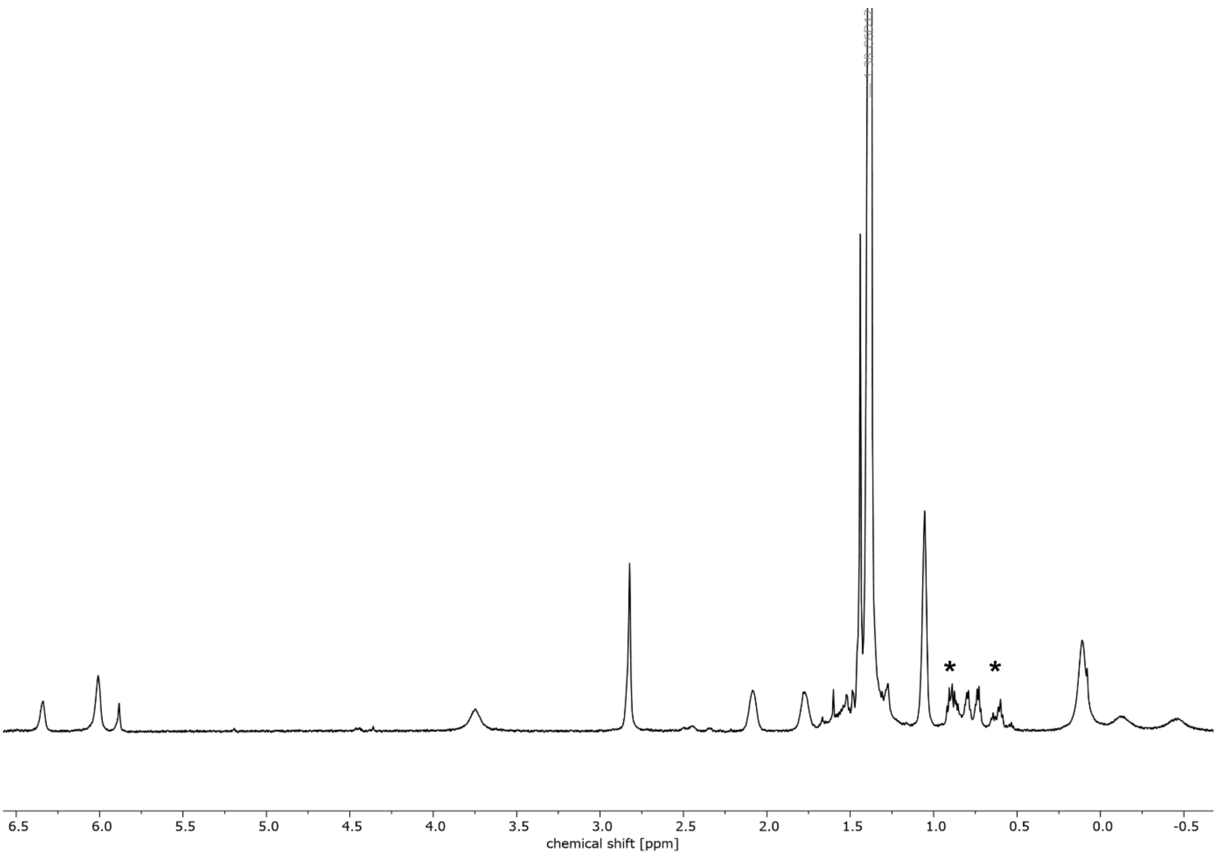

Figure S22. ^{1}H NMR (600.13 MHz, 298 K, cylcohexane-d_{12}) of $[(^{DIPeP}BDI)Sr]_2(C_6H_6)$, an assignment is not possible, due to paramagnetic character which causes signal broadening and an upfield shift. Residual solvent (pentane) is marked with asterisks.

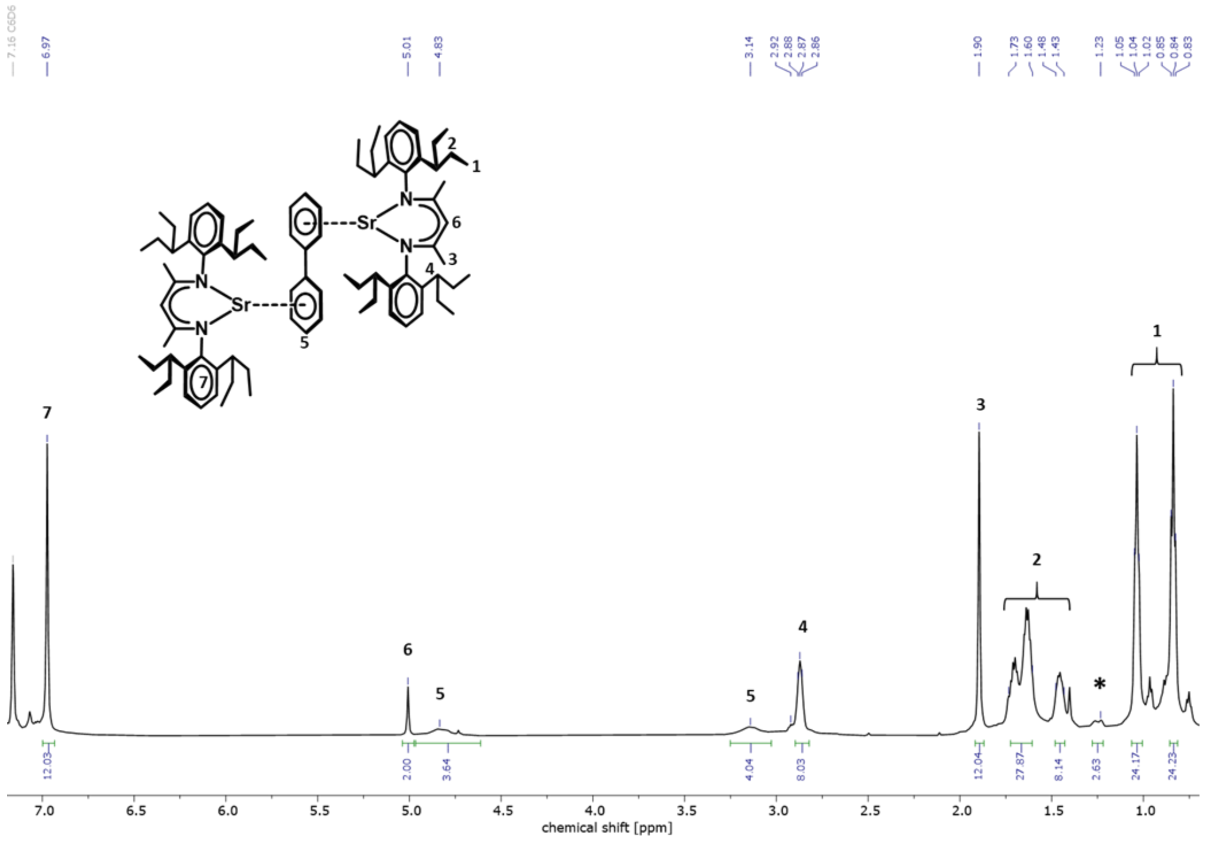

Figure S23. ^{1}H NMR (600.13 MHz, 298 K, C_6D_6) of [(^{DIPeP}BDI)Sr]$_2$(biphenyl). Co-crystallized pentane is marked with an asterisk.

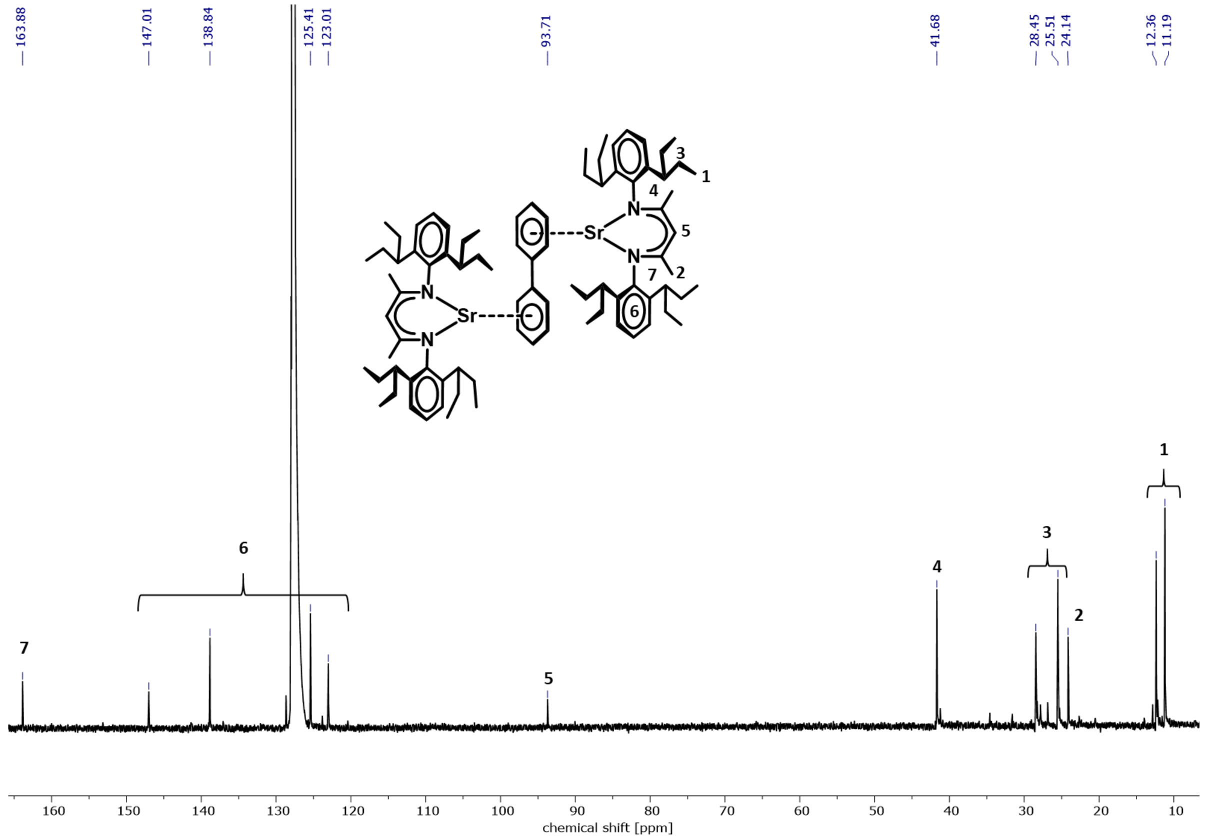

Figure S24. ^{13}C NMR (151 MHz, 298 K, C_6D_6) of [(^{DIPeP}BDI)Sr]$_2$(biphenyl). Biphenyl signals not visible.

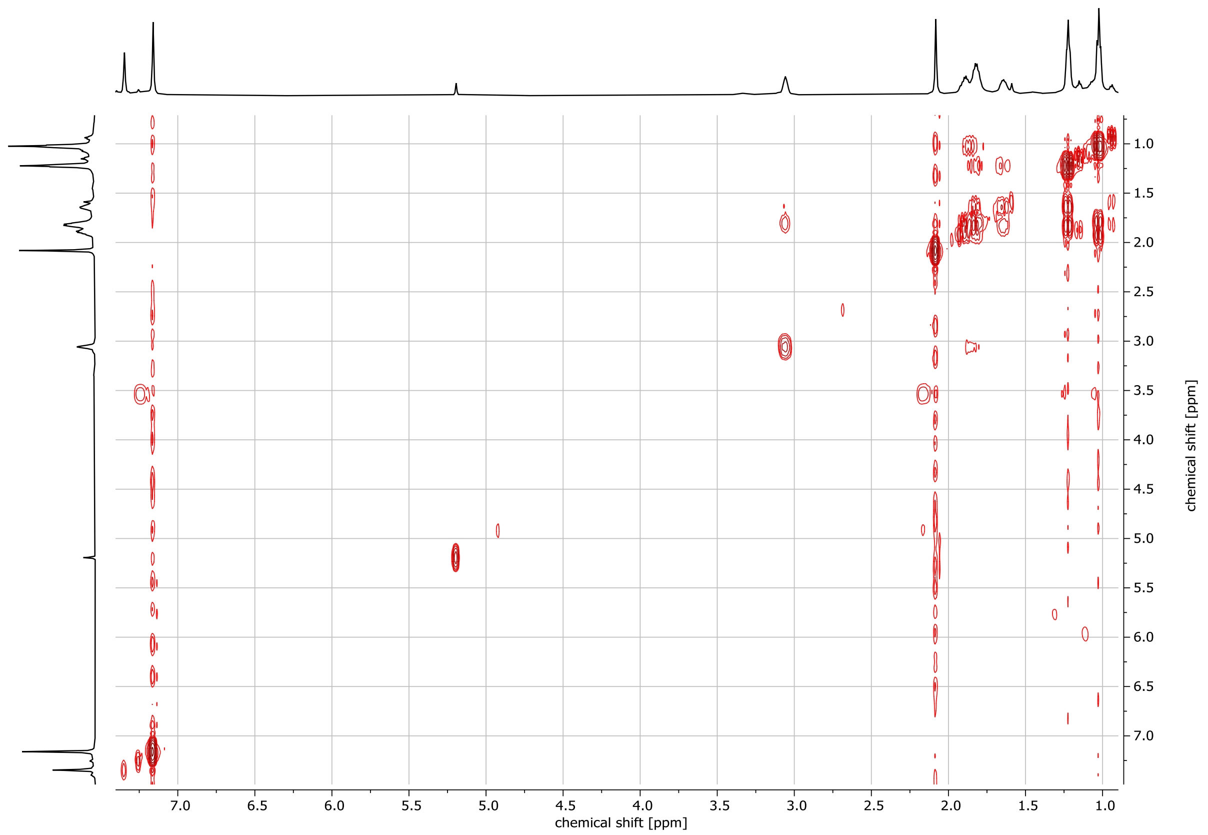

Figure S25. 2D-COSY NMR spectrum (600.13 MHz, 298 K, C_6D_6) of [(^{DIPeP}BDI)Sr]$_2$(biphenyl).

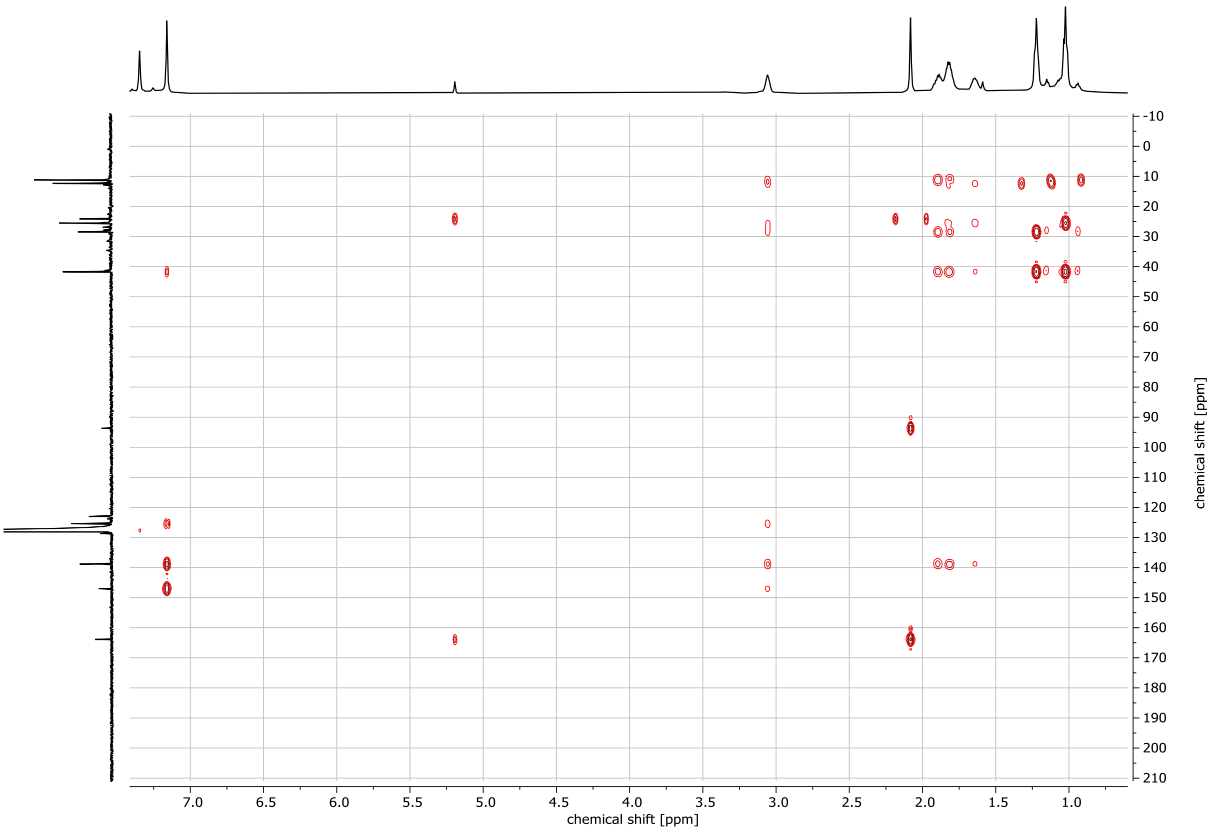

Figure S26. 2D-HMBC NMR spectrum of [(^{DIPeP}BDI)Sr]$_2$(biphenyl).

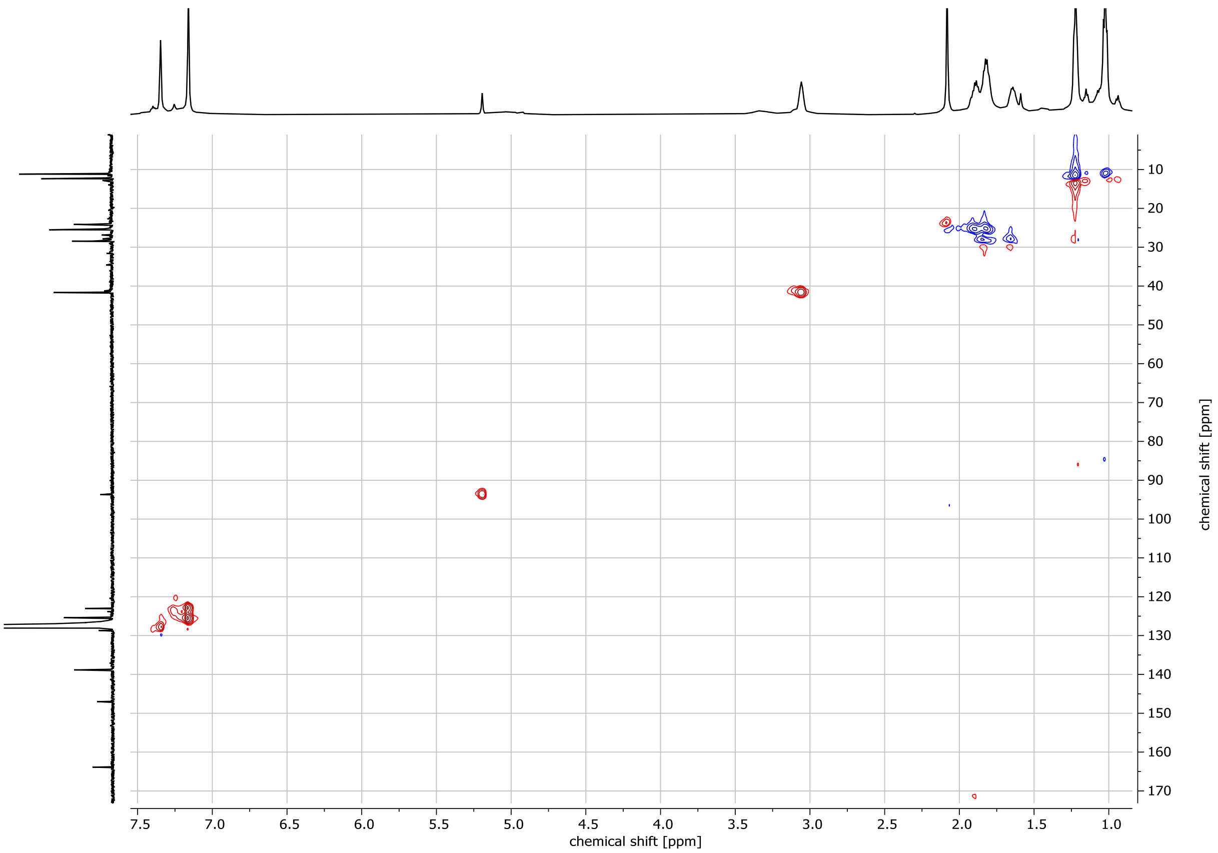

Figure S27. 2D-HSQC NMR spectrum of [(^{DIPeP}BDI)Sr]$_2$(biphenyl).

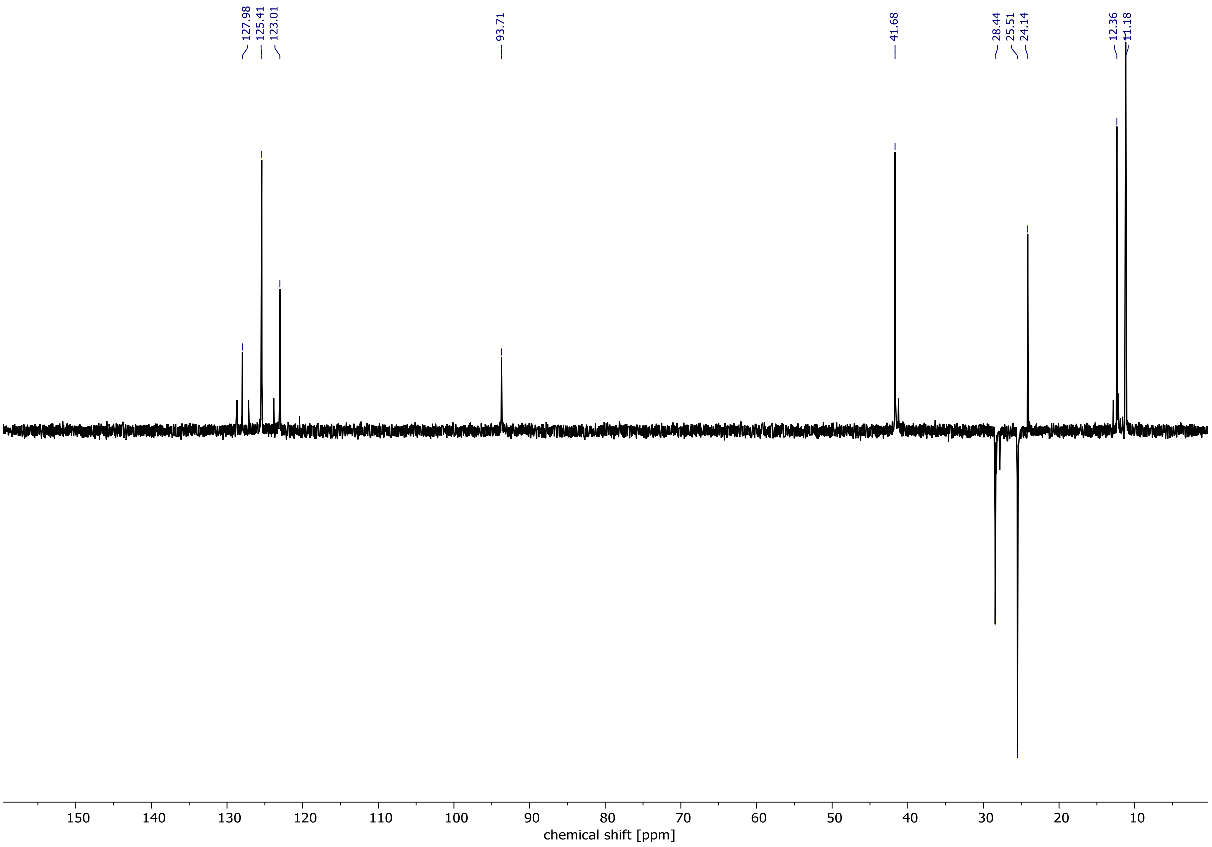

Figure S28. ^{13}C(DEPT 135) NMR (151 MHz, 298 K, C_6D_6) of [(^{DIPeP}BDI)Sr]$_2$(biphenyl).

3.2. NMR reactions

Ball-mill reduction of [(^{DIPP}BDI)Ca(μ-I)(THF)]$_2$ with K/KI and subsequent extraction with C_6D_6

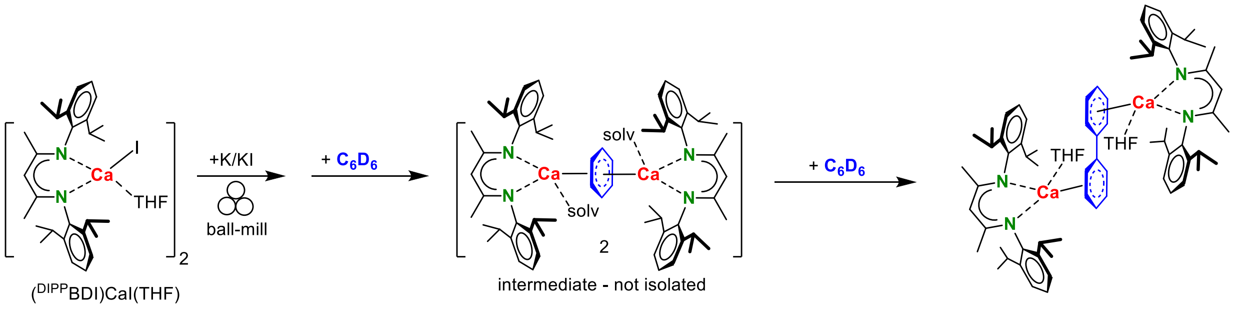

[(^{DIPP}BDI)Ca(μ-I)(THF)]$_2$ (131 mg, 0.100 mmol) was reduced with 5% K/KI (468 mg, 0.300 mmol) by ball-milling. The resulting powder (200 mg) was extracted with C_6D_6 (550 µL) and a ^{1}H NMR was measured three times over a period of 20 min; see Figure S29. At the start (2 min) the spectrum shows the paramagnetic intermediate, [(^{DIPP}BDI)Ca(THF)]$_2$(C_6H_6), which decomposes over time. After 20 min [(^{DIPP}BDI)Ca(THF)]$_2$(C_6H_6) is fully converted into [(^{DIPP}BDI)Ca(THF)]$_2$(biphenyl) (which completely crystallized) and various soluble side-products described in more detail in Figure S30.

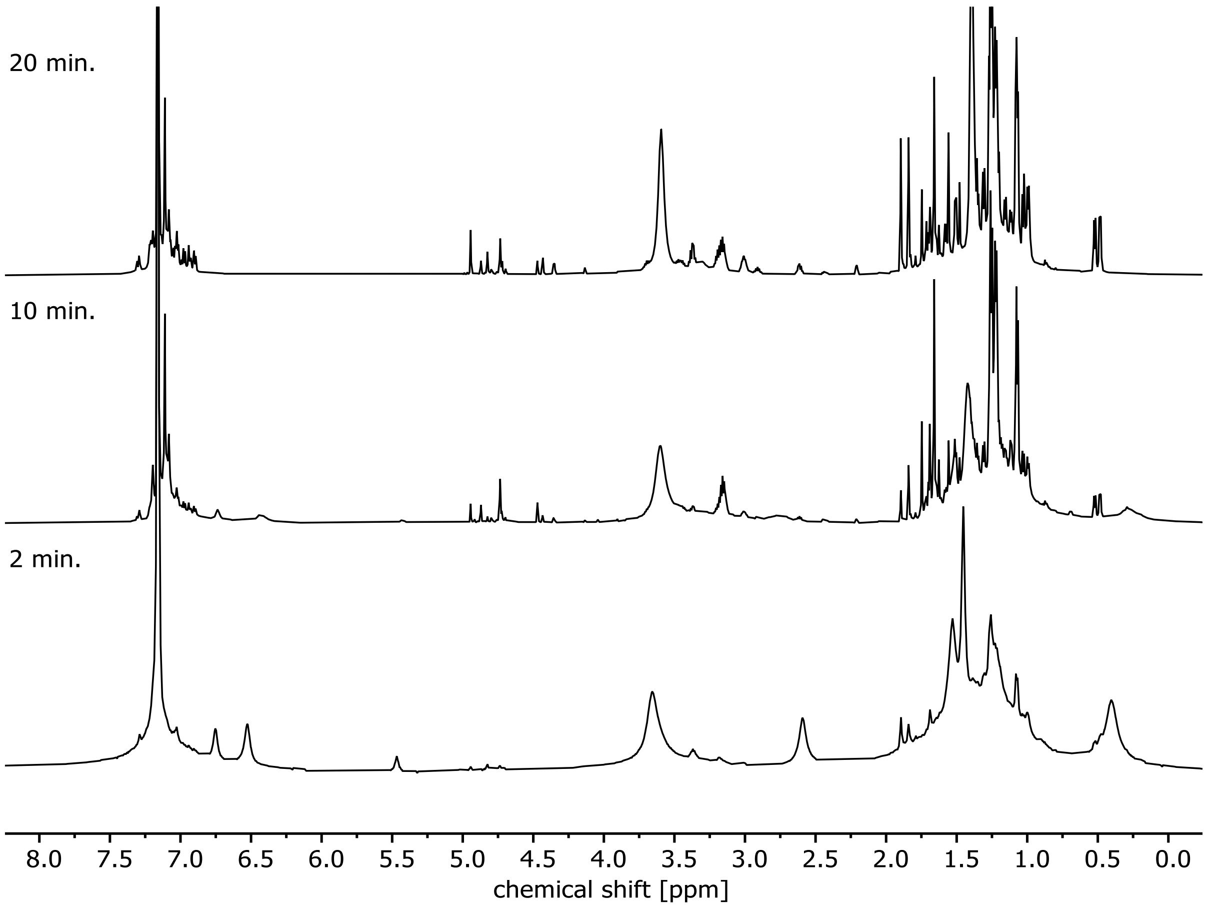

Figure S29. ^{1}H NMR (600.13 MHz, 298 K, C_6D_6) of sample dissolved in C_6D_6 after complete reduction of iodide precursor [(^{DIPP}BDI)Ca(μ-I)(THF)]$_2$ with K/KI by ball-milling and extraction with C_6D_6. Characteristic broadened signals indicate the main product [(^{DIPP}BDI)Ca(THF)]$_2$(C_6D_6) (t = 0) which is rapidly decomposing to [(^{DIPP}BDI)Ca(THF)]$_2$(biphenyl) and other products over 20 minutes. See Figure S30 for signal assignment.

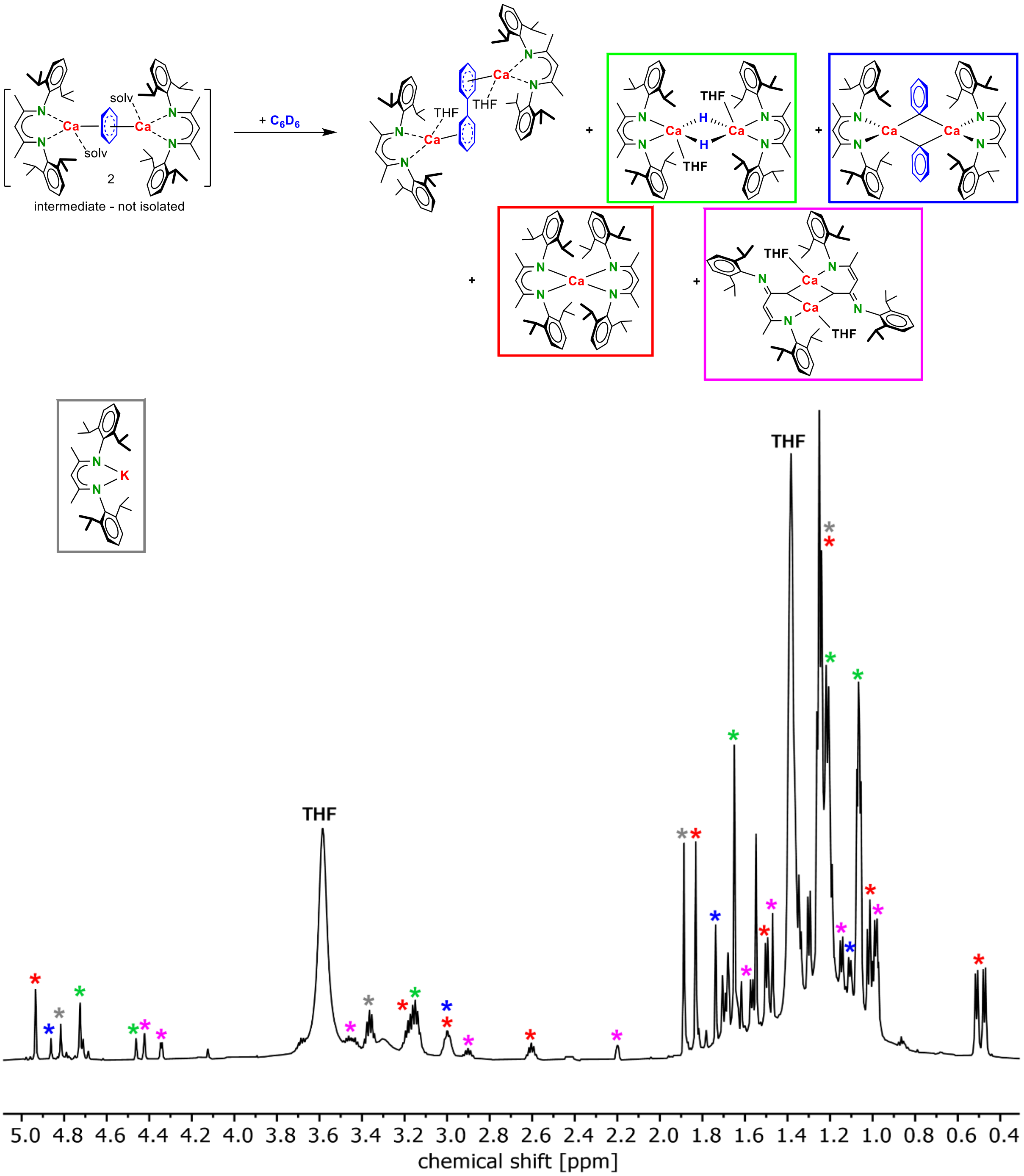

Figure S30. ^{1}H NMR (600.13 MHz, 298 K, C_6D_6) of a reaction mixture obtained after 20 min of $[(^{DIPP}BDI)Ca(THF)]_2(C_6D_6)$ in C_6D_6. Products identification based on chemical shifts of selected signals in different regions (the CH ligand backbone region 4-5 ppm, the C*H*Me₂ region 2.5-3.5 ppm, the ligand backbone Me region 1.5-2 ppm and the CH*Me₂* region 0.4-1.8 ppm): $[(^{DIPP}BDI)Ca(\mu\text{-H})(THF)]_2$, $[(^{DIPP}BDI)Ca(\mu\text{-D})(THF)]_2$, $[(^{DIPP}BDI)Ca(\mu\text{-}C_6D_5)]_2$,[S6] $[(^{DIPP}BDI^{-H})Ca(THF)]_2$, $[(^{DIPP}BDI)_2Ca]$ observed in approximate ratio: 2/1/1/2/3 respectively. The presence of signals corresponding to potassium salt is due to overreduction by K/KI.

C_6D_6/C_6H_6 exchange in complex $[(^{DIPeP}BDI)Ca]_2(C_6D_6)$

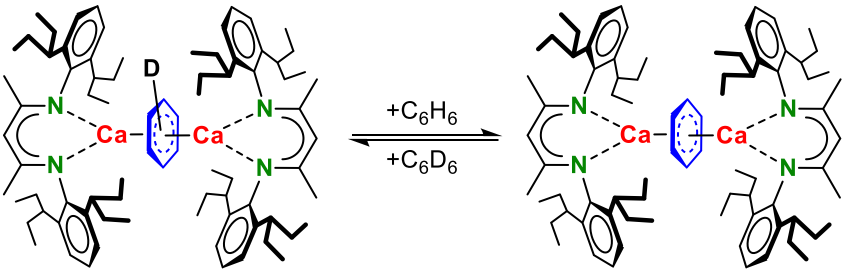

Complex $[(^{DIPeP}BDI)Ca]_2(C_6D_6)$ (12.5 mg, 0.010 mmol) was dissolved in hexane (550 μL) and C_6H_6 (10 μL) was added. The mixture was stirred at room temperature for 8 days. The formation of free C_6D_6 was monitored *via* ^{2}D NMR spectroscopy. In Figure S31 the ^{2}D spectra are depicted over time. The peaks at 1.7 and 1.4 ppm are impurities in the used solvents. The peak at 7.5 ppm can be assigned to free C_6D_6 which has been exchanged in the complex with C_6H_6. The dianion $C_6D_6^{2-}$ does not show any signals in the ^{2}D NMR spectra.

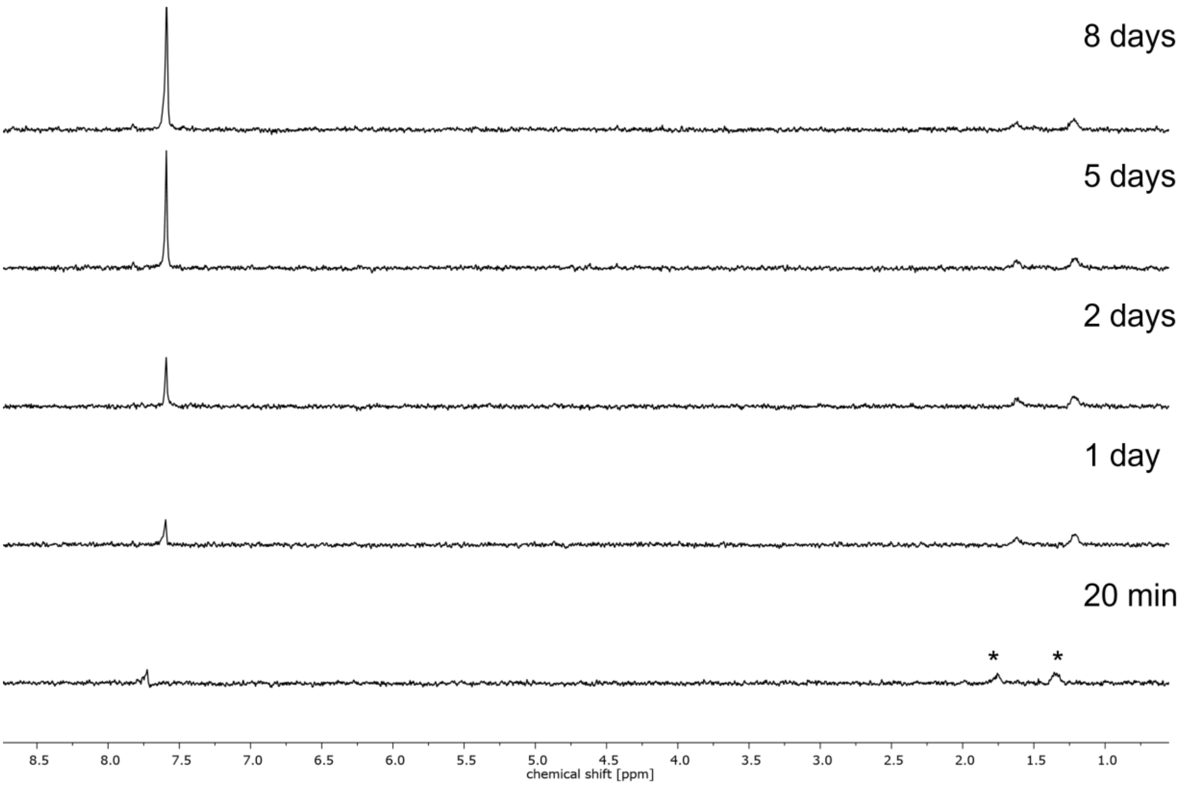

Figure S31. ^{2}D NMR spectrum (92.12 MHz, 289 K, hexane) for the reaction of $[(^{DIPeP}BDI)Ca]_2(C_6D_6)$ with C_6H_6. Impurities in the used solvents are marked with asterisks. Over a time of 8 days, the signal of free C_6D_6 gets more intense, which indicates exchange of the bridging $C_6D_6^{2-}$ dianion for $C_6H_6^{2-}$ and the generation of free C_6D_6.

Reduction of [(^{DIPeP}BDI)Ca(μ-I)]$_2$in presence of biphenyl

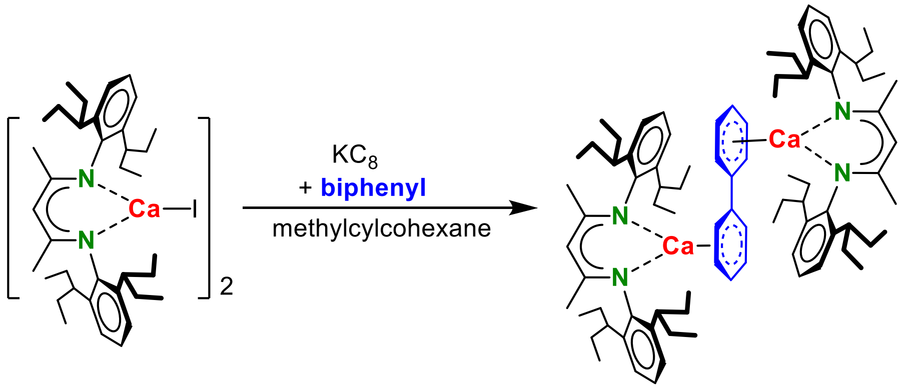

[(^{DIPeP}BDI)Ca(μ-I)]$_2$ (300 mg, 0.431 mmol), KC_8 (348 mg, 2.58 mmol) and biphenyl (33.1 mg, 0.215 mmol) was suspended in methylcyclohexane (8 mL) and stirred for 7 days. The dark brown reaction mixture was filtered, and the solvent was removed, resulting in a dark brown solid. Figure S32 shows an NMR of this solid, which is the raw [(^{DIPeP}BDI)Ca]$_2$(biphenyl) complex (backbone C-H at 5.00 ppm). The NMR show a clean reaction with minor amounts of potassium salt (backbone C-H signal at 4.72 ppm) due to overreduction.

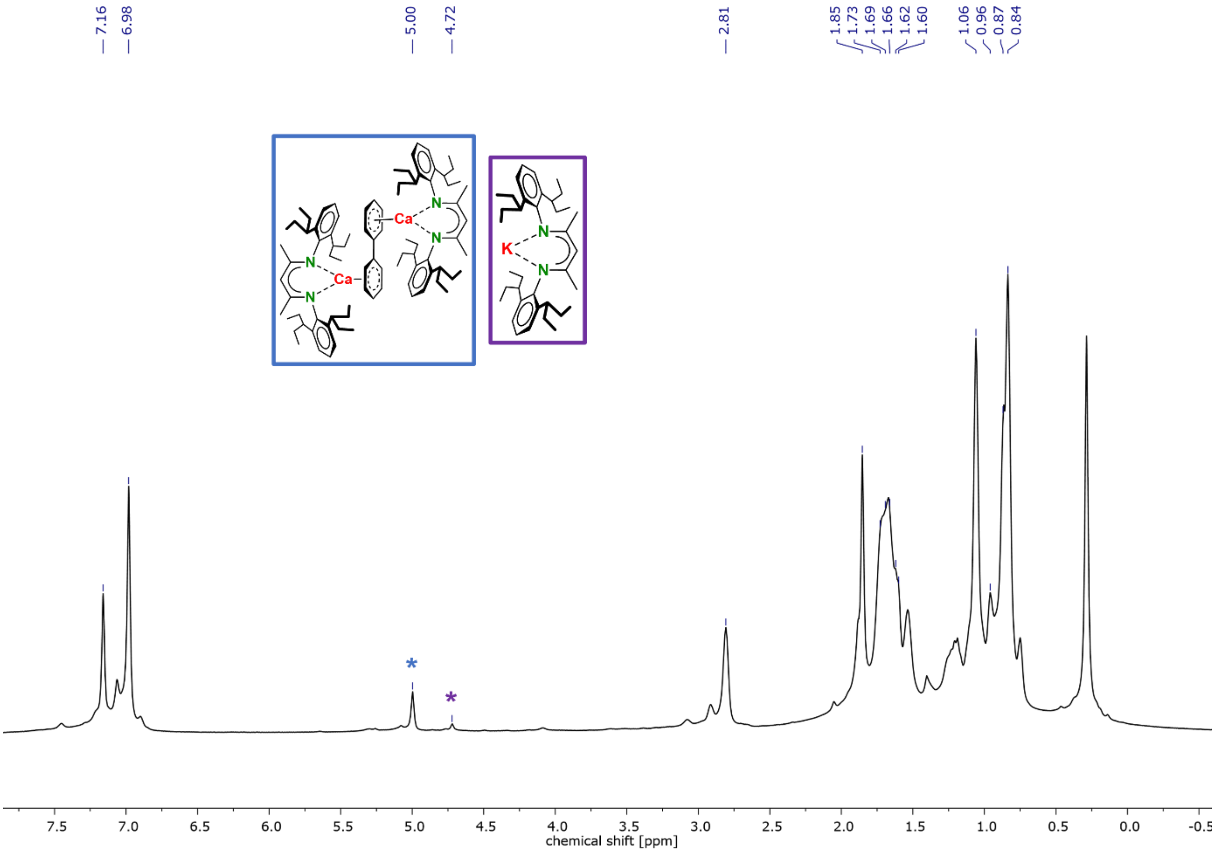

Figure S32. ^{1}H NMR (600.13 MHz, 289 K, C_6D_6) spectrum of crude [(^{DIPeP}BDI)Ca]$_2$(biphenyl), before treating with THF. The characteristic backbone signal at 5.00 ppm is shifted compared to the complex with coordinating THF (4.82 ppm) (see Figure S10).

Reaction of [(^{DIPeP}BDI)Ca]$_2$(C_6H_6) with biphenyl

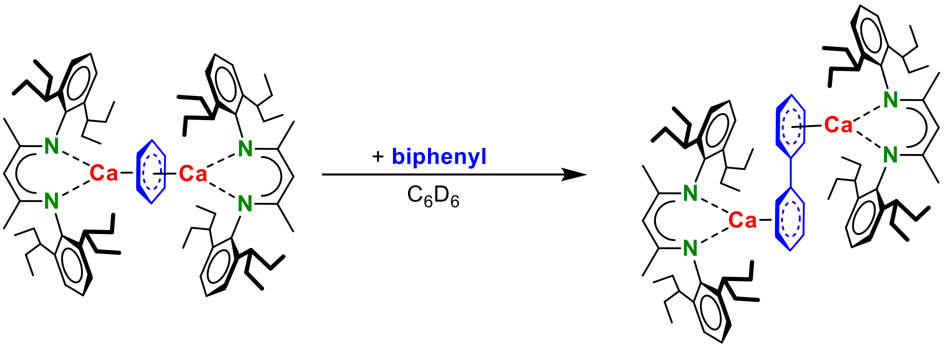

[(^{DIPeP}BDI)Ca(C_6D_6)]$_2$ (19.5 mg, 0.016 mmol) and biphenyl (2.47 mg, 0.016 mmol) were mixed in C_6D_6 (550 µL) and stirred at 20 °C. The reaction was monitored *via* ^{1}H NMR spectroscopy. The reaction was finished after 7 days. Figure S33 shows a ^{1}H NMR spectrum of the mixture after complete reaction. The main product is [(^{DIPeP}BDI)Ca]$_2$(biphenyl) (C-H backbone at 5.00 ppm). Besides the formation of the main product [(^{DIPeP}BDI)Ca]$_2$(biphenyl) complexes (88% conversion), also traces of [(^{DIPeP}BDI)$_2$Ca] (5%) and [(^{DIPeP}BDI)Ca(μ-H)]$_2$ (7%) are formed.

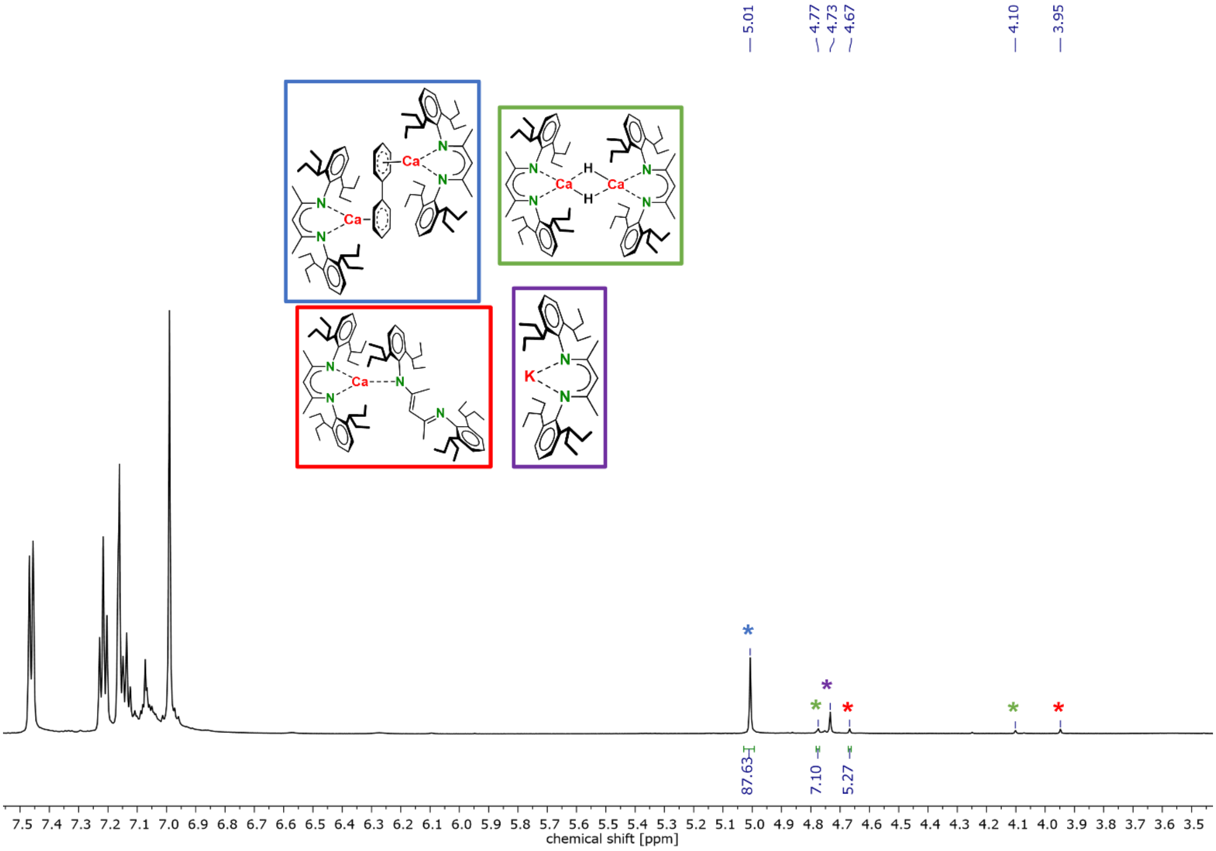

Figure S33. ^{1}H NMR (600.13 MHz, 289 K, C_6D_6) spectrum of the reaction of [(^{DIPeP}BDI)Ca]$_2$(C_6H_6) with biphenyl with a conversion of 88% to the [(^{DIPeP}BDI)Ca]$_2$(biphenyl) complex. Only traces of other side-products like [(^{DIPeP}BDI)$_2$Ca] (4.67/3.95 ppm) and [(^{DIPeP}BDI)Ca(μ-H)]$_2$ (4.77/4.10 ppm) are formed. The peak at 4.73 ppm can be assigned to the potassium complex, which is an impurity in the starting material [(^{DIPeP}BDI)Ca]$_2$(C_6H_6), due to overreduction.

Reaction of [(^{DIPeP}BDI)Ca]$_2$(C_6H_6) with C_6D_6 at 20 °C

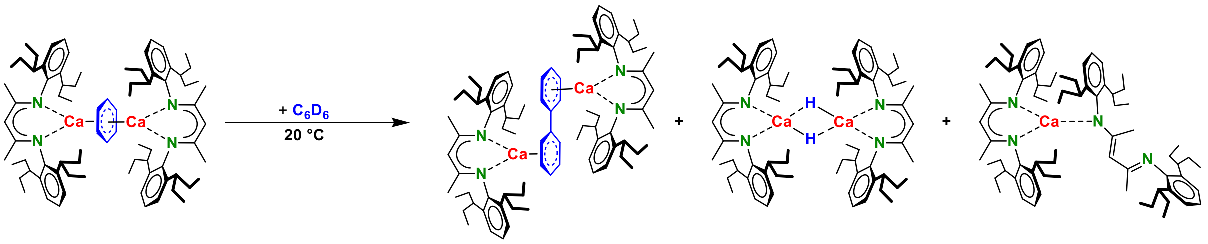

[(^{DIPeP}BDI)Ca]$_2$(C_6H_6) (15.1 mg, 0.012 mmol) was stirred in C_6D_6 (550 µL) at 20 °C overnight. Figure S34 shows that the [(^{DIPeP}BDI)Ca]$_2$(biphenyl) complex is formed, as well as two other side-products. Note that [(^{DIPeP}BDI)Ca]$_2$(C_6H_6) is only partially converted and still the major species in the mixture.

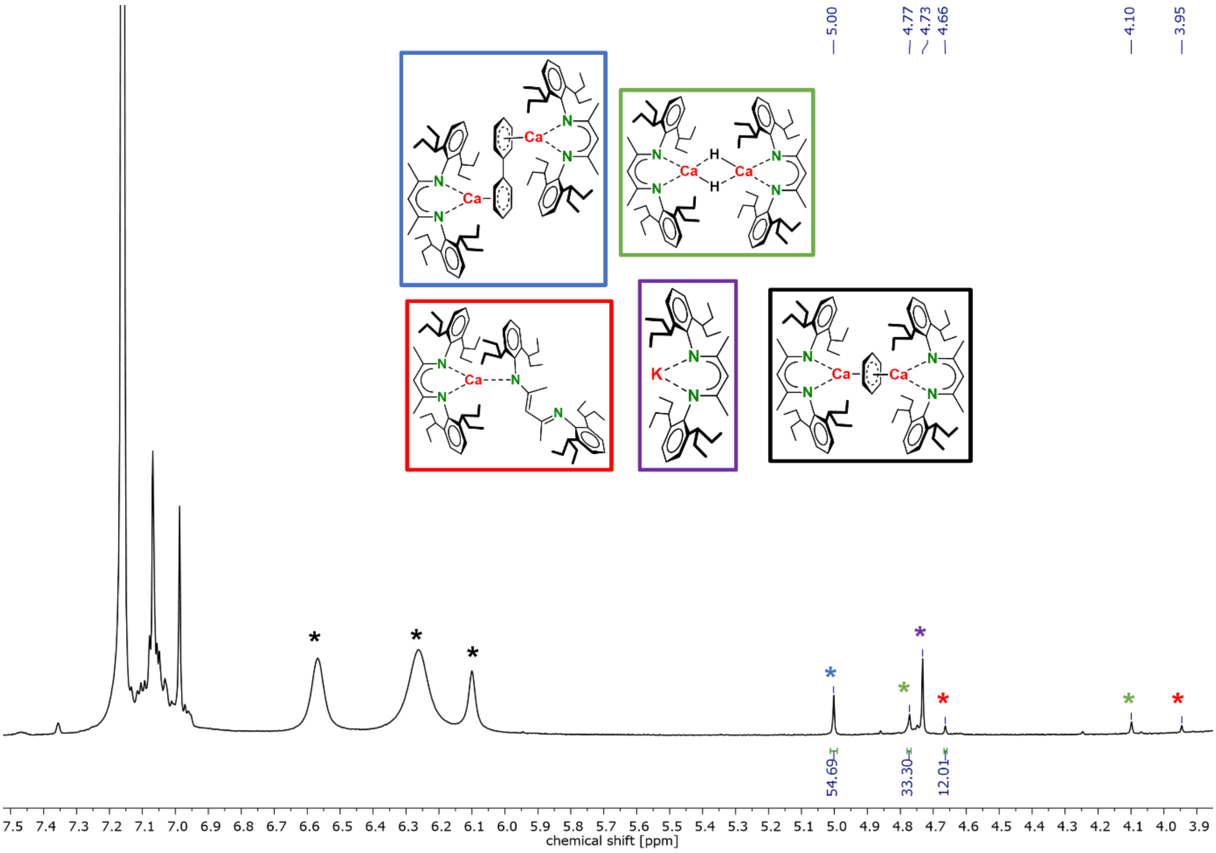

Figure S34. ^{1}H NMR (600.13 MHz, 289 K, C_6D_6) reaction of [(^{DIPeP}BDI)Ca]$_2$(C_6H_6) with C_6D_6 at 20 °C after 1 day. The [(^{DIPeP}BDI)Ca]$_2$(C_6H_6) complex is still present, but already reaction/decomposition products are observable (product ratio: [(^{DIPeP}BDI)Ca]$_2$(biphenyl) 55%, [(^{DIPeP}BDI)Ca(μ-H)]$_2$ 33% and (^{DIPeP}BDI)$_2$Ca 12%). (^{DIPeP}BDI)K is an impurity of the starting material [(^{DIPeP}BDI)Ca]$_2$(C_6H_6), due to overreduction.

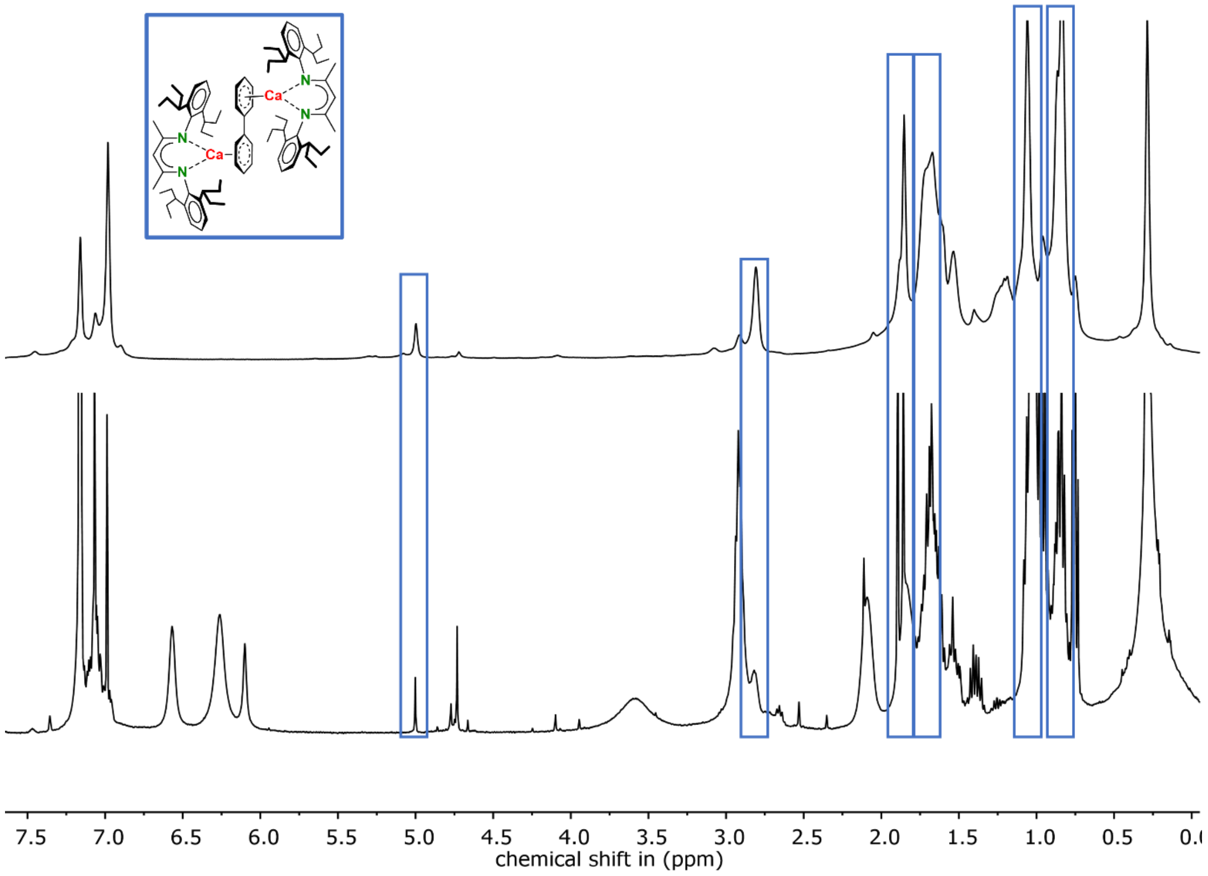

Figure 35. ^{1}H NMR comparison of [(^{DIPeP}BDI)Ca]$_2$(biphenyl) complex without THF (top) and the reaction mixture of [(^{DIPeP}BDI)Ca]$_2$(C_6H_6) with C_6D_6 at 20 °C after 1 day. For clarity the blue boxes show the same chemical shift. It is clearly observable, that the [(^{DIPeP}BDI)Ca]$_2$(biphenyl) complex is formed in the reaction with C_6D_6.

Reaction of [(^{DIPeP}BDI)Ca]$_2$(C_6H_6) with C_6D_6 at 30 °C

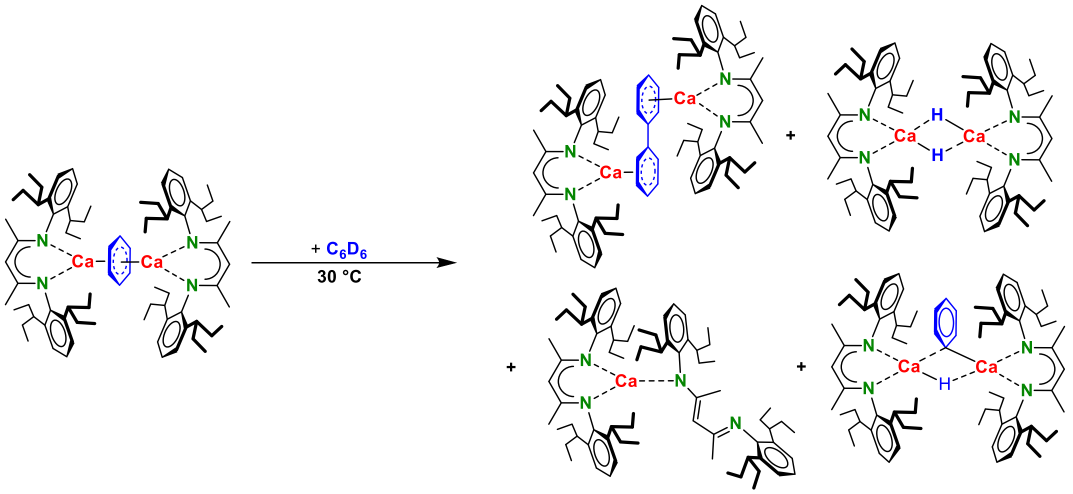

[(^{DIPeP}BDI)Ca]$_2$(C_6H_6) (12.3 mg, 0.010 mmol) was stirred in C_6D_6 (550 µL) at 30 °C for 14 days. Figure S35 shows that the [(^{DIPeP}BDI)Ca]$_2$(biphenyl) complex is formed in traces, as well as four other side-products. Note that [(^{DIPeP}BDI)Ca]$_2$(C_6H_6) is nearly fully converted.

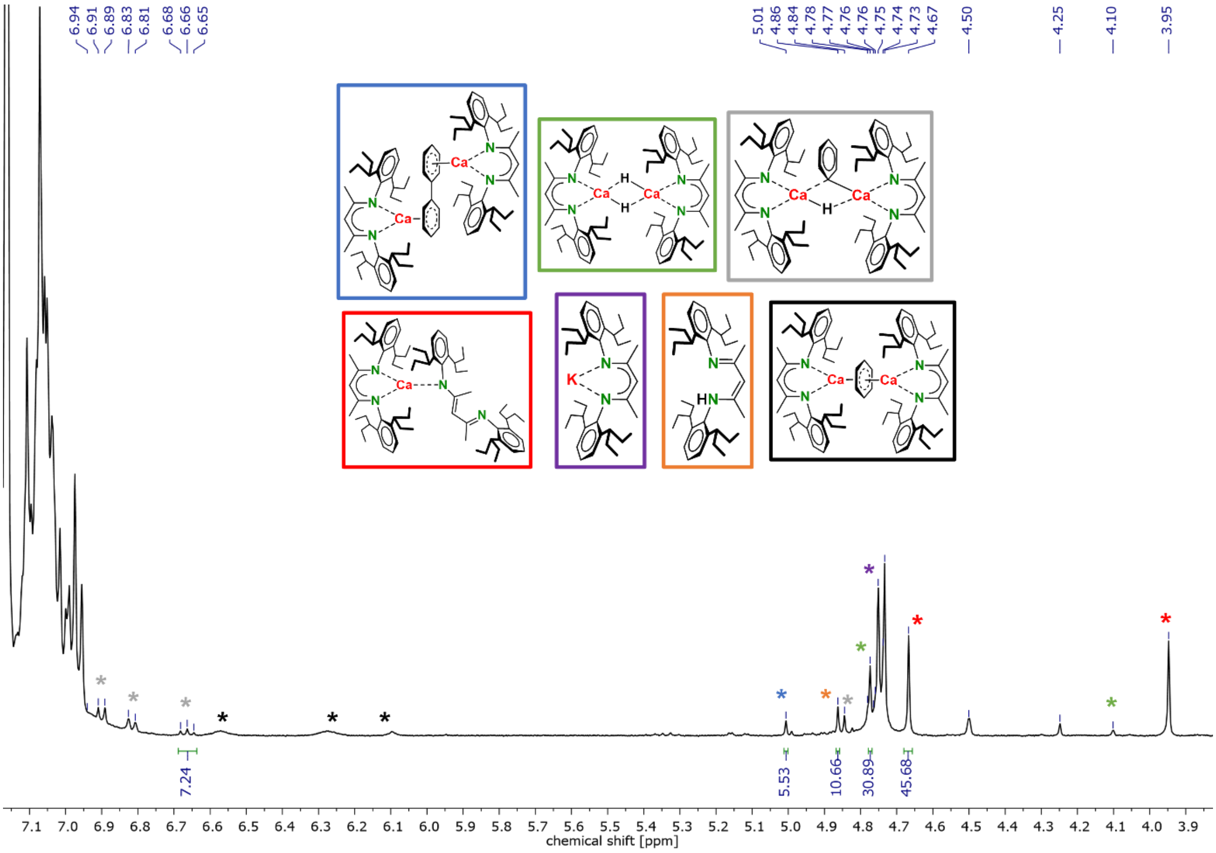

Figure S36. ^{1}H NMR (600.13 MHz, 289 K, C_6D_6) of reaction of [(^{DIPeP}BDI)Ca]$_2$(C_6H_6) with C_6D_6 at 30 °C after 14 days. The [(^{DIPeP}BDI)Ca]$_2$(C_6H_6) complex is still present, but reaction/decomposition products are observable. (product ratio: [(^{DIPeP}BDI)Ca]$_2$(biphenyl) 6%, [(^{DIPeP}BDI)Ca(μ-H)]$_2$ 31%, (^{DIPeP}BDI)$_2$Ca 46%, (^{DIPeP}BDI)H 11% and [(^{DIPeP}BDI)Ca]$_2$(μ-H)(μ-Ph) 7%). (^{DIPeP}BDI)K is an impurity of the starting material [(^{DIPeP}BDI)Ca]$_2$(C_6H_6), due to overreduction.

Reaction of [(^{DIPeP}BDI)Ca]$_2$(C_6H_6) with C_6D_6 at 50 °C

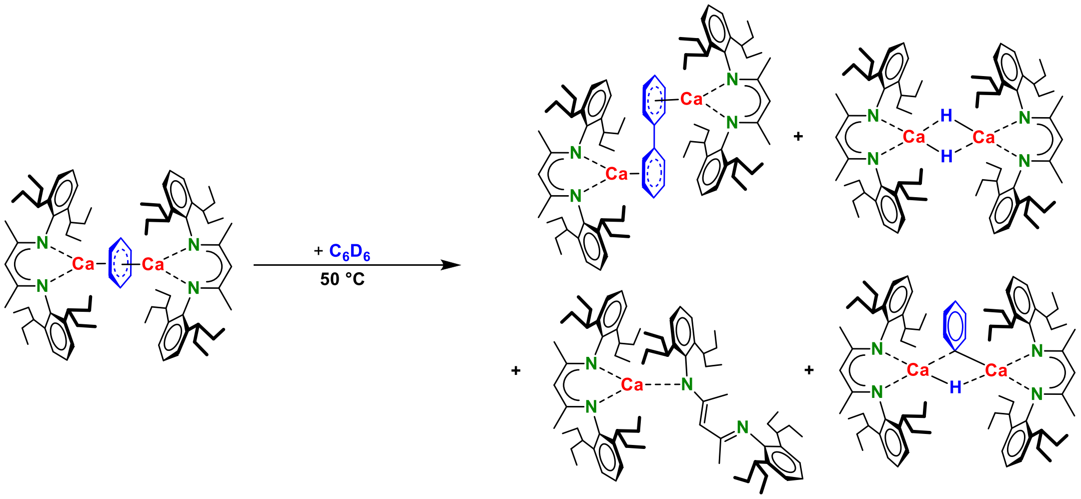

[(^{DIPeP}BDI)Ca]$_2$(C_6H_6) (11.4 mg, 0.009 mmol) was stirred in C_6D_6 (550 µL) at 50 °C for 3 days. Figure S36 shows that the [(^{DIPeP}BDI)Ca]$_2$(biphenyl) complex is formed in trace amounts, as well as other side-products. The starting material [(^{DIPeP}BDI)Ca]$_2$(C_6H_6) is fully decomposed.

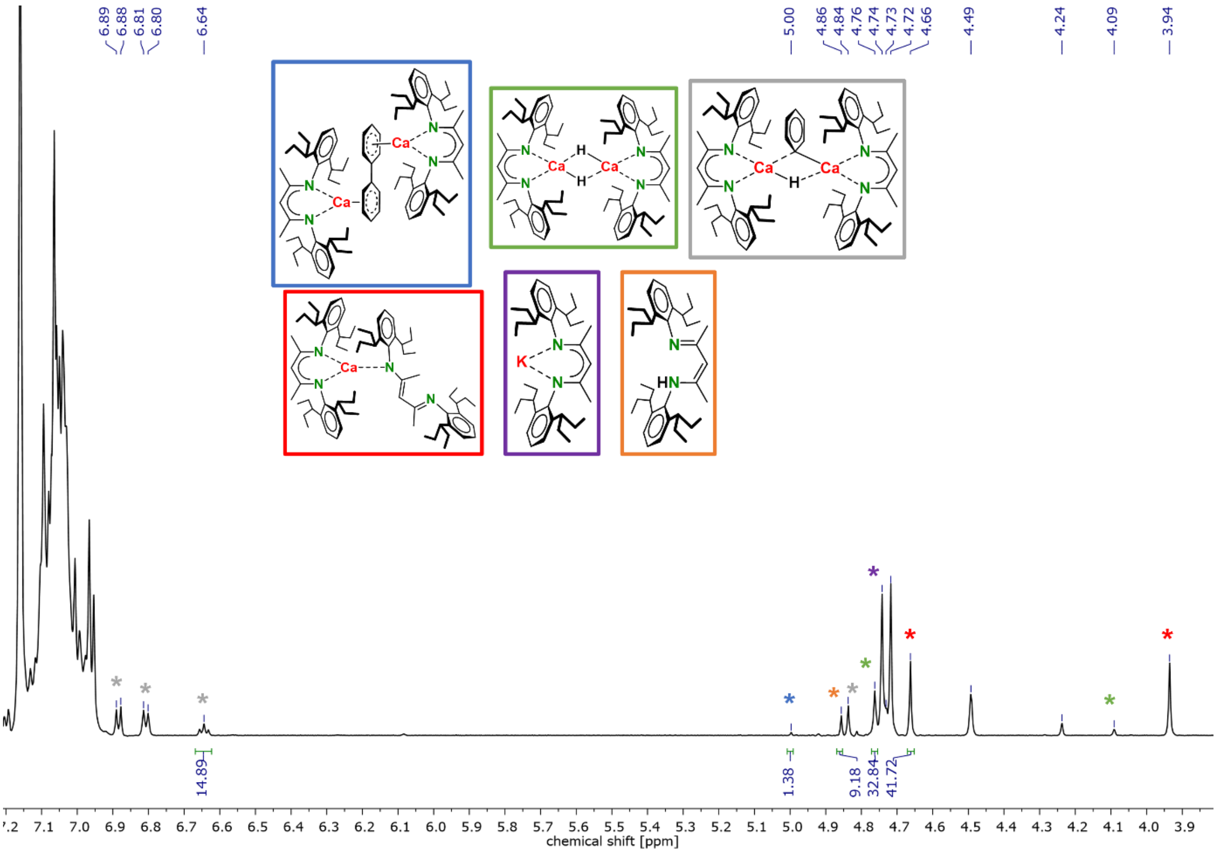

Figure S37. ^{1}H NMR (600.13 MHz, 289 K, C_6D_6) of reaction of [(^{DIPeP}BDI)Ca]$_2$(C_6H_6) with C_6D_6 at 50 °C after 3 days. Reaction/decomposition products are observable. (product ratio: [(^{DIPeP}BDI)Ca]$_2$(biphenyl) 1%, [(^{DIPeP}BDI)Ca(μ-H)]$_2$ 33%, (^{DIPeP}BDI)$_2$Ca 42%, (^{DIPeP}BDI)H 9% and [(^{DIPeP}BDI)Ca]$_2$(μ-H)(μ-Ph) 15%). (^{DIPeP}BDI)K is an impurity of the starting material [(^{DIPeP}BDI)Ca]$_2$(C_6H_6), due to overreduction.

Reaction of [(^{DIPeP}BDI)Sr]$_2$(C_6H_6) with C_6D_6 at 20 °C

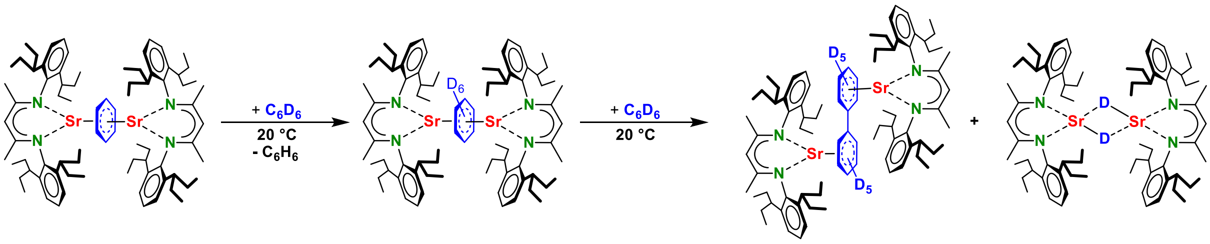

[(^{DIPeP}BDI)Sr]$_2$(C_6H_6) (15 mg, 0.011 mmol) was dissolved in C_6D_6 (2.75 mL) and stirred at 20 °C for 10 minutes. Figure S38 shows that [(^{DIPeP}BDI)Sr]$_2$(C_6H_6) reacted in a highly selective fashion to the [(^{DIPeP}BDI)Sr]$_2$(biphenyl) complex. Due to prior exchange of C_6H_6 for C_6D_6, the resonance signals attributed to the biphenyl (C_6D_5-C_6D_5) moiety of the complex cannot be observed in the ^{1}H NMR spectrum. Thus, the reaction mixture was dried *in vacuo* and the black residue was dissolved in methylcyclohexane to afford a dark brown solution. The ^{2}D NMR spectrum in Figure S39 shows signals which can be assigned to the deuterated biphenyl moiety of [(^{DIPeP}BDI)Sr]$_2$(biphenyl). Signals at 1.78 and 1.39 ppm are due to impurities in the used solvents.

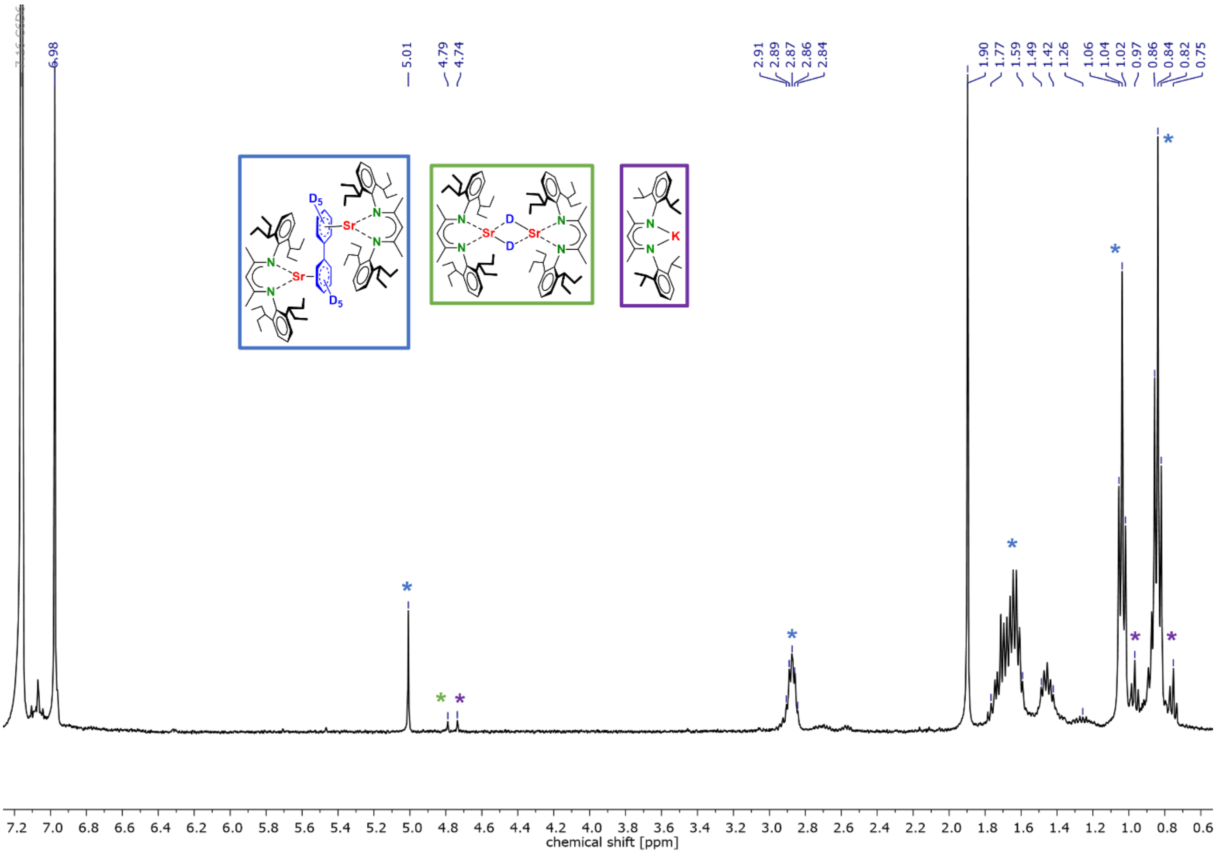

Figure S38. ^{1}H NMR (600.13 MHz, 289 K, C_6D_6) reaction of [(^{DIPeP}BDI)Sr]$_2$(C_6H_6) with C_6D_6 at 20 °C. The spectrum was measured 10 minutes after [(^{DIPeP}BDI)Sr]$_2$(C_6H_6) was dissolved in C_6D_6. The potassium salt (^{DIPeP}BDI)K is a side-product formed during the synthesis of [(^{DIPeP}BDI)Sr]$_2$(C_6H_6) *via* reduction of [(^{DIPeP}BDI)Sr(μ-I)]$_2$ with KC_8. Since the bridging C_6H_6 ring exchanges fast with C_6D_6 prior to biphenyl formation, mainly biphenyl-d_{10} is formed. This is visible in the ^{2}D NMR spectrum Figure S39.

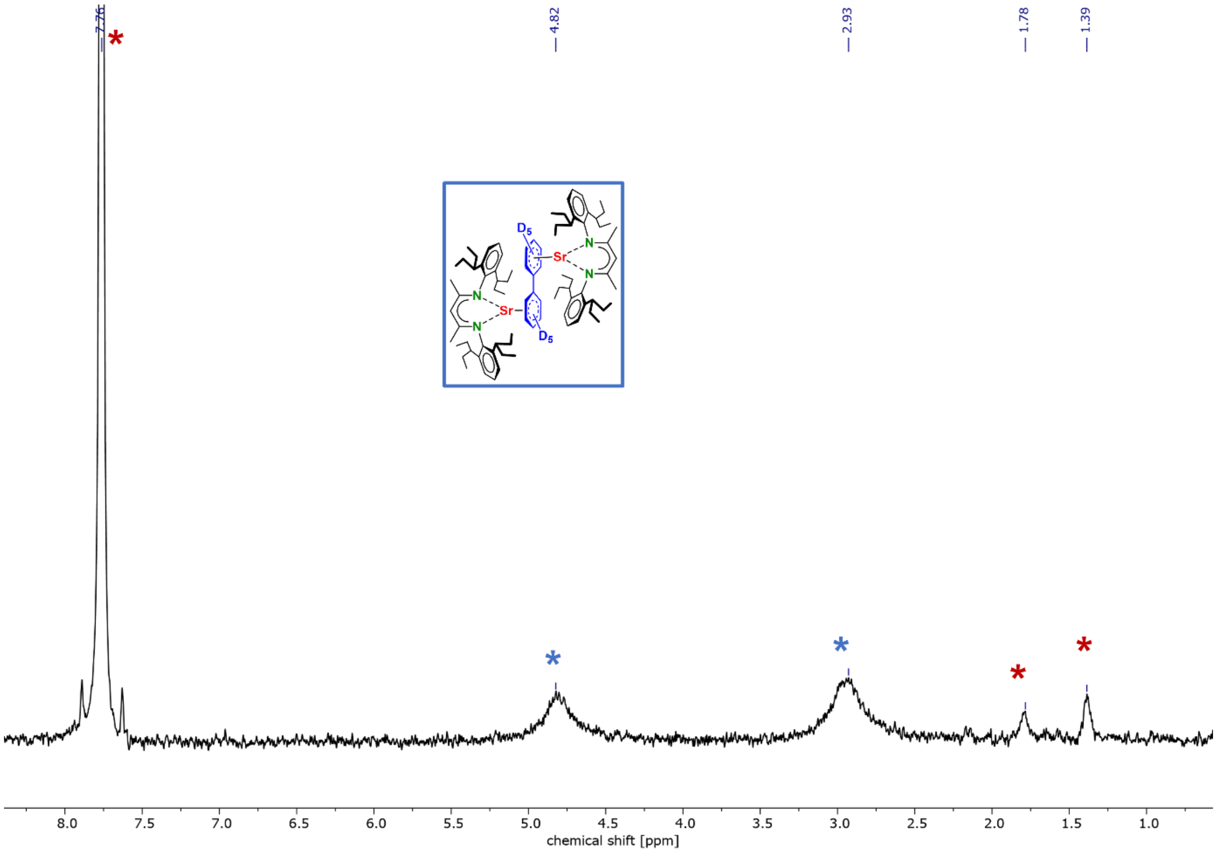

Figure S39. ^{2}D NMR (600.13 MHz, 289 K, methylcyclohexane) spectrum of $[(^{DIPeP}BDI)Sr]_2$(biphenyl) obtained *via* the reaction of $[(^{DIPeP}BDI)Sr]_2(C_6H_6)$ with C_6D_6. Signals of the deuterated biphenyl anion are marked with blue asterisks. Residual C_6D_6 and impurities are marked with red asterisks.

Reaction of [(^{DIPeP}BDI)Sr]$_2$(C_6H_6) with C_6H_6 at 20 °C

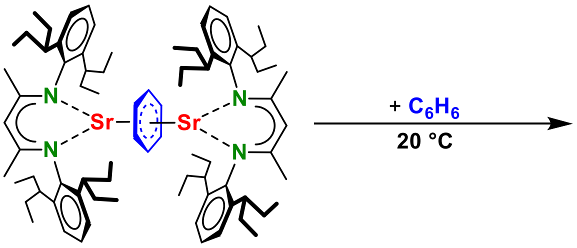

[(^{DIPeP}BDI)Sr]$_2$(C_6H_6) (15 mg, 0.011 mmol) was dissolved in C_6H_6 (2.75 mL) and stirred at 20 °C. Figures S40 show that the [(^{DIPeP}BDI)Sr]$_2$(C_6H_6) react slowly over 3 days with C_6H_6 to [(^{DIPeP}BDI)Sr]$_2$(biphenyl). Compared to the reaction in C_6D_6 the reaction is not selective, as also [(^{DIPeP}BDI)Sr(μ-H)]$_2$ is formed, as the main product.

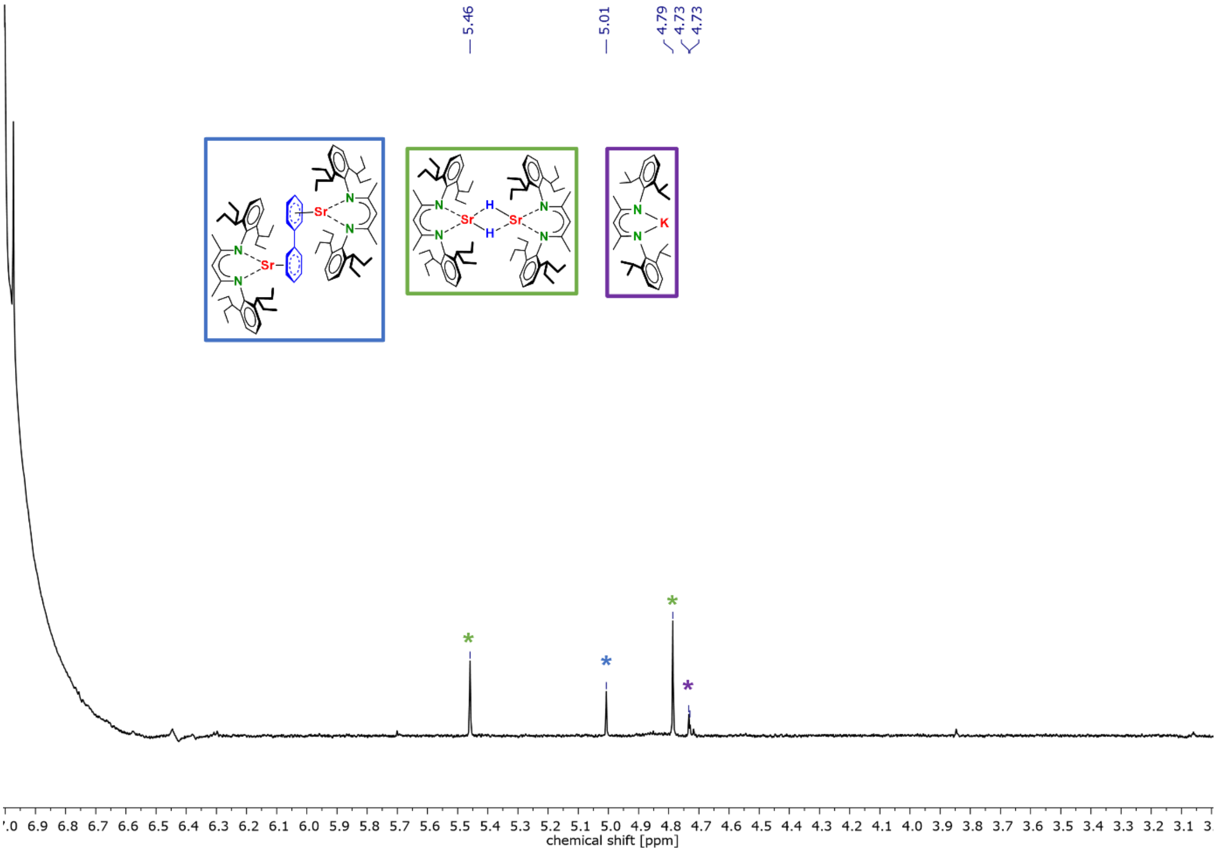

Figure S40. ^{1}H NMR (600.13 MHz, 298 K, C_6H_6) reaction spectrum of [(^{DIPeP}BDI)Sr]$_2$(C_6H_6) with C_6H_6. After 3 days the starting material is fully decomposed to mainly the [(^{DIPeP}BDI)Sr(μ-H)]$_2$ but also [(^{DIPeP}BDI)Sr]$_2$(biphenyl) is formed, in a ratio 3:1. (^{DIPeP}BDI)K is an impurity from the starting material [(^{DIPeP}BDI)Sr]$_2$(C_6D_6), due to overreduction

4. GC-MS Data

Reaction of $[(^{DIPeP}BDI)Sr]_2(C_6H_6)$ with C_6D_6

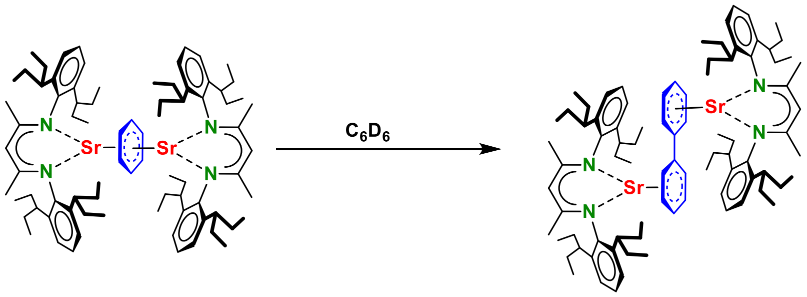

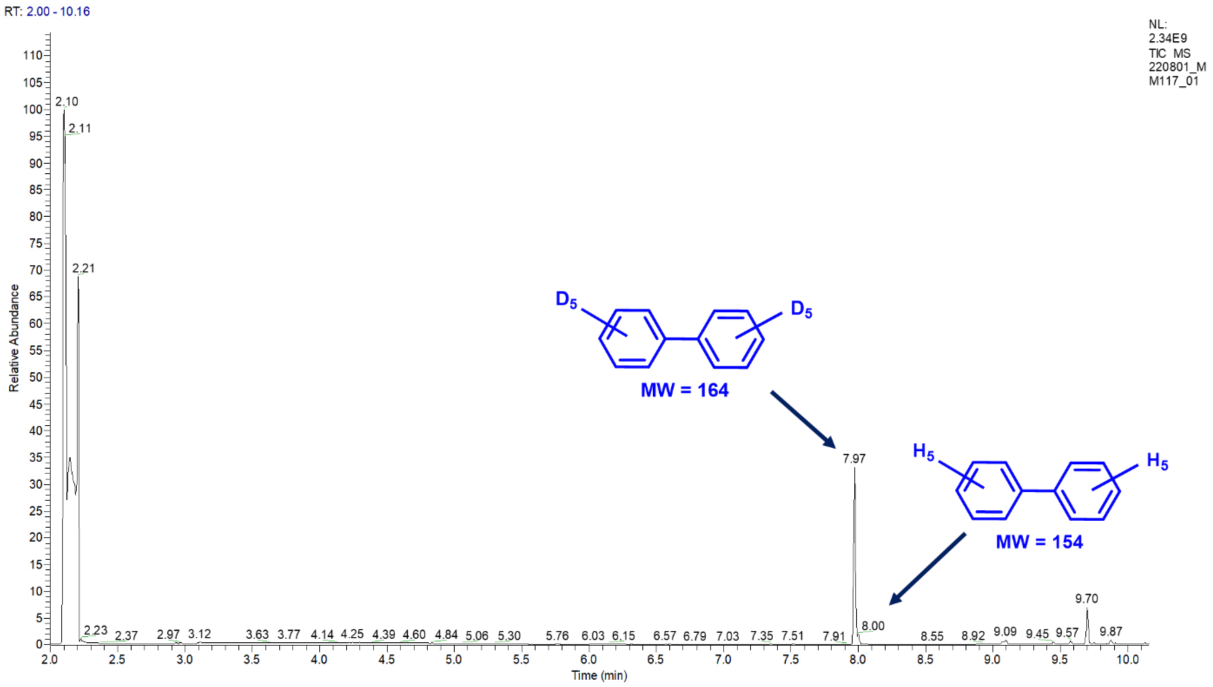

Figure S41. Chromatogram of the reaction of $[(^{DIPeP}BDI)Sr]_2(C_6H_6)$ (15.0 mg, 0.0100 mmol) in C_6D_6 (2.75 mL). After completion of the reaction the reaction mixture was exposed to air, which lead to the rapid decomposition of the air-sensitive $[(^{DIPeP}BDI)Sr]_2$(biphenyl) complex. The chromatogram shows a peak at a retention time (RT) of 7.97 min, which can be attributed to fully deuterated biphenyl-d_{10} based on the mass spectrum depicted in Figure **S43**. The peak appearing at RT = 8.00 min can be attributed to biphenyl-h_{10}. Furthermore, the spectrum shows that the primary product of the biphenyl formation is the deuterated species of biphenyl-d_{10}.

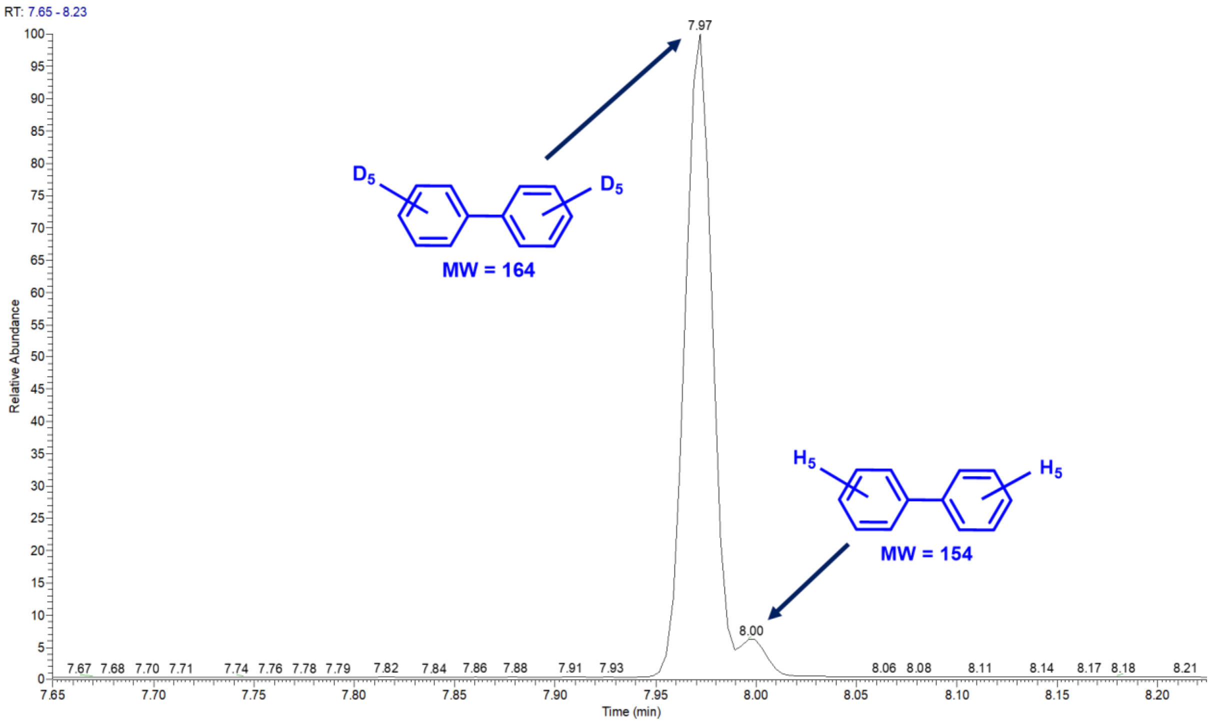

Figure S42. Zoomed in in the chromatogram of the reaction of [(^{DIPeP}BDI)Sr]$_2$(C_6H_6) (15.0 mg, 0.0100 mmol) in C_6D_6 (2.75 mL). The chromatogram shows a peak at a retention time (RT) of 7.97 min, which can be attributed to fully deuterated biphenyl-*d*$_{10}$ based on the mass spectrum depicted in Figure S43. The peak appearing at 8.00 min can be attributed to biphenyl-*h*$_{10}$. Furthermore, the spectrum shows that the primary product of the biphenyl formation is the deuterated specie of biphenyl-*d*$_{10}$.

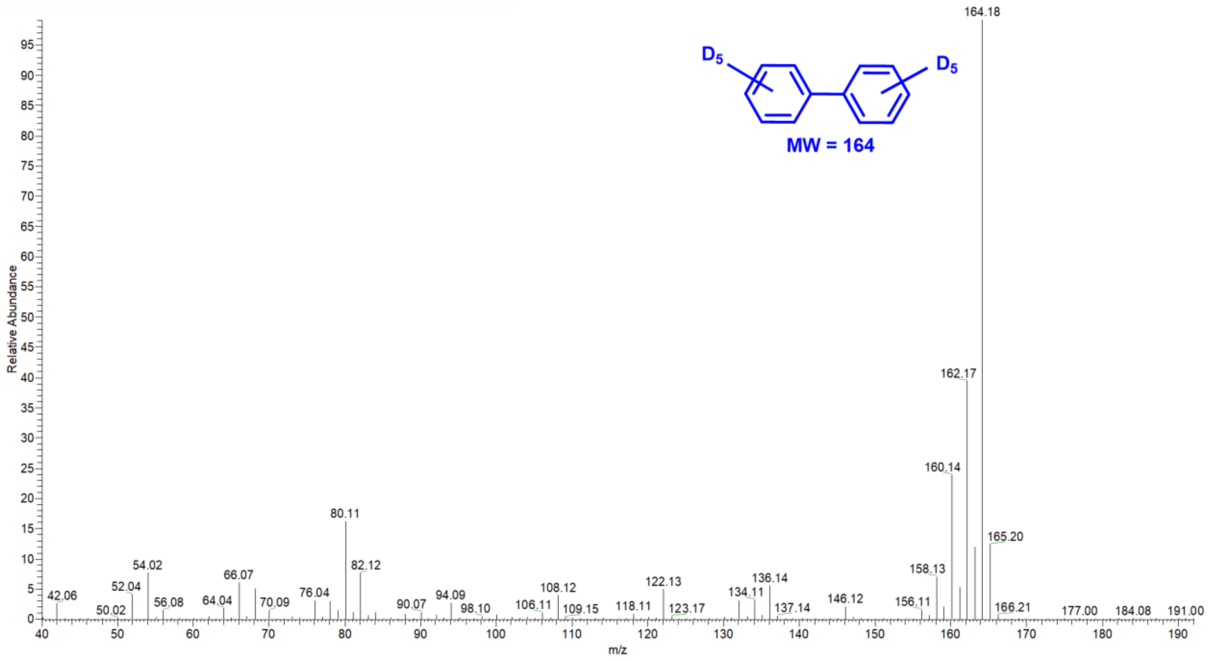

Figure S43. Mass spectrum (RT = 7.97 min) of the reaction of [(^{DIPeP}BDI)Sr]$_2$(C_6H_6) (15.0 mg, 0.0100 mmol) in C_6D_6 (2.75 mL).

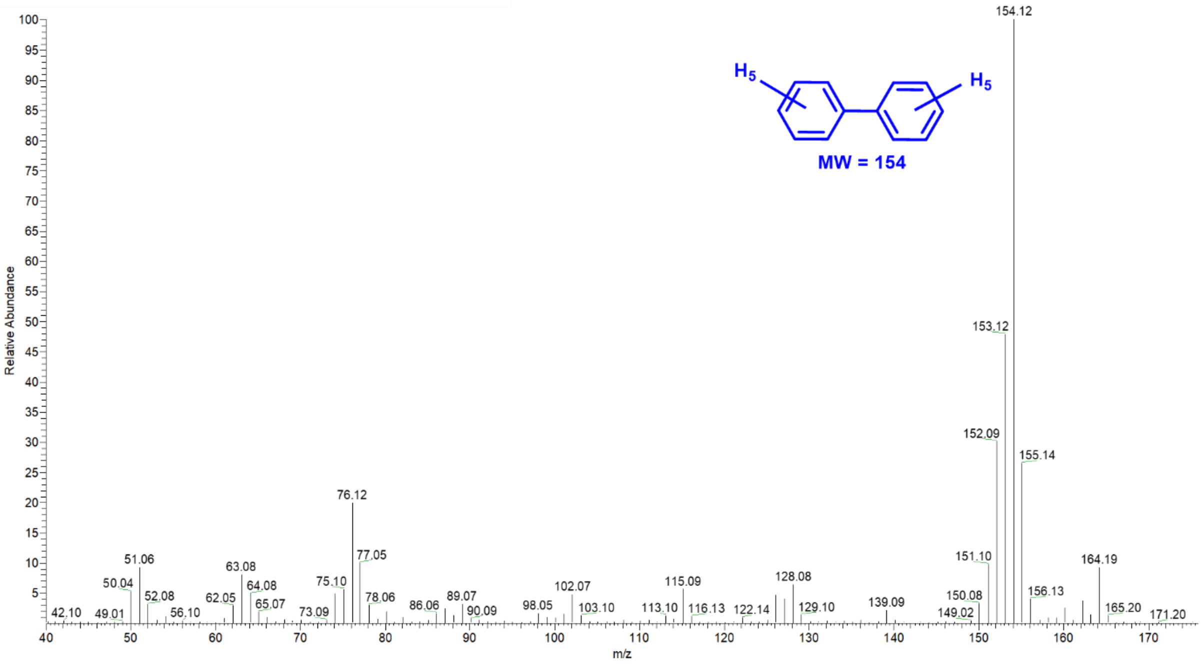

Figure S44. Mass spectrum (RT = 8.00 min) of the reaction of [(^{DIPeP}BDI)Sr]$_2$(C_6H_6) (15.0 mg, 0.0100 mmol) in C_6D_6 (2.75 mL).

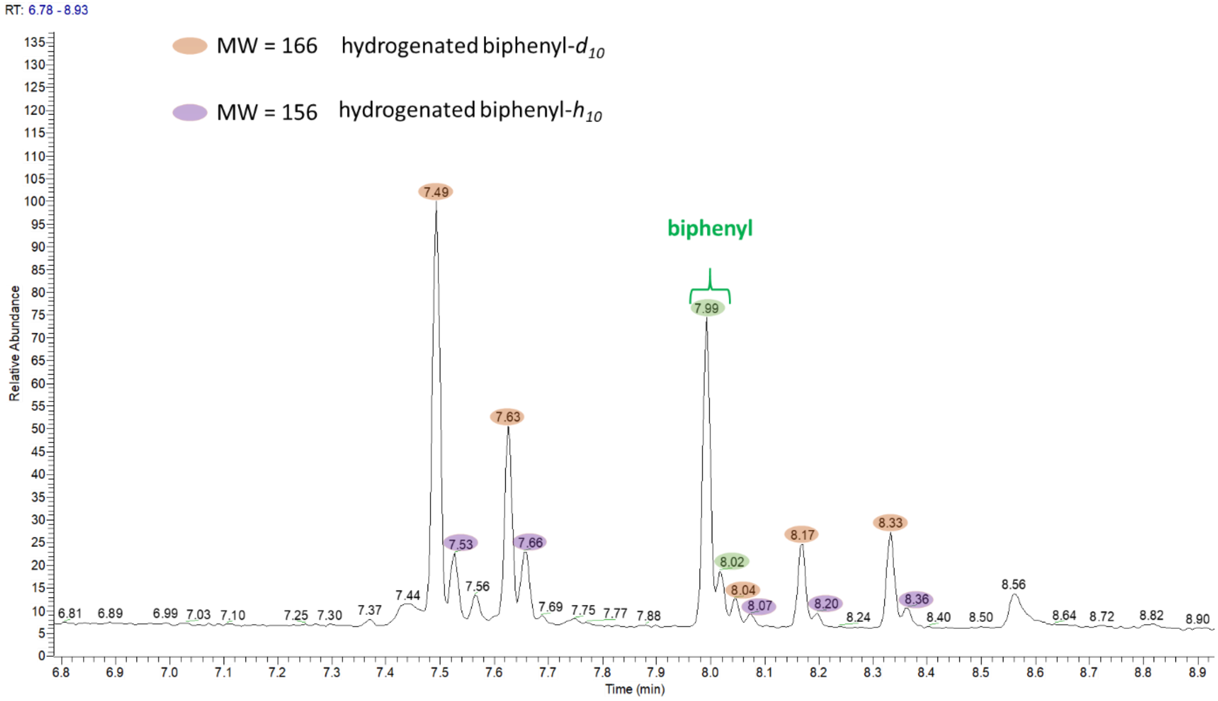

Figure S45. Chromatogram of the reaction of [(^{DIPeP}BDI)Sr]$_2$(C_6H_6) in C_6D_6. After the reaction was finished the mixture was quenched with isopropanol. The hydrogenated biphenyl-d_{10} species are marked in orange, while the hydrogenated biphenyl-h_{10} species are marked in purple. A set of five pairs of signals can be observed for the hydrogenation products.

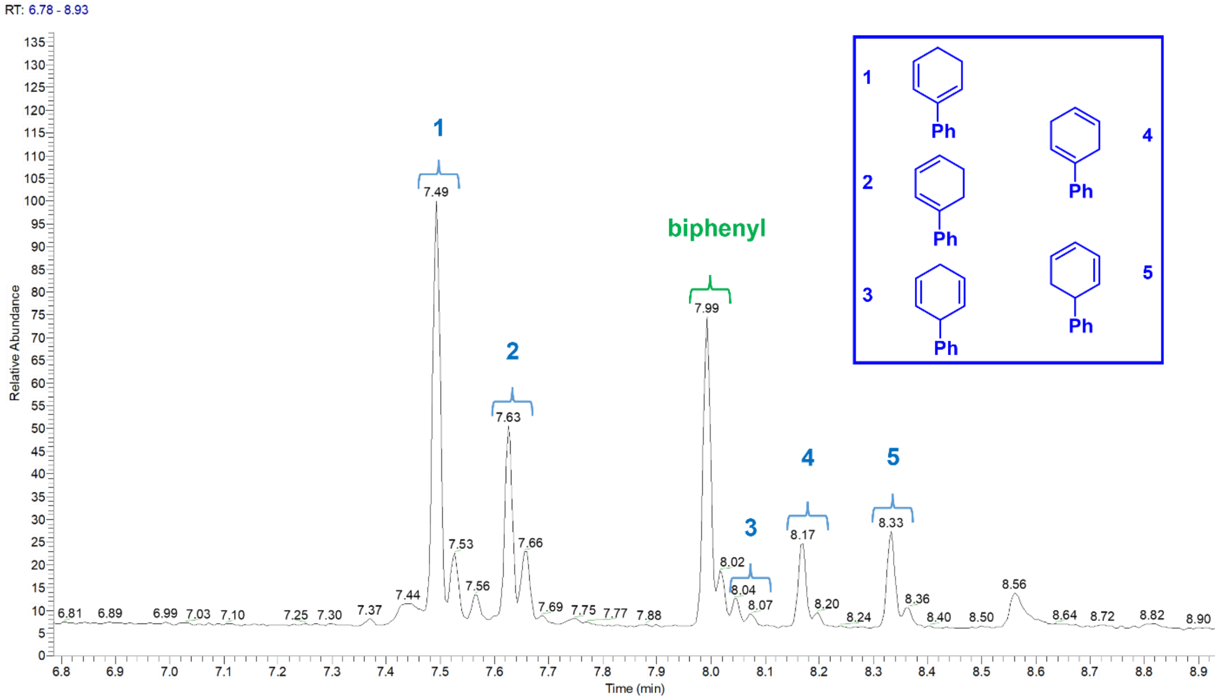

Figure S46. Chromatogram of the reaction of [(^{DIPeP}BDI)Sr]$_2$(C_6H_6) in C_6D_6. After the reaction was finished, the mixture was quenched with isopropanol. The figure shows the assignment of the peaks with their respective hydrogenated biphenyl species (1 – 5). Note that in each case only one phenyl ring was hydrogenated. The molecular identity was confirmed by comparison with entries in the NIST/EPA/NIH mass spectral library (version 2.2, built June 10, 2014).

Reaction of $[(^{DIPeP}BDI)Sr]_2(C_6H_6)$ with a mixture of C_6D_6 and C_6H_6 (ratio 1:1)

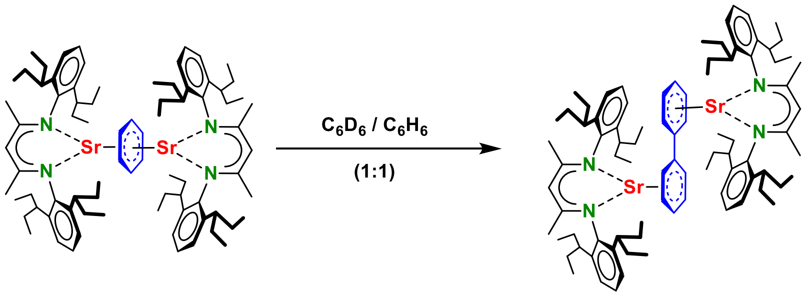

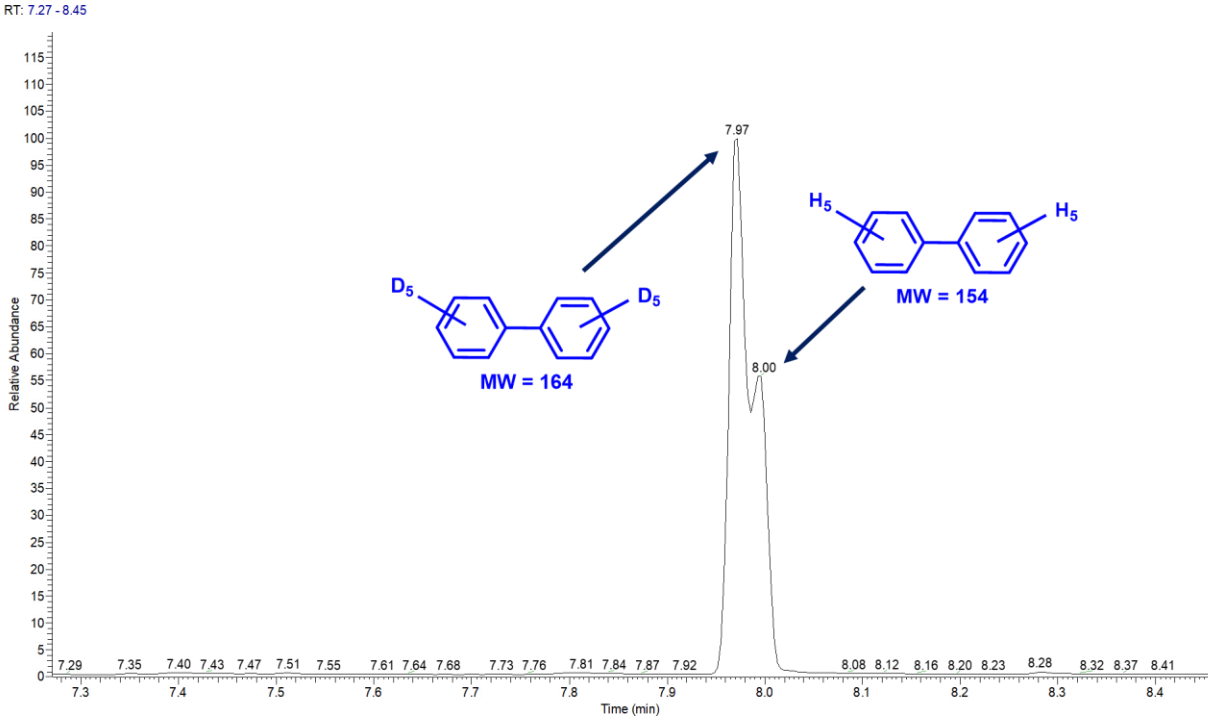

Figure S47. Chromatogram of the reaction of $[(^{DIPeP}BDI)Sr]_2(C_6H_6)$ (15.0 mg, 0.0100 mmol) in a mixture of C_6D_6 and C_6H_6 (2.75 mL, ratio 1:1). The chromatogram shows a peak at a retention time (RT) of 7.97 min, which can be attributed to fully deuterated biphenyl-d_{10}. The peak appearing at RT = 8.00 min can be attributed to biphenyl-h_{10}. In comparison to the reaction in pure C_6D_6 the reaction shows an increased formation of biphenyl-h_{10}. However, biphenyl-d_{10} is again the main product of the reaction. In addition, the mass spectrum at RT = 7.99 min shows the presence of a molecule with a molecular weight of MW = 159. The fragmentation pattern which is depicted in Figure S48 indicates the formation of a mixed biphenyl-d_5h_5 species.[S5]

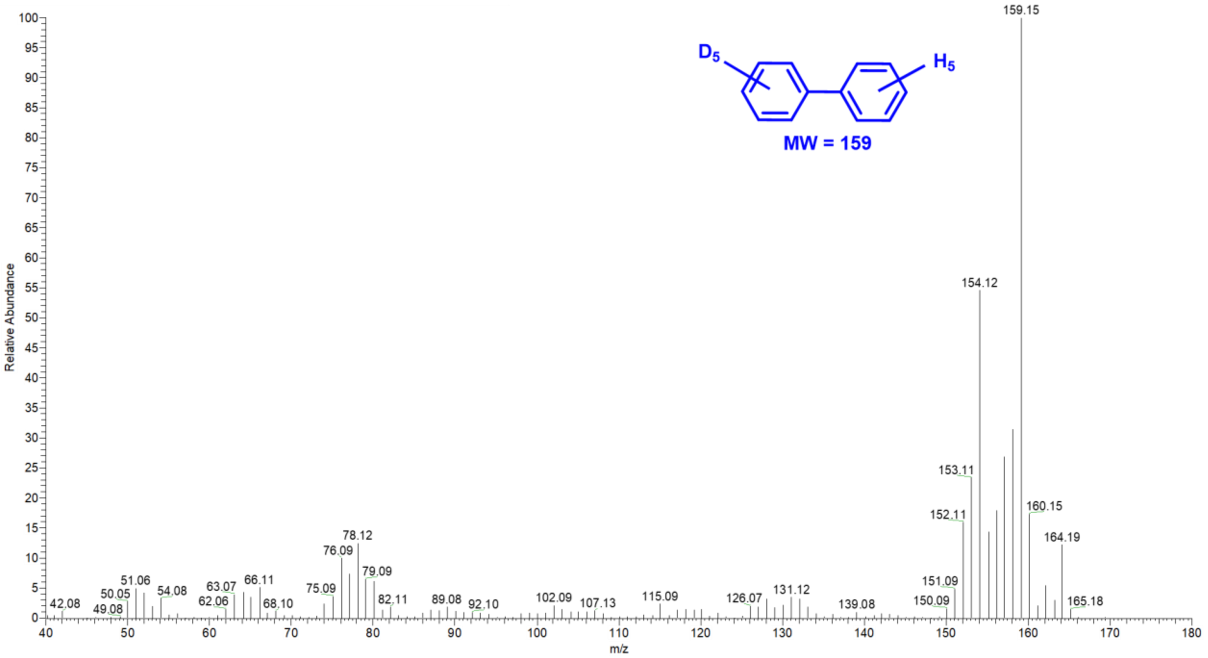

Figure S48. Mass spectrum (RT = 7.99 min) of the reaction of [(^{DIPeP}BDI)Sr]$_2$(C_6H_6) (15.0 mg, 0.0100 mmol) in a mixture of C_6D_6 and C_6H_6 (2.75 mL, ratio 1:1). Based on the fragmentation pattern the molecule was identified as the mixed biphenyl-d_5h_5 species.

Reaction of [(^{DIPeP}BDI)Ca]$_2$(C_6H_6) with C_6D_6

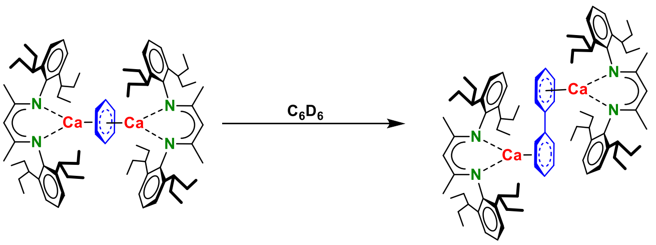

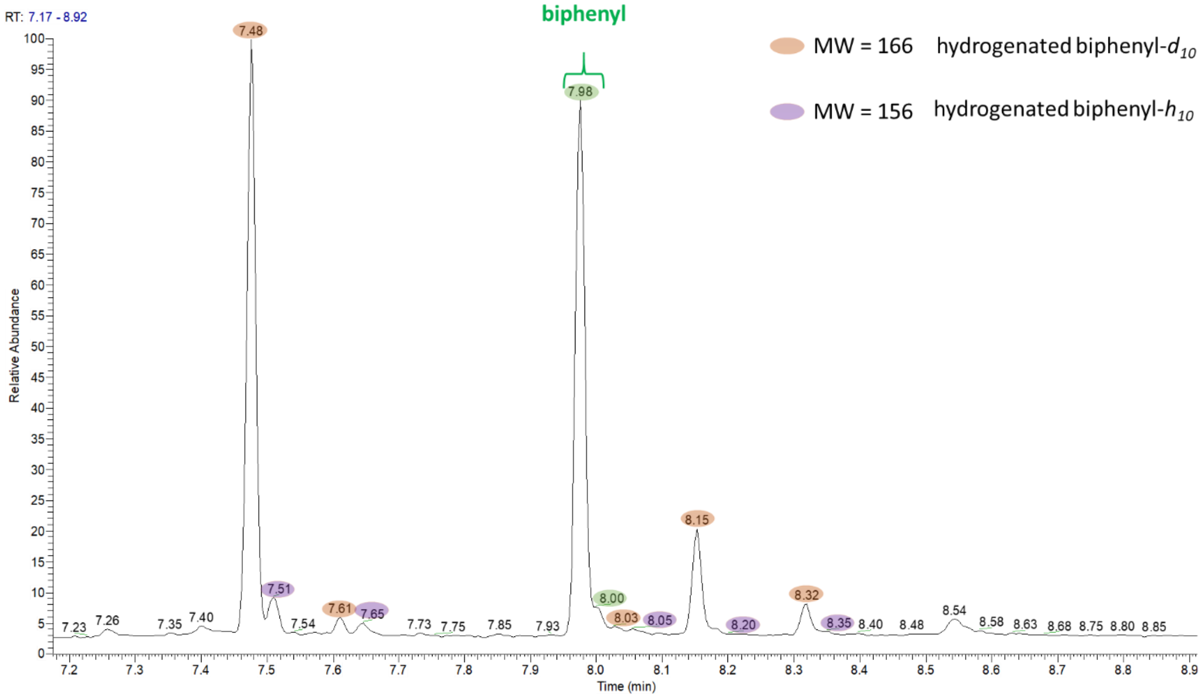

Figure S49. Chromatogram of the reaction of [(^{DIPeP}BDI)Ca]$_2$(C_6H_6) (13.5 mg, 0.0113 mmol) in C_6D_6 (0.550 mL). After the reaction was finished the mixture was quenched with isopropanol. The hydrogenated biphenyl-*d*$_{10}$ species are marked in orange, while the hydrogenated biphenyl-*h*$_{10}$ species are marked in purple.

Reaction of [(^{DIPeP}BDI)Ca]$_2$(C_6H_6)] with a mixture of C_6D_6 and C_6H_6 (ratio 1:1)

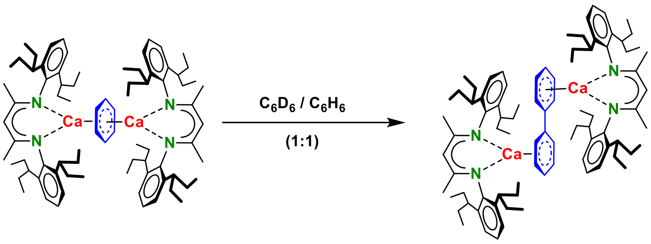

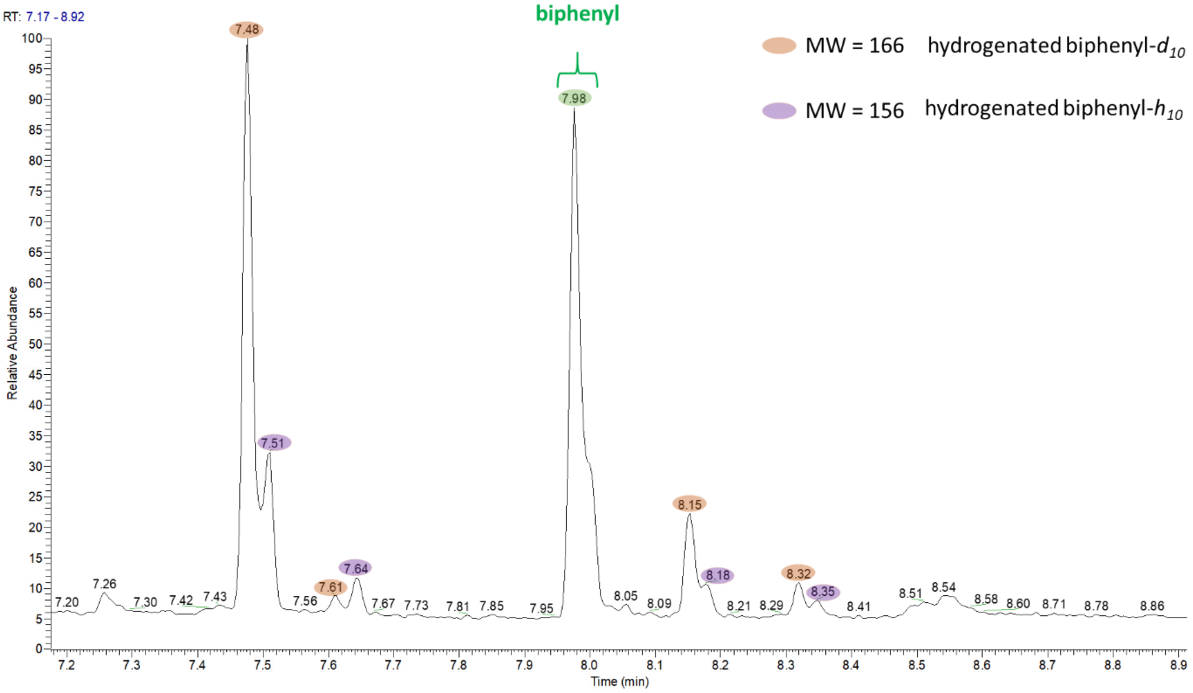

Figure S50. Chromatogram of the reaction of [(^{DIPeP}BDI)Ca]$_2$(C_6H_6) (14.5 mg, 0.0119 mmol) in a mixture of C_6D_6 and C_6H_6 (0.550 mL, ratio 1:1). After the reaction was finished the mixture was quenched with isopropanol. The hydrogenated biphenyl-d_{10} species are marked in orange, while the hydrogenated biphenyl-h_{10} species are marked in purple.

5. Crystal structure determinations

In each case, a crystal of compound **1-5** was embedded in inert perfluoropolyalkylether (viscosity 1800 cSt; ABCR GmbH) and mounted using a Hampton Research CryoLoop. The selected crystal was then flash cooled to 100 K in a nitrogen gas stream and kept at this temperature during the experiment. The crystal structures were measured on an Agilent SuperNova diffractometer with Atlas S2 detector using a CuKα microfocus source. The measured data were processed with the CrysAlisPro software package.[S7] Using Olex2,[S8] the structures were solved with the ShelXT[S9] structure solution program using Intrinsic Phasing and refined with the ShelXL[S10] refinement package using Least Squares Minimization. All non-hydrogen atoms were refined anisotropically. Most hydrogen atoms were placed in ideal positions and refined as riding atoms with relative isotropic displacement parameters.

Complex $[(^{DIPeP}BDI)Ca(THF)]_2$(biphenyl) (**1**-THF), crystallized with half of a molecule per asymmetric unit. The THF ligand showed disorder, which was treated using similarity restraints (SADI) and rigid bond restraints (RIGU).[S11] The relative occupancies of the two alternative orientations were refined to 0.853(12) and 0.147(12).

In case of compound $[(^{DIPP}BDI)Ca(THF)]_2$(biphenyl) (**2**-THF), disorder of two of the *i*Pr groups was observed and treated with the help of similarity restraints (SADI). The relative occupancies of the two alternative orientations of each group were refined to 0.770(12)/0.230(12) and 0.579(18)/0.421(18), respectively.

Complex $[(^{DIPP}BDI)Ca(THP)]_2$(biphenyl) (**2**-THP) crystallized with half of a molecule per asymmetric unit. The THP ligand, *i*Pr groups and one Me group showed disorder, which was treated using similarity restraints (SADI, SIMU). The relative occupancies of the two alternative orientations were refined to 0.53(2)/0.47(2) (Me), 0.82(2)/0.18(2) (*i*Pr), 0.673(19)/0.327(19) (*i*Pr), and 0.930(2)/0.070(2) (THP), respectively. Additionally, the position of H3 in the ligand backbone deviated noticeably from the idealized position and was therefore refined freely.

Disorder was also present in case of derivative $[(^{DIPeP}BDI)Sr(\mu\text{-}I)]_2$ (**3**). One Aryl ring with an attached 3-pentyl moiety, three additional 3-pentyl groups as well as both iodide ligands were affected. The disorder was modelled with the help of similarity restraints (SADI, SIMU) and rigid bond restraints (RIGU).[S11] The relative occupancies of the two alternative orientations of each group were refined to 0.522(7)/0.478(7) (Ar + 3-pentyl), 0.728(7)/0.272(7) (3-pentyl), 0.562(8)/0.438(8) (3-pentyl), 0.755(10)/0.245(10) (3-pentyl) and 0.776(3)/0.224(3) (iodide), respectively. The second iodide is disordered over three positions with site occupancy factors (sof's) of 0.776(3), 0.149(3) and 0.075(3).

In case of compound $[(^{DIPeP}BDI)Sr]_2(C_6H_6)$ (**4**), the positions of the hydrogen atoms of the central $C_6H_6^{2-}$ ligand were observed from difference Fourier maps and refined. Disorder of two of the 3-pentyl groups

was observed and treated with the help of similarity restraints (SADI, SIMU) and rigid bond restraints (RIGU).[S11] The relative occupancies of the alternative orientations of each group were refined to 0.696(7)/0.304(7) and 0.567(3)/0.433(3), respectively. However, an additional orientation of one ethyl group of the second disordered 3-pentyl group was detected and consequently three different orientations with site occupancy factors of 0.567(3), 0.222(3) and 0.211(3) were refined for this part of the structure.

Complex [(^{DIPeP}BDI)Sr]$_2$(biphenyl) (**5**) crystallized with three symmetry-independent half-molecules per asymmetric unit. Disorder of one biphenyl subunit, a DIPeP-N moiety, four additional 3-pentyl groups and two methyl groups was detected and treated with the help of similarity restraints (SADI, SIMU) and rigid bond restraints (RIGU).[S11] The relative occupancies of the two alternative orientations of each group were refined to 0.640(19)/0.360(19) (biphenyl), 0.551(4)/0.449(4) (DIPeP-N), 0.710(7)/0.290(7) (3-pentyl), 0.618(4)/0.382(4) (3-pentyl), 0.760(4)/0.240(4) (3-pentyl), 0.702(5)/0.298(5) (3-pentyl) and 0.55(3)/0.45(3) (2 x Me), respectively. Additionally, the co-crystallized solvent (a mixture of *n*-pentane, hexanes (isomeric mixture), methylcyclohexane and toluene was used for crystallization) was severely disordered. A suitable disorder model for these solvent molecules could not be built. Therefore, their contribution to the structure factors was secured by back-Fourier transformation using the solvent mask routine[S12,S13] of the program Olex2.[S8] The solvent accessible voids treated this way had a size of 543.9 Å^3 (8.9% of the unit cell) and contained 115.8 electrons/unit cell.

Crystallographic and refinement data are summarized in Table S1.

The crystal structure data of the compounds have been deposited with the Cambridge Crystallographic Data Centre. CCDC 2202205 [(^{DIPeP}BDI)Ca(THF)]$_2$(biphenyl) (**1**-THF), 2202206 [(^{DIPP}BDI)Ca(THF)]$_2$(biphenyl) (**2**-THF), 2202207 [(^{DIPP}BDI)Ca(THP)]$_2$(biphenyl) (**2**-THP), 2202208 [(^{DIPeP}BDI)Sr(μ-I)]$_2$ (**3**), 2202209 [(^{DIPeP}BDI)Sr]$_2$(C_6H_6) (**4**) and 2202210 [(^{DIPeP}BDI)Sr]$_2$(biphenyl) (**5**) contain the supplementary crystallographic data for the compounds. This data can be obtained free of charge from The Cambridge Crystallographic Data Centre via www.ccdc.cam.ac.uk/data_request/cif.

Table S1. Crystal data and structure refinement for compounds **1-5**.

Compound	[(^{DIPeP}BDI)Ca(THF)]$_2$(biphenyl) (**1**-THF)	[(^{DIPP}BDI)Ca(THF)]$_2$(biphenyl)·C_6H_6 (**2**-THF)	[(^{DIPP}BDI)Ca(THP)]$_2$(biphenyl)·C_6H_6 (**2**-THP)
Identification code	hasj220317c	hasj160120a	hasj220322a
Empirical formula	$C_{94}H_{140}Ca_2N_4O_2$	$C_{84}H_{114}Ca_2N_4O_2$	$C_{86}H_{118}Ca_2N_4O_2$
Formula weight	1438.25	1291.95	1320.00
Temperature/K	100.0(2)	100(1)	100.0(2)
Crystal system	triclinic	triclinic	triclinic
Space group	P-1	P-1	P-1
a/Å	12.8704(9)	9.6888(2)	9.6849(2)
b/Å	12.9986(10)	18.1667(3)	12.3764(3)
c/Å	14.4650(10)	23.0332(4)	17.3405(4)
α/°	79.863(6)	108.956(2)	74.110(2)
β/°	83.938(6)	95.646(2)	89.6894(19)
γ/°	60.863(8)	101.372(2)	71.395(2)
Volume/Å^3	2080.2(3)	3700.88(13)	1886.94(8)
Z	1	2	1
ρ_{calc}g/cm^3	1.148	1.159	1.162
μ/mm^{-1}	1.560	1.704	1.680
F(000)	788.0	1404.0	718.0
Crystal size/mm^3	0.304 × 0.161 × 0.021	0.361 × 0.215 × 0.076	0.613 × 0.216 × 0.191
Radiation	Cu Kα (λ = 1.54184)	Cu Kα (λ = 1.54184)	Cu Kα (λ = 1.54184)
2Θ range for data collection/°	6.208 to 144.946	7.782 to 147.28	5.32 to 145.194
Index ranges	-15 ≤ h ≤ 15, -10 ≤ k ≤ 16, -17 ≤ l ≤ 17	-11 ≤ h ≤ 9, -22 ≤ k ≤ 22, -28 ≤ l ≤ 28	-11 ≤ h ≤ 9, -15 ≤ k ≤ 15, -21 ≤ l ≤ 17
Reflections collected	15208	42549	12508
Independent reflections	7925 [R_{int} = 0.0481, R_{sigma} = 0.0646]	14603 [R_{int} = 0.0341, R_{sigma} = 0.0332]	7262 [R_{int} = 0.0235, R_{sigma} = 0.0325]
Data/restraints/parameters	7925/22/489	14603/34/909	7262/664/554
Goodness-of-fit on F^2	1.036	1.048	1.039
Final R indexes [I>=2σ (I)]	R_1 = 0.0557, wR_2 = 0.1458	R_1 = 0.0345, wR_2 = 0.0946	R_1 = 0.0312, wR_2 = 0.0793
Final R indexes [all data]	R_1 = 0.0704, wR_2 = 0.1597	R_1 = 0.0377, wR_2 = 0.0974	R_1 = 0.0342, wR_2 = 0.0815
Largest diff. peak/hole / e Å^{-3}	0.88/-0.46	0.35/-0.28	0.22/-0.30

Table S1. Crystal data and structure refinement for compounds **1-5** (continued).

Compound	[(^{DIPeP}BDI)Sr(μ-I)]$_2$ (**3**)	[(^{DIPeP}BDI)Sr]$_2$(C_6H_6) (**4**)	[(^{DIPeP}BDI)Sr]$_2$(biphenyl)·solvent (**5**)
Identification code	hasj200123b	hasj220505a	hasj220525a
Empirical formula	$C_{74}H_{114}I_2N_4Sr_2$	$C_{80}H_{120}N_4Sr_2$	$C_{86}H_{124}N_4Sr_2$ $^{a)}$
Formula weight	1488.73	1313.03	1389.12 $^{a)}$
Temperature/K	100.0(6)	100.0(1)	100.0(2)
Crystal system	monoclinic	monoclinic	triclinic
Space group	I2/a	P2$_1$/c	P-1
a/Å	28.4079(3)	11.32376(20)	17.6023(2)
b/Å	12.30640(10)	29.1714(4)	18.6147(3)
c/Å	42.2132(4)	22.4192(5)	18.7068(2)
α/°	90	90	90.0610(10)
β/°	93.7560(10)	100.469(2)	90.5610(10)
γ/°	90	90	94.5690(10)
Volume/Å^3	14726.0(2)	7282.4(2)	6109.72(14)
Z	8	4	3
ρ_{calc}g/cm^3	1.343	1.198	1.133 $^{a)}$
μ/mm^{-1}	8.791	2.250	2.038 $^{a)}$
F(000)	6144.0	2816.0	2232.0 $^{a)}$
Crystal size/mm^3	0.213 × 0.097 × 0.046	0.393 × 0.323 × 0.186	0.318 × 0.253 × 0.236
Radiation	Cu Kα (λ = 1.54184)	Cu Kα (λ = 1.54184)	Cu Kα (λ = 1.54184)
2Θ range for data collection/°	7.286 to 145.676	6.06 to 145.15	6.652 to 145.21
Index ranges	-34 ≤ h ≤ 32, -15 ≤ k ≤ 14, -52 ≤ l ≤ 51	-12 ≤ h ≤ 13, -34 ≤ k ≤ 35, -27 ≤ l ≤ 18	-21 ≤ h ≤ 21, -23 ≤ k ≤ 23, -22 ≤ l ≤ 23
Reflections collected	55409	27544	91655
Independent reflections	14281 [R_{int} = 0.0473, R_{sigma} = 0.0348]	13976 [R_{int} = 0.0272, R_{sigma} = 0.0373]	23677 [R_{int} = 0.0294, R_{sigma} = 0.0238]
Data/restraints/parameters	14281/1606/1026	13976/650/926	23677/814/1662
Goodness-of-fit on F^2	1.024	1.079	1.019
Final R indexes [I>=2σ (I)]	R_1 = 0.0415, wR_2 = 0.1082	R_1 = 0.0400, wR_2 = 0.0914	R_1 = 0.0299, wR_2 = 0.0741
Final R indexes [all data]	R_1 = 0.0476, wR_2 = 0.1134	R_1 = 0.0477, wR_2 = 0.0950	R_1 = 0.0337, wR_2 = 0.0773
Largest diff. peak/hole / e Å^{-3}	0.91/-0.89	0.81/-1.23	0.59/-1.00

a) Contribution of the masked disordered solvent neglected.

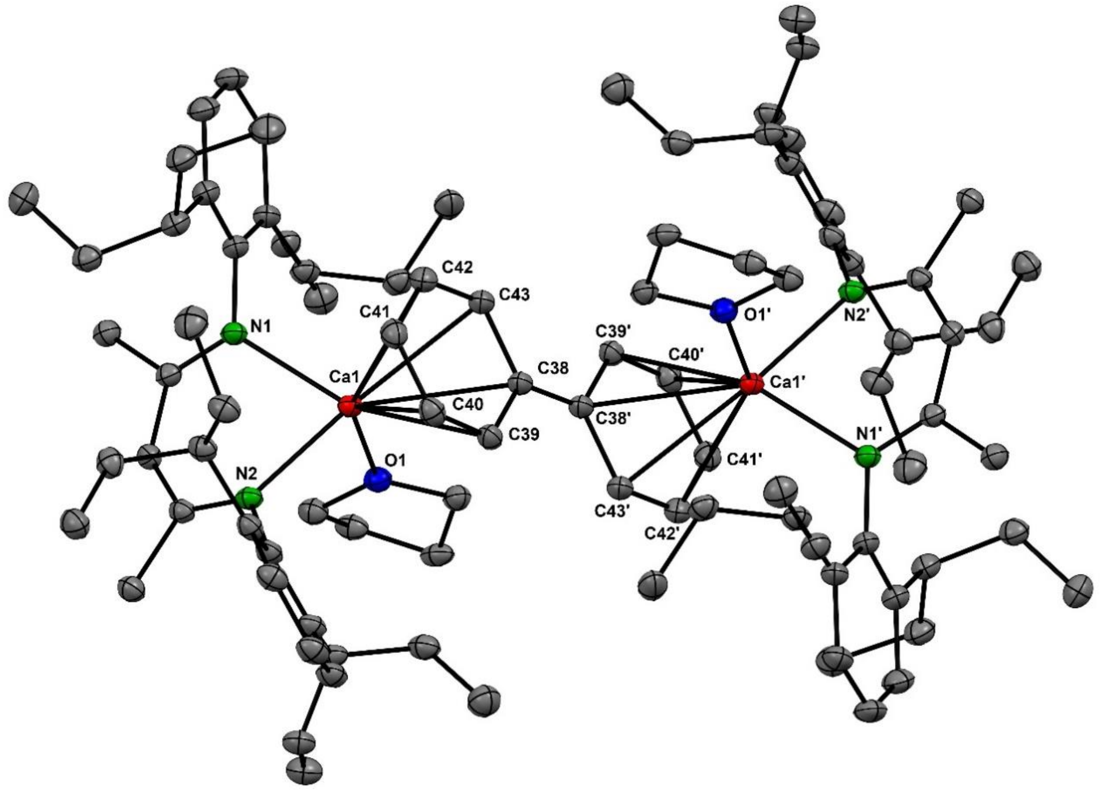

Figure S51. Molecular structure of [(^{DIPeP}BDI)Ca(THF)]$_2$(biphenyl) (**1**-THF). Ellipsoids represent 30% probability. Hydrogen atoms and disorder have been omitted for clarity. ^{1}1-X,1-Y,1-Z.

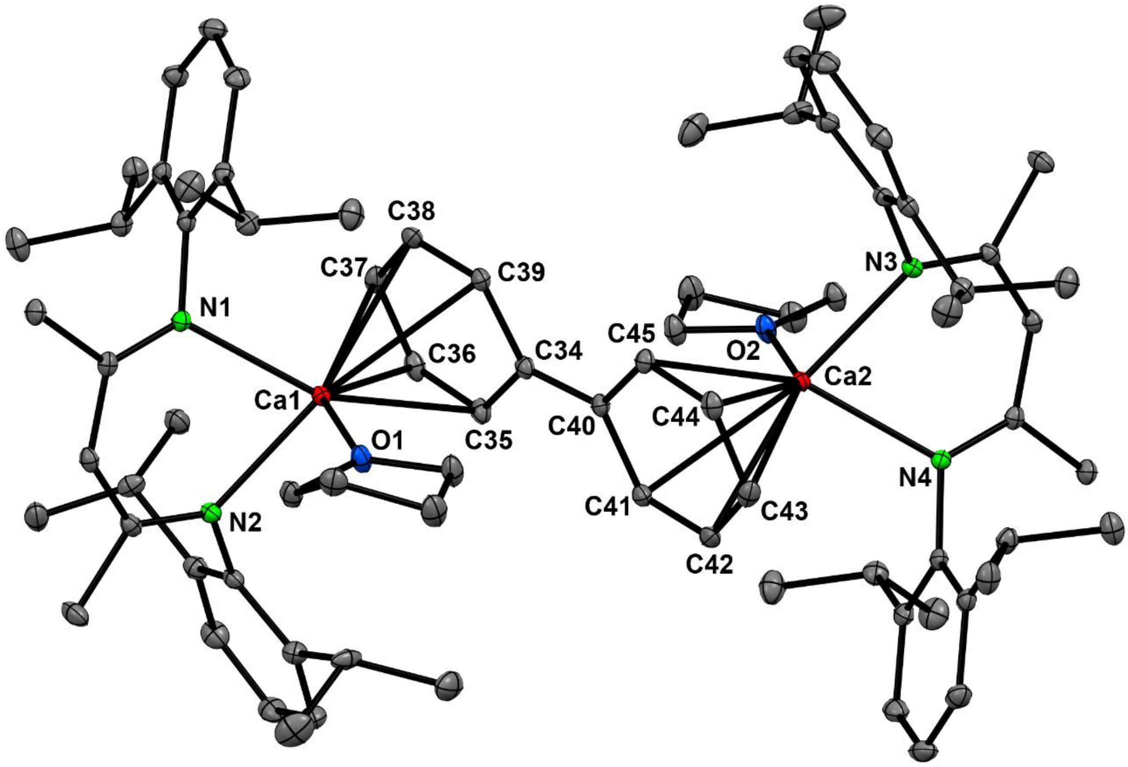

Figure S52. Molecular structure of [(^{DIPP}BDI)Ca(THF)]$_2$(biphenyl) (**2**-THF). Ellipsoids represent 30% probability. Disorder, hydrogen atoms and co-crystallized benzene have been omitted for clarity.

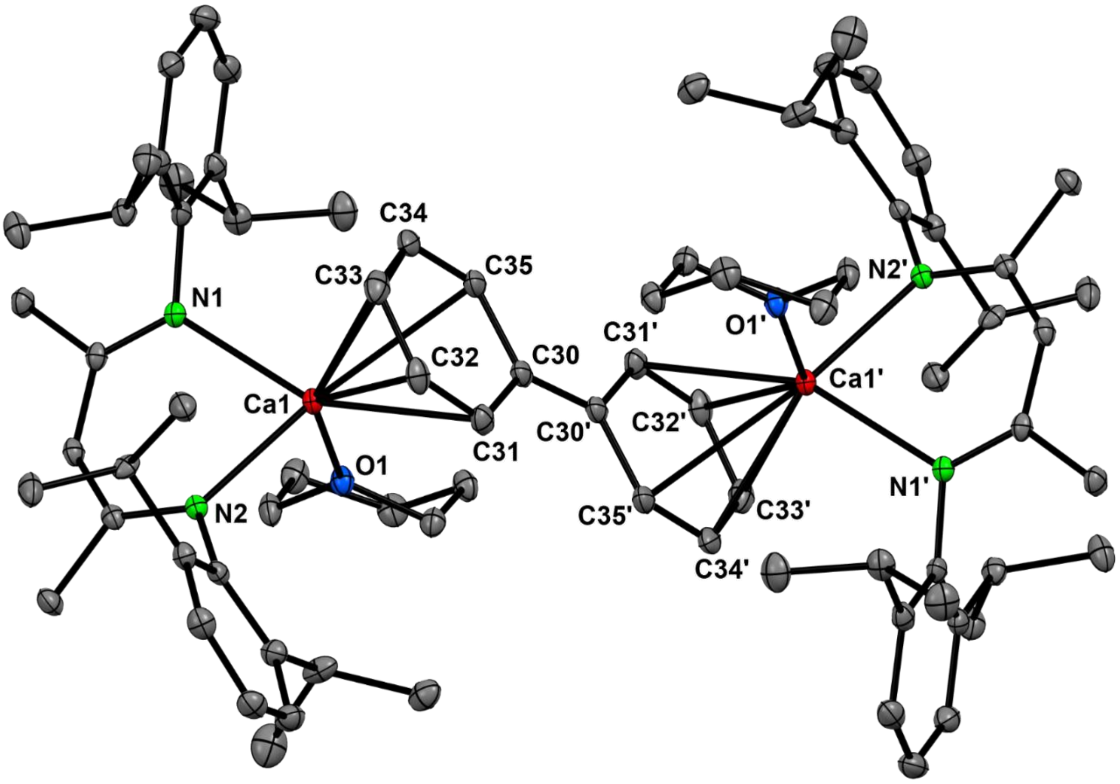

Figure S53. Molecular structure of [(^{DIPP}BDI)Ca(THP)]$_2$(biphenyl) (**2**-THP). Ellipsoids represent 30% probability. Disorder, hydrogen atoms and co-crystallized benzene have been omitted for clarity. ′1-X,1-Y,1-Z.

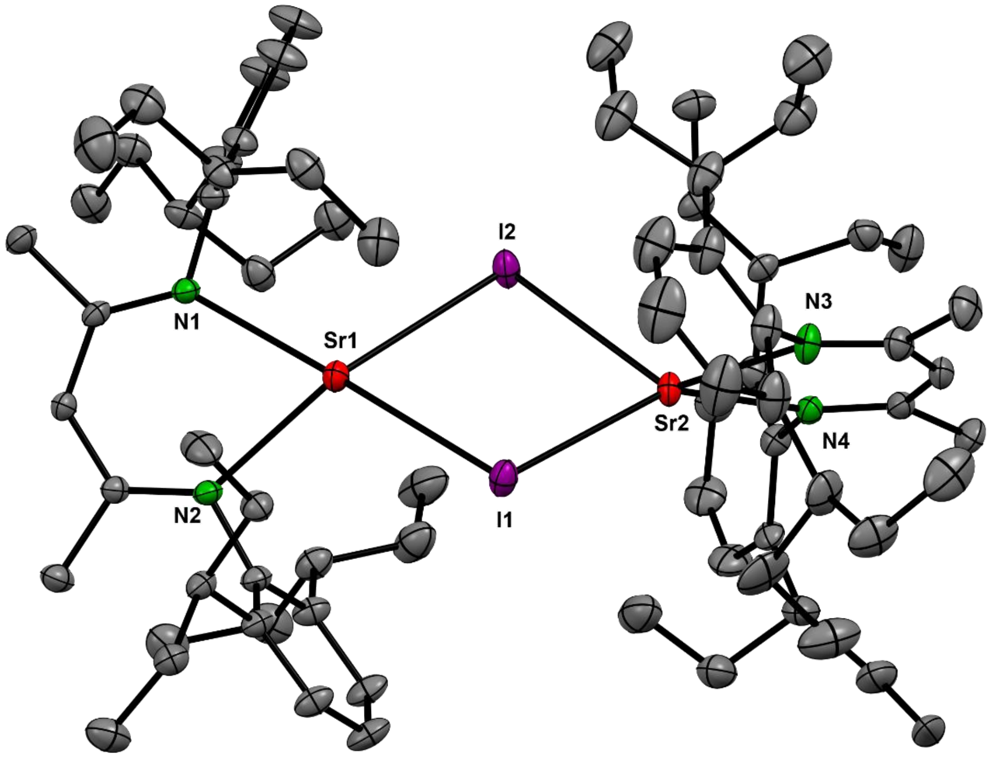

Figure S54. Molecular structure of [(^{DIPeP}BDI)SrI]$_2$ (**3**). Ellipsoids represent 30% probability. Hydrogen atoms and disorder have been omitted for clarity.

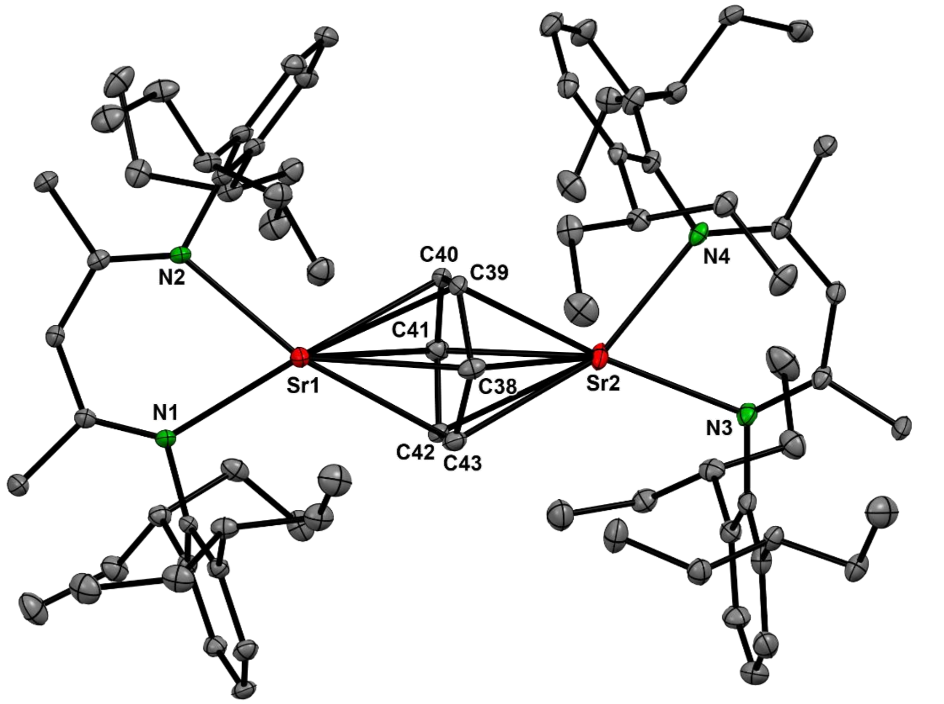

Figure S55. Molecular structure of [(^{DIPeP}BDI)Sr]$_2$(C_6H_6) (**4**). Ellipsoids represent 30% probability. Hydrogen atoms and disorder have been omitted for clarity.

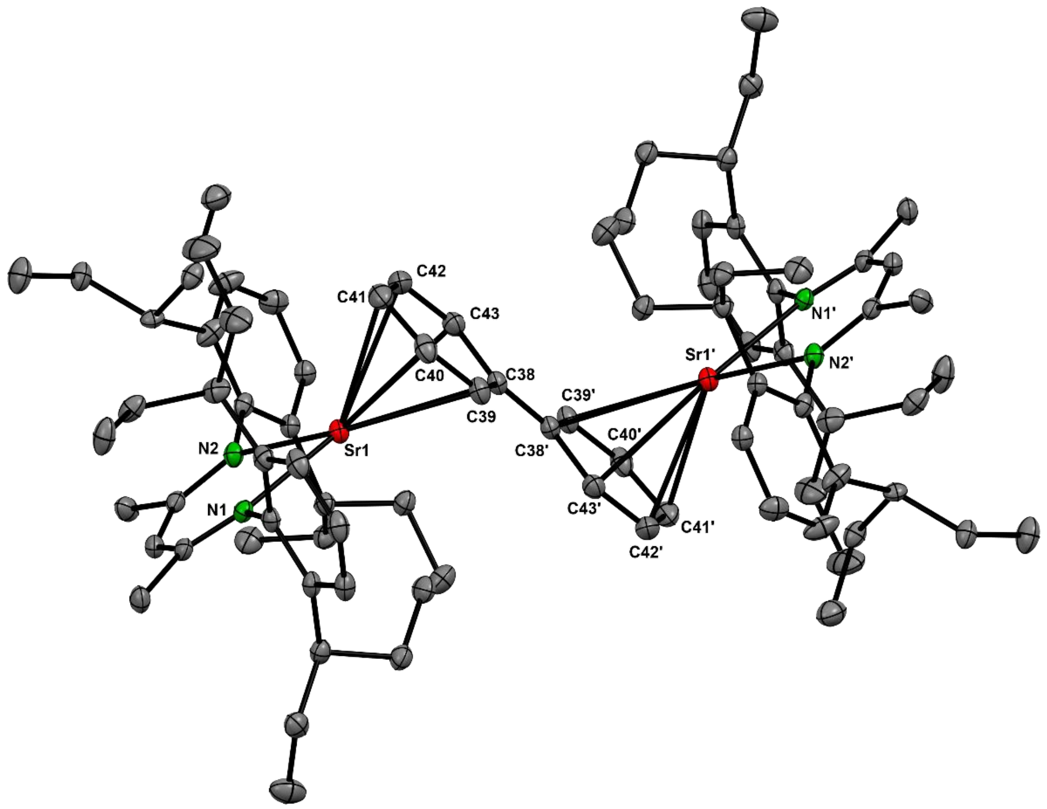

Figure S56. Molecular structure of [(^{DIPeP}BDI)Sr]$_2$(biphenyl) (**5**). Ellipsoids represent 30% probability. Hydrogen atoms and disorder have been omitted for clarity. 1-X,-Y,1-Z; 1-X,1-Y,-Z; -X,-Y,-Z.

6. DFT calculation

All calculations were carried out using Gaussian 16A.[S14] All methods were used as implemented. All structures were fully optimized at a B3PW91/def2SVP level of theory.[S15–S18] All structures were characterized as true minima (Nimag=0) or as transition states (Nimag=1) by frequency calculations on the same level of theory. Energies were determined at a B3PW91/def2TZVP level of theory. In all cases Grimme`s third dispersion correction with Becke-Johnson damping (GD3BJ) was applied.[S19] Charges were calculated via NBO7 Analyses.[S20] All structures were evaluated using Molecule 2.3.[S21]

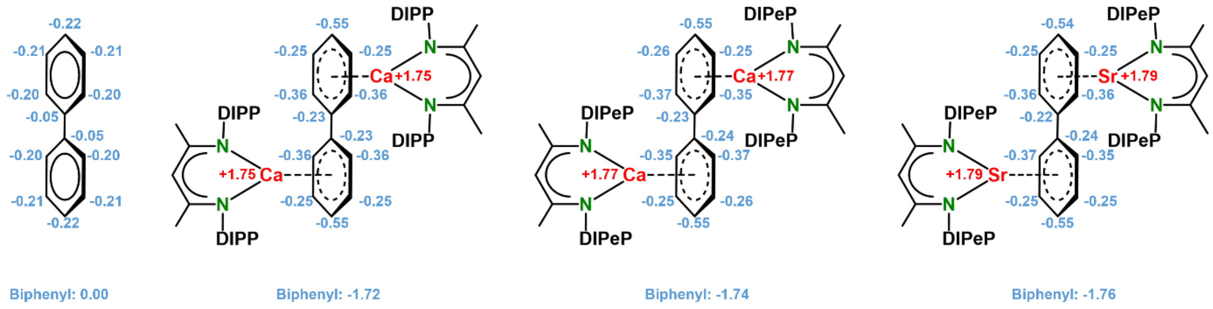

Figure S57. Calculated NPA charges in free biphenyl compared to that in the biphenyl complexes, $[(^{DIPP}BDI)Ca]_2$(biphenyl) and $[(^{DIPeP}BDI)M]_2$(biphenyl) (M=Ca, Sr).

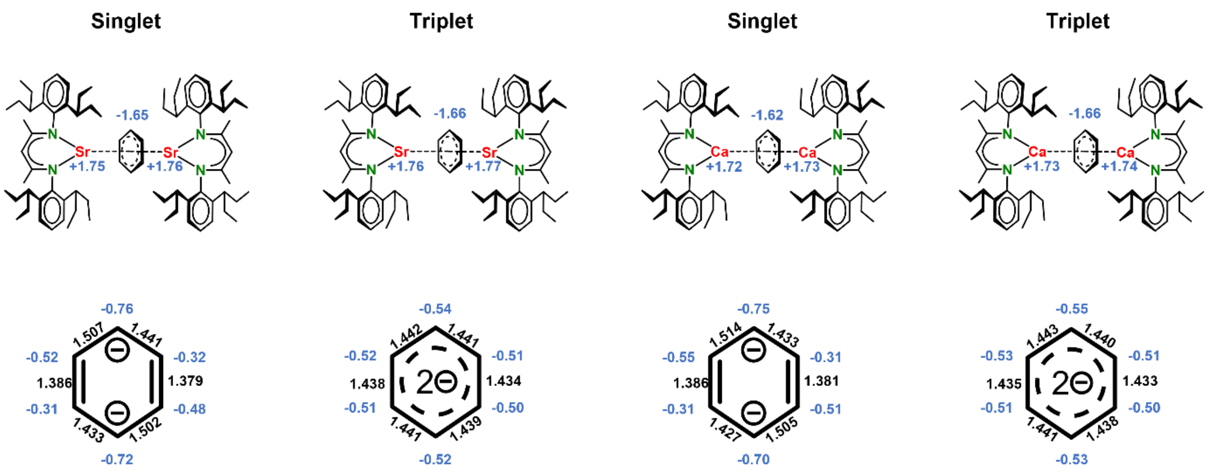

Figure S58. Calculated structures of $[(^{DIPeP}BDI)M]_2(C_6H_6)$ (M = Ca, Sr). Charges given in blue, calculated distances in black. As both complexes show paramagnetic behavior, both complexes were also calculated as a triplet state. In the singlet state, the electron density of the $(C_6H_6)^{2-}$ is mainly on two carbon atoms in *para*-position to each other, in the case of the triplet state the negative charge is delocalized over the complete ring.

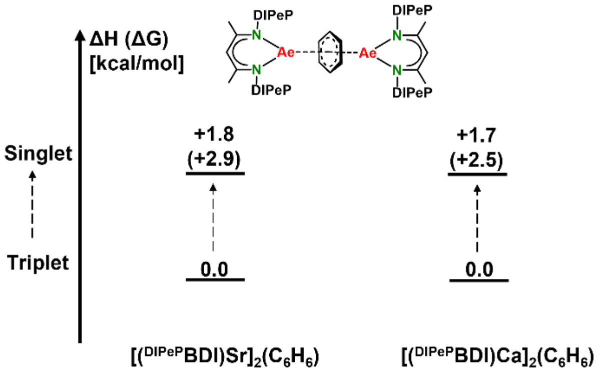

Figure S59. Calculated energy gap between singlet and triplet state of the benzene structures $[(^{DIPeP}BDI)M]_2(C_6H_6)$ (M = Ca, Sr). In both cases the gap is very low, and the triplet state is the more stable one.

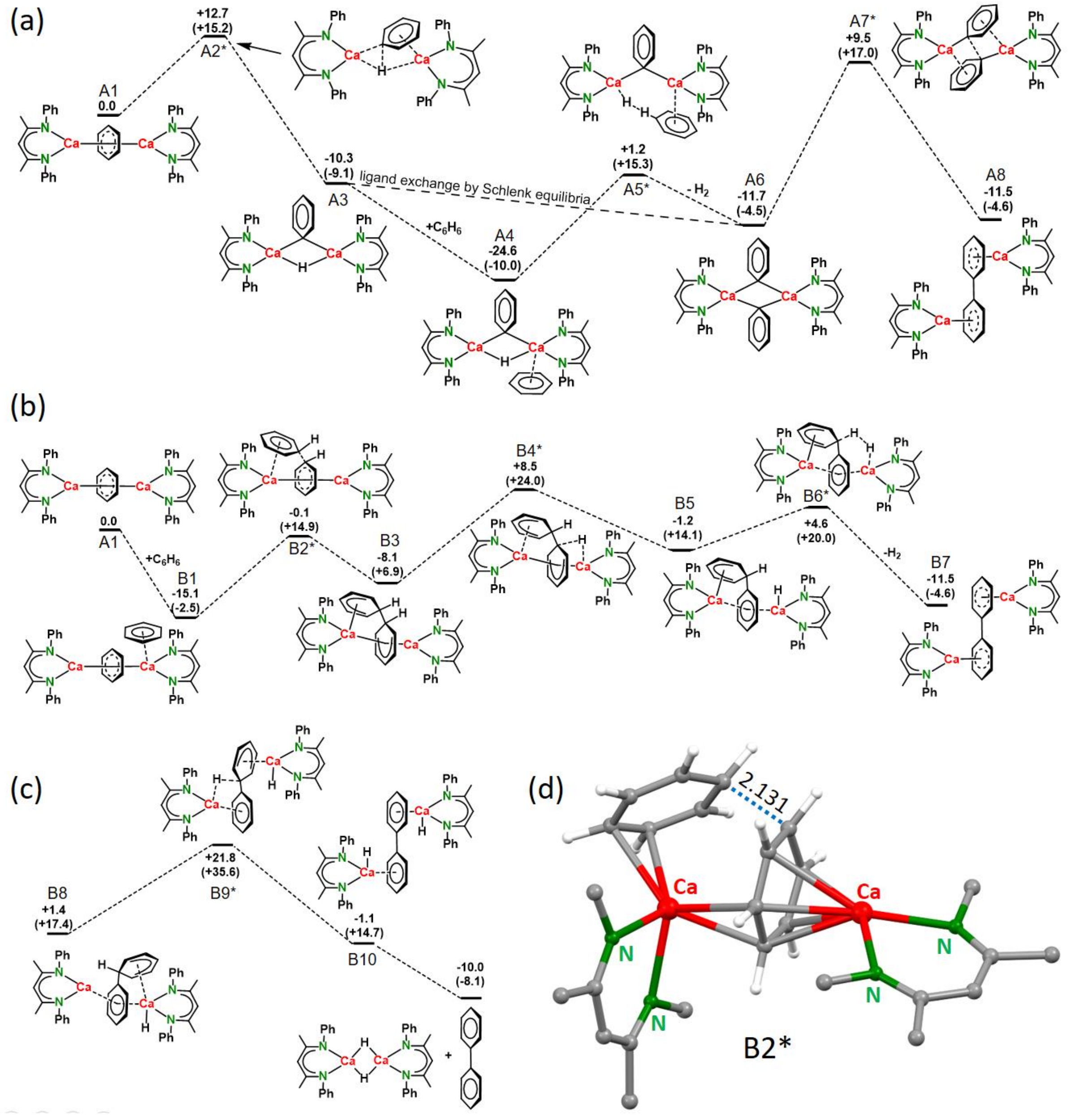

Figure S60. Energy profiles (ΔH(ΔG) in kcal/mol) for benzene-benzene coupling calculated at the B3PW91/def2tzvp//def2svp level of theory with a model system with Ph-substituents. (a) Pathway A *via* Ph^{-}-Ph^{-} coupling. (b) Pathway B *via* $C_6H_6^{2-}$→ benzene attack. (c) Alternative formation of neutral biphenyl and $[(BDI)Ca(\mu\text{-}H)]_2$. (d) Transition state B2* for C-C coupling (Ph groups and H atoms partially omitted for clarity).

XYZ-coordinates

22

Biphenyl

C 0.740232 0.000076 -0.000087
C 1.461108 -1.138724 -0.393964
H 0.919567 -2.026505 -0.728743
C 2.854558 -1.139242 -0.393955
H 3.395448 -2.033740 -0.712801
C 3.557881 -0.000047 0.000038
H 4.650505 -0.000111 0.000104
C 2.854620 1.139200 0.393999
H 3.395548 2.033654 0.712893
C 1.461170 1.138790 0.393898
H 0.919676 2.026623 0.728622
C -0.740231 0.000078 -0.000056
C -1.461198 1.138784 -0.393952
H -0.919744 2.026629 -0.728716
C -2.854655 1.139178 -0.393940
H -3.395615 2.033633 -0.712780
C -3.557881 -0.000069 0.000058
H -4.650506 -0.000107 0.000081
C -2.854523 -1.139264 0.394014
H -3.395380 -2.033760 0.712914
C -1.461080 -1.138729 0.393909
H -0.919501 -2.026501 0.728650

168

[(^{DIPP}BDI)Ca]$_2$(biphenyl)

Ca -3.153995 -0.003058 -0.055489
N -4.822085 1.428116 0.729315
N -4.820464 -1.433487 0.730884
C -6.622447 2.471271 2.027335
H -7.642702 2.216115 2.341107
H -6.662694 3.272799 1.276690
H -6.093042 2.883882 2.901578
C -5.884834 1.261259 1.502624
C -6.370430 -0.003453 1.889928
H -7.252010 -0.003629 2.531293
C -5.883155 -1.267988 1.504261
C -6.618197 -2.478591 2.031092
H -6.649745 -3.284720 1.284993
H -7.641641 -2.226583 2.337029
H -6.092431 -2.883069 2.911362
C -4.392858 2.699133 0.305379
C -4.670783 3.100257 -1.026731
C -4.127245 4.302251 -1.486735
H -4.332739 4.631790 -2.506768
C -3.309941 5.083802 -0.671447
H -2.888523 6.016669 -1.053641
C -3.024291 4.668250 0.625953
H -2.370633 5.278353 1.254079
C -3.557303 3.480945 1.136338
C -5.522725 2.212361 -1.918759
H -5.232538 1.170825 -1.689471
C -7.014944 2.327884 -1.592387
H -7.606472 1.687945 -2.266094
H -7.232459 2.011993 -0.564747
C -5.289526 2.431762 -3.410676
H -5.811030 1.654938 -3.990188
H -5.681630 3.404660 -3.747859
C -3.191431 2.995468 2.527206
H -3.920800 2.222343 2.807280
C -1.814701 2.329160 2.519592
H -1.561496 1.906094 3.503704
H -1.744400 1.505753 1.788928
C -3.260332 4.099164 3.582033
H -3.094853 3.682061 4.587502
H -2.490927 4.870435 3.420071
C -4.387546 -2.702878 0.306195
C -4.663954 -3.103416 -1.026470
C -4.115382 -4.302457 -1.488161
H -4.319487 -4.631376 -2.508677
C -3.294817 -5.081707 -0.673942
H -2.869396 -6.012212 -1.057457
C -3.011058 -4.666894 0.624117
H -2.354942 -5.275198 1.251431
C -3.548935 -3.482493 1.136159
C -3.185130 -2.997436 2.527672
H -3.915852 -2.225494 2.807457
C -1.809231 -2.329343 2.522038
H -1.738901 -1.505561 1.791734
H -1.557984 -1.906237 3.506635
C -3.253926 -4.102058 3.581551
H -4.233966 -4.602902 3.573173
H -2.483793 -4.872572 3.419462
C -0.470927 0.000528 -0.522320
C -1.008144 -1.227680 -1.116007
H -0.656653 -2.194942 -0.759228
C -1.949320 -1.209716 -2.122787
H -2.305212 -2.168147 -2.507812
C -2.530683 0.002730 -2.599411
H -3.241080 0.003732 -3.425225
C -1.951450 1.214377 -2.118061
H -2.309226 2.173649 -2.499221
C -1.010131 1.230082 -1.111426
H -0.660035 2.196573 -0.751167
C -5.520354 -2.218098 -1.916750
H -5.235427 -1.175503 -1.685641
C -5.286940 -2.433730 -3.409163
H -5.674193 -3.408063 -3.747785
H -5.812826 -1.658628 -3.987010
C -7.011743 -2.341907 -1.589550
H -7.229883 -2.030362 -0.560717
H -7.607033 -1.702871 -2.260781
Ca 3.153292 0.002570 0.054576
N 4.821790 -1.428049 -0.730110
N 4.820022 1.433621 -0.730531
C 6.622160 -2.470685 -2.028532
H 7.642365 -2.215327 -2.342296
H 6.662549 -3.272328 -1.278006
H 6.092773 -2.883253 -2.902805
C 5.884405 -1.260888 -1.503528
C 6.369783 0.003998 -1.890509
H 7.251281 0.004487 -2.531986

C 5.882670 1.268365 -1.504040
C 6.617947 2.479163 -2.030118
H 6.650022 3.284624 -1.283322
H 7.641208 2.227056 -2.336583
H 6.092056 2.884630 -2.909855
C 4.392830 -2.699131 -0.306107
C 4.671031 -3.100202 1.025970
C 4.127717 -4.302237 1.486095
H 4.333388 -4.631707 2.506118
C 3.310391 -5.083922 0.670943
H 2.889143 -6.016827 1.053236
C 3.024512 -4.668459 -0.626429
H 2.370869 -5.278678 -1.254453
C 3.557280 -3.481074 -1.136921
C 5.523170 -2.212231 1.917717
H 5.233126 -1.170722 1.688195
C 7.015284 -2.328139 1.591006
H 7.607146 -1.688034 2.264261
H 7.232537 -2.012736 0.563157
C 5.290135 -2.431305 3.409697
H 5.811775 -1.654424 3.989009
H 5.682167 -3.404174 3.747045
C 3.191122 -2.995669 -2.527741
H 3.920287 -2.222379 -2.807896
C 1.814233 -2.329681 -2.519955
H 1.560752 -1.906772 -3.504063
H 1.743837 -1.506187 -1.789392
C 3.260149 -4.099324 -3.582600
H 3.094488 -3.682232 -4.588045
H 2.490920 -4.870760 -3.420594
C 4.387610 2.702963 -0.305156
C 4.664398 3.102852 1.027609
C 4.116510 4.302003 1.489842
H 4.320960 4.630434 2.510446
C 3.296183 5.081952 0.676059
H 2.871280 6.012526 1.059981
C 3.012006 4.667751 -0.622108
H 2.356096 5.276618 -1.249091
C 3.549261 3.483311 -1.134694
C 3.185078 2.999012 -2.526371
H 3.915608 2.227079 -2.806703
C 1.809062 2.331155 -2.520763
H 1.738853 1.506872 -1.791022
H 1.557407 1.908801 -3.505579
C 3.253843 4.104140 -3.579734
H 4.233935 4.604876 -3.571254
H 2.483813 4.874655 -3.417157
C 0.470486 -0.000788 0.521962
C 1.007794 1.227401 1.115603
H 0.656226 2.194680 0.758954
C 1.949193 1.209375 2.122187
H 2.305132 2.167779 2.507240
C 2.530616 -0.003090 2.598645
H 3.241158 -0.004124 3.424332
C 1.951192 -1.214730 2.117482
H 2.309006 -2.173997 2.498620
C 1.009642 -1.230363 1.111063
H 0.659315 -2.196825 0.750934
C 5.520480 2.216774 1.917433
H 5.234978 1.174428 1.685951
C 5.287315 2.431939 3.409958
H 5.675166 3.405903 3.748959
H 5.812786 1.656290 3.987454
C 7.011892 2.339925 1.590125
H 7.229767 2.028575 0.561175
H 7.606913 1.700365 2.261100
H -1.021347 -3.047104 2.252247
H -3.089522 -3.685688 4.587499
H -7.353501 -3.381609 -1.716764
H -4.220751 -2.383526 -3.672516
H -4.222918 2.387611 -3.673478
H -7.361841 3.366225 -1.716795
H -1.028213 3.048133 2.248892
H -4.240753 4.599286 3.574855
H 1.021414 3.048890 -2.250188
H 3.089248 3.688269 -4.585857
H 7.354219 3.379405 1.717630
H 4.221112 2.382266 3.673356
H 7.362022 -3.366501 1.715706
H 4.223548 -2.386988 3.672549
H 1.027974 -3.048814 -2.249036
H 4.240674 -4.599241 -3.575532

216

[(^{DIPeP}BDI)Ca]$_2$(biphenyl)

Ca 3.112170 0.059344 0.213746
N 4.871692 -1.344389 0.903151
N 4.658759 1.714046 0.834800
C 6.750098 -2.061059 2.312430
H 7.497892 -1.635206 2.991693
H 7.270448 -2.573518 1.491125
H 6.187536 -2.838272 2.844128
C 5.824650 -0.997163 1.769235
C 6.085128 0.315220 2.213162
H 6.876406 0.389773 2.959356
C 5.619114 1.559918 1.738304
C 6.307733 2.767333 2.338293
H 6.193159 3.660766 1.713254
H 7.376771 2.571732 2.495037
H 5.867127 2.991339 3.323067
C 4.874545 -2.682044 0.426805
C 5.500047 -2.970327 -0.812798
C 5.569954 -4.306217 -1.224872
H 6.081003 -4.553945 -2.156429
C 5.002193 -5.329607 -0.472117
H 5.074177 -6.365504 -0.811823
C 4.326068 -5.024317 0.704843
H 3.857541 -5.827818 1.276062
C 4.244250 -3.708658 1.171962
C 6.091848 -1.856170 -1.663919
H 5.396474 -1.001374 -1.581152
C 7.446740 -1.350943 -1.127706
H 7.785329 -0.536755 -1.790331
H 7.292224 -0.878325 -0.147891
C 8.536267 -2.409736 -1.028165
H 8.216586 -3.260179 -0.405302

H 9.450702 -1.991527 -0.580125
H 8.811710 -2.816220 -2.013667
C 6.217665 -2.202608 -3.155064
H 6.680925 -1.331237 -3.648843
H 6.944874 -3.021568 -3.285251
C 4.928239 -2.553194 -3.884842
H 4.406868 -3.401195 -3.417007
H 5.133380 -2.826733 -4.931330
H 4.230165 -1.705052 -3.900920
C 3.433111 -3.376837 2.412853
H 3.852003 -2.454577 2.845240
C 1.978304 -3.044953 2.018493
H 1.438672 -2.688418 2.912065
H 1.983977 -2.194882 1.312719
C 1.210994 -4.189982 1.373041
H 0.165617 -3.912084 1.179686
H 1.183348 -5.078429 2.021522
H 1.663346 -4.487615 0.415932
C 3.470302 -4.447276 3.510763
H 2.788057 -4.121184 4.314584
H 3.042339 -5.391112 3.136203
C 4.846996 -4.720719 4.097347
H 4.802058 -5.509418 4.864006
H 5.265109 -3.821153 4.575895
H 5.556480 -5.049952 3.322503
C 4.339121 3.001707 0.339480
C 4.830632 3.391740 -0.933032
C 4.463775 4.647426 -1.427477
H 4.852331 4.979541 -2.391097
C 3.624531 5.495547 -0.709233
H 3.353458 6.472192 -1.117132
C 3.148356 5.101786 0.536887
H 2.513812 5.782602 1.106800
C 3.492748 3.859773 1.083097
C 2.999504 3.457169 2.459521
H 3.582327 2.575752 2.762263
C 1.532266 2.984659 2.450812
H 1.417215 2.212673 1.669431
H 1.325860 2.465173 3.400991
C 0.484538 4.066745 2.236279
H -0.527537 3.655786 2.352844
H 0.551579 4.508132 1.231756
H 0.587528 4.882773 2.967996
C 3.284746 4.543653 3.507111
H 4.342555 4.837945 3.411446
H 2.708740 5.455740 3.280473
C 3.002192 4.107394 4.937908
H 1.932762 3.906743 5.104892
H 3.306099 4.882675 5.657827
H 3.552158 3.185265 5.188976
C 0.495996 -0.003065 -0.492415
C 1.042773 1.223831 -1.075110
H 0.614269 2.182893 -0.794559
C 2.087919 1.213106 -1.971296
H 2.460772 2.167813 -2.346730
C 2.747127 0.004858 -2.346263
H 3.548190 0.006734 -3.081144
C 2.144919 -1.213025 -1.914663
H 2.563783 -2.165504 -2.248760
C 1.100957 -1.230319 -1.015086
H 0.713762 -2.193580 -0.691632
C 5.790269 2.491388 -1.695977
H 5.401513 1.460989 -1.602175
C 5.922332 2.807036 -3.192609
H 6.342312 3.818873 -3.322095
H 6.693051 2.127192 -3.593880
C 4.665446 2.668475 -4.038728
H 4.272960 1.642632 -4.009407
H 4.875516 2.909444 -5.092196
H 3.861186 3.338821 -3.702332
C 7.192017 2.481001 -1.046911
H 7.132392 1.982245 -0.070401
H 7.841157 1.835034 -1.661646
C 7.839966 3.849271 -0.887906
H 7.216340 4.521658 -0.278949
H 8.003888 4.346074 -1.856872
H 8.820511 3.763358 -0.394859
Ca -3.111069 -0.062308 -0.210302
N -4.865490 1.346568 -0.903535
N -4.658745 -1.713211 -0.838782
C -6.739240 2.067912 -2.316690
H -7.485359 1.644143 -2.999087
H -7.261648 2.578988 -1.495825
H -6.174499 2.845823 -2.844990
C -5.816420 1.002022 -1.772909
C -6.077517 -0.309257 -2.219896
H -6.866511 -0.381144 -2.968749
C -5.615668 -1.555625 -1.745400
C -6.304602 -2.760811 -2.349461
H -6.198880 -3.653869 -1.722301
H -7.371442 -2.560943 -2.515321
H -5.856699 -2.988302 -3.330144
C -4.868083 2.683579 -0.425191
C -5.496234 2.970673 0.813337
C -5.565848 4.306016 1.227259
H -6.078881 4.552829 2.157965
C -4.995442 5.329998 0.477344
H -5.067255 6.365459 0.818416
C -4.316987 5.025814 -0.698555
H -3.846495 5.829732 -1.267567
C -4.235266 3.710781 -1.167450
C -6.091569 1.855966 1.661246
H -5.397088 1.000390 1.579040
C -7.445699 1.353381 1.120663
H -7.787251 0.538820 1.781285
H -7.289177 0.881853 0.140638
C -8.533473 2.413826 1.019569
H -8.210978 3.264605 0.398619
H -9.447303 1.997442 0.568605
H -8.810947 2.819467 2.004848
C -6.220934 2.200260 3.152587
H -6.686278 1.328560 3.643823
H -6.947744 3.019680 3.282100
C -4.933140 2.548601 3.886304
H -4.409744 3.396789 3.421076
H -5.140843 2.820834 4.932627

H -4.235887 1.699802 3.903046
C -3.421588 3.380343 -2.407058
H -3.840111 2.459068 -2.841876
C -1.967985 3.046741 -2.009796
H -1.426564 2.690973 -2.902589
H -1.976213 2.195644 -1.305337
C -1.201258 4.190346 -1.361081
H -0.156516 3.911559 -1.165436
H -1.171229 5.079542 -2.008433
H -1.655786 4.487261 -0.404777
C -3.455346 4.452591 -3.503323
H -2.771508 4.127159 -4.306060
H -3.027376 5.395422 -3.126252
C -4.830354 4.728368 -4.092760
H -4.782769 5.518336 -4.857953
H -5.248209 3.830055 -4.573871
H -5.541382 5.057007 -3.319080
C -4.345181 -3.002357 -0.343308
C -4.843400 -3.391846 0.926734
C -4.483726 -4.649563 1.421312
H -4.877620 -4.981112 2.382969
C -3.645162 -5.500355 0.705473
H -3.379878 -6.478640 1.113256
C -3.162082 -5.106995 -0.538112
H -2.527844 -5.789670 -1.106139
C -3.498964 -3.862881 -1.084221
C -2.997344 -3.460292 -2.457597
H -3.575891 -2.576771 -2.762477
C -1.528789 -2.992229 -2.440043
H -1.416297 -2.220402 -1.658169
H -1.314832 -2.473589 -3.389002
C -0.485854 -4.077538 -2.218557
H 0.528284 -3.670078 -2.329434
H -0.560346 -4.518011 -1.214140
H -0.587104 -4.893732 -2.950338
C -3.279905 -4.544752 -3.508001
H -4.338991 -4.836362 -3.418322
H -2.707541 -5.458649 -3.279445
C -2.988542 -4.107565 -4.936746
H -1.917701 -3.909587 -5.097769
H -3.290660 -4.881185 -5.659201
H -3.534687 -3.183672 -5.189660
C -0.497205 -0.002393 0.500362
C -1.044754 -1.229255 1.082523
H -0.615504 -2.188333 0.803020
C -2.091766 -1.218346 1.976545
H -2.465561 -2.172912 2.351499
C -2.751374 -0.009907 2.350321
H -3.553748 -0.011645 3.083762
C -2.147281 1.207859 1.920936
H -2.565866 2.160404 2.255163
C -1.101631 1.225017 1.023364
H -0.712812 2.188229 0.701686
C -5.803038 -2.488851 1.686469
H -5.411398 -1.459466 1.593680
C -5.940426 -2.803866 3.182719
H -6.364330 -3.814198 3.311174
H -6.709793 -2.121232 3.581856
C -4.685164 -2.669739 4.031929
H -4.289416 -1.645117 4.004010
H -4.898389 -2.910602 5.084784
H -3.882289 -3.342430 3.696901
C -7.202674 -2.475138 1.032910
H -7.138674 -1.976648 0.056516
H -7.852223 -1.827539 1.645476
C -7.853574 -3.841777 0.871919
H -7.229947 -4.515728 0.264694
H -8.021552 -4.338261 1.840358
H -8.832480 -3.753310 0.376076

216

[(^{DIPeP}BDI)Sr]$_2$(biphenyl)

Sr -3.229369 0.161229 0.127254
N -4.923708 -1.426045 -0.916149
N -4.942061 1.620125 -0.993763
C -6.613666 -2.339396 -2.443050
H -7.296633 -2.005718 -3.233197
H -7.205401 -2.801964 -1.639869
H -5.972218 -3.134503 -2.840467
C -5.792820 -1.192643 -1.896916
C -6.069257 0.068081 -2.467462
H -6.760467 0.042638 -3.309904
C -5.751877 1.362903 -2.011033
C -6.413427 2.500460 -2.760187
H -6.543344 3.387096 -2.126126
H -7.388505 2.200116 -3.165117
H -5.779611 2.797806 -3.611524
C -4.910846 -2.722392 -0.344362
C -5.624146 -2.940125 0.860882
C -5.660599 -4.232839 1.395241
H -6.231384 -4.421031 2.306347
C -4.985621 -5.285374 0.785712
H -5.032696 -6.289526 1.213563
C -4.234904 -5.047930 -0.362395
H -3.687496 -5.873158 -0.821759
C -4.172925 -3.776519 -0.940776
C -6.357257 -1.794410 1.538913
H -5.753355 -0.885478 1.362538
C -7.725925 -1.499075 0.895168
H -8.186886 -0.669984 1.457924
H -7.565720 -1.108319 -0.120328
C -8.685569 -2.679604 0.852256
H -8.259685 -3.525124 0.289841
H -9.632917 -2.397443 0.367891
H -8.930140 -3.050223 1.860071
C -6.486569 -1.945800 3.059056
H -7.025032 -1.056526 3.427962
H -7.139467 -2.800348 3.304212
C -5.164494 -2.071055 3.799672
H -4.618726 -2.981980 3.513655
H -5.316328 -2.096584 4.889492
H -4.513202 -1.215456 3.571766
C -3.275755 -3.512980 -2.137101
H -3.679130 -2.635607 -2.666013
C -1.863178 -3.112148 -1.661266
H -1.278735 -2.769667 -2.531458

H -1.943792 -2.232331 -0.998527
C -1.097449 -4.201349 -0.924241
H -0.075924 -3.876201 -0.683006
H -0.996784 -5.112167 -1.533025
H -1.595520 -4.478281 0.016380
C -3.198778 -4.663069 -3.149870
H -2.460682 -4.377117 -3.919016
H -2.779575 -5.562147 -2.670189
C -4.513078 -5.027808 -3.823002
H -4.381285 -5.873301 -4.515653
H -4.913986 -4.184705 -4.407008
H -5.276008 -5.319213 -3.084535
C -4.846324 2.917628 -0.448340
C -5.589977 3.205499 0.724567
C -5.428719 4.455100 1.329963
H -5.998110 4.692884 2.230568
C -4.571236 5.411715 0.793069
H -4.465435 6.386135 1.275809
C -3.864502 5.129228 -0.372612
H -3.221680 5.897364 -0.806212
C -3.978114 3.887200 -1.007390
C -3.217005 3.587367 -2.282595
H -3.640676 2.658556 -2.691146
C -1.732473 3.270606 -2.020370
H -1.675663 2.486617 -1.245460
H -1.301938 2.808830 -2.923592
C -0.866062 4.445663 -1.594138
H 0.191017 4.152055 -1.528099
H -1.163412 4.838223 -0.610881
H -0.920951 5.274674 -2.316378
C -3.433091 4.673077 -3.346649
H -4.517870 4.844979 -3.440078
H -3.015888 5.634211 -3.003598
C -2.850740 4.323446 -4.709051
H -1.752718 4.249477 -4.682356
H -3.109635 5.085403 -5.460116
H -3.235431 3.355189 -5.069663
C -0.434192 0.196412 0.715451
C -0.915431 1.457361 1.285242
H -0.521873 2.398380 0.905289
C -1.847171 1.499316 2.299273
H -2.169066 2.475930 2.673329
C -2.435033 0.315662 2.838919
H -3.080022 0.367603 3.714011
C -1.867516 -0.922365 2.416506
H -2.206703 -1.852339 2.882485
C -0.947686 -0.996465 1.393060
H -0.587001 -1.977427 1.091360
C -6.606291 2.201189 1.236974
H -6.207782 1.199156 1.002571
C -6.843946 2.252527 2.748403
H -7.276265 3.224582 3.040500
H -7.624041 1.508390 2.983292
C -5.605144 1.958812 3.577369
H -5.182279 0.980453 3.306009
H -5.830812 1.940227 4.654515
H -4.816259 2.707132 3.410131
C -7.934443 2.301863 0.456881
H -7.766955 1.950929 -0.571526
H -8.638442 1.578028 0.901011
C -8.571952 3.683401 0.427433
H -7.918175 4.418374 -0.066556
H -8.783716 4.062390 1.439791
H -9.526605 3.661910 -0.120471
Sr 3.314762 0.070683 -0.071227
N 5.250351 1.278036 1.006262
N 4.921714 -1.668642 0.752416
C 7.121036 1.890297 2.466644
H 7.869021 1.419282 3.115773
H 7.641910 2.461011 1.684332
H 6.556331 2.627179 3.052921
C 6.188159 0.869078 1.856692
C 6.408712 -0.470820 2.227654
H 7.191639 -0.626081 2.970749
C 5.873630 -1.650503 1.669788
C 6.484309 -2.940170 2.175758
H 6.201491 -3.799361 1.555473
H 7.580523 -2.865788 2.203355
H 6.147533 -3.135606 3.205785
C 5.314833 2.603607 0.523795
C 6.007834 2.839927 -0.690600
C 6.105443 4.152937 -1.162176
H 6.654733 4.352930 -2.084474
C 5.512210 5.209532 -0.477512
H 5.606176 6.230283 -0.855433
C 4.780940 4.957843 0.680740
H 4.299115 5.789650 1.198283
C 4.656721 3.663370 1.194832
C 6.631248 1.681200 -1.449970
H 6.021948 0.791178 -1.211220
C 8.048246 1.325546 -0.958564
H 8.419351 0.497802 -1.586996
H 7.976004 0.908688 0.056669
C 9.049229 2.471657 -0.976545
H 8.725468 3.299524 -0.326705
H 10.036507 2.135873 -0.623924
H 9.186017 2.885594 -1.988039
C 6.592202 1.850168 -2.973011
H 7.026325 0.935876 -3.412685
H 7.259712 2.669732 -3.288906
C 5.197392 2.066537 -3.538364
H 4.761974 3.018341 -3.200547
H 5.203330 2.069680 -4.638966
H 4.517617 1.264423 -3.211629
C 3.784435 3.379300 2.403922
H 4.147182 2.441276 2.853007
C 2.327377 3.111942 1.967788
H 1.750322 2.773471 2.844726
H 2.304411 2.261414 1.261807
C 1.621060 4.286033 1.308148
H 0.576425 4.037309 1.080570
H 1.602455 5.170597 1.962098
H 2.109940 4.573466 0.365853
C 3.836512 4.458438 3.492693
H 3.111539 4.178393 4.276236
H 3.472516 5.418668 3.092736

C 5.206368 4.666039 4.120303
H 5.181326 5.469149 4.872723
H 5.557645 3.752951 4.626318
H 5.957080 4.940876 3.363317
C 4.430415 -2.853806 0.174773
C 4.797376 -3.160158 -1.164431
C 4.170182 -4.239096 -1.793600
H 4.441818 -4.497567 -2.818006
C 3.211960 -5.006751 -1.135264
H 2.731548 -5.842052 -1.649980
C 2.891918 -4.725854 0.189478
H 2.177174 -5.363290 0.712704
C 3.488624 -3.658409 0.869527
C 3.190184 -3.403154 2.334633
H 3.868345 -2.602071 2.657633
C 1.776388 -2.852660 2.609901
H 1.575229 -2.023201 1.910371
H 1.782355 -2.386410 3.608990
C 0.630689 -3.851724 2.545547
H -0.309954 -3.377845 2.857764
H 0.478827 -4.244716 1.531354
H 0.798778 -4.709527 3.214279
C 3.533553 -4.637181 3.184708
H 4.543245 -4.976360 2.904652
H 2.864672 -5.473651 2.922658
C 3.478807 -4.385672 4.685030
H 2.460980 -4.144138 5.027628
H 3.814010 -5.269363 5.249394
H 4.128103 -3.541451 4.970327
C 0.414532 0.130983 -0.402736
C 0.883468 -1.133551 -0.972409
H 0.560906 -2.070207 -0.525071
C 1.708221 -1.188469 -2.074097
H 2.010799 -2.172238 -2.444565
C 2.235458 -0.011443 -2.682889
H 2.794965 -0.065259 -3.613511
C 1.748061 1.235206 -2.191614
H 2.073248 2.163863 -2.671161
C 0.925998 1.318589 -1.088389
H 0.627176 2.303736 -0.738085
C 5.909723 -2.372689 -1.843653
H 5.723042 -1.301569 -1.640610
C 5.989522 -2.542065 -3.364859
H 6.128510 -3.606615 -3.617190
H 6.917581 -2.047185 -3.697243
C 4.823772 -1.966172 -4.150804
H 4.745597 -0.882605 -3.985210
H 4.951499 -2.131026 -5.231678
H 3.863972 -2.412950 -3.855167
C 7.283082 -2.678372 -1.207594
H 7.299483 -2.282287 -0.184415
H 8.042884 -2.096712 -1.756833
C 7.669889 -4.150254 -1.197778
H 6.930512 -4.754493 -0.649721
H 7.742258 -4.565969 -2.214942
H 8.647712 -4.296032 -0.713605

206

[(^{DIPeP}BDI)Ca]$_2$(C$_6$H$_6$) singlet
N -3.608388 1.855483 -0.630267
N 4.089402 1.160405 0.367607
C -4.924761 1.929434 -0.527358
C -5.634827 3.235798 -0.801454
C -2.820346 2.948760 -1.045294
C -2.268128 2.911832 -2.354960
C -1.405252 3.934799 -2.751132
H -0.982285 3.928879 -3.756105
C -1.049418 4.957610 -1.872691
H -0.360668 5.741561 -2.195458
C -1.562946 4.963853 -0.582499
H -1.268880 5.757205 0.109287
C -2.452955 3.973181 -0.146908
C -2.600737 1.744582 -3.268874
H -2.522286 0.827036 -2.652640
C -1.587913 1.569338 -4.402783
H -1.662720 2.426254 -5.094486
H -0.576270 1.613097 -3.972816
C -4.062919 1.790661 -3.777297
H -4.060386 2.148639 -4.821943
C -4.801799 0.463697 -3.670902
C -2.970143 4.025695 1.278125
H -3.693144 3.204702 1.404400
C -1.800941 3.758750 2.245957
H -1.459563 2.724941 2.070333
H -0.951917 4.400667 1.956314
C -3.718850 5.351976 1.536955
H -4.129606 5.707568 0.578304
C -4.859438 5.260378 2.544120
C 5.389810 0.932426 0.471210
C 6.346150 2.099335 0.570565
C 3.559379 2.462631 0.291059
C 2.874880 3.000268 1.409624
C 2.243482 4.241085 1.271690
H 1.718021 4.675079 2.123340
C 2.265135 4.934447 0.065341
H 1.759887 5.898698 -0.021150
C 2.925776 4.390294 -1.032810
H 2.933573 4.938124 -1.976948
C 3.573537 3.155022 -0.946783
C 2.827028 2.228072 2.716077
H 2.714411 1.164088 2.444053
C 1.611560 2.593198 3.576447
H 1.723915 3.611504 3.984669
H 0.726424 2.632439 2.923863
C 4.157215 2.319574 3.488294
C 4.589473 3.733139 3.849754
H 4.075293 1.708224 4.402215
C 4.232052 2.525376 -2.158275
H 4.936404 1.767573 -1.788340
C 3.215588 1.738988 -3.009827
H 2.660281 1.049737 -2.349296
C 2.205124 2.570583 -3.780349
C 5.059786 3.526608 -2.973261
H 5.728235 4.059246 -2.276083
C 5.883317 2.881489 -4.079696
Ca -2.114786 0.085976 -0.238599

Ca 2.253101 -0.216398 -0.094419
N -4.185664 -0.987078 0.115511
N 4.023716 -1.723837 0.083802
C -5.754241 0.842015 -0.171082
H -6.820640 1.067845 -0.144561
C -5.421543 -0.496749 0.113457
C -6.567935 -1.432433 0.409022
C -3.939998 -2.330854 0.483285
C -3.745155 -2.653589 1.850743
C -3.318611 -3.939938 2.188344
H -3.146593 -4.190090 3.237681
C -3.116251 -4.911794 1.211526
H -2.783657 -5.913833 1.492736
C -3.361150 -4.604160 -0.123022
H -3.236742 -5.379777 -0.881296
C -3.774619 -3.324630 -0.511820
C -3.932558 -1.591680 2.916891
H -4.611000 -0.827365 2.506059
C -2.593103 -0.891165 3.186828
H -1.944040 -1.572131 3.763166
H -2.060047 -0.753088 2.233456
C -4.561560 -2.118648 4.211265
H -3.887198 -2.860153 4.673895
H -4.613409 -1.286791 4.932343
C -4.030272 -3.001247 -1.970900
H -4.663936 -2.101436 -1.988709
C -2.739889 -2.609370 -2.712044
H -2.302888 -1.715867 -2.231742
C -1.661803 -3.677083 -2.814448
C -4.792766 -4.102929 -2.717144
C -6.174964 -4.392464 -2.151266
H -4.883400 -3.792873 -3.772655
C 5.964426 -0.358060 0.476065
H 7.044096 -0.395818 0.623346
C 5.317384 -1.601600 0.339612
C 6.147719 -2.849405 0.508580
C 3.326427 -2.945393 0.118980
C 2.959368 -3.581528 -1.091460
C 2.006700 -4.605936 -1.049821
H 1.689576 -5.080422 -1.981886
C 1.468828 -5.042865 0.156582
H 0.720226 -5.838216 0.169717
C 1.912534 -4.479851 1.352934
H 1.525848 -4.865035 2.298469
C 2.835250 -3.430828 1.361871
C 3.587635 -3.167808 -2.409371
H 4.448913 -2.523160 -2.166378
C 2.614513 -2.318495 -3.238749
H 1.712771 -2.916242 -3.460715
H 2.248370 -1.492118 -2.607380
C 4.112180 -4.374399 -3.201754
H 3.259423 -5.001519 -3.516551
H 4.566760 -4.007552 -4.135866
C 3.312709 -2.805728 2.658767
H 4.203532 -2.210567 2.415089
C 2.314329 -1.788160 3.241704
H 2.021544 -1.081093 2.446854
C 1.056341 -2.360659 3.873718
C 3.761386 -3.865272 3.674583
H 4.452745 -4.555672 3.162557
C 4.441386 -3.281624 4.905566
H -2.999428 6.128702 1.851618
H -4.619831 2.551591 -3.213343
C -1.729934 0.277176 -5.192638
H -4.201105 -5.033225 -2.736516
H -3.021580 -2.276704 -3.724694
C -5.949952 -2.717064 4.038773
C -2.707745 0.458121 3.873594
C 5.129323 -5.224930 -2.455558
C 3.186689 -1.736944 -4.522953
H 2.905804 -4.487241 3.986039
C -2.075007 3.950798 3.729932
H 2.844934 -1.169196 3.982968
C 1.341365 1.624210 4.717703
H 4.938835 1.833721 2.886962
H 4.410468 4.304500 -3.407654
H 3.767213 1.088687 -3.705808
H -7.535557 -0.916737 0.384331
H -6.582941 -2.253110 -0.322798
H -6.441447 -1.905868 1.392604
H -6.607176 3.060543 -1.281173
H -5.825101 3.766905 0.144288
H -5.032016 3.901643 -1.433065
H 7.290877 1.802669 1.044330
H 6.581982 2.480187 -0.436662
H 5.905782 2.935818 1.129932
H 7.219897 -2.624027 0.567025
H 5.855024 -3.380495 1.428156
H 5.968624 -3.547702 -0.320700
C 0.166101 1.318961 0.197468
C 0.032625 0.295984 1.192722
C -0.032480 -1.140428 0.719118
C -0.005013 -1.413110 -0.639586
C 0.190254 1.037961 -1.153925
C 0.063624 -0.385264 -1.626618
H 0.054589 0.532233 2.252845
H -0.093853 -1.958278 1.432344
H -0.032787 -2.457900 -0.956293
H 0.261691 1.852402 -1.869320
H 0.091780 -0.620736 -2.687554
H 0.255310 2.362229 0.507832
H 1.523094 1.920725 -4.347762
H 1.592598 3.190526 -3.109258
H 2.695132 3.239641 -4.504466
H 6.534414 3.618881 -4.573581
H 6.527698 2.081096 -3.680442
H 5.247864 2.433185 -4.859046
H -5.836595 0.551012 -4.036298
H -4.309104 -0.330053 -4.250444
H -4.849585 0.136193 -2.621471
H -0.929191 0.188259 -5.942960
H -1.666190 -0.599134 -4.530100
H -2.688451 0.221204 -5.730218
H -6.677005 -5.198892 -2.707625
H -6.117915 -4.698100 -1.095068
H -6.820156 -3.501161 -2.207217

H -0.773305 -3.279561 -3.328328
H -1.340719 -4.028670 -1.822918
H -2.004731 -4.554193 -3.384823
H 5.444439 -6.091011 -3.057605
H 6.034534 -4.643042 -2.219538
H 4.719124 -5.604794 -1.507366
H 2.485095 -1.017161 -4.972174
H 4.131516 -1.203924 -4.328890
H 3.391470 -2.509301 -5.279506
H 0.442170 1.916850 5.280876
H 1.178485 0.604442 4.338588
H 2.174745 1.579980 5.435062
H 5.554947 3.728128 4.378632
H 4.701547 4.359029 2.950467
H 3.859676 4.232861 4.506281
H 4.831159 -4.076178 5.560106
H 5.288609 -2.636017 4.621790
H 3.751156 -2.670909 5.507868
H 0.390807 -1.553148 4.213168
H 0.484332 -2.980782 3.168441
H 1.287829 -2.983071 4.751750
H -6.343557 -3.099353 4.993183
H -6.662444 -1.963771 3.666758
H -5.941363 -3.551772 3.320948
H -1.719486 0.922688 4.000754
H -3.321676 1.144888 3.269791
H -3.169530 0.385005 4.870125
H -5.381867 6.225249 2.638523
H -5.603754 4.511367 2.228359
H -4.517231 4.969807 3.546281
H -2.290824 5.001647 3.975366
H -2.923963 3.343358 4.076561
H -1.195352 3.654602 4.321149

206

[(^{DIPeP}BDI)Ca]$_2$(C_6H_6) triplet

N -3.571853 1.885180 -0.557232
N 4.087683 1.131023 0.365930
C -4.886355 1.986881 -0.452141
C -5.570488 3.312847 -0.698469
C -2.766020 2.977546 -0.942498
C -2.239763 2.988532 -2.261950
C -1.367063 4.014717 -2.629424
H -0.963203 4.046185 -3.641711
C -0.977323 4.991346 -1.714379
H -0.279197 5.776021 -2.014483
C -1.471677 4.953018 -0.416586
H -1.155169 5.713526 0.301500
C -2.372064 3.961059 -0.008919
C -2.614490 1.873579 -3.223206
H -2.593887 0.937379 -2.634019
C -1.590105 1.695311 -4.346912
H -1.617893 2.577389 -5.009640
H -0.584452 1.684781 -3.899021
C -4.064919 2.009637 -3.749142
H -4.032233 2.404571 -4.779948
C -4.868581 0.716824 -3.699634
C -2.892472 3.979579 1.416596
H -3.618744 3.158010 1.519184
C -1.732024 3.689796 2.387003
H -1.381246 2.666506 2.178017
H -0.883130 4.344353 2.126848
C -3.641252 5.300778 1.701560
H -4.046279 5.678918 0.749258
C -4.787317 5.188558 2.700376
C 5.387854 0.895023 0.464115
C 6.342087 2.052340 0.657577
C 3.568075 2.440355 0.415620
C 2.892531 2.871182 1.585314
C 2.286738 4.131736 1.579732
H 1.770297 4.485664 2.472708
C 2.318501 4.944982 0.450558
H 1.832526 5.923010 0.466043
C 2.964057 4.503626 -0.700434
H 2.978315 5.144259 -1.584030
C 3.591305 3.254609 -0.744033
C 2.822339 1.964655 2.802370
H 2.672423 0.938502 2.421001
C 1.623367 2.278053 3.705210
H 1.759034 3.258590 4.190838
H 0.732053 2.385972 3.068809
C 4.155555 1.922400 3.573274
C 4.621901 3.267411 4.110639
H 4.059971 1.200814 4.401317
C 4.241230 2.748455 -2.016695
H 4.943829 1.953487 -1.729884
C 3.216878 2.058556 -2.938469
H 2.700243 1.273652 -2.357796
C 2.165928 2.961027 -3.561058
C 5.069011 3.826011 -2.728718
H 5.736641 4.288930 -1.982570
C 5.893838 3.295519 -3.893522
Ca -2.115992 0.073915 -0.175373
Ca 2.251236 -0.241692 -0.148226
N -4.211560 -0.949500 0.145850
N 4.024640 -1.751561 -0.019479
C -5.741867 0.912690 -0.116991
H -6.802880 1.162652 -0.087459
C -5.438993 -0.437156 0.141126
C -6.604974 -1.356763 0.408396
C -3.999212 -2.314649 0.448975
C -3.803064 -2.708030 1.797221
C -3.410054 -4.020941 2.067785
H -3.237635 -4.327701 3.101959
C -3.241566 -4.947702 1.042021
H -2.933614 -5.970790 1.270567
C -3.487228 -4.568813 -0.274342
H -3.387814 -5.309393 -1.070440
C -3.869050 -3.261494 -0.596197
C -3.960730 -1.692790 2.912741
H -4.619048 -0.894447 2.535516
C -2.605698 -1.035478 3.220087
H -2.009037 -1.718805 3.848976
H -2.025774 -0.955921 2.287706
C -4.604428 -2.259973 4.182301
H -3.958178 -3.050918 4.600790

H -4.617439 -1.464400 4.945572
C -4.134594 -2.857058 -2.034070
H -4.755694 -1.949060 -1.997511
C -2.847929 -2.442929 -2.769664
H -2.382720 -1.599598 -2.230333
C -1.797316 -3.525299 -2.959991
C -4.920073 -3.906310 -2.830274
C -6.303230 -4.202946 -2.270325
H -5.012578 -3.540858 -3.867758
C 5.967033 -0.390975 0.384833
H 7.048963 -0.430573 0.513201
C 5.325521 -1.630394 0.196425
C 6.172028 -2.877446 0.262089
C 3.351923 -2.987281 -0.057834
C 2.977075 -3.539998 -1.306092
C 2.062634 -4.598013 -1.326589
H 1.741942 -5.011894 -2.285870
C 1.563480 -5.140906 -0.146720
H 0.843961 -5.962166 -0.180197
C 2.008591 -4.650841 1.080233
H 1.652227 -5.117351 2.000700
C 2.900310 -3.576902 1.153230
C 3.553356 -2.990134 -2.598196
H 4.422429 -2.368733 -2.324569
C 2.545614 -2.062527 -3.290865
H 1.629638 -2.633030 -3.524104
H 2.212192 -1.302523 -2.565373
C 4.051198 -4.099975 -3.534516
H 3.191822 -4.699537 -3.883019
H 4.467995 -3.631285 -4.440224
C 3.390865 -3.044997 2.486839
H 4.264666 -2.413171 2.273869
C 2.383996 -2.098173 3.165771
H 2.091211 -1.326678 2.434941
C 1.127034 -2.738758 3.731892
C 3.880242 -4.172980 3.405913
H 4.573453 -4.806666 2.827247
C 4.574001 -3.677477 4.667456
H -2.921635 6.068582 2.037160
H -4.591917 2.775712 -3.163398
C -1.770167 0.439447 -5.185943
H -4.343921 -4.843621 -2.901899
H -3.138490 -2.034653 -3.751314
C -6.016098 -2.793407 3.988663
C -2.704941 0.339049 3.858848
C 5.102558 -5.011712 -2.919133
C 3.055441 -1.350299 -4.534404
H 3.042773 -4.834682 3.682860
C -2.022152 3.828915 3.873767
H 2.907840 -1.549741 3.965323
C 1.348817 1.230394 4.773941
H 4.923509 1.498068 2.911480
H 4.418618 4.641612 -3.085290
H 3.762007 1.521148 -3.729955
H -7.562558 -0.822652 0.383285
H -6.628803 -2.164896 -0.336843
H -6.496815 -1.848701 1.385182
H -6.518778 3.168027 -1.233905
H -5.807131 3.798171 0.261229
H -4.933805 4.002343 -1.267601
H 7.297072 1.716973 1.081938
H 6.554470 2.534668 -0.310385
H 5.908316 2.826240 1.305569
H 7.243363 -2.644354 0.302186
H 5.912010 -3.469143 1.153951
H 5.974202 -3.524025 -0.604557
C 0.162759 1.254048 0.352430
C 0.081580 0.113786 1.228865
C -0.010308 -1.214091 0.671262
C -0.030800 -1.397115 -0.752324
C 0.171467 1.066628 -1.067871
C 0.051881 -0.253476 -1.625159
H 0.087854 0.253812 2.308784
H -0.075547 -2.085444 1.319532
H -0.062945 -2.405716 -1.158891
H 0.217943 1.939176 -1.714942
H 0.032605 -0.387884 -2.706617
H 0.268112 2.260354 0.751745
H 1.481484 2.378649 -4.195285
H 1.558562 3.464428 -2.794893
H 2.618004 3.737634 -4.197675
H 6.543674 4.079691 -4.311221
H 6.539864 2.460489 -3.576113
H 5.259156 2.926370 -4.713922
H -5.890493 0.865643 -4.081462
H -4.403289 -0.080665 -4.296885
H -4.952776 0.356989 -2.662788
H -0.966911 0.349928 -5.933484
H -1.743875 -0.464200 -4.558439
H -2.725718 0.437943 -5.731690
H -6.820796 -4.972334 -2.863648
H -6.244745 -4.563228 -1.231573
H -6.935017 -3.300438 -2.276597
H -0.909190 -3.117075 -3.466189
H -1.466570 -3.942390 -1.997614
H -2.172580 -4.356281 -3.576988
H 5.409417 -5.800467 -3.623049
H 6.006710 -4.444036 -2.646982
H 4.728069 -5.500545 -2.006877
H 2.333440 -0.591566 -4.873331
H 4.008565 -0.835978 -4.332265
H 3.221586 -2.042453 -5.373307
H 0.448778 1.485786 5.353696
H 1.186204 0.238349 4.326771
H 2.179924 1.135066 5.488814
H 5.595356 3.174917 4.616232
H 4.732255 4.004564 3.299786
H 3.913024 3.688835 4.840868
H 4.994574 -4.514430 5.245707
H 5.401138 -2.991295 4.422156
H 3.883915 -3.135194 5.332096
H 0.457012 -1.974116 4.153275
H 0.559265 -3.281695 2.962389
H 1.357481 -3.450433 4.539836
H -6.419748 -3.210812 4.923949
H -6.700549 -1.994494 3.661883

H -6.042927 -3.588454 3.227477
H -1.709313 0.769171 4.040244
H -3.251817 1.031948 3.199565
H -3.234305 0.314486 4.823856
H -5.312107 6.150622 2.809363
H -5.528167 4.443933 2.366679
H -4.450634 4.880413 3.699146
H -2.242444 4.869860 4.154784
H -2.874158 3.208538 4.189198
H -1.148190 3.512635 4.463538

206

[(^{DIPeP}BDI)Sr]$_2$(C$_6$H$_6$) singlet

N -3.929757 1.783689 -0.384562
N 4.163923 1.395844 0.213856
C -5.231834 1.922913 -0.210362
C -5.874747 3.283014 -0.367688
C -3.098305 2.859261 -0.744700
C -2.603447 2.914169 -2.076297
C -1.709396 3.928747 -2.421673
H -1.335562 3.995231 -3.443927
C -1.262331 4.849190 -1.474924
H -0.550938 5.627018 -1.760472
C -1.712817 4.758699 -0.163802
H -1.345161 5.467663 0.581942
C -2.633266 3.777764 0.223158
C -3.036301 1.852220 -3.071135
H -3.032143 0.891383 -2.521952
C -2.050315 1.688974 -4.231284
H -2.080250 2.591003 -4.866666
H -1.031614 1.640857 -3.816931
C -4.496261 2.058664 -3.548187
H -4.477425 2.488377 -4.565352
C -5.350643 0.798543 -3.518409
C -3.076011 3.707204 1.671501
H -3.866439 2.943999 1.744293
C -1.893816 3.223163 2.532187
H -1.613500 2.211501 2.187730
H -1.016293 3.853466 2.309692
C -3.682096 5.050944 2.133206
H -4.084868 5.569035 1.247991
C -4.796018 4.929905 3.166842
C 5.486288 1.374072 0.282599
C 6.242727 2.676688 0.425083
C 3.455515 2.610527 0.281992
C 2.793378 2.960990 1.486118
C 2.040466 4.139614 1.514881
H 1.538383 4.434837 2.437112
C 1.915523 4.947923 0.389009
H 1.326972 5.866401 0.436268
C 2.535227 4.575114 -0.799828
H 2.423008 5.207980 -1.682053
C 3.301171 3.408758 -0.880590
C 2.893645 2.057849 2.703384
H 2.871949 1.022925 2.320444
C 1.692511 2.206817 3.646113
H 1.716490 3.191019 4.142841
H 0.774545 2.204648 3.038638
C 4.244573 2.204377 3.429953
C 4.544185 3.605001 3.943831
H 4.272844 1.486060 4.266033
C 3.929371 2.974043 -2.190373
H 4.722563 2.253195 -1.944281
C 2.930525 2.192741 -3.069340
H 2.482272 1.383080 -2.466183
C 1.788782 2.998330 -3.665246
C 4.606935 4.130665 -2.936644
H 5.265268 4.653776 -2.223026
C 5.415636 3.689032 -4.148776
Sr -2.315576 -0.107607 -0.057644
Sr 2.443707 -0.309983 -0.423896
N -4.617932 -1.045859 0.294076
N 4.609827 -1.515061 -0.143160
C -6.104051 0.863821 0.128472
H -7.154725 1.142220 0.215229
C -5.827846 -0.501563 0.342040
C -7.010800 -1.393153 0.633765
C -4.438492 -2.428941 0.500258
C -4.071102 -2.907884 1.783938
C -3.782832 -4.265906 1.947521
H -3.496336 -4.636765 2.934832
C -3.857089 -5.152705 0.878268
H -3.634074 -6.212271 1.023680
C -4.213043 -4.677740 -0.381257
H -4.265719 -5.376335 -1.218679
C -4.501254 -3.326869 -0.597137
C -3.914501 -1.954477 2.952519
H -4.394770 -1.004139 2.666043
C -2.419273 -1.667324 3.181859
H -1.935920 -2.584542 3.562222
H -1.921950 -1.494468 2.211204
C -4.588697 -2.448906 4.238664
H -4.084007 -3.367733 4.585585
H -4.414734 -1.703225 5.031126
C -4.809516 -2.813206 -1.990441
H -5.392388 -1.886838 -1.876307
C -3.522074 -2.402526 -2.731718
H -3.064032 -1.540171 -2.214937
C -2.464225 -3.480352 -2.907606
C -5.654812 -3.770797 -2.838232
C -7.026861 -4.071699 -2.253276
H -5.773230 -3.315960 -3.836669
C 6.270549 0.200512 0.220933
H 7.344018 0.351847 0.336160
C 5.860399 -1.140920 0.086929
C 6.927672 -2.199972 0.233163
C 4.220500 -2.864969 -0.092182
C 3.917073 -3.559183 -1.289455
C 3.403191 -4.858128 -1.210119
H 3.166372 -5.396746 -2.131447
C 3.200361 -5.478951 0.017410
H 2.806873 -6.497143 0.061795
C 3.499051 -4.791481 1.193473
H 3.335230 -5.283681 2.154134
C 3.994541 -3.486091 1.166907
C 4.101058 -2.894429 -2.640058

H 4.647398 -1.952674 -2.460092
C 2.731937 -2.533570 -3.244587
H 2.202647 -3.463516 -3.517359
H 2.089442 -2.082494 -2.467052
C 4.939655 -3.736078 -3.611258
H 4.394830 -4.666597 -3.849850
H 5.024583 -3.190498 -4.565157
C 4.230257 -2.707158 2.445501
H 4.947111 -1.906439 2.214901
C 2.950471 -1.974510 2.894806
H 2.580483 -1.359115 2.057404
C 1.811298 -2.849920 3.389376
C 4.862504 -3.550449 3.559251
H 5.739631 -4.069715 3.137463
C 5.287264 -2.733967 4.772572
H -2.881118 5.707480 2.517209
H -4.975694 2.822932 -2.920580
C -2.286516 0.460301 -5.096197
H -5.111000 -4.714321 -3.012114
H -3.813395 -2.006021 -3.717825
C -6.083013 -2.700124 4.109968
C -2.125992 -0.492148 4.099311
C 6.332056 -4.071489 -3.100159
C 2.780509 -1.587628 -4.435632
H 4.174367 -4.349636 3.880941
C -2.115634 3.190476 4.036619
H 3.223527 -1.244092 3.672053
C 1.587241 1.119829 4.705268
H 5.041803 1.878163 2.747145
H 3.860524 4.879654 -3.248323
H 3.488776 1.687228 -3.873754
H -7.948648 -0.826922 0.687121
H -7.108369 -2.165529 -0.142939
H -6.862604 -1.930702 1.580400
H -6.895775 3.194200 -0.762264
H -5.940636 3.783726 0.611274
H -5.285964 3.937084 -1.024646
H 7.272638 2.509776 0.764833
H 6.285578 3.196181 -0.545855
H 5.737800 3.359843 1.121894
H 7.935051 -1.765304 0.225357
H 6.797590 -2.747049 1.180443
H 6.848156 -2.946029 -0.569044
C 0.168161 1.096345 0.224111
C 0.174934 -0.023765 1.130173
C 0.093074 -1.403241 0.528564
C -0.042611 -1.545303 -0.843542
C 0.040020 0.943762 -1.140439
C -0.115682 -0.426266 -1.735300
H 0.320444 0.113050 2.197917
H 0.136853 -2.286075 1.165540
H -0.104771 -2.556427 -1.256877
H 0.005893 1.826898 -1.774788
H -0.183387 -0.564536 -2.812806
H 0.271932 2.109095 0.621126
H 1.120162 2.347669 -4.247841
H 1.184408 3.477389 -2.881743
H 2.153760 3.785886 -4.342327
H 5.963244 4.534890 -4.591723
H 6.155454 2.919396 -3.873945
H 4.777176 3.264679 -4.939101
H -6.378530 1.004948 -3.854331
H -4.941989 0.008424 -4.164734
H -5.410906 0.400850 -2.493924
H -1.515769 0.376979 -5.877939
H -2.249110 -0.458116 -4.490921
H -3.263369 0.489588 -5.602038
H -7.591848 -4.768651 -2.891178
H -6.946313 -4.523007 -1.252349
H -7.625503 -3.151901 -2.157198
H -1.588596 -3.076074 -3.438054
H -2.120049 -3.868471 -1.937616
H -2.840358 -4.333031 -3.493657
H 6.895291 -4.667989 -3.834051
H 6.911360 -3.156118 -2.900390
H 6.284072 -4.648960 -2.164102
H 1.766450 -1.289593 -4.741972
H 3.333477 -0.667602 -4.187285
H 3.271281 -2.042174 -5.308901
H 0.695711 1.265788 5.333746
H 1.508448 0.124267 4.243506
H 2.459322 1.103787 5.376377
H 5.534887 3.647334 4.421936
H 4.534240 4.341929 3.125276
H 3.806275 3.937680 4.690992
H 5.826767 -3.356103 5.502956
H 5.953650 -1.906082 4.480199
H 4.424458 -2.291957 5.294449
H 0.927814 -2.239088 3.629637
H 1.508744 -3.585065 2.628949
H 2.085187 -3.402322 4.301540
H -6.511759 -3.055865 5.059408
H -6.616919 -1.778214 3.830289
H -6.296590 -3.458454 3.340892
H -1.047170 -0.290208 4.152545
H -2.611323 0.422465 3.727982
H -2.481863 -0.665146 5.126016
H -5.213976 5.917956 3.415589
H -5.621759 4.308846 2.783458
H -4.455720 4.468957 4.103830
H -2.245673 4.200090 4.454429
H -3.004143 2.599666 4.308130
H -1.250506 2.736678 4.542568

206

[(^{DIPeP}BDI)Sr]$_2$(C_6H_6) triplet

N -3.894565 1.782232 -0.381931
N 4.159971 1.390733 0.230388
C -5.198320 1.935334 -0.234129
C -5.830111 3.298638 -0.411368
C -3.050819 2.851769 -0.737144
C -2.558583 2.910758 -2.069072
C -1.668690 3.929795 -2.413795
H -1.297140 4.000204 -3.436552
C -1.225670 4.851507 -1.466899
H -0.519094 5.633941 -1.752022

C -1.674579 4.757502 -0.155446
H -1.311887 5.469722 0.589461
C -2.586991 3.769819 0.232435
C -2.997925 1.858602 -3.071677
H -3.022026 0.899521 -2.521408
C -1.996473 1.681817 -4.216974
H -2.003346 2.585382 -4.850536
H -0.984817 1.619968 -3.786717
C -4.446368 2.093083 -3.570185
H -4.405856 2.524803 -4.585884
C -5.322502 0.847372 -3.555763
C -3.040844 3.708969 1.678421
H -3.784869 2.900644 1.760651
C -1.842620 3.326585 2.566836
H -1.488469 2.334988 2.237773
H -1.006733 4.010695 2.344225
C -3.737450 5.023714 2.094156
H -4.163149 5.489002 1.190760
C -4.850984 4.860201 3.122239
C 5.482014 1.367722 0.312818
C 6.235349 2.664918 0.513885
C 3.458128 2.606891 0.344922
C 2.787526 2.907622 1.557850
C 2.049722 4.092970 1.637095
H 1.542618 4.350561 2.567678
C 1.942351 4.953465 0.548420
H 1.362538 5.874979 0.633019
C 2.568435 4.629411 -0.650896
H 2.469642 5.303009 -1.504030
C 3.325720 3.460964 -0.778924
C 2.863219 1.944523 2.730441
H 2.822056 0.928336 2.299774
C 1.662989 2.073698 3.677025
H 1.705399 3.034707 4.215744
H 0.746806 2.117852 3.068616
C 4.213692 2.022182 3.468455
C 4.537853 3.386254 4.059759
H 4.223653 1.258513 4.263723
C 3.967180 3.086044 -2.100731
H 4.766924 2.364374 -1.879503
C 2.981852 2.328177 -3.013317
H 2.568039 1.473348 -2.448954
C 1.811132 3.133037 -3.550340
C 4.634360 4.279562 -2.796537
H 5.276923 4.786809 -2.057364
C 5.462576 3.896142 -4.015285
Sr -2.316377 -0.137562 -0.025269
Sr 2.436171 -0.330485 -0.407497
N -4.636047 -1.042997 0.271186
N 4.617584 -1.517527 -0.159618
C -6.090785 0.887793 0.091774
H -7.138598 1.181905 0.161466
C -5.839091 -0.481059 0.304899
C -7.037353 -1.357384 0.579234
C -4.477419 -2.428526 0.476875
C -4.164468 -2.915716 1.772253
C -3.877147 -4.273173 1.938188
H -3.628350 -4.650892 2.932952
C -3.906094 -5.151855 0.859262
H -3.683080 -6.211329 1.005371
C -4.220304 -4.670934 -0.409058
H -4.244582 -5.365340 -1.251303
C -4.504433 -3.319142 -0.626831
C -4.069956 -1.958462 2.943526
H -4.592264 -1.034852 2.645954
C -2.597790 -1.583650 3.206974
H -2.119707 -2.390339 3.790316
H -2.032651 -1.584305 2.257801
C -4.737798 -2.477909 4.222071
H -4.223052 -3.395434 4.556396
H -4.568858 -1.740779 5.024707
C -4.786327 -2.797331 -2.022977
H -5.364117 -1.867476 -1.911210
C -3.488540 -2.391330 -2.747771
H -3.018734 -1.553941 -2.202332
C -2.450114 -3.483769 -2.944560
C -5.628521 -3.745458 -2.884472
C -7.012072 -4.031098 -2.319345
H -5.727031 -3.289734 -3.884756
C 6.271690 0.199957 0.219022
H 7.344697 0.354412 0.334087
C 5.869540 -1.140248 0.055643
C 6.948164 -2.193553 0.154038
C 4.240376 -2.871511 -0.149449
C 3.914474 -3.521173 -1.366293
C 3.413399 -4.826801 -1.326331
H 3.159627 -5.331360 -2.262308
C 3.242715 -5.495592 -0.119220
H 2.857688 -6.517891 -0.105328
C 3.561884 -4.851408 1.075743
H 3.422668 -5.381836 2.019771
C 4.047783 -3.541918 1.089635
C 4.065098 -2.802905 -2.694148
H 4.597883 -1.859033 -2.486306
C 2.681618 -2.446138 -3.269699
H 2.186112 -3.372519 -3.609948
H 2.023545 -2.085014 -2.458425
C 4.904960 -3.591558 -3.707813
H 4.376467 -4.526009 -3.966748
H 4.958534 -3.012738 -4.644391
C 4.314008 -2.815186 2.393700
H 5.020506 -2.001308 2.177652
C 3.044728 -2.107970 2.909003
H 2.661737 -1.452790 2.108661
C 1.913681 -3.010313 3.373306
C 4.981154 -3.702975 3.451348
H 5.844690 -4.203290 2.981240
C 5.443543 -2.937862 4.684178
H -2.983154 5.740764 2.464325
H -4.921072 2.864178 -2.947262
C -2.236340 0.460452 -5.091166
H -5.092843 -4.695102 -3.049438
H -3.768586 -1.963935 -3.724072
C -6.229084 -2.743998 4.088494
C -2.416517 -0.243551 3.901002
C 6.313871 -3.911910 -3.234182

C 2.697126 -1.418347 -4.392310
H 4.303337 -4.515733 3.760739
C -2.080380 3.306865 4.069142
H 3.332707 -1.420502 3.719821
C 1.535171 0.945142 4.689619
H 5.007199 1.717722 2.771719
H 3.879490 5.027607 -3.089593
H 3.545505 1.883492 -3.849776
H -7.970284 -0.781271 0.607334
H -7.125817 -2.136911 -0.191442
H -6.915474 -1.887004 1.534478
H -6.808836 3.214908 -0.903322
H -5.996131 3.766747 0.571614
H -5.188800 3.974531 -0.991865
H 7.265883 2.484955 0.844741
H 6.274998 3.231778 -0.430083
H 5.729027 3.312087 1.243407
H 7.951486 -1.750239 0.128590
H 6.847732 -2.760156 1.093383
H 6.853962 -2.923987 -0.660676
C 0.155764 1.054564 0.310281
C 0.191370 -0.107012 1.162644
C 0.093248 -1.424770 0.585429
C -0.065190 -1.577980 -0.836079
C 0.036468 0.896976 -1.110146
C -0.090404 -0.415272 -1.686576
H 0.297563 0.007887 2.240416
H 0.119176 -2.308386 1.223051
H -0.141227 -2.577309 -1.265019
H -0.007615 1.785400 -1.737051
H -0.199270 -0.529056 -2.766406
H 0.264631 2.056010 0.723843
H 1.155728 2.499079 -4.165737
H 1.200597 3.544334 -2.733967
H 2.144456 3.971478 -4.181427
H 6.000782 4.766585 -4.420632
H 6.212095 3.128678 -3.761385
H 4.839148 3.491528 -4.827610
H -6.340826 1.070791 -3.909546
H -4.916149 0.050511 -4.195498
H -5.407302 0.449884 -2.532711
H -1.456299 0.371235 -5.862970
H -2.220197 -0.463124 -4.492786
H -3.205827 0.504882 -5.609904
H -7.575419 -4.721976 -2.965217
H -6.950289 -4.483385 -1.317502
H -7.601882 -3.104794 -2.231572
H -1.563757 -3.085116 -3.461172
H -2.117151 -3.898376 -1.981962
H -2.840696 -4.314019 -3.552846
H 6.877017 -4.467761 -3.999387
H 6.875544 -2.991160 -3.010417
H 6.296041 -4.524770 -2.319782
H 1.674024 -1.152665 -4.698513
H 3.197138 -0.489555 -4.074677
H 3.224400 -1.785818 -5.285345
H 0.637936 1.075475 5.313725
H 1.453859 -0.031745 4.189258
H 2.398978 0.893905 5.369465
H 5.529858 3.384490 4.536951
H 4.539143 4.167926 3.283812
H 3.806976 3.689030 4.826296
H 6.003771 -3.590144 5.371414
H 6.102255 -2.098756 4.406740
H 4.597455 -2.517769 5.249725
H 1.038292 -2.414224 3.674003
H 1.590331 -3.692610 2.573231
H 2.206020 -3.622502 4.240588
H -6.654509 -3.124566 5.029742
H -6.774462 -1.823552 3.826520
H -6.432126 -3.489153 3.303722
H -1.354503 -0.006608 4.056357
H -2.852238 0.569523 3.299081
H -2.910453 -0.217210 4.884189
H -5.339879 5.824150 3.333496
H -5.626557 4.169034 2.754192
H -4.488683 4.456443 4.077239
H -2.279554 4.313371 4.466773
H -2.930673 2.664626 4.344673
H -1.192366 2.920842 4.592284

Calculations with DIPP-substituents

158

A1

N -4.054372 1.411029 -0.187799
N 4.044268 1.422059 0.157936
C -5.367357 1.255835 -0.135101
C -6.249797 2.475911 -0.223795
C -3.416136 2.661758 -0.276593
C -2.995329 3.155166 -1.535945
C -2.137005 4.259269 -1.572313
H -1.796930 4.640579 -2.538886
C -1.706553 4.878415 -0.403197
H -1.026041 5.730621 -0.453117
C -2.164046 4.417963 0.829993
H -1.836950 4.919163 1.744203
C -3.027353 3.323946 0.917190
C -3.483271 2.524637 -2.827075
H -4.085070 1.646191 -2.546472
C -2.334297 2.034619 -3.708788
H -1.688073 2.866089 -4.032799
H -1.705779 1.314046 -3.166507
C -4.389643 3.489423 -3.598744
H -3.829634 4.376120 -3.936691
H -4.812115 3.000377 -4.490847
C -3.540714 2.839836 2.263095
H -4.391058 2.172171 2.061300
C -2.501669 2.010700 3.020105
H -2.188742 1.110905 2.466545
H -1.589436 2.594571 3.218723
C -4.055224 3.990021 3.130467
H -4.793570 4.600219 2.588506
H -4.535178 3.600468 4.041833
C 5.357204 1.255620 0.158085
C 6.242885 2.463978 0.333147
C 3.422901 2.665985 0.380467
C 3.093888 3.051506 1.704621
C 2.255347 4.151542 1.897617
H 1.981223 4.445165 2.914243
C 1.753771 4.874582 0.818270
H 1.088410 5.723296 0.989330
C 2.120950 4.520204 -0.476607
H 1.743984 5.103847 -1.320686
C 2.964049 3.431750 -0.719589
C 3.609971 2.261137 2.893223
H 4.379993 1.571718 2.517096
C 2.506195 1.401797 3.512299
H 1.674922 2.023488 3.881808
H 2.075245 0.694820 2.785450
C 4.266820 3.159239 3.942989
H 3.537219 3.832034 4.420852
H 4.723103 2.551628 4.740141
C 3.397069 3.100207 -2.136403
H 4.055710 2.220255 -2.071807
C 2.220396 2.726002 -3.039277
H 1.695831 1.835238 -2.664274
H 1.483364 3.542470 -3.104520
C 4.210811 4.248444 -2.741295
H 5.061304 4.521401 -2.098615
H 4.606029 3.968164 -3.730543
Ca -2.203909 0.000530 0.000439
Ca 2.194423 -0.000158 0.001916
N -4.053264 -1.412168 0.185810
N 4.044780 -1.421466 -0.156827
C -5.991305 -0.001392 -0.001928
H -7.081322 -0.001890 -0.002453
C -5.366330 -1.258075 0.131922
C -6.247861 -2.478855 0.220029
C -3.414363 -2.662532 0.275660
C -2.995090 -3.155634 1.535597
C -2.136268 -4.259334 1.573205
H -1.797335 -4.640428 2.540266
C -1.703940 -4.878321 0.404716
H -1.023067 -5.730181 0.455587
C -2.160069 -4.418237 -0.829137
H -1.831562 -4.919425 -1.742841
C -3.023824 -3.324692 -0.917572
C -3.485101 -2.525297 2.826038
H -4.087412 -1.647472 2.544589
C -2.337585 -2.033987 3.708942
H -1.690949 -2.864793 4.033834
H -1.709137 -1.312944 3.167202
C -4.391408 -3.490765 3.596922
H -3.830932 -4.376837 3.935729
H -4.815346 -3.001906 4.488433
C -3.536200 -2.841248 -2.264097
H -4.387705 -2.174774 -2.063274
C -2.497532 -2.010626 -3.019966
H -2.186453 -1.110452 -2.466005
H -1.584298 -2.593217 -3.217727
C -4.048124 -3.992110 -3.132111
H -4.786182 -4.603414 -2.591006
H -4.527642 -3.603156 -4.043962
C 5.979880 0.000684 0.000275
H 7.069838 0.000882 0.000130
C 5.357642 -1.254443 -0.157591
C 6.243705 -2.462425 -0.333426
C 3.423668 -2.665288 -0.380184
C 3.094991 -3.050356 -1.704558
C 2.256551 -4.150367 -1.898141
H 1.982703 -4.443619 -2.914947
C 1.754706 -4.873815 -0.819193
H 1.089402 -5.722476 -0.990732
C 2.121548 -4.519894 0.475907
H 1.744413 -5.103852 1.319691
C 2.964577 -3.431539 0.719467
C 3.611018 -2.259308 -2.892724
H 4.381157 -1.570186 -2.516309
C 2.507163 -1.399460 -3.510930
H 1.675859 -2.020865 -3.880863
H 2.076311 -0.693181 -2.783360
C 4.267553 -3.156720 -3.943255
H 3.537803 -3.829096 -4.421483
H 4.723718 -2.548571 -4.740064
C 3.397524 -3.100614 2.136458
H 4.056365 -2.220784 2.072214
C 2.220907 -2.726503 3.039438

H 1.696608 -1.835313 2.664998
H 1.483539 -3.542717 3.104110
C 4.211044 -4.249244 2.740908
H 5.061489 -4.522072 2.098109
H 4.606301 -3.969444 3.730276
H -3.240884 4.659005 3.450784
H -5.225073 3.845590 -2.977827
H -2.720472 1.541293 -4.614681
H -3.232478 -4.659888 -3.451623
H -2.900219 -1.665548 -3.985060
H -5.225804 -3.847902 2.975170
H -2.725249 -1.540853 4.614304
H 5.055271 -3.784107 -3.499079
H 2.892742 -0.806481 -4.355165
H 3.593412 -5.152185 2.871668
H -2.904832 1.665124 3.984821
H 2.568378 -2.506886 4.061262
H 2.891924 0.809600 4.357016
H 5.054495 3.786226 3.498180
H 3.593340 5.151440 -2.872438
H 2.567887 2.505644 -4.060933
H -7.312477 -2.223597 0.150611
H -6.000480 -3.189894 -0.583463
H -6.071270 -3.010185 1.167051
H -7.314305 2.219708 -0.156221
H -6.004304 3.186580 0.580608
H -6.072136 3.008061 -1.170156
H 7.307635 2.202085 0.301522
H 6.034647 3.205755 -0.452710
H 6.028587 2.961682 1.291359
H 7.308382 -2.200276 -0.301392
H 6.035476 -3.204894 0.451784
H 6.029770 -2.959411 -1.292094
C 0.000527 1.187686 0.819098
C -0.003674 -0.185929 1.437135
C -0.009026 -1.320664 0.557396
C 0.000875 -1.187446 -0.816438
C -0.008321 1.320796 -0.554746
C -0.002495 0.186022 -1.434407
H -0.011639 -0.306035 2.517038
H -0.021857 -2.328657 0.979304
H 0.021757 -2.077113 -1.442800
H -0.020624 2.328770 -0.976738
H -0.010011 0.306210 -2.514298
H 0.020991 2.077318 1.445520

158
A2*
Ca 1.932234 0.074974 0.255456
Ca -2.280012 0.239527 -0.753824
C 0.260323 0.101671 -1.446842
C -0.354242 -1.044711 -2.118847
C -1.397191 -0.946628 -3.026104
C -1.948900 0.307860 -3.389231
C -0.194779 1.365400 -2.040812
C -1.234132 1.457014 -2.948184
H 0.013153 -2.051938 -1.905461
H -1.803202 -1.865139 -3.459012
H -2.716882 0.387574 -4.159663
H 0.301437 2.296412 -1.751785
H -1.504954 2.439997 -3.344385
H -0.233108 0.140611 -0.068126
N -3.712841 -1.528260 -0.071168
N -3.566903 1.339853 0.901479
C -5.574691 -2.756947 0.973752
H -5.872219 -2.783607 2.031402
H -4.989936 -3.656231 0.741447
H -6.498963 -2.790744 0.375415
C -4.803079 -1.490638 0.671324
C -5.329717 -0.308275 1.242895
H -6.290441 -0.435059 1.744906
C -4.743883 0.958923 1.405536
C -5.517799 1.917721 2.287729
H -5.155825 1.868913 3.326439
H -6.585787 1.663803 2.295247
H -5.394739 2.959112 1.960482
C -3.179447 -2.728653 -0.583418
C -1.965323 -3.202067 -0.027602
C -1.330570 -4.302359 -0.607425
H -0.396874 -4.673092 -0.178117
C -1.867402 -4.924039 -1.732882
H -1.355164 -5.775284 -2.187456
C -3.061724 -4.454964 -2.273121
H -3.479539 -4.947491 -3.155082
C -3.740256 -3.365992 -1.714760
C -2.973102 2.508741 1.421561
C -2.454801 2.511703 2.743557
C -1.940194 3.701483 3.263308
H -1.557759 3.719538 4.286453
C -1.890591 4.863412 2.496416
H -1.489795 5.786641 2.921180
C -2.319292 4.830534 1.172434
H -2.245263 5.733977 0.564506
C -2.852209 3.663687 0.611354
C -2.397481 1.226995 3.554571
C -3.353589 3.651568 -0.821681
C -5.035662 -2.881801 -2.341283
C -1.386738 -2.523219 1.196884
C -2.728920 1.420395 5.033186
C -1.027861 0.561851 3.381921
H -3.137898 0.530884 3.136117
H -2.790496 0.446057 5.542226
H -1.959247 2.010360 5.555813
H -3.691992 1.935956 5.168631
H -0.996617 -0.412683 3.893058
H -0.804973 0.396658 2.314018
H -0.228096 1.190598 3.804622
C -2.578881 4.591274 -1.741020
C -4.859559 3.917434 -0.888987
H -3.204712 2.630008 -1.214288
H -2.886568 4.438643 -2.786534
H -2.772335 5.648636 -1.501663
H -1.494689 4.416790 -1.673955
H -5.095998 4.907729 -0.468286
H -5.215309 3.894261 -1.931291
H -5.421326 3.162029 -0.323037

C 0.135379 -2.497382 1.208731
H -1.732708 -1.476044 1.185117
C -1.949036 -3.143079 2.478316
H -1.547894 -2.638148 3.371098
H -3.045084 -3.062649 2.506486
H -1.684192 -4.210541 2.540569
H 0.490695 -1.807596 1.987828
H 0.583622 -3.475345 1.430015
H 0.519392 -2.185808 0.225695
C -6.069900 -4.005368 -2.445785
H -5.448896 -2.104241 -1.684014
C -4.795301 -2.223929 -3.702045
H -7.036948 -3.611146 -2.795936
H -5.753703 -4.780805 -3.161425
H -6.231259 -4.496919 -1.475031
H -5.746275 -1.888255 -4.145541
H -4.139935 -1.348281 -3.602916
H -4.322484 -2.924048 -4.409405
N 3.575730 1.604581 -0.371595
N 3.888282 -1.122108 0.772353
C 5.674148 2.846853 -0.522668
H 6.702373 2.803985 -0.143104
H 5.188395 3.763093 -0.154095
H 5.709523 2.938275 -1.618807
C 4.878255 1.628333 -0.121381
C 5.595794 0.568191 0.471515
H 6.663438 0.745711 0.599667
C 5.156780 -0.727202 0.810191
C 6.228168 -1.719867 1.203613
H 6.108878 -2.048677 2.246780
H 7.232043 -1.294538 1.085509
H 6.151298 -2.627510 0.586367
C 2.990227 2.623974 -1.158926
C 2.249075 3.650455 -0.529080
C 1.578531 4.589340 -1.318842
H 0.997594 5.379844 -0.838627
C 1.636617 4.524621 -2.708782
H 1.103725 5.260497 -3.315467
C 2.366517 3.508572 -3.320946
H 2.393422 3.451129 -4.411688
C 3.052018 2.549076 -2.570583
C 3.555256 -2.456742 1.080295
C 3.449450 -2.872857 2.429945
C 3.074962 -4.192843 2.696411
H 2.996443 -4.534257 3.730396
C 2.795981 -5.083676 1.662406
H 2.514684 -6.114819 1.888781
C 2.851024 -4.649730 0.339932
H 2.597319 -5.346658 -0.460256
C 3.208735 -3.335271 0.023566
C 3.683737 -1.877916 3.555867
C 3.200693 -2.836833 -1.413848
C 3.794452 1.421617 -3.265092
C 2.133538 3.672682 0.981047
C 4.245603 -2.513249 4.825819
C 2.406617 -1.091496 3.866781
H 4.420620 -1.143224 3.201028
H 4.522897 -1.734572 5.552750
H 3.511958 -3.169839 5.319507
H 5.142269 -3.114127 4.610778
H 2.575704 -0.358364 4.670549
H 2.056106 -0.526056 2.989095
H 1.588363 -1.759750 4.175375
C 2.331669 -3.677447 -2.345315
C 4.611524 -2.683080 -1.989714
H 2.760337 -1.821640 -1.405682
H 2.211027 -3.162086 -3.309070
H 2.786827 -4.659854 -2.549554
H 1.327115 -3.847036 -1.931299
H 5.149531 -3.643907 -1.957107
H 4.561121 -2.356387 -3.039547
H 5.197164 -1.935798 -1.439506
C 0.843136 2.979788 1.424363
H 2.986354 3.097245 1.374049
C 2.207293 5.075164 1.582166
H 2.218238 5.021681 2.681921
H 3.115584 5.602994 1.254726
H 1.336734 5.686428 1.298462
H 0.801477 2.851212 2.514636
H -0.038672 3.562850 1.126376
H 0.673076 1.993136 0.959970
C 4.751731 1.929166 -4.344534
H 4.396342 0.905510 -2.502790
C 2.808556 0.394758 -3.829193
H 5.344040 1.096332 -4.754886
H 4.211137 2.390480 -5.186149
H 5.451869 2.680782 -3.947914
H 3.346117 -0.449057 -4.290467
H 2.147307 0.006354 -3.041061
H 2.160481 0.846063 -4.596887

158

A3

Ca -1.661319 -0.484468 0.401999
Ca 1.669376 0.318044 0.411649
C -0.039747 0.131256 -1.497477
C -0.371150 1.304410 -2.219513
C -0.366690 1.365192 -3.615816
C 0.006817 0.245163 -4.360272
C 0.321368 -0.980059 -2.301884
C 0.361470 -0.930412 -3.698776
H -0.630135 2.220919 -1.678255
H -0.635347 2.296744 -4.120861
H 0.023764 0.289962 -5.452703
H 0.568759 -1.938983 -1.829290
H 0.651416 -1.817795 -4.267690
H 0.085697 -0.451829 1.689616
N 2.775641 2.383754 0.155977
N 3.841300 -0.272383 1.073257
C 4.355096 4.213464 0.526031
H 4.553976 4.460785 -0.527777
H 5.256170 4.435731 1.110730
H 3.539586 4.878128 0.848470
C 3.946343 2.765338 0.660281
C 4.876256 1.907433 1.278956
H 5.786770 2.393227 1.630045

C 4.871938 0.500598 1.390972
C 6.150689 -0.128115 1.896848
H 6.374806 -1.057617 1.354577
H 6.056622 -0.398413 2.959639
H 6.998158 0.561354 1.797100
C 2.076334 3.270167 -0.699769
C 0.966976 3.999277 -0.215160
C 0.211909 4.764520 -1.109194
H -0.655002 5.318923 -0.742475
C 0.550465 4.828898 -2.458608
H -0.050644 5.427131 -3.147562
C 1.661026 4.128963 -2.923488
H 1.922955 4.180739 -3.983175
C 2.440290 3.348963 -2.065111
C 3.935769 -1.675643 1.190251
C 3.860886 -2.309617 2.454425
C 3.948138 -3.703534 2.508673
H 3.900260 -4.204853 3.478198
C 4.077200 -4.466425 1.352113
H 4.145821 -5.554851 1.417552
C 4.087374 -3.839567 0.108918
H 4.159573 -4.445650 -0.795697
C 4.007738 -2.447791 0.002635
C 3.626014 -1.509527 3.723830
C 4.045921 -1.760171 -1.351797
C 3.624576 2.567358 -2.604896
C 0.572448 3.890808 1.242275
C 4.553856 -1.921217 4.867662
C 2.156641 -1.595854 4.150429
H 3.828101 -0.454120 3.491233
H 4.433729 -1.240298 5.724579
H 4.330702 -2.937914 5.227793
H 5.611312 -1.903722 4.562484
H 1.969571 -0.959739 5.030517
H 1.475595 -1.272722 3.347116
H 1.887672 -2.629765 4.420684
C 3.532479 -2.628236 -2.496464
C 5.445032 -1.217439 -1.658757
H 3.385328 -0.875878 -1.295077
H 3.406209 -2.017487 -3.402289
H 4.238170 -3.438124 -2.740404
H 2.559326 -3.082432 -2.259674
H 6.183327 -2.034919 -1.654164
H 5.468306 -0.741262 -2.650800
H 5.756290 -0.466813 -0.920316
C -0.528600 2.844243 1.403472
H 1.460398 3.536345 1.788728
C 0.139537 5.217475 1.862662
H -0.037043 5.094956 2.942410
H 0.907244 5.993839 1.724790
H -0.800934 5.584833 1.424117
H -0.755711 2.645675 2.458038
H -1.454045 3.170653 0.910827
H -0.248848 1.883770 0.939886
C 4.582807 3.441397 -3.415320
H 4.182592 2.175011 -1.742660
C 3.151023 1.363857 -3.420184
H 5.475651 2.865839 -3.706014
H 4.115387 3.810497 -4.342018
H 4.917176 4.318370 -2.840221
H 4.005229 0.753950 -3.752698
H 2.473523 0.721896 -2.839786
H 2.593600 1.682798 -4.313430
N -2.916891 -2.399974 -0.048018
N -3.827977 0.192352 1.052367
C -4.690562 -4.103107 -0.130983
H -3.885495 -4.807149 -0.377889
H -5.338387 -4.011678 -1.017096
H -5.305465 -4.523049 0.677586
C -4.154293 -2.745805 0.264761
C -5.061153 -1.894274 0.929892
H -6.034019 -2.334220 1.151910
C -4.934059 -0.525847 1.235337
C -6.169219 0.149981 1.788010
H -6.033223 0.406432 2.849415
H -7.051404 -0.495882 1.701153
H -6.364634 1.096675 1.263666
C -2.113381 -3.173171 -0.905503
C -0.960292 -3.802838 -0.372641
C -0.086171 -4.457130 -1.246131
H 0.807737 -4.941958 -0.849925
C -0.337213 -4.494461 -2.616452
H 0.357458 -5.007158 -3.286037
C -1.468576 -3.865803 -3.129926
H -1.648476 -3.882200 -4.207609
C -2.367821 -3.193666 -2.298450
C -3.842763 1.573971 1.340574
C -3.705887 2.033465 2.672903
C -3.688263 3.411676 2.908466
H -3.584211 3.780032 3.931649
C -3.798122 4.323676 1.862960
H -3.792015 5.396754 2.067885
C -3.892667 3.863668 0.550964
H -3.949145 4.586220 -0.264603
C -3.897566 2.495096 0.262826
C -3.537499 1.052536 3.821392
C -3.948923 1.991375 -1.170984
C -3.552062 -2.450630 -2.890498
C -0.725555 -3.799465 1.126642
C -4.373902 1.424160 5.046290
C -2.061643 0.886887 4.197881
H -3.883880 0.071275 3.466665
H -4.327687 0.623121 5.800189
H -4.008920 2.344615 5.528709
H -5.430545 1.584646 4.782559
H -1.947800 0.153312 5.011724
H -1.450017 0.533938 3.352288
H -1.632512 1.841431 4.543514
C -3.467446 3.015313 -2.194851
C -5.334909 1.462712 -1.553052
H -3.261798 1.127627 -1.232790
H -3.343714 2.532128 -3.175034
H -4.192441 3.835460 -2.319980
H -2.499057 3.454745 -1.915092
H -6.097000 2.248064 -1.426180
H -5.345668 1.145745 -2.607243

H -5.622545 0.596388 -0.943928
C 0.738432 -3.928635 1.516972
H -1.080248 -2.829882 1.520729
C -1.575309 -4.873321 1.813244
H -1.418448 -4.854949 2.903304
H -2.646213 -4.722362 1.623120
H -1.301151 -5.874589 1.444439
H 0.857233 -3.790261 2.599512
H 1.146012 -4.919927 1.270212
H 1.368785 -3.178198 1.017981
C -4.369791 -3.322300 -3.844376
H -4.210760 -2.156763 -2.062125
C -3.101104 -1.156622 -3.570875
H -5.276605 -2.789853 -4.171491
H -3.799304 -3.580960 -4.750516
H -4.678646 -4.264301 -3.366453
H -3.970256 -0.579333 -3.923164
H -2.518756 -0.519032 -2.889982
H -2.456539 -1.365236 -4.437225

170
A4
Ca -1.484012 0.071459 0.190783
Ca 1.863312 -0.003602 -0.521419
C 0.515400 -0.963604 1.444541
C 0.488562 -2.371439 1.602014
C 0.803605 -3.009588 2.804822
C 1.192673 -2.254872 3.912373
C 0.911880 -0.244681 2.601735
C 1.255427 -0.865717 3.806719
H 0.224656 -3.009175 0.751451
H 0.761425 -4.099875 2.869394
H 1.449089 -2.748677 4.853653
H 0.939595 0.852097 2.580912
H 1.553961 -0.259747 4.666045
H 0.001692 0.937000 -1.134271
N 3.231615 -1.737395 -1.341946
N 3.780079 1.214411 -1.109444
C 4.897645 -2.897972 -2.704885
H 5.340761 -3.519136 -1.911734
H 5.674819 -2.674954 -3.446133
H 4.113837 -3.512038 -3.173181
C 4.306952 -1.636699 -2.119536
C 4.984431 -0.436215 -2.409985
H 5.850402 -0.544929 -3.063138
C 4.803412 0.857293 -1.875097
C 5.876710 1.868949 -2.208761
H 6.104076 2.503629 -1.340568
H 5.540065 2.542511 -3.011667
H 6.796629 1.375065 -2.545360
C 2.836840 -3.019875 -0.885189
C 1.742712 -3.683639 -1.484170
C 1.293007 -4.888671 -0.935833
H 0.436943 -5.397113 -1.385404
C 1.918464 -5.445234 0.176889
H 1.555063 -6.385671 0.598130
C 3.010058 -4.796403 0.748700
H 3.495888 -5.233754 1.624430
C 3.488760 -3.588499 0.234677
C 3.698033 2.522302 -0.585053
C 3.299408 3.611698 -1.397116
C 3.217890 4.881730 -0.819050
H 2.918314 5.732085 -1.436121
C 3.496342 5.082517 0.529417
H 3.429428 6.084272 0.960253
C 3.831218 3.996896 1.334156
H 4.020240 4.156533 2.397021
C 3.926850 2.707560 0.802374
C 2.903747 3.407746 -2.848993
C 4.314224 1.525804 1.674804
C 4.651615 -2.879789 0.905311
C 1.021961 -3.046435 -2.652476
C 3.519419 4.446316 -3.787255
C 1.378602 3.375071 -2.988027
H 3.270974 2.418073 -3.156073
H 3.306649 4.190507 -4.836866
H 3.107748 5.452228 -3.608907
H 4.611501 4.509828 -3.665127
H 1.086076 3.163230 -4.028969
H 0.928451 2.599756 -2.347983
H 0.938449 4.344627 -2.703790
C 3.940237 1.698494 3.143775
C 5.800450 1.188893 1.520085
H 3.764135 0.643073 1.301556
H 4.071327 0.746578 3.678616
H 4.578211 2.446364 3.640809
H 2.892058 2.009274 3.263907
H 6.421441 2.055218 1.798019
H 6.078729 0.345973 2.170781
H 6.043210 0.907778 0.486710
C -0.204297 -2.289971 -2.143452
H 1.714331 -2.314072 -3.096314
C 0.621353 -4.033954 -3.746040
H 0.188130 -3.497995 -4.604580
H 1.488061 -4.611530 -4.101446
H -0.142320 -4.744020 -3.393688
H -0.679742 -1.698898 -2.934628
H -0.951951 -2.985004 -1.738133
H 0.061080 -1.604373 -1.320880
C 5.858635 -3.795602 1.112150
H 4.966752 -2.060349 0.243510
C 4.203565 -2.247885 2.223389
H 6.711130 -3.223862 1.511260
H 5.641869 -4.603195 1.829284
H 6.177423 -4.265593 0.169223
H 5.021921 -1.671824 2.682216
H 3.345903 -1.575605 2.079733
H 3.883457 -3.015537 2.943576
N -2.817813 1.295881 1.684649
N -3.642758 -0.723610 -0.350131
C -4.739433 2.409159 2.748414
H -3.989332 3.055538 3.221198
H -5.224000 1.818731 3.541506
H -5.517782 3.033678 2.287303
C -4.127318 1.491840 1.714573
C -5.037897 0.867883 0.835852

H -6.077019 1.177376 0.954293
C -4.829723 -0.202645 -0.058777
C -6.071552 -0.779015 -0.699719
H -6.146343 -0.449617 -1.747565
H -6.979649 -0.457416 -0.175140
H -6.035324 -1.877454 -0.717470
C -1.979756 1.743618 2.726141
C -1.035228 2.764629 2.455124
C -0.124699 3.125824 3.452397
H 0.607710 3.910134 3.252894
C -0.135296 2.494920 4.694624
H 0.585154 2.785504 5.462936
C -1.059190 1.484775 4.947021
H -1.048643 0.978173 5.915203
C -1.986317 1.086704 3.980206
C -3.557278 -1.880740 -1.158752
C -3.627393 -1.793298 -2.569283
C -3.479097 -2.962314 -3.323045
H -3.529921 -2.904698 -4.412986
C -3.264051 -4.193305 -2.712195
H -3.157757 -5.096518 -3.317689
C -3.167361 -4.267331 -1.323759
H -2.977150 -5.233105 -0.853277
C -3.294774 -3.124934 -0.527461
C -3.818441 -0.458190 -3.263029
C -3.158186 -3.202406 0.984333
C -2.927018 -0.070948 4.262496
C -1.059797 3.477495 1.117955
C -4.896268 -0.499960 -4.346117
C -2.501200 0.074772 -3.831021
H -4.140650 0.264775 -2.503482
H -5.095803 0.517278 -4.716189
H -4.587884 -1.109725 -5.210270
H -5.839925 -0.919814 -3.965116
H -2.664860 1.039100 -4.332972
H -1.751889 0.247375 -3.043017
H -2.070010 -0.627857 -4.563121
C -2.376189 -4.421237 1.465509
C -4.517905 -3.129222 1.686875
H -2.594483 -2.307290 1.303243
H -2.141794 -4.316006 2.534775
H -2.957337 -5.349446 1.344383
H -1.425660 -4.540379 0.925966
H -5.174250 -3.944632 1.343373
H -4.392743 -3.229714 2.775876
H -5.023938 -2.173707 1.499162
C 0.293806 4.013072 0.678045
H -1.375089 2.738531 0.361843
C -2.120204 4.581850 1.108494
H -2.162783 5.074204 0.125177
H -3.119836 4.182022 1.325423
H -1.885159 5.342644 1.869820
H 0.235311 4.401994 -0.347458
H 0.638400 4.841114 1.314344
H 1.073840 3.237724 0.694009
C -3.709490 0.118489 5.563023
H -3.654141 -0.122393 3.440289
C -2.166503 -1.398161 4.262562
H -4.456235 -0.681934 5.683777
H -3.048476 0.083650 6.443557
H -4.236068 1.084467 5.582412
H -2.858934 -2.243568 4.398228
H -1.616518 -1.550214 3.323262
H -1.425185 -1.433480 5.074591
C -2.410609 3.724066 -3.580740
C -3.662800 3.793762 -4.193704
C -4.798903 3.342767 -3.516259
C -4.681074 2.830093 -2.223565
C -2.292530 3.202057 -2.291242
C -3.428363 2.760841 -1.611919
H -3.755011 4.198621 -5.204721
H -5.778969 3.396246 -3.997083
H -5.560194 2.475301 -1.680988
H -1.309447 3.120195 -1.824793
H -3.356978 2.360113 -0.599113
H -1.519508 4.066935 -4.111501

170
A5*
Ca 1.797412 -0.163600 0.076700
Ca -2.027780 0.232103 0.077619
H -0.107395 0.104241 -0.696467
C 1.494998 -0.135254 2.635293
C 2.538151 0.672853 3.141335
C 3.426967 0.200154 4.109074
C 3.322285 -1.109740 4.588745
C 1.399316 -1.434391 3.173965
C 2.304180 -1.936875 4.111127
H 2.723512 1.684404 2.770392
H 4.213555 0.856139 4.482739
H 4.026481 -1.478929 5.338670
H 0.563888 -2.073004 2.858191
H 2.203611 -2.958265 4.488852
C -0.082098 0.476412 2.342785
C -0.346507 1.272467 3.512014
C -1.108159 0.780362 4.549855
C -1.746479 -0.476073 4.472281
C -0.863077 -0.711600 2.230086
C -1.616108 -1.206685 3.297948
H 0.125041 2.252799 3.603183
H -1.240041 1.394071 5.445598
H -2.350567 -0.847624 5.301260
H -0.693040 -1.358472 1.369564
H -2.121819 -2.167033 3.185717
H 0.165743 1.039810 1.437078
N -4.565854 -0.869251 -0.597115
N -4.143970 1.826479 0.008071
C -6.766505 -1.409286 -1.543346
H -6.605752 -2.447508 -1.222131
H -6.632138 -1.386926 -2.636424
H -7.804066 -1.118844 -1.331919
C -5.788996 -0.460483 -0.889119
C -6.226954 0.850086 -0.664997
H -7.272537 1.064471 -0.890613
C -5.432385 1.937271 -0.267183
C -6.118538 3.280669 -0.167501

H -6.732232 3.474092 -1.059204
H -5.395683 4.098463 -0.045933
H -6.799956 3.291757 0.698304
C -4.117163 -2.147621 -0.952442
C -3.901939 -3.154557 0.021414
C -3.343986 -4.367474 -0.395556
H -3.174537 -5.161147 0.333309
C -2.979492 -4.581272 -1.726064
H -2.544147 -5.537484 -2.025005
C -3.164007 -3.571821 -2.668264
H -2.864661 -3.741600 -3.705906
C -3.734144 -2.348880 -2.305861
C -3.360369 2.910354 0.354548
C -3.261377 3.319976 1.717680
C -2.324633 4.267642 2.117949
H -2.253357 4.556793 3.168443
C -1.504697 4.860859 1.185970
C -1.606611 4.518717 -0.144394
H -0.939191 5.000508 -0.859929
C -2.512320 3.538258 -0.606271
C -4.199712 2.670107 2.721554
C -2.477467 3.292415 -2.082462
C -3.914061 -1.223358 -3.326544
C -4.265878 -2.905880 1.473081
C -4.365358 3.471881 4.009771
C -3.778988 1.236163 3.034483
H -5.190247 2.613106 2.243596
H -5.155761 3.026326 4.632487
H -3.442548 3.473962 4.611249
H -4.641833 4.517520 3.806274
H -4.421154 0.794725 3.812110
H -3.890273 0.591238 2.148271
H -2.742658 1.196801 3.386350
C -1.117380 3.717218 -2.540726
C -3.604552 3.960083 -2.875290
H -2.628491 2.209236 -2.259029
H -0.940709 3.413799 -3.581045
H -0.994076 4.809295 -2.495913
H -0.368603 3.288171 -1.893508
H -3.573090 5.053283 -2.742259
H -3.503700 3.742329 -3.950189
H -4.592016 3.607206 -2.549881
C -3.521361 -3.807452 2.453942
H -3.982891 -1.859820 1.699131
C -5.776629 -2.993643 1.710542
H -6.010805 -2.826360 2.773642
H -6.323160 -2.242803 1.126132
H -6.153036 -3.990676 1.431609
H -3.696996 -3.470420 3.486422
H -3.869538 -4.850283 2.390259
H -2.436227 -3.804115 2.277968
C -4.645018 -1.710562 -4.578961
H -4.529851 -0.450801 -2.846068
C -2.598266 -0.548384 -3.724931
H -4.874558 -0.865489 -5.246670
H -4.036104 -2.426073 -5.153983
H -5.590656 -2.211571 -4.323667
H -2.783275 0.274547 -4.433323
H -2.067711 -0.125811 -2.859740
H -1.914959 -1.261110 -4.204598
N 4.330973 0.730448 -1.102524
N 3.578385 -2.004685 -1.150561
C 6.150702 1.208176 -2.676892
H 6.370552 2.097217 -2.071242
H 5.659377 1.561434 -3.597899
H 7.092571 0.725196 -2.969093
C 5.240494 0.240196 -1.952411
C 5.431086 -1.112449 -2.279965
H 6.268201 -1.338528 -2.941982
C 4.620498 -2.190630 -1.911116
C 4.982124 -3.565435 -2.424284
H 5.200885 -3.537202 -3.501638
H 4.177859 -4.290696 -2.239885
H 5.893858 -3.926819 -1.921888
C 4.314532 2.134843 -0.965587
C 4.893056 2.537005 0.270995
C 4.894054 3.880636 0.636749
H 5.348207 4.189533 1.580020
C 4.310104 4.836108 -0.188089
H 4.312860 5.889227 0.103060
C 3.714648 4.440126 -1.379010
H 3.239140 5.194610 -2.010929
C 3.703060 3.098826 -1.793289
C 2.618771 -2.894898 -0.710706
C 2.892416 -3.571483 0.496613
C 1.838650 -4.248540 1.119634
H 2.006563 -4.771535 2.063784
C 0.569598 -4.255983 0.540130
C 0.313579 -3.598283 -0.658708
H -0.693643 -3.603410 -1.080077
C 1.338398 -2.889369 -1.301975
C 4.307825 -3.463089 1.051600
C 1.154078 -2.118614 -2.593889
C 2.977391 2.759642 -3.059112
C 5.510883 1.450984 1.139431
C 4.622718 -4.538266 2.087531
C 4.607914 -2.049712 1.588955
H 4.996006 -3.607207 0.205111
H 5.691151 -4.509666 2.349666
H 4.057054 -4.385391 3.020046
H 4.391997 -5.546150 1.710308
H 5.612089 -2.008539 2.038847
H 4.615892 -1.324615 0.760819
H 3.890757 -1.721806 2.343088
C -0.262200 -1.645928 -2.841061
C 1.667302 -2.923466 -3.792265
H 1.804493 -1.224181 -2.528117
H -0.283554 -0.968382 -3.704112
H -0.944019 -2.480333 -3.062698
H -0.670135 -1.111966 -1.977051
H 1.086926 -3.852716 -3.905970
H 1.569753 -2.341997 -4.722601
H 2.723410 -3.196030 -3.673809
C 5.664912 1.801839 2.603372
H 4.835153 0.575975 1.076626
C 6.867675 0.990646 0.595464

H 7.299244 0.214315 1.246405
H 6.784767 0.569108 -0.411609
H 7.572956 1.836190 0.558911
H 5.967530 0.896149 3.151966
H 6.446594 2.562284 2.761378
H 4.733187 2.180922 3.040392
C 3.403003 3.601021 -4.263378
H 3.192990 1.709174 -3.310572
C 1.499209 2.870981 -2.773477
H 2.900245 3.250232 -5.178320
H 3.140449 4.662532 -4.132349
H 4.489566 3.546291 -4.427349
H 0.909709 2.588683 -3.655307
H 1.208243 2.205788 -1.947374
H 1.220546 3.896395 -2.481014
H -0.233384 -4.785076 1.034109
H -0.778176 5.597800 1.498731

170
A5*-ALT
Ca -1.933342 0.275019 -0.294845
Ca 1.945489 0.013588 0.459224
H 0.228652 0.131812 -0.850179
C -2.361740 -0.446773 2.119369
C -2.630389 -1.818174 2.278285
C -3.515181 -2.278924 3.253824
C -4.164206 -1.364213 4.088403
C -3.032483 0.454577 2.963818
C -3.934722 0.006615 3.931396
H -2.135573 -2.553446 1.632496
H -3.711680 -3.350323 3.354009
H -4.855436 -1.718520 4.857645
H -2.821327 1.528535 2.882767
H -4.450419 0.722329 4.578733
C -0.329254 0.000125 2.043520
C 0.090890 -0.380996 3.368343
C 0.326482 0.574613 4.335834
C 0.266482 1.951896 4.045097
C -0.093008 1.380548 1.712890
C 0.069879 2.333588 2.723299
H 0.088637 -1.436183 3.637140
H 0.566854 0.246277 5.350817
H 0.422668 2.696590 4.827142
H -0.209956 1.750687 0.688603
H 0.071649 3.387780 2.441508
H -0.191351 -0.767777 1.257416
N 4.009725 1.145156 0.408966
N 3.373731 -1.881428 0.683925
C 6.421028 1.467341 0.774911
H 6.828606 1.315967 -0.238431
H 7.177625 1.127562 1.493419
H 6.260742 2.546131 0.890696
C 5.145326 0.664880 0.905510
C 5.302540 -0.613836 1.485330
H 6.263530 -0.765530 1.976924
C 4.564088 -1.796410 1.290296
C 5.272069 -3.063068 1.730699
H 5.727625 -3.567653 0.866014
H 4.579793 -3.787910 2.173818
H 6.068130 -2.834075 2.450317
C 4.079307 2.393821 -0.263528
C 3.808590 3.589140 0.442133
C 3.953362 4.803184 -0.237884
H 3.766716 5.740510 0.288232
C 4.311904 4.841996 -1.583037
H 4.418109 5.802516 -2.092768
C 4.503590 3.654673 -2.283009
H 4.746920 3.690572 -3.347029
C 4.390346 2.417851 -1.640933
C 3.013580 -3.185806 0.249579
C 2.150558 -3.995384 1.021846
C 1.920868 -5.311619 0.607004
H 1.271532 -5.957555 1.199658
C 2.500800 -5.812953 -0.555457
C 3.267451 -4.974554 -1.359603
H 3.664027 -5.354813 -2.302937
C 3.515541 -3.648652 -0.990229
C 1.443151 -3.429312 2.241400
C 4.236396 -2.699962 -1.936694
C 4.511537 1.119508 -2.417205
C 3.330424 3.546783 1.885850
C 2.307559 -3.391169 3.504702
C 0.117343 -4.127589 2.532422
H 1.212453 -2.378459 1.992866
H 1.711644 -3.048010 4.364538
H 2.692316 -4.395372 3.743810
H 3.158735 -2.707070 3.400830
H -0.450145 -3.574215 3.294773
H -0.512260 -4.201200 1.634636
H 0.275218 -5.144717 2.924138
C 3.908415 -2.987644 -3.402355
C 5.754985 -2.650854 -1.740219
H 3.854705 -1.694400 -1.696186
H 4.306561 -2.187554 -4.044991
H 4.365019 -3.928248 -3.748108
H 2.823969 -3.057708 -3.572263
H 6.195751 -3.657807 -1.819411
H 6.218581 -2.020348 -2.515227
H 6.030235 -2.223286 -0.767956
C 2.508352 4.774007 2.272933
H 2.664601 2.669176 1.967232
C 4.455166 3.337988 2.904676
H 4.047148 3.374379 3.926751
H 4.947665 2.365494 2.784898
H 5.218536 4.127495 2.815236
H 2.048737 4.620827 3.260123
H 3.133336 5.678225 2.343472
H 1.705020 4.973959 1.549118
C 5.600271 1.140921 -3.487108
H 4.773877 0.330681 -1.696514
C 3.151872 0.753579 -3.024776
H 5.713999 0.142487 -3.936343
H 5.363548 1.838009 -4.306235
H 6.572244 1.436513 -3.063996
H 3.195676 -0.205276 -3.561759
H 2.355057 0.670818 -2.266599

H 2.821360 1.527131 -3.735960
N -3.325388 -0.943114 -1.739853
N -3.029353 1.970884 -1.514430
C -4.649992 -1.639524 -3.694093
H -5.743821 -1.582755 -3.582681
H -4.333459 -2.654991 -3.423674
H -4.423455 -1.462566 -4.755437
C -3.983001 -0.589928 -2.833514
C -4.137496 0.742817 -3.266522
H -4.693808 0.871362 -4.197109
C -3.765109 1.934224 -2.613607
C -4.254624 3.216413 -3.254274
H -3.866508 3.292255 -4.281789
H -3.936236 4.104818 -2.693631
H -5.352702 3.220489 -3.328795
C -3.291257 -2.265922 -1.257698
C -4.386594 -2.818205 -0.549030
C -4.230790 -4.068496 0.055961
H -5.067533 -4.495481 0.614765
C -3.033661 -4.774619 -0.025326
H -2.933311 -5.746206 0.464246
C -1.965381 -4.236054 -0.738561
H -1.030124 -4.795006 -0.817106
C -2.074929 -2.989507 -1.363167
C -2.836006 3.125052 -0.736630
C -3.873905 3.603738 0.107797
C -3.571416 4.617250 1.021405
H -4.350195 4.989040 1.688719
C -2.295105 5.171056 1.098332
C -1.297574 4.727535 0.236621
H -0.309077 5.187409 0.279582
C -1.543338 3.705869 -0.686501
C -5.276198 3.024402 0.003586
C -0.471988 3.250365 -1.660695
C -0.933792 -2.440584 -2.196482
C -5.710470 -2.086973 -0.430513
C -6.351952 3.917656 0.616198
C -5.370079 1.610038 0.575898
H -5.506511 2.945060 -1.069561
H -7.349578 3.511711 0.389694
H -6.266352 3.967756 1.713461
H -6.306157 4.945046 0.223628
H -6.390588 1.216726 0.459268
H -4.698820 0.918951 0.048412
H -5.117138 1.589496 1.644783
C 0.943482 3.510076 -1.171126
C -0.679636 3.876059 -3.042968
H -0.578298 2.158596 -1.793342
H 1.679307 3.041580 -1.837419
H 1.185178 4.582259 -1.143540
H 1.109851 3.118487 -0.157780
H -0.612286 4.974174 -2.983912
H 0.091214 3.523122 -3.746071
H -1.660981 3.617692 -3.458661
C -6.027911 -1.720923 1.019221
H -5.618765 -1.145811 -0.991384
C -6.851538 -2.897632 -1.052615
H -7.785180 -2.313036 -1.056867
H -6.625701 -3.191068 -2.088069
H -7.043232 -3.820371 -0.481873
H -6.978988 -1.169163 1.079535
H -6.123006 -2.619751 1.647966
H -5.239172 -1.098221 1.459056
C -1.021387 -2.941022 -3.641047
H -1.047935 -1.345092 -2.243172
C 0.431007 -2.725427 -1.591588
H -0.210483 -2.513088 -4.251959
H -0.936085 -4.038897 -3.678189
H -1.978624 -2.660895 -4.102845
H 1.206634 -2.131461 -2.095752
H 0.446460 -2.463406 -0.523961
H 0.721973 -3.782849 -1.664979
H -2.083634 5.960189 1.823738
H 2.322335 -6.848590 -0.854401

168

A6

Ca 1.584487 -0.470417 0.360994
Ca -1.624314 0.541692 0.018837
N 2.226865 -2.686744 0.596375
N 3.921853 -0.239086 0.693877
N -3.850348 -0.241962 -0.293286
N -2.769074 2.579431 0.186127
C 0.659713 -0.270406 4.381883
C 0.81259 -0.235753 2.993647
C -0.248886 -0.020686 2.087192
C -1.501507 0.152955 2.718266
C -1.693002 0.117233 4.10133
C -0.603618 -0.093981 4.943745
C 3.391062 -4.695145 1.419855
H 2.644392 -5.244299 0.832835
H 3.244319 -4.940466 2.482669
H 4.394361 -5.046517 1.140282
C 3.27527 -3.204237 1.206897
C 4.368817 -2.431082 1.655184
H 5.140927 -2.989448 2.187349
C 4.715103 -1.10708 1.321548
C 6.134383 -0.71087 1.668538
H 6.238659 0.373045 1.805269
H 6.819264 -1.004153 0.858615
H 6.465305 -1.227111 2.579958
C 1.131766 -3.409814 0.086155
C 0.971088 -3.454445 -1.328972
C -0.228553 -3.94299 -1.852232
H -0.372315 -3.981293 -2.932398
C -1.251374 -4.39151 -1.020403
H -2.193181 -4.738001 -1.445217
C -1.063091 -4.394075 0.355185
H -1.858353 -4.771851 0.999909
C 0.11793 -3.920633 0.936852
C 4.501476 0.919399 0.125406
C 5.274425 0.812687 -1.056434
C 5.79323 1.976626 -1.632385
H 6.385522 1.901265 -2.547907
C 5.568785 3.224869 -1.063981
H 5.988715 4.12375 -1.521725

C 4.792331 3.324254 0.08806
H 4.607903 4.309164 0.517546
C 4.238319 2.19156 0.691904
C -6.290778 -0.158713 -0.61925
H -6.282302 -0.382987 -1.696616
H -7.133642 0.510872 -0.410772
H -6.459521 -1.117916 -0.115425
C -4.974291 0.476611 -0.220523
C -5.064959 1.828382 0.163618
H -6.083547 2.207383 0.247409
C -4.06444 2.812953 0.305541
C -4.567536 4.209749 0.592292
H -3.836061 4.976925 0.308178
H -4.758715 4.30843 1.672511
H -5.516159 4.404941 0.074602
C -1.830304 3.630955 0.084872
C -1.407912 4.00841 -1.216871
C -0.403924 4.96971 -1.343032
H -0.060567 5.260989 -2.336813
C 0.180275 5.55199 -0.219812
H 0.967664 6.30047 -0.337687
C -0.241021 5.175373 1.051155
H 0.216138 5.638837 1.928771
C -1.241828 4.213634 1.231002
H -0.737371 -0.123482 6.027808
H -2.695032 0.254405 4.516478
H 1.529488 -0.44219 5.021577
H 1.853814 -0.403285 2.662528
C 0.385105 0.766171 -1.486934
H -2.435292 0.3195 2.151722
C -4.002547 -1.566502 -0.774003
C -3.80356 -1.810886 -2.152776
C -4.164054 -3.059548 -2.67082
H -4.05118 -3.257574 -3.738093
C -4.670823 -4.054233 -1.841073
H -4.976085 -5.01615 -2.259984
C -4.727824 -3.84496 -0.464353
H -5.054687 -4.66394 0.176922
C -4.36642 -2.618958 0.10581
C 5.512515 -0.521375 -1.743223
C -2.060239 3.383447 -2.438207
C -3.208525 -0.732477 -3.048846
C -4.292844 -2.452618 1.624306
C -1.696738 3.852432 2.633067
C 3.364957 2.319393 1.929246
C 0.290426 -4.013528 2.442319
C 2.11387 -3.029565 -2.240053
C 0.591081 0.277982 -2.79596
C 1.500963 0.853463 -3.687399
C 2.258971 1.959661 -3.293684
C 1.161785 1.901528 -1.147141
C 2.085228 2.491497 -2.017966
H 0.022339 -0.592936 -3.133413
H 1.631064 0.431981 -4.688382
H 2.98797 2.401785 -3.976931
H 1.045727 2.371928 -0.157677
H 2.669307 3.355724 -1.697091
C 4.722575 -0.597152 -3.050592
C 6.997089 -0.796083 -1.994531
H 5.128858 -1.314254 -1.085228
H 4.865356 -1.573552 -3.538845
H 3.649034 -0.449146 -2.876942
H 5.047183 0.183132 -3.757154
H 7.139678 -1.816017 -2.38478
H 7.418158 -0.09938 -2.736686
H 7.593831 -0.696529 -1.075838
C 2.852273 3.735231 2.159029
H 2.472024 1.686764 1.76492
C 4.041567 1.792747 3.197405
H 2.146869 3.744158 2.998836
H 3.672508 4.424751 2.413415
H 2.328104 4.130175 1.277478
H 3.369179 1.907164 4.061926
H 4.301416 0.730608 3.11244
H 4.963808 2.358161 3.405049
C -0.83772 -3.334911 3.215635
C 0.398036 -5.47939 2.883663
H 1.225386 -3.495956 2.702618
H -0.60858 -3.314493 4.291688
H -0.98931 -2.302466 2.884617
H -1.785175 -3.876723 3.091667
H 0.6312 -5.545182 3.958385
H -0.558835 -6.000409 2.720173
H 1.169535 -6.030628 2.330475
C 1.727732 -2.923441 -3.711932
H 2.454961 -2.031021 -1.91022
C 3.316981 -3.971072 -2.104165
H 4.105681 -3.6904 -2.818572
H 3.755367 -3.937776 -1.100898
H 3.017788 -5.009057 -2.319255
H 2.555349 -2.480962 -4.284765
H 1.522704 -3.916593 -4.142276
H 0.841182 -2.29668 -3.870674
C -4.374435 -3.786054 2.366883
H -3.290898 -2.028574 1.819112
C -5.309767 -1.498543 2.2634
H -4.150196 -3.634782 3.432796
H -5.386513 -4.21733 2.304974
H -3.668579 -4.532323 1.977571
H -5.167308 -1.496309 3.355581
H -5.207455 -0.463683 1.917985
H -6.34174 -1.831395 2.069287
C -2.437311 -1.310523 -4.233493
C -4.231261 0.29988 -3.530557
H -2.471895 -0.179041 -2.438125
H -1.873041 -0.51395 -4.740558
H -1.726719 -2.085524 -3.910754
H -3.111536 -1.757865 -4.980618
H -3.756106 1.010794 -4.224087
H -5.057886 -0.193334 -4.066495
H -4.650775 0.881289 -2.699619
C -1.163139 3.360323 -3.670154
H -2.290947 2.336741 -2.173641
C -3.395822 4.058951 -2.767129
H -1.624531 2.746912 -4.459085
H -1.020382 4.369445 -4.089267

H -0.17686 2.937164 -3.446771
H -3.840723 3.612482 -3.670007
H -4.124057 3.952676 -1.95429
H -3.247221 5.134063 -2.956547
C -0.550142 3.342103 3.505228
C -2.383689 5.039301 3.318131
H -2.431352 3.03852 2.534706
H -0.931627 2.977493 4.470286
H -0.011762 2.513189 3.026545
H 0.170404 4.146839 3.718282
H -2.796485 4.739322 4.294517
H -1.665886 5.855629 3.498259
H -3.201599 5.449706 2.711175

168
A7*
Ca 2.322691 0.225708 0.118433
Ca -2.421304 -0.160264 0.158544
N 3.723687 -1.068973 1.523879
N 4.420135 1.414270 0.054872
N -3.651128 -1.364556 -1.494188
N -4.391439 1.196122 -0.080834
C -1.785854 0.953669 2.659187
C -0.735146 1.198771 1.781246
C 0.038791 0.162216 1.147151
C -0.323499 -1.159909 1.588847
C -1.359990 -1.413978 2.480292
C -2.143745 -0.365979 2.999539
C 5.594508 -1.914865 2.886344
H 5.498777 -1.565313 3.926073
H 6.666537 -2.033110 2.678741
H 5.103728 -2.894704 2.824050
C 4.975833 -0.909818 1.937998
C 5.815392 0.156603 1.563857
H 6.813468 0.138901 2.004956
C 5.570877 1.222520 0.676384
C 6.728621 2.178881 0.473556
H 6.501800 2.952812 -0.270610
H 7.633619 1.636521 0.161482
H 6.969320 2.676109 1.426101
C 2.937780 -2.132822 2.013255
C 2.610223 -3.196559 1.132478
C 1.749348 -4.203010 1.580379
H 1.492104 -5.028873 0.915709
C 1.210616 -4.169270 2.864885
H 0.536944 -4.962333 3.197352
C 1.528134 -3.116452 3.716506
H 1.088790 -3.084890 4.716414
C 2.386424 -2.088438 3.316877
C 4.279020 2.335942 -0.995635
C 4.803309 2.017942 -2.280398
C 4.497605 2.865588 -3.348525
H 4.878982 2.637569 -4.344446
C 3.717919 4.008337 -3.173394
H 3.490995 4.652759 -4.025823
C 3.246159 4.328685 -1.905380
H 2.654840 5.236780 -1.756571
C 3.510664 3.508242 -0.802741
C -5.474771 -2.191002 -2.940577
H -6.325146 -2.675261 -2.434340
H -4.761742 -2.970771 -3.235333
H -5.874651 -1.712282 -3.846384
C -4.858398 -1.167893 -2.005465
C -5.673181 -0.058019 -1.717955
H -6.626949 -0.038614 -2.249181
C -5.456457 1.035116 -0.856277
C -6.562608 2.073218 -0.867613
H -6.297899 2.970214 -0.294723
H -7.490494 1.657663 -0.445915
H -6.784669 2.366555 -1.904234
C -4.365357 2.204720 0.903068
C -3.435430 3.270146 0.780475
C -3.299575 4.170001 1.840856
H -2.585596 4.990935 1.766177
C -4.056634 4.037808 3.003534
H -3.926589 4.744856 3.826178
C -4.985508 3.007352 3.104107
H -5.586053 2.913836 4.012444
C -5.167508 2.087369 2.066368
H -2.933764 -0.560317 3.723259
H -1.556934 -2.441909 2.792666
H -2.343102 1.792904 3.083634
H -0.518171 2.246951 1.556308
C -0.183337 0.218084 -0.893106
H 0.239580 -2.023145 1.218604
C -2.769635 -2.314548 -2.036430
C -2.217516 -2.118330 -3.332161
C -1.250602 -3.019216 -3.782841
H -0.811121 -2.883814 -4.771677
C -0.820427 -4.083853 -2.994426
H -0.059736 -4.771840 -3.370113
C -1.362610 -4.266540 -1.726643
H -1.024290 -5.105647 -1.117517
C -2.338178 -3.396337 -1.228403
C 5.726018 0.816360 -2.463250
C -2.620349 3.421411 -0.493389
C -2.657323 -0.945350 -4.197150
C -2.983407 -3.637454 0.125756
C -6.206301 0.987400 2.218089
C 3.019392 3.962877 0.563443
C 2.678612 -0.942067 4.268879
C 3.225595 -3.249552 -0.255437
C 0.357058 -0.966183 -1.484184
C 1.274927 -0.944480 -2.537983
C 1.735267 0.275338 -3.059282
C 0.313858 1.430537 -1.463996
C 1.236165 1.467774 -2.508767
H 0.018921 -1.946379 -1.128006
H 1.621834 -1.888778 -2.965300
H 2.451083 0.306925 -3.881612
H -0.048567 2.386797 -1.078934
H 1.580493 2.427817 -2.899590
C 3.149928 -1.438382 5.637975
H 3.490816 -0.345379 3.830390
C 1.471762 -0.011691 4.408574
H 3.476755 -0.592458 6.262863

H 2.341641 -1.951448 6.182702
H 3.989068 -2.144789 5.549683
H 1.705151 0.826200 5.084949
H 1.169209 0.401493 3.437826
H 0.598976 -0.545117 4.816362
C 2.443612 -4.107475 -1.242640
H 3.227805 -2.219804 -0.655781
C 4.692352 -3.687208 -0.201790
H 4.779248 -4.683381 0.260115
H 5.116757 -3.742546 -1.216327
H 5.303339 -2.983939 0.377851
H 2.504927 -5.177896 -0.990041
H 1.382191 -3.827246 -1.278608
H 2.859926 -3.992743 -2.254916
C 4.162764 4.468355 1.446860
H 2.362696 4.825058 0.358387
C 2.166453 2.945900 1.320573
H 3.766001 4.938427 2.360862
H 4.813781 3.638858 1.755597
H 4.778520 5.210921 0.918287
H 1.622178 3.425542 2.147527
H 1.397247 2.505698 0.668207
H 2.784501 2.155999 1.774156
C 6.464436 0.816747 -3.799590
H 6.497879 0.903061 -1.682487
C 5.052182 -0.541473 -2.242731
H 5.790930 -1.349834 -2.349952
H 4.638679 -0.621763 -1.228869
H 4.248231 -0.715473 -2.969794
H 7.200027 -0.001477 -3.820956
H 5.777556 0.657335 -4.646467
H 7.004391 1.759842 -3.972618
C -2.311674 -1.114552 -5.674459
H -3.753709 -0.887153 -4.137683
C -2.120212 0.390082 -3.678592
H -2.768653 -0.302876 -6.260733
H -1.224788 -1.064641 -5.847594
H -2.678426 -2.071499 -6.076732
H -2.482039 1.217874 -4.308009
H -2.459109 0.585157 -2.652880
H -1.022122 0.403562 -3.683822
C -2.148626 -4.509373 1.056765
H -3.107066 -2.657016 0.623930
C -4.394917 -4.210648 -0.032579
H -4.357161 -5.183066 -0.548438
H -4.868450 -4.361271 0.950537
H -5.033807 -3.537944 -0.617532
H -2.100148 -5.549670 0.698404
H -1.119885 -4.135867 1.156381
H -2.601595 -4.537230 2.059397
C -1.449713 4.391724 -0.364035
H -2.199194 2.427194 -0.734236
C -3.491337 3.820415 -1.688938
H -2.874990 3.915750 -2.596382
H -4.266205 3.072536 -1.892097
H -3.982328 4.788764 -1.502930
H -0.854094 4.392928 -1.288793
H -1.799581 5.423972 -0.205861
H -0.780893 4.131030 0.469542
C -7.570816 1.546384 2.627961
H -6.329243 0.502672 1.241241
C -5.744919 -0.107900 3.180211
H -8.335870 0.754568 2.600572
H -7.556706 1.950713 3.652494
H -7.891505 2.356509 1.956184
H -6.524828 -0.876984 3.298335
H -4.838516 -0.599252 2.801356
H -5.516642 0.300940 4.177835

168
A8
Ca 2.806574 -0.006799 0.263864
Ca -2.808906 0.209023 -0.366662
N 4.643706 -1.367439 -0.232177
N 3.969768 1.208609 -1.385794
N -4.750618 -1.047231 -0.020757
N -3.947317 1.518496 1.172540
C 1.502934 1.219958 2.358761
C 0.658209 1.215050 1.269437
C 0.280313 -0.032538 0.605370
C 0.690620 -1.245821 1.314500
C 1.545453 -1.200654 2.396936
C 2.072109 0.024827 2.882774
C 6.814597 -2.275512 -0.966299
H 6.605595 -3.077596 -0.246821
H 7.797783 -1.837110 -0.738882
H 6.889803 -2.722901 -1.969880
C 5.736057 -1.213318 -0.961473
C 5.957760 -0.099557 -1.792851
H 6.891377 -0.108230 -2.358660
C 5.120455 1.011814 -2.013832
C 5.634821 2.025228 -3.015600
H 5.803833 1.556727 -3.996901
H 6.607725 2.414055 -2.676998
H 4.946483 2.869717 -3.143370
C 4.504019 -2.345780 0.762351
C 3.540527 -3.371559 0.585751
C 3.299803 -4.252453 1.643931
H 2.567340 -5.052264 1.527027
C 3.968553 -4.115608 2.856570
H 3.760514 -4.805902 3.677470
C 4.902138 -3.094903 3.025965
H 5.410991 -3.001855 3.985930
C 5.194179 -2.196046 1.996048
C 3.054390 2.187054 -1.808835
C 2.346868 2.021911 -3.030323
C 1.420775 3.003182 -3.391767
H 0.875324 2.908868 -4.330442
C 1.163008 4.099161 -2.571529
H 0.441971 4.857084 -2.884174
C 1.815105 4.218646 -1.348740
H 1.589870 5.069500 -0.704516
C 2.765831 3.274847 -0.946132
C -6.939387 -1.837881 0.802573
H -6.859008 -2.625997 0.044781
H -7.935049 -1.376413 0.742465

H -6.865891 -2.319595 1.790097
C -5.852582 -0.790644 0.669401
C -6.075835 0.404410 1.387724
H -7.051110 0.483069 1.870976
C -5.168006 1.438230 1.685622
C -5.633452 2.469055 2.689605
H -5.451819 2.110153 3.715171
H -6.712965 2.648024 2.591926
H -5.095378 3.419810 2.577829
C -2.991241 2.433023 1.649482
C -2.567806 3.486789 0.797359
C -1.547911 4.337719 1.235536
H -1.216891 5.157418 0.596158
C -0.949762 4.160961 2.480826
H -0.162503 4.842039 2.811585
C -1.348948 3.105135 3.295414
H -0.853308 2.952329 4.256659
C -2.360191 2.225689 2.901590
H 2.724350 0.044141 3.754744
H 1.861064 -2.139817 2.857901
H 1.750474 2.178045 2.821600
H 0.236070 2.156630 0.917659
C -0.304530 -0.058100 -0.673713
H 0.322782 -2.211749 0.975613
C -4.550106 -2.298258 -0.638218
C -5.001145 -2.519033 -1.962180
C -4.781678 -3.773846 -2.539859
H -5.141355 -3.969908 -3.551943
C -4.093576 -4.771822 -1.856711
H -3.936832 -5.747863 -2.322133
C -3.566110 -4.507766 -0.594811
H -2.979795 -5.279014 -0.093319
C -3.763825 -3.272014 0.027977
C 2.588149 0.802383 -3.911148
C -3.270563 3.716623 -0.530154
C -5.649865 -1.399297 -2.759175
C -3.102215 -2.949193 1.360794
C -2.718428 1.035003 3.772734
C 3.542077 3.444431 0.349218
C 6.231402 -1.091139 2.174544
C 2.797095 -3.488880 -0.735096
C -0.804173 -1.284936 -1.304000
C -1.677182 -1.241917 -2.372039
C -2.128564 -0.007995 -2.925221
C -0.576392 1.164969 -1.429895
C -1.434143 1.166372 -2.507503
H -0.504686 -2.255406 -0.908988
H -2.068112 -2.184112 -2.766148
H -2.787871 0.012113 -3.791128
H -0.066229 2.087285 -1.151661
H -1.603134 2.106586 -3.038536
C 1.909927 0.895377 -5.275331
C 2.181960 -0.505366 -3.221075
H 3.672387 0.740993 -4.097645
H 2.200303 0.034454 -5.896061
H 0.812596 0.874263 -5.180251
H 2.192801 1.810349 -5.817765
H 2.301910 -1.354708 -3.910506
H 2.830150 -0.712424 -2.357939
H 1.133319 -0.476171 -2.895012
C 2.849065 4.351472 1.360347
C 4.973296 3.922692 0.086945
H 3.631780 2.447969 0.820904
H 3.363987 4.300943 2.331662
H 2.869997 5.404350 1.037577
H 1.799079 4.064654 1.511020
H 4.964783 4.899590 -0.421627
H 5.525153 4.033823 1.033701
H 5.523352 3.212582 -0.542463
C 1.553011 -4.367923 -0.669379
H 2.459274 -2.471871 -1.010263
C 3.711179 -3.959505 -1.870434
H 3.144999 -4.040532 -2.811272
H 4.538291 -3.260356 -2.037714
H 4.134462 -4.949673 -1.638836
H 0.982105 -4.284365 -1.605806
H 1.813913 -5.429935 -0.537080
H 0.889596 -4.081188 0.158178
C 7.078656 -1.256588 3.434009
H 6.923403 -1.165814 1.320759
C 5.639371 0.324744 2.128553
H 7.883088 -0.505826 3.446508
H 6.482690 -1.110748 4.349227
H 7.546019 -2.251337 3.487217
H 6.415563 1.070084 2.360088
H 5.265830 0.572631 1.124060
H 4.824159 0.440147 2.856828
C -2.429028 4.488095 -1.539932
H -3.482653 2.724763 -0.971993
C -4.631763 4.390158 -0.329552
H -2.310306 5.543171 -1.246486
H -1.427207 4.051291 -1.646905
H -2.916373 4.480792 -2.526728
H -4.507195 5.369233 0.159564
H -5.132031 4.552302 -1.297468
H -5.295598 3.777566 0.293401
C -2.917286 1.414328 5.240333
H -3.668314 0.623474 3.404454
C -1.672424 -0.071206 3.622677
H -3.287046 0.549792 5.813423
H -1.974810 1.735864 5.710919
H -3.642181 2.235174 5.351944
H -1.954999 -0.960422 4.207391
H -1.558084 -0.377147 2.573270
H -0.678223 0.259849 3.958024
C -5.285011 -1.454278 -4.243405
C -7.171914 -1.341892 -2.599491
H -5.242101 -0.459609 -2.348818
H -5.580960 -0.518362 -4.741955
H -4.205397 -1.596414 -4.394190
H -5.806461 -2.274115 -4.762741
H -7.595535 -0.552989 -3.241170
H -7.632025 -2.299451 -2.892730
H -7.469917 -1.124178 -1.567655
C -1.913416 -3.851753 1.677253
H -2.702685 -1.920198 1.276159

C -4.073763 -2.931441 2.544745
H -1.351312 -3.447335 2.531363
H -2.238517 -4.869649 1.945149
H -1.224771 -3.932785 0.824648
H -3.520634 -2.804804 3.487830
H -4.790497 -2.105304 2.471702
H -4.631676 -3.879267 2.602581

170
B1
Ca 2.131751 0.261608 -0.248290
Ca -2.230700 0.147620 -0.191494
C 0.622075 -2.346865 4.136049
C -0.040072 -3.345149 3.419548
C 0.175517 -4.688134 3.732309
C 1.046152 -5.032530 4.768451
C 1.702123 -4.033147 5.489953
C 1.492511 -2.690269 5.171305
H 2.011944 -1.907709 5.729837
H 2.384873 -4.302884 6.299380
H 0.462612 -1.297492 3.881315
H -0.726046 -3.075494 2.615474
C -0.054389 -1.043546 0.519096
H 1.216336 -6.083858 5.013172
C -0.016431 -1.048704 -0.893103
C -0.032018 0.124033 -1.652829
C -0.143232 1.456181 -0.939660
C -0.007961 0.295357 1.231581
C -0.075752 1.465817 0.469981
H 0.025674 -2.008267 -1.411930
H -0.099223 0.075510 -2.737322
H -0.084312 2.387097 -1.498762
H -0.054800 0.347697 2.317624
H -0.089921 2.428272 0.985094
H -0.337365 -5.468301 3.164528
H 0.111349 -1.966836 1.068182
N -4.038981 -0.914807 -1.217538
N -4.108511 1.192176 0.713425
C -6.222182 -1.500224 -2.176338
H -5.736055 -2.416873 -2.536870
H -6.418108 -0.866432 -3.056808
H -7.195152 -1.753949 -1.733647
C -5.351708 -0.756582 -1.188995
C -6.004986 0.106882 -0.284954
H -7.095623 0.089846 -0.305831
C -5.413998 0.999026 0.633856
C -6.342817 1.733291 1.573549
H -7.309665 1.941104 1.095215
H -5.901263 2.675144 1.926494
H -6.542946 1.113571 2.463159
C -3.354620 -1.674497 -2.177315
C -2.742436 -2.892151 -1.778808
C -1.911285 -3.553864 -2.686413
H -1.430030 -4.488123 -2.393330
C -1.668861 -3.032215 -3.954919
H -1.010754 -3.560474 -4.648285
C -2.261999 -1.830344 -4.333473
H -2.053676 -1.419456 -5.324350
C -3.105764 -1.134140 -3.464543
C -3.480379 2.006876 1.666270
C -3.249002 1.516186 2.976089
C -2.450500 2.265470 3.843815
H -2.256769 1.892769 4.852687
C -1.886033 3.473630 3.441491
H -1.262764 4.044782 4.133168
C -2.112412 3.947745 2.151032
H -1.653358 4.887393 1.839351
C -2.898478 3.231599 1.244325
C -3.798840 0.165415 3.398685
C -3.139353 3.729968 -0.169232
C -3.687094 0.211064 -3.863925
C -3.004202 -3.446031 -0.389475
C -4.438822 0.195842 4.786478
C -2.721441 -0.918443 3.314495
H -4.581383 -0.112678 2.679787
H -4.920925 -0.768531 5.010199
H -3.695528 0.379057 5.578379
H -5.202086 0.985618 4.855515
H -3.129690 -1.905068 3.583075
H -2.291141 -1.009289 2.303122
H -1.886456 -0.701970 3.997279
C -2.041065 4.651138 -0.689928
C -4.514885 4.391358 -0.300270
H -3.158462 2.835520 -0.816283
H -2.181306 4.835920 -1.765981
H -2.058046 5.632378 -0.188835
H -1.041260 4.218373 -0.547929
H -4.591668 5.264392 0.367653
H -4.683817 4.735749 -1.332989
H -5.323050 3.694076 -0.043972
C -1.887250 -4.342701 0.134938
H -3.076537 -2.575789 0.286925
C -4.356447 -4.162416 -0.322550
H -4.546271 -4.543298 0.693645
H -5.181241 -3.487998 -0.586927
H -4.377697 -5.017135 -1.017622
H -2.073848 -4.596041 1.189693
H -1.831295 -5.294312 -0.417333
H -0.905303 -3.853684 0.067303
C -4.290802 0.196765 -5.268394
H -4.497033 0.444011 -3.159333
C -2.650974 1.329632 -3.719052
H -4.802443 1.149078 -5.477998
H -3.521969 0.062503 -6.045672
H -5.022731 -0.617581 -5.379350
H -3.087745 2.304434 -3.987542
H -2.270180 1.418442 -2.688931
H -1.778747 1.154009 -4.368322
N 3.891700 1.780846 -0.030826
N 4.028206 -0.961231 -0.880754
C 6.043613 2.957809 -0.116785
H 6.131511 3.415335 -1.115835
H 7.060574 2.733439 0.232301
H 5.589206 3.709753 0.542702
C 5.204364 1.702683 -0.195006
C 5.884234 0.498358 -0.467903

H 6.973914 0.556127 -0.487837
C 5.326227 -0.763871 -0.770851
C 6.298770 -1.901443 -0.989509
H 6.914589 -2.059001 -0.090600
H 6.990663 -1.660364 -1.811188
H 5.780018 -2.838844 -1.230231
C 3.196690 2.994467 0.090055
C 2.611002 3.334204 1.337625
C 1.787696 4.461315 1.409048
H 1.328459 4.732738 2.360984
C 1.525840 5.235286 0.281271
H 0.874615 6.109024 0.355236
C 2.086637 4.883050 -0.944110
H 1.858610 5.482538 -1.828647
C 2.921205 3.769511 -1.065204
C 3.388421 -2.197719 -1.026758
C 3.196405 -3.031852 0.108435
C 2.365255 -4.148331 -0.020946
H 2.188261 -4.791216 0.841891
C 1.740661 -4.449651 -1.230032
H 1.093965 -5.326708 -1.305010
C 1.936953 -3.629716 -2.337484
H 1.433448 -3.866728 -3.275790
C 2.744651 -2.492165 -2.256617
C 3.864843 -2.685454 1.432707
C 2.952312 -1.572327 -3.444231
C 3.455099 3.347395 -2.423345
C 2.877803 2.468238 2.556177
C 3.786101 -3.810413 2.459402
C 3.327547 -1.375800 2.033726
H 4.932942 -2.517304 1.217802
H 4.368460 -3.545423 3.354188
H 2.753115 -3.990343 2.789430
H 4.189083 -4.753705 2.060799
H 3.617295 -1.280807 3.090257
H 3.750307 -0.498618 1.521519
H 2.226447 -1.348430 2.004359
C 1.807176 -1.602237 -4.451314
C 4.292183 -1.854018 -4.131411
H 3.016180 -0.549047 -3.035301
H 1.930142 -0.795129 -5.189789
H 1.779523 -2.550846 -5.011314
H 0.831003 -1.473510 -3.963430
H 4.312752 -2.878790 -4.535911
H 4.459055 -1.153827 -4.965463
H 5.130799 -1.750492 -3.430955
C 1.790993 2.554283 3.623082
H 2.898635 1.424825 2.198909
C 4.258241 2.747609 3.156972
H 4.438281 2.104811 4.033426
H 5.058323 2.554028 2.430793
H 4.335934 3.797487 3.482062
H 1.955742 1.783333 4.391583
H 1.796766 3.528722 4.137401
H 0.787563 2.402797 3.201069
C 3.998717 4.520682 -3.238212
H 4.286907 2.650718 -2.251967
C 2.397065 2.570831 -3.212903
H 4.480612 4.158979 -4.159891
H 3.200999 5.217354 -3.540808
H 4.742583 5.094651 -2.664990
H 2.798429 2.235579 -4.182209
H 2.053311 1.674016 -2.674891
H 1.506414 3.189935 -3.404226

170
B2*
Ca 2.375835 -0.602733 -0.839162
Ca -2.268227 0.423044 -0.446783
C 2.357364 -2.329782 -3.425671
C 1.084166 -2.095474 -3.899804
C 0.594573 -0.754085 -4.117396
C 1.610504 0.271458 -4.108255
C 2.875230 0.030898 -3.614453
C 3.277889 -1.268278 -3.189514
H 4.326513 -1.488310 -2.979189
H 3.587359 0.858353 -3.586973
H 2.688605 -3.360228 -3.272423
H 0.422783 -2.942337 -4.101577
C -0.667026 -0.266323 -2.484942
H 1.366466 1.272934 -4.471805
C -0.102561 0.963881 -1.905217
C 0.275088 1.075336 -0.586019
C 0.148013 -0.030752 0.334779
C -0.655005 -1.404700 -1.556968
C -0.256822 -1.299115 -0.242775
H 0.042605 1.827401 -2.554106
H 0.684976 2.026773 -0.247137
H 0.412466 0.055822 1.385659
H -0.973548 -2.383544 -1.922528
H -0.264232 -2.191438 0.385733
H -0.220327 -0.638923 -4.838514
H -1.545962 -0.149227 -3.138340
N -3.635362 2.233070 0.062763
N -4.350024 -0.598800 -0.412009
C -5.596859 3.641080 0.507371
H -5.019543 4.499610 0.138156
H -5.627159 3.727987 1.604944
H -6.628628 3.710138 0.139165
C -4.962737 2.327631 0.108677
C -5.850893 1.262819 -0.137730
H -6.909956 1.520074 -0.090337
C -5.573697 -0.111942 -0.309741
C -6.768257 -1.035048 -0.361094
H -7.546748 -0.712057 0.343582
H -6.486283 -2.072857 -0.138690
H -7.211739 -1.016304 -1.369282
C -2.830968 3.290058 0.540175
C -2.084732 4.073850 -0.375941
C -1.271241 5.096760 0.122994
H -0.701222 5.717140 -0.569978
C -1.177516 5.344260 1.489807
H -0.543224 6.154137 1.857652
C -1.869340 4.534448 2.385056
H -1.756344 4.702711 3.458615
C -2.686239 3.494241 1.935402

C -4.030846 -1.970505 -0.459489
C -4.124901 -2.720044 -1.655563
C -3.616380 -4.023738 -1.671725
H -3.684064 -4.610622 -2.591384
C -3.014551 -4.582611 -0.549858
H -2.615096 -5.598244 -0.588613
C -2.915973 -3.837576 0.623836
H -2.423105 -4.273929 1.493377
C -3.419685 -2.537747 0.692742
C -4.737011 -2.141832 -2.917082
C -3.340354 -1.731253 1.977944
C -3.325705 2.542398 2.930156
C -2.196112 3.821529 -1.869242
C -5.977047 -2.928830 -3.352202
C -3.717182 -2.070461 -4.056666
H -5.047338 -1.111169 -2.688652
H -6.465288 -2.443779 -4.212172
H -5.709484 -3.952684 -3.658227
H -6.713337 -3.009507 -2.540072
H -4.158042 -1.594273 -4.946562
H -2.830988 -1.491040 -3.764609
H -3.372485 -3.073830 -4.352368
C -2.228981 -2.178995 2.916868
C -4.692218 -1.709445 2.698538
H -3.140886 -0.677743 1.703871
H -2.142516 -1.486660 3.765856
H -2.425927 -3.177955 3.334127
H -1.253275 -2.220917 2.415989
H -4.988794 -2.731096 2.983603
H -4.635097 -1.100589 3.614505
H -5.482585 -1.288457 2.063873
C -0.991349 4.325909 -2.659770
H -2.242592 2.725178 -2.006237
C -3.496656 4.385150 -2.449393
H -3.546756 4.204188 -3.534775
H -4.377969 3.918519 -1.991250
H -3.554937 5.472438 -2.282669
H -1.030181 3.949612 -3.693220
H -0.979326 5.425764 -2.718575
H -0.038538 4.004900 -2.213170
C -4.018640 3.252181 4.092573
H -4.087255 1.956967 2.395216
C -2.274329 1.552201 3.440129
H -4.547357 2.524513 4.727625
H -3.300184 3.784136 4.735831
H -4.753452 3.989373 3.735394
H -2.729239 0.788180 4.088601
H -1.766641 1.035546 2.609992
H -1.493049 2.068515 4.018809
N 3.075048 -2.046919 0.915487
N 4.192395 0.672332 0.150847
C 4.777123 -3.429383 2.036195
H 5.865722 -3.462562 2.168553
H 4.454699 -4.323759 1.486490
H 4.305230 -3.501699 3.025462
C 4.349501 -2.168352 1.310605
C 5.346250 -1.190173 1.158704
H 6.321662 -1.466277 1.562264
C 5.225794 0.174726 0.797109
C 6.375889 1.052721 1.241321
H 7.283914 0.800513 0.671492
H 6.604459 0.874636 2.302180
H 6.154585 2.118772 1.102258
C 2.137595 -2.881137 1.566025
C 1.553946 -3.986152 0.900870
C 0.705081 -4.834083 1.620766
H 0.265482 -5.700161 1.123178
C 0.414367 -4.598007 2.962224
H -0.231767 -5.285049 3.514031
C 0.914897 -3.455874 3.580101
H 0.633816 -3.238003 4.613539
C 1.748553 -2.567398 2.893954
C 4.021659 2.034113 -0.183495
C 4.700721 2.643938 -1.278843
C 4.199538 3.865501 -1.749808
H 4.668906 4.337456 -2.612788
C 3.127512 4.511902 -1.141047
H 2.755732 5.455503 -1.547427
C 2.568306 3.976165 0.014316
H 1.768706 4.513950 0.525988
C 3.007556 2.746749 0.513855
C 6.020308 2.100720 -1.834992
C 2.454521 2.187675 1.815191
C 2.183911 -1.267032 3.553457
C 1.827933 -4.230354 -0.571383
C 6.705333 3.048103 -2.819013
C 6.005989 0.691515 -2.431320
H 6.683560 2.055892 -0.957720
H 7.690179 2.641825 -3.095210
H 6.127118 3.157221 -3.750721
H 6.864841 4.050527 -2.394938
H 7.039451 0.330656 -2.548584
H 5.472652 -0.026746 -1.800160
H 5.547090 0.684927 -3.429551
C 1.161559 2.844673 2.285845
C 3.499716 2.275963 2.933013
H 2.262550 1.114600 1.649876
H 0.769082 2.310570 3.161431
H 1.327770 3.889023 2.591623
H 0.369045 2.853896 1.526197
H 3.814155 3.320341 3.086992
H 3.081804 1.903536 3.880841
H 4.390470 1.679239 2.709381
C 0.678363 -4.932974 -1.286543
H 1.916929 -3.237272 -1.041645
C 3.150539 -4.956143 -0.825152
H 3.271786 -5.177260 -1.897309
H 4.008821 -4.347506 -0.512830
H 3.183844 -5.912839 -0.279836
H 0.835161 -4.897942 -2.374705
H 0.604973 -5.994889 -1.001825
H -0.287296 -4.455792 -1.068194
C 3.267004 -1.451730 4.619646
H 2.611899 -0.642801 2.754927
C 0.987073 -0.519113 4.145621
H 3.495660 -0.489398 5.104063

H 2.938503 -2.153224 5.403931
H 4.203044 -1.830954 4.190355
H 1.298335 0.466144 4.524105
H 0.195843 -0.362444 3.399707
H 0.538646 -1.064490 4.990576

170
B3
Ca 2.196114 -0.144560 -0.328339
Ca -2.498369 0.368003 -0.304264
C 1.500394 -0.330417 -4.133565
C 0.554319 0.648587 -4.120428
C 0.498783 1.576556 -2.939979
C 1.898028 1.878690 -2.442894
C 2.882730 0.928786 -2.607004
C 2.611969 -0.322444 -3.215518
H 3.426510 -1.034211 -3.366076
H 3.896640 1.166751 -2.276372
H 1.484858 -1.088983 -4.924489
H -0.185098 0.735913 -4.921179
C -0.383487 0.974486 -1.775403
H 2.168240 2.891737 -2.140005
C -0.032947 1.605735 -0.437293
C -0.099042 0.922667 0.765161
C -0.322605 -0.486781 0.826204
C -0.320206 -0.528718 -1.635292
C -0.356619 -1.183579 -0.421052
H 0.146572 2.678083 -0.417417
H 0.038464 1.484150 1.694359
H -0.292089 -1.030589 1.763502
H -0.331371 -1.109076 -2.557564
H -0.395025 -2.273735 -0.412792
H 0.000943 2.519607 -3.216430
H -1.424436 1.283022 -2.117893
N -4.093565 1.869906 0.449066
N -4.508201 -0.706605 -0.680918
C -6.148447 2.988276 1.190228
H -5.601852 3.938613 1.121662
H -6.243746 2.749167 2.261616
H -7.162000 3.120772 0.789487
C -5.421560 1.869798 0.481145
C -6.217279 0.850286 -0.075019
H -7.296608 1.001770 -0.029180
C -5.784004 -0.386431 -0.598237
C -6.850325 -1.353667 -1.056738
H -7.649121 -1.442148 -0.306197
H -6.434536 -2.350222 -1.257254
H -7.320272 -0.983362 -1.982018
C -3.327844 2.846207 1.113808
C -2.665204 3.837776 0.343669
C -1.821271 4.739181 0.995412
H -1.311208 5.513487 0.420743
C -1.602407 4.658553 2.369686
H -0.936776 5.372439 2.860235
C -2.221690 3.656774 3.109801
H -2.027653 3.583922 4.182922
C -3.087433 2.740049 2.504940
C -3.979089 -1.938955 -1.091926
C -3.764221 -2.215833 -2.466469
C -3.027259 -3.353741 -2.806172
H -2.842763 -3.583112 -3.856542
C -2.514281 -4.202955 -1.828070
H -1.936108 -5.082499 -2.118472
C -2.734872 -3.927989 -0.482141
H -2.313647 -4.588323 0.275005
C -3.457530 -2.799357 -0.089137
C -4.302808 -1.268937 -3.526406
C -3.698710 -2.478838 1.376028
C -3.701124 1.618410 3.323543
C -2.879071 3.897383 -1.159334
C -4.418434 -1.903392 -4.909965
C -3.478938 0.020984 -3.600722
H -5.318910 -0.981828 -3.213543
H -4.938793 -1.219028 -5.596867
H -3.429568 -2.111127 -5.348218
H -4.982631 -2.847697 -4.878784
H -3.833587 0.670414 -4.415652
H -3.587691 0.610599 -2.676780
H -2.413270 -0.190997 -3.774796
C -2.643312 -3.055453 2.311639
C -5.104342 -2.906635 1.810196
H -3.675478 -1.377368 1.476014
H -2.784443 -2.666598 3.330972
H -2.707740 -4.152618 2.371715
H -1.624289 -2.806439 1.988539
H -5.220817 -3.997459 1.710366
H -5.285297 -2.637442 2.862743
H -5.880248 -2.426150 1.201043
C -1.756059 4.602575 -1.914019
H -2.913195 2.850979 -1.519389
C -4.233737 4.513099 -1.522627
H -4.355840 4.559241 -2.616156
H -5.068145 3.926939 -1.118406
H -4.308211 5.538744 -1.127836
H -1.883829 4.464847 -2.997914
H -1.757570 5.687099 -1.722610
H -0.765938 4.212896 -1.640288
C -4.398065 2.126717 4.586424
H -4.459978 1.127417 2.697107
C -2.648538 0.559011 3.663895
H -4.921138 1.303495 5.097552
H -3.680684 2.554343 5.304461
H -5.136438 2.907930 4.351402
H -3.096194 -0.274888 4.226426
H -2.184402 0.139079 2.758540
H -1.836850 0.984125 4.275382
N 2.926291 -2.042222 1.029530
N 4.286217 0.724523 0.612993
C 4.570550 -3.577275 2.069637
H 5.075923 -3.551948 3.045096
H 5.315652 -3.914551 1.330574
H 3.770296 -4.325438 2.098336
C 4.090213 -2.195485 1.658816
C 4.996000 -1.165609 1.963881
H 5.818907 -1.470428 2.612986
C 5.071153 0.170333 1.529911

C 6.165805 0.979982 2.203432
H 7.123495 0.439612 2.200286
H 5.890674 1.133155 3.259327
H 6.311741 1.962530 1.739961
C 1.993103 -3.096094 1.115109
C 1.596938 -3.784507 -0.056109
C 0.682759 -4.837568 0.052817
H 0.391339 -5.389297 -0.841576
C 0.146025 -5.198205 1.283430
H -0.555689 -6.032822 1.355740
C 0.506365 -4.489274 2.426741
H 0.065013 -4.773885 3.382199
C 1.421044 -3.433027 2.375717
C 4.739155 1.897712 -0.031533
C 5.867825 1.824741 -0.894761
C 6.223827 2.967070 -1.617584
H 7.078691 2.931365 -2.293783
C 5.506005 4.154908 -1.501540
H 5.800919 5.031974 -2.082291
C 4.414766 4.219295 -0.642307
H 3.862667 5.155851 -0.550889
C 4.014433 3.103355 0.099543
C 6.662545 0.531125 -1.042420
C 2.837890 3.173813 1.053262
C 1.838283 -2.705981 3.651663
C 2.191052 -3.414559 -1.402565
C 8.046652 0.732353 -1.654679
C 5.898432 -0.554241 -1.805814
H 6.827778 0.131417 -0.031521
H 8.616209 -0.208484 -1.609491
H 7.989033 1.024233 -2.715442
H 8.622769 1.503398 -1.120647
H 6.498635 -1.475349 -1.862069
H 4.957008 -0.808032 -1.300038
H 5.665941 -0.233756 -2.832450
C 1.877416 4.321774 0.767706
C 3.289734 3.207313 2.515674
H 2.268470 2.238758 0.921415
H 0.965939 4.213285 1.372177
H 2.323551 5.296861 1.020468
H 1.581182 4.352763 -0.290976
H 3.908796 4.097580 2.709541
H 2.419274 3.242725 3.190546
H 3.879215 2.318387 2.770999
C 1.326141 -3.816438 -2.592300
H 2.263084 -2.313380 -1.465012
C 3.619331 -3.941194 -1.556156
H 4.043649 -3.632663 -2.524203
H 4.271727 -3.553607 -0.763352
H 3.633282 -5.041633 -1.507932
H 1.720153 -3.354843 -3.508874
H 1.319234 -4.907394 -2.746005
H 0.285237 -3.482725 -2.469973
C 1.436532 -3.435082 4.931230
H 2.937412 -2.663618 3.653735
C 1.368724 -1.250472 3.707345
H 1.865029 -2.920751 5.804876
H 0.343244 -3.451514 5.069047
H 1.796109 -4.475133 4.942491
H 1.772517 -0.756425 4.604430
H 1.715961 -0.689393 2.829975
H 0.270138 -1.187974 3.757900

170

B4*

Ca 2.321005 -0.218559 -0.340615
Ca -2.532251 0.592861 -0.779677
C 1.591349 -0.596519 -4.107864
C 0.670417 0.401462 -4.163095
C 0.650400 1.395989 -3.035683
C 2.071706 1.734549 -2.622941
C 3.015886 0.735793 -2.676351
C 2.685639 -0.559174 -3.164146
H 3.475243 -1.309775 -3.248954
H 4.038464 0.961785 -2.364481
H 1.565521 -1.407535 -4.843940
H -0.077105 0.462006 -4.955697
C -0.016560 0.805273 -1.784788
H 2.354021 2.761418 -2.382909
C 0.070365 1.553830 -0.566062
C -0.210020 0.985570 0.682135
C -0.499159 -0.394215 0.787197
C -0.159014 -0.611731 -1.621967
C -0.444676 -1.183145 -0.376674
H 0.281018 2.619967 -0.623263
H -0.234068 1.622323 1.569892
H -0.695542 -0.857947 1.749672
H -0.160278 -1.224191 -2.522687
H -0.628075 -2.255451 -0.301875
H 0.093597 2.302503 -3.300816
H -1.828949 1.228378 -2.589744
N -4.001991 2.076053 0.266766
N -4.497666 -0.597648 -0.758879
C -6.055223 3.247385 0.939386
H -5.435211 4.152872 0.979399
H -6.327001 2.984589 1.973910
H -6.988902 3.470702 0.405105
C -5.328042 2.094253 0.285451
C -6.142696 1.072406 -0.249653
H -7.216033 1.266786 -0.232499
C -5.759241 -0.211769 -0.689011
C -6.856463 -1.160226 -1.108584
H -7.790061 -0.970328 -0.562448
H -6.556948 -2.207906 -0.966693
H -7.064408 -1.022664 -2.182243
C -3.219883 3.012254 0.967460
C -2.449088 3.955330 0.232272
C -1.588448 4.806312 0.930092
H -1.001564 5.543892 0.381307
C -1.456821 4.730695 2.315948
H -0.777983 5.407554 2.840140
C -2.189580 3.784294 3.023573
H -2.072115 3.714377 4.108184
C -3.073655 2.916702 2.373214
C -4.052200 -1.906870 -1.002856
C -3.813291 -2.369797 -2.319195

C -3.188753 -3.610983 -2.492170
H -2.998104 -3.974423 -3.505197
C -2.806117 -4.386147 -1.403828
H -2.323137 -5.353242 -1.560620
C -3.033998 -3.919816 -0.109740
H -2.711292 -4.523057 0.738240
C -3.641286 -2.684129 0.116194
C -4.212000 -1.553045 -3.533425
C -3.854386 -2.150979 1.521710
C -3.800374 1.853638 3.176182
C -2.573871 4.033983 -1.279990
C -5.252411 -2.293268 -4.379466
C -2.994358 -1.152131 -4.369550
H -4.676467 -0.625445 -3.164226
H -5.610069 -1.654030 -5.201866
H -4.826748 -3.204385 -4.829580
H -6.121319 -2.597088 -3.776908
H -3.306664 -0.577409 -5.255712
H -2.306773 -0.510061 -3.795264
H -2.435843 -2.034174 -4.721223
C -2.887634 -2.738815 2.543036
C -5.301513 -2.332986 1.989742
H -3.680805 -1.061179 1.469917
H -2.982430 -2.216234 3.505768
H -3.092579 -3.803963 2.734093
H -1.841986 -2.667116 2.214859
H -5.571393 -3.401009 2.008457
H -5.431318 -1.929900 3.006715
H -6.010590 -1.812406 1.334551
C -1.416669 4.756606 -1.963294
H -2.560681 3.000189 -1.684368
C -3.907808 4.652208 -1.708649
H -3.975601 4.694771 -2.806791
H -4.761123 4.066526 -1.345460
H -4.001632 5.678768 -1.319275
H -1.493728 4.631215 -3.053212
H -1.423863 5.838474 -1.754642
H -0.438600 4.360654 -1.653734
C -4.601847 2.445067 4.337352
H -4.504754 1.346182 2.501733
C -2.815472 0.792979 3.673627
H -5.199843 1.664577 4.833448
H -3.943326 2.889271 5.100585
H -5.286570 3.234092 3.993242
H -3.338589 0.001543 4.232011
H -2.289527 0.319457 2.833162
H -2.055716 1.231114 4.340258
N 2.875630 -2.035289 1.117491
N 4.434226 0.546951 0.557993
C 4.333771 -3.691445 2.224942
H 3.841562 -3.878491 3.190748
H 5.412525 -3.839236 2.360482
H 3.952506 -4.453493 1.531573
C 4.050598 -2.296597 1.697701
C 5.081616 -1.365783 1.896467
H 5.909366 -1.727806 2.508089
C 5.219291 -0.035339 1.455338
C 6.353935 0.730877 2.107578
H 7.269442 0.124629 2.158659
H 6.066745 0.961950 3.146342
H 6.576392 1.676265 1.599009
C 1.858804 -2.996562 1.321025
C 1.394714 -3.779183 0.238158
C 0.423504 -4.756322 0.482863
H 0.070273 -5.380805 -0.338167
C -0.098357 -4.945651 1.757688
H -0.845251 -5.722826 1.935636
C 0.322458 -4.131422 2.806540
H -0.116569 -4.274175 3.794702
C 1.292615 -3.143349 2.616161
C 4.879551 1.724947 -0.081927
C 5.996194 1.665846 -0.961342
C 6.329665 2.816579 -1.681556
H 7.175692 2.794280 -2.369173
C 5.600213 3.995696 -1.550043
H 5.878809 4.879036 -2.129092
C 4.517025 4.043758 -0.679667
H 3.955374 4.973394 -0.579443
C 4.138187 2.919311 0.060670
C 6.801241 0.380924 -1.129566
C 2.964278 2.970095 1.021467
C 1.742731 -2.257879 3.774545
C 1.952555 -3.572321 -1.159405
C 8.171820 0.599840 -1.765902
C 6.036482 -0.711239 -1.883024
H 6.990695 -0.020209 -0.123662
H 8.751368 -0.335325 -1.736279
H 8.091707 0.896577 -2.823762
H 8.750079 1.373464 -1.238096
H 6.645283 -1.626125 -1.946223
H 5.103838 -0.974541 -1.365431
H 5.788984 -0.394767 -2.907345
C 2.014207 4.135555 0.776364
C 3.417290 2.949635 2.483906
H 2.376438 2.045438 0.869394
H 1.100954 4.019473 1.376574
H 2.472735 5.094860 1.063423
H 1.719463 4.210697 -0.280328
H 4.045173 3.827415 2.702756
H 2.547854 2.972945 3.159971
H 3.997873 2.046962 2.709277
C 1.016648 -4.039322 -2.270515
H 2.072198 -2.485092 -1.323435
C 3.346567 -4.179870 -1.329924
H 3.715704 -4.011629 -2.353544
H 4.069075 -3.730379 -0.636227
H 3.321418 -5.266301 -1.149699
H 1.380994 -3.671460 -3.240701
H 0.968798 -5.138307 -2.330270
H -0.009335 -3.667328 -2.132433
C 1.467086 -2.865032 5.148022
H 2.832104 -2.133571 3.694614
C 1.165304 -0.843303 3.692377
H 1.933307 -2.249758 5.932526
H 0.389027 -2.909612 5.370842
H 1.871429 -3.885392 5.232322

H 1.556876 -0.221284 4.512212
H 1.443947 -0.358646 2.747188
H 0.066945 -0.852075 3.773720

170

B5

Ca 3.272001 -0.085267 0.178492
Ca -2.796174 -0.086697 0.597135
C 2.132500 -1.416133 2.200723
C 1.049216 -1.232614 1.363924
C 0.821242 -2.226358 0.246144
C 2.188660 -2.526356 -0.352817
C 3.255766 -2.730227 0.507558
C 3.243790 -2.222322 1.827692
H 4.088186 -2.381808 2.496172
H 4.182440 -3.168813 0.125311
H 2.176272 -0.842572 3.133436
H 0.229624 -0.568870 1.668240
C -0.133528 -1.773546 -0.827175
H 2.239339 -2.868753 -1.390451
C -0.707545 -2.741576 -1.664465
C -1.506303 -2.379884 -2.743483
C -1.765129 -1.031452 -3.003883
C -0.410101 -0.422774 -1.085594
C -1.223035 -0.055278 -2.169545
H -0.521661 -3.796550 -1.455217
H -1.938410 -3.154709 -3.378810
H -2.385488 -0.741706 -3.853199
H 0.033850 0.355070 -0.455231
H -1.410772 1.001736 -2.358326
H 0.376138 -3.168506 0.648303
H -1.710583 0.350767 2.270400
N -4.385124 -1.800197 0.869456
N -4.807955 1.134123 0.474606
C -6.427525 -2.943849 1.652583
H -5.792753 -3.837270 1.696056
H -7.237638 -3.129563 0.931150
H -6.899844 -2.795404 2.634938
C -5.645756 -1.703903 1.270315
C -6.365854 -0.495273 1.357361
H -7.388133 -0.594965 1.726213
C -6.012747 0.795774 0.920895
C -7.118542 1.827996 0.977101
H -8.097543 1.361358 0.805718
H -6.967189 2.628900 0.241400
H -7.147644 2.299377 1.971963
C -3.815965 -3.044673 0.530013
C -2.755606 -3.563843 1.322101
C -2.113357 -4.728680 0.890589
H -1.300476 -5.144784 1.486858
C -2.494449 -5.375591 -0.283562
H -1.974203 -6.282437 -0.601835
C -3.546504 -4.869561 -1.040129
H -3.850616 -5.388710 -1.952864
C -4.229188 -3.713007 -0.650817
C -4.539113 2.442200 0.024264
C -4.335672 3.504222 0.939793
C -3.976599 4.760709 0.438802
H -3.809223 5.584564 1.136493
C -3.816289 4.979325 -0.925410
H -3.529121 5.966655 -1.293971
C -4.010509 3.928490 -1.819956
H -3.880648 4.109785 -2.887913
C -4.367424 2.653783 -1.370384
C -4.477674 3.305962 2.438863
C -4.618306 1.514118 -2.344654
C -5.398462 -3.212124 -1.485180
C -2.383923 -2.902693 2.642391
C -5.383681 4.365534 3.071137
C -3.112822 3.257832 3.130805
H -4.943608 2.324188 2.601012
H -5.582424 4.119805 4.125881
H -4.917717 5.363506 3.052513
H -6.349128 4.441218 2.548172
H -3.238318 3.174441 4.222054
H -2.534629 2.377919 2.802738
H -2.529711 4.170298 2.925650
C -3.986034 1.726648 -3.716494
C -6.115356 1.224227 -2.498157
H -4.170791 0.604869 -1.903292
H -4.097789 0.818968 -4.328112
H -4.477771 2.544004 -4.266774
H -2.914474 1.967411 -3.651039
H -6.646155 2.118790 -2.860490
H -6.277779 0.412181 -3.223675
H -6.567000 0.914436 -1.548006
C -1.063183 -3.382288 3.235812
H -2.265974 -1.811819 2.478342
C -3.498451 -3.065245 3.682121
H -3.197069 -2.601663 4.634051
H -4.431076 -2.586246 3.363216
H -3.701549 -4.131999 3.869670
H -0.810521 -2.766310 4.110984
H -1.125736 -4.429064 3.575394
H -0.228039 -3.297695 2.528805
C -6.428586 -4.315754 -1.737116
H -5.898523 -2.415671 -0.919410
C -4.947149 -2.574827 -2.798779
H -7.326629 -3.902854 -2.223146
H -6.030640 -5.101486 -2.398735
H -6.737545 -4.799189 -0.798677
H -5.815665 -2.212726 -3.371367
H -4.282675 -1.721273 -2.611827
H -4.403604 -3.294493 -3.431476
N 3.251206 2.205103 -0.298421
N 5.527236 0.383608 -0.310515
C 3.821130 4.533036 -0.888441
H 4.058198 4.836976 -1.919583
H 4.425511 5.168190 -0.223035
H 2.760182 4.736732 -0.697883
C 4.175710 3.078508 -0.663077
C 5.532439 2.743770 -0.839593
H 6.186295 3.566535 -1.132388
C 6.160293 1.496364 -0.667312
C 7.657162 1.466597 -0.886778
H 8.188410 1.552836 0.074468

H 7.979530 2.301170 -1.522563
H 7.979366 0.519266 -1.339933
C 1.883524 2.519232 -0.291444
C 1.197650 2.548203 0.947540
C -0.178190 2.793300 0.947509
H -0.723940 2.804094 1.887281
C -0.862806 3.035408 -0.239045
H -1.926599 3.271975 -0.218443
C -0.183102 2.981317 -1.455040
H -0.741502 3.170400 -2.372559
C 1.179417 2.678683 -1.516325
C 6.258128 -0.795328 -0.059817
C 6.937290 -0.956725 1.172187
C 7.563960 -2.177813 1.433767
H 8.087207 -2.322219 2.381544
C 7.533073 -3.217453 0.507480
H 8.033024 -4.163343 0.728582
C 6.854683 -3.052278 -0.697565
H 6.825978 -3.878361 -1.410041
C 6.198821 -1.854646 -0.998030
C 6.936932 0.157565 2.206426
C 5.416422 -1.679722 -2.289627
C 1.895272 2.476416 -2.844736
C 1.957871 2.366513 2.249691
C 8.269688 0.292861 2.941563
C 5.779095 -0.002560 3.194739
H 6.757492 1.102345 1.675135
H 8.266559 1.191124 3.578174
H 8.468485 -0.568137 3.598965
H 9.111077 0.375756 2.236589
H 5.756056 0.832199 3.912596
H 4.808666 -0.025755 2.677116
H 5.864229 -0.943154 3.761343
C 4.958368 -2.994259 -2.914731
C 6.171842 -0.830471 -3.315800
H 4.503553 -1.110739 -2.033879
H 4.262534 -2.794989 -3.743682
H 5.804532 -3.563168 -3.331399
H 4.442976 -3.638664 -2.187754
H 7.139940 -1.293512 -3.564197
H 5.589016 -0.739776 -4.246042
H 6.360204 0.183686 -2.941704
C 1.070993 1.943078 3.417050
H 2.702974 1.561513 2.094895
C 2.765472 3.620341 2.599843
H 3.321255 3.473066 3.539275
H 3.489359 3.868462 1.813120
H 2.091791 4.481274 2.733270
H 1.692092 1.629090 4.269768
H 0.446861 2.781527 3.764417
H 0.388286 1.122590 3.148657
C 1.061956 2.890851 -4.054040
H 2.796786 3.109541 -2.843476
C 2.377874 1.026619 -3.008055
H 1.661990 2.806723 -4.972492
H 0.179613 2.243348 -4.180609
H 0.713063 3.931369 -3.974591
H 2.790724 0.863877 -4.014986
H 3.194700 0.808773 -2.304012
H 1.557443 0.309493 -2.855999

170

B6*

Ca 2.321005 -0.218559 -0.340615
Ca -2.532251 0.592861 -0.779677
C 1.591349 -0.596519 -4.107864
C 0.670418 0.401461 -4.163094
C 0.650401 1.395989 -3.035683
C 2.071706 1.734549 -2.622940
C 3.015886 0.735793 -2.676351
C 2.685639 -0.559174 -3.164146
H 3.475243 -1.309775 -3.248953
H 4.038464 0.961785 -2.364481
H 1.565521 -1.407535 -4.843939
H -0.077105 0.462006 -4.955697
C -0.016559 0.805273 -1.784787
H 2.354020 2.761418 -2.382908
C 0.070365 1.553830 -0.566062
C -0.210020 0.985570 0.682135
C -0.499158 -0.394215 0.787196
C -0.159014 -0.611731 -1.621967
C -0.444676 -1.183145 -0.376674
H 0.281019 2.619967 -0.623263
H -0.234067 1.622324 1.569891
H -0.695543 -0.857946 1.749672
H -0.160278 -1.224191 -2.522687
H -0.628075 -2.255451 -0.301875
H 0.093598 2.302502 -3.300817
H -1.828949 1.228378 -2.589744
N -4.001992 2.076053 0.266766
N -4.497666 -0.597647 -0.758880
C -6.055222 3.247386 0.939386
H -5.435210 4.152872 0.979399
H -6.327001 2.984590 1.973910
H -6.988902 3.470703 0.405105
C -5.328042 2.094254 0.285450
C -6.142696 1.072406 -0.249653
H -7.216033 1.266786 -0.232498
C -5.759242 -0.211769 -0.689012
C -6.856464 -1.160226 -1.108585
H -7.790061 -0.970328 -0.562448
H -6.556948 -2.207906 -0.966692
H -7.064407 -1.022664 -2.182243
C -3.219883 3.012254 0.967460
C -2.449088 3.955330 0.232272
C -1.588449 4.806312 0.930092
H -1.001564 5.543892 0.381306
C -1.456821 4.730695 2.315948
H -0.777983 5.407554 2.840140
C -2.189580 3.784293 3.023574
H -2.072115 3.714377 4.108183
C -3.073654 2.916701 2.373214
C -4.052200 -1.906870 -1.002856
C -3.813292 -2.369797 -2.319194
C -3.188753 -3.610983 -2.492170
H -2.998103 -3.974423 -3.505197

C -2.806117 -4.386148 -1.403828
H -2.323137 -5.353242 -1.560620
C -3.033998 -3.919816 -0.109740
H -2.711292 -4.523056 0.738240
C -3.641286 -2.684129 0.116194
C -4.212000 -1.553045 -3.533425
C -3.854386 -2.150979 1.521710
C -3.800375 1.853638 3.176182
C -2.573871 4.033983 -1.279990
C -5.252412 -2.293268 -4.379466
C -2.994359 -1.152130 -4.369550
H -4.676467 -0.625445 -3.164227
H -5.610069 -1.654030 -5.201866
H -4.826748 -3.204385 -4.829581
H -6.121319 -2.597088 -3.776908
H -3.306664 -0.577410 -5.255712
H -2.306773 -0.510061 -3.795264
H -2.435843 -2.034175 -4.721223
C -2.887634 -2.738815 2.543036
C -5.301512 -2.332986 1.989742
H -3.680806 -1.061179 1.469917
H -2.982430 -2.216233 3.505768
H -3.092580 -3.803963 2.734093
H -1.841987 -2.667115 2.214859
H -5.571392 -3.401009 2.008458
H -5.431318 -1.929900 3.006715
H -6.010591 -1.812406 1.334551
C -1.416669 4.756607 -1.963294
H -2.560681 3.000189 -1.684368
C -3.907807 4.652208 -1.708648
H -3.975601 4.694770 -2.806791
H -4.761122 4.066526 -1.345460
H -4.001631 5.678768 -1.319275
H -1.493728 4.631215 -3.053213
H -1.423863 5.838473 -1.754642
H -0.438601 4.360654 -1.653734
C -4.601847 2.445067 4.337352
H -4.504754 1.346182 2.501733
C -2.815471 0.792979 3.673627
H -5.199844 1.664577 4.833447
H -3.943326 2.889271 5.100584
H -5.286570 3.234092 3.993242
H -3.338588 0.001544 4.232011
H -2.289527 0.319456 2.833162
H -2.055715 1.231114 4.340258
N 2.875630 -2.035289 1.117492
N 4.434226 0.546951 0.557993
C 4.333771 -3.691445 2.224941
H 3.841562 -3.878491 3.190749
H 5.412525 -3.839236 2.360481
H 3.952506 -4.453492 1.531574
C 4.050598 -2.296597 1.697702
C 5.081616 -1.365783 1.896468
H 5.909366 -1.727807 2.508090
C 5.219291 -0.035339 1.455338
C 6.353936 0.730877 2.107578
H 7.269442 0.124629 2.158660
H 6.066745 0.961950 3.146342
H 6.576392 1.676265 1.599009
C 1.858803 -2.996561 1.321025
C 1.394715 -3.779183 0.238159
C 0.423504 -4.756322 0.482863
H 0.070273 -5.380806 -0.338167
C -0.098357 -4.945651 1.757688
H -0.845250 -5.722826 1.935637
C 0.322458 -4.131421 2.806540
H -0.116569 -4.274175 3.794702
C 1.292615 -3.143349 2.616161
C 4.879551 1.724947 -0.081927
C 5.996194 1.665846 -0.961342
C 6.329665 2.816579 -1.681557
H 7.175693 2.794280 -2.369173
C 5.600213 3.995696 -1.550044
H 5.878809 4.879036 -2.129092
C 4.517025 4.043758 -0.679667
H 3.955374 4.973394 -0.579443
C 4.138187 2.919311 0.060669
C 6.801241 0.380924 -1.129566
C 2.964278 2.970096 1.021467
C 1.742731 -2.257879 3.774545
C 1.952554 -3.572321 -1.159405
C 8.171819 0.599840 -1.765901
C 6.036482 -0.711239 -1.883023
H 6.990695 -0.020209 -0.123662
H 8.751368 -0.335325 -1.736279
H 8.091707 0.896577 -2.823762
H 8.750080 1.373464 -1.238096
H 6.645283 -1.626125 -1.946223
H 5.103838 -0.974541 -1.365431
H 5.788985 -0.394768 -2.907345
C 2.014207 4.135555 0.776364
C 3.417290 2.949635 2.483906
H 2.376438 2.045438 0.869393
H 1.100955 4.019474 1.376574
H 2.472735 5.094860 1.063423
H 1.719463 4.210697 -0.280329
H 4.045173 3.827415 2.702756
H 2.547853 2.972946 3.159971
H 3.997873 2.046963 2.709277
C 1.016648 -4.039322 -2.270515
H 2.072199 -2.485092 -1.323435
C 3.346567 -4.179870 -1.329924
H 3.715704 -4.011629 -2.353544
H 4.069075 -3.730380 -0.636226
H 3.321418 -5.266301 -1.149699
H 1.380994 -3.671460 -3.240701
H 0.968798 -5.138308 -2.330270
H -0.009335 -3.667329 -2.132432
C 1.467085 -2.865031 5.148022
H 2.832104 -2.133571 3.694614
C 1.165303 -0.843302 3.692377
H 1.933308 -2.249758 5.932526
H 0.389027 -2.909612 5.370842
H 1.871428 -3.885392 5.232322
H 1.556876 -0.221284 4.512212
H 1.443946 -0.358646 2.747188

H 0.066945 -0.852075 3.773720

Calculations with phenyl-substituents

86
A1
Ca 2.188076 -0.035296 0.256611
Ca -2.188153 0.035223 0.257477
N 3.825012 1.619287 0.140482
N 4.129571 -1.265952 -0.041097
N -3.825112 -1.619251 0.140609
N -4.129496 1.265960 -0.041091
C 0.018005 0.757092 -0.957036
C -0.018651 -0.757186 -0.957042
C -0.023737 -1.433018 0.275464
C -0.006339 -0.757655 1.508895
C 0.006865 0.757484 1.508909
C 0.023626 1.432872 0.275490
C 5.907989 2.877169 -0.076258
H 5.554585 3.516774 -0.899878
H 6.985119 2.709583 -0.198325
H 5.738854 3.446463 0.850983
C 5.136836 1.578933 -0.045356
C 5.869830 0.384407 -0.221675
H 6.944116 0.508171 -0.361961
C 5.396237 -0.941093 -0.268171
C 6.403751 -2.025439 -0.572782
H 6.207581 -2.923756 0.031333
H 7.427601 -1.679176 -0.381052
H 6.347169 -2.331963 -1.629295
C 3.121543 2.830356 0.266301
C 2.777462 3.323980 1.536660
C 1.945753 4.436504 1.667001
H 1.687854 4.804137 2.663506
C 1.435322 5.071457 0.533812
H 0.777332 5.936824 0.637051
C 1.769406 4.589301 -0.733683
H 1.364201 5.072412 -1.626114
C 2.604506 3.481109 -0.868404
C 3.594441 -2.542178 -0.241868
C 2.949065 -3.192188 0.830604
C 2.235771 -4.373697 0.625849
H 1.730766 -4.849625 1.469683
C 2.160208 -4.941642 -0.644821
H 1.597602 -5.863682 -0.805145
C 2.809538 -4.316641 -1.714191
H 2.750677 -4.749747 -2.716214
C 3.511492 -3.131026 -1.520777
C -5.908052 -2.877105 -0.076595
H -5.553920 -3.517167 -0.899534
H -6.985065 -2.709530 -0.199713
H -5.739810 -3.445907 0.851120
C -5.136882 -1.578886 -0.045571
C -5.869793 -0.384341 -0.222010
H -6.944055 -0.508051 -0.362534
C -5.396133 0.941132 -0.268369
C -6.403568 2.025533 -0.573076
H -6.207625 2.923682 0.031362
H -7.427478 1.679203 -0.381786
H -6.346621 2.332358 -1.629479
C -3.594345 2.542170 -0.241819
C -3.511176 3.130922 -1.520758
C -2.809190 4.316522 -1.714139
H -2.750160 4.749551 -2.716186
C -2.160041 4.941606 -0.644708
H -1.597398 5.863626 -0.805006
C -2.235826 4.373759 0.625993
H -1.730973 4.849751 1.469881
C -2.949165 3.192269 0.830711
H 0.044070 2.526020 0.272959
H 0.044463 1.313345 2.445307
H 0.015343 1.314045 -1.893243
H -0.016792 -1.314101 -1.893273
H -0.044279 -2.526160 0.272882
H -0.043412 -1.313538 2.445302
C -3.121643 -2.830312 0.266415
C -2.604576 -3.481064 -0.868284
C -1.769468 -4.589242 -0.733549
H -1.364239 -5.072346 -1.625972
C -1.435384 -5.071386 0.533957
H -0.777379 -5.936740 0.637206
C -1.945839 -4.436439 1.667135
H -1.687948 -4.804062 2.663644
C -2.777572 -3.323929 1.536780
H 3.019226 -2.752240 1.829245
H -3.979578 2.621367 -2.365895
H -2.852892 -3.085490 -1.855851
H -3.168855 -2.812494 2.419635
H -3.019524 2.752422 1.829386
H 3.980040 -2.621533 -2.365872
H 2.852844 3.085543 -1.855969
H 3.168698 2.812515 2.419517

86
A2*
Ca -1.811545 -0.264719 -0.422944
Ca 2.000978 -1.255884 0.441129
N -1.891627 1.891245 -1.425006
N -4.003424 -0.147319 -1.213290
N 3.930342 0.048936 0.437830
N 1.608407 0.200439 2.279580
C -2.525677 3.778887 -2.874995
H -2.962023 4.594430 -2.276814
H -2.985420 3.825611 -3.871695
H -1.448786 3.976690 -2.965936
C -2.789826 2.430404 -2.238554
C -4.022764 1.825722 -2.578087
H -4.621585 2.372511 -3.308714
C -4.639255 0.690489 -2.021230
C -6.079344 0.439516 -2.409992
H -6.248554 0.741929 -3.452922
H -6.769138 1.031017 -1.786893
H -6.349054 -0.619602 -2.297032
C -0.826775 2.627171 -0.888125
C 0.487741 2.124560 -0.983829
C 1.536103 2.724107 -0.287283

H 2.535538 2.289031 -0.336510
C 1.309487 3.856617 0.495949
H 2.134322 4.313543 1.046349
C 0.018072 4.386582 0.573306
H -0.174330 5.271966 1.185147
C -1.038427 3.776792 -0.101691
C -4.592906 -1.094847 -0.391390
C -3.949640 -2.347745 -0.256659
C -4.357289 -3.266142 0.710265
H -3.835338 -4.222942 0.791679
C -5.426102 -2.973317 1.556960
H -5.746688 -3.691377 2.314765
C -6.086785 -1.747422 1.419703
H -6.924481 -1.505403 2.079477
C -5.679266 -0.818349 0.467992
C 5.739840 1.592921 0.985603
H 6.512104 0.808500 0.972687
H 6.009158 2.344914 1.737183
H 5.761678 2.063138 -0.010013
C 4.380573 0.991696 1.250416
C 3.646055 1.501922 2.343131
H 4.146665 2.288003 2.909704
C 2.331998 1.207690 2.759092
C 1.768354 2.113387 3.831568
H 1.312045 3.009036 3.380809
H 2.570015 2.456361 4.499800
H 0.996685 1.608968 4.429044
C 0.234411 0.095337 2.468077
C -0.324946 -1.159090 2.804533
C -1.708244 -1.350943 2.828642
H -2.112868 -2.337587 3.065995
C -2.583484 -0.295516 2.552549
H -3.663777 -0.454930 2.564109
C -2.041420 0.967800 2.253193
H -2.703842 1.809464 2.032394
C -0.660868 1.162445 2.205117
C -0.118693 -1.915810 -1.005353
C 4.648655 -0.382667 -0.691782
C 5.499097 -1.499296 -0.618554
C 6.082356 -2.024379 -1.770478
H 6.735901 -2.897141 -1.694457
C 5.827296 -1.449386 -3.016905
H 6.278024 -1.868178 -3.919410
C 4.985818 -0.338541 -3.100401
H 4.774379 0.116477 -4.071346
C 4.400510 0.190530 -1.951469
H -3.128394 -2.600921 -0.938917
H 0.347209 -1.990128 3.034988
H 5.680671 -1.956351 0.357292
H 3.725966 1.047479 -2.017132
H -0.258070 2.131217 1.911254
H -6.169926 0.154612 0.406440
H -2.054576 4.162844 0.004549
H 0.671691 1.249605 -1.612101
C 0.978530 -1.979271 -1.969028
C 2.103362 -2.776645 -1.814365
C 2.244975 -3.653543 -0.703451
C -0.011165 -2.995092 -0.021960
C 1.111803 -3.806222 0.128149
H 0.944692 -1.323332 -2.848035
H 2.906876 -2.724838 -2.556129
H 3.109509 -4.312585 -0.615310
H -0.857224 -3.190854 0.648496
H 1.114806 -4.583160 0.901597
H 0.218622 -0.591167 -0.243861

86

A3

Ca -1.619492 -0.302223 -0.543306
Ca 1.619325 0.302901 -0.542229
N -1.348819 -2.641835 -0.736447
N -3.818088 -1.091455 -0.102147
N 3.818155 1.091392 -0.100928
N 1.349130 2.642592 -0.734238
H 0.000306 0.001049 -1.929194
C -1.677806 -5.061305 -0.730430
H -1.254205 -5.208950 -1.735821
H -2.468167 -5.803985 -0.567287
H -0.859560 -5.252152 -0.018806
C -2.191611 -3.649147 -0.572796
C -3.556352 -3.498901 -0.248868
H -4.113849 -4.430091 -0.149356
C -4.299896 -2.328223 -0.011373
C -5.750286 -2.514440 0.371522
H -5.938072 -2.117990 1.381350
H -6.042720 -3.571175 0.351108
H -6.406159 -1.949280 -0.308558
C 0.001833 -2.865163 -1.052299
C 0.979612 -2.891442 -0.038928
C 2.341625 -2.908674 -0.361988
H 3.095328 -2.908978 0.430878
C 2.745946 -2.927070 -1.701553
H 3.808123 -2.938420 -1.947503
C 1.779554 -2.945571 -2.711804
H 2.086605 -2.963481 -3.760503
C 0.425080 -2.909593 -2.392366
C -4.630364 0.009083 0.226229
C -4.722917 0.463980 1.555243
C -5.426794 1.626605 1.864246
H -5.480152 1.965667 2.902070
C -6.060495 2.357771 0.856671
H -6.608952 3.270676 1.099047
C -5.989827 1.907076 -0.462593
H -6.489951 2.466526 -1.257645
C -5.279227 0.748967 -0.776747
C 5.751042 2.513739 0.371748
H 5.939124 2.116747 1.381308
H 6.043708 3.570416 0.351701
H 6.406530 1.948757 -0.308852
C 4.300459 2.327995 -0.010632
C 3.557249 3.498916 -0.248053
H 4.115138 4.429913 -0.148914
C 2.192435 3.649623 -0.571430
C 1.679134 5.061937 -0.729289
H 1.255938 5.209711 -1.734830
H 2.469684 5.804369 -0.565925

H 0.860697 5.253056 -0.017956
C -0.001460 2.866295 -1.050069
C -0.979363 2.891256 -0.036777
C -2.341342 2.908825 -0.359988
H -3.095151 2.908131 0.432774
C -2.745493 2.928861 -1.699582
H -3.807640 2.940487 -1.945645
C -1.778978 2.948634 -2.709694
H -2.085906 2.967792 -3.758408
C -0.424544 2.912323 -2.390136
C -0.000508 -0.000804 1.403661
C 4.630228 -0.009394 0.227172
C 4.721972 -0.465087 1.555959
C 5.425591 -1.627956 1.864667
H 5.478332 -1.967648 2.902315
C 6.059822 -2.358549 0.857016
H 6.608071 -3.271642 1.099152
C 5.989956 -1.907049 -0.462020
H 6.490476 -2.466071 -1.257124
C 5.279621 -0.748700 -0.775884
H -4.225789 -0.112505 2.338821
H -0.652110 2.858855 1.003627
H 4.224439 0.110980 2.339586
H 5.212036 -0.397415 -1.808503
H 0.332081 2.870265 -3.175933
H -5.211066 0.398280 -1.809533
H -0.331456 -2.866622 -3.178199
H 0.652243 -2.860290 1.001478
C 1.118905 -0.376533 2.171360
C 1.141381 -0.375043 3.568837
C -0.001090 -0.001876 4.276650
C -1.120242 0.374267 2.171200
C -1.143295 0.371782 3.568658
H 2.054232 -0.714305 1.692109
H 2.046927 -0.674343 4.104906
H -0.001297 -0.002254 5.369636
H -2.055373 0.712392 1.691814
H -2.049068 0.670651 4.104581

98

A4

Ca 1.879980 0.150369 -0.456914
Ca -1.394268 -0.070296 -0.410398
N 1.579155 2.481511 -0.995086
N 4.106474 0.956882 -0.376613
N -3.395206 -0.545088 0.792936
N -1.136658 -2.403312 0.031428
H 0.256792 -0.125734 -1.808031
C 1.964860 4.877717 -1.294178
H 1.434591 4.914851 -2.258100
H 2.789944 5.599820 -1.315959
H 1.238389 5.194837 -0.530100
C 2.454641 3.475151 -1.010249
C 3.838205 3.335081 -0.766338
H 4.406656 4.263869 -0.808817
C 4.597187 2.189364 -0.466479
C 6.071660 2.407663 -0.211961
H 6.353591 2.007150 0.774265
H 6.335443 3.471350 -0.251475
H 6.682726 1.866379 -0.950228
C 0.216187 2.756848 -1.198914
C -0.633727 3.043852 -0.111705
C -2.011616 3.195216 -0.301545
H -2.659150 3.394359 0.554625
C -2.563692 3.084370 -1.580986
H -3.639419 3.205932 -1.720603
C -1.724083 2.828135 -2.670232
H -2.144783 2.751971 -3.676269
C -0.354068 2.652977 -2.481535
C 4.956617 -0.113153 -0.043665
C 4.974858 -0.612915 1.270400
C 5.751280 -1.724153 1.598109
H 5.754042 -2.095725 2.626173
C 6.521095 -2.360934 0.623134
H 7.127953 -3.231751 0.881070
C 6.503954 -1.876811 -0.687872
H 7.097916 -2.372007 -1.460540
C 5.726035 -0.769591 -1.020776
C -5.274297 -1.719643 1.822829
H -5.221795 -1.152853 2.765449
H -5.610875 -2.739763 2.043914
H -6.036513 -1.218785 1.206213
C -3.930194 -1.708475 1.131229
C -3.324707 -2.964347 0.912006
H -3.926367 -3.821210 1.215775
C -2.015278 -3.280280 0.490519
C -1.613417 -4.735290 0.584789
H -1.112564 -5.057105 -0.341035
H -2.480101 -5.382265 0.769646
H -0.890977 -4.889529 1.400961
C 0.217950 -2.747558 -0.102159
C 1.045687 -2.856840 1.034628
C 2.426207 -3.002329 0.904754
H 3.051175 -3.050857 1.798582
C 3.020048 -3.058071 -0.361447
H 4.103346 -3.147294 -0.455632
C 2.205819 -2.989206 -1.496749
H 2.653411 -3.040849 -2.492818
C 0.821367 -2.836576 -1.371192
C 0.327662 0.321689 1.557318
C -4.005063 0.663126 1.181032
C -3.568865 1.326985 2.342075
C -4.094012 2.570622 2.690059
H -3.738366 3.068906 3.595517
C -5.057721 3.183532 1.885943
H -5.463684 4.160665 2.157104
C -5.493214 2.535776 0.728089
H -6.245374 3.005534 0.088807
C -4.972423 1.290872 0.377528
H 4.365344 -0.113670 2.028491
H 0.594336 -2.768471 2.022967
H -2.797501 0.857894 2.955327
H -5.308037 0.788840 -0.531038
H 0.188690 -2.744036 -2.251467
H 5.697711 -0.395558 -2.047124
H 0.300342 2.407879 -3.319549

H -0.204136 3.113981 0.888235
C -0.408189 -0.367970 2.550924
C -0.168523 -0.229794 3.920814
C 0.854431 0.606003 4.375303
C 1.337471 1.165923 2.074896
C 1.614723 1.305696 3.440904
H -1.208488 -1.057287 2.252606
H -0.777128 -0.787876 4.639611
H 1.054142 0.709261 5.445333
H 1.954017 1.784055 1.401726
H 2.418038 1.971875 3.770206
C -2.443048 -0.535357 -3.474574
C -3.645917 -0.312002 -2.796974
C -4.333548 -1.387344 -2.223986
C -3.824257 -2.680983 -2.328415
C -1.934842 -1.833512 -3.578459
C -2.622559 -2.902686 -3.005189
H -4.044043 0.702072 -2.714020
H -5.267953 -1.216915 -1.686269
H -4.356004 -3.513430 -1.865881
H -0.991422 -1.998572 -4.102760
H -2.215983 -3.914288 -3.074913
H -1.892622 0.298281 -3.912610

98
A5*
Ca -2.497616 0.048970 0.911482
Ca 2.716594 -0.139304 1.001416
N -3.945813 -1.615097 0.235683
N -4.205606 1.289891 -0.104782
N 3.708935 1.647681 -0.187253
N 3.722085 -1.286066 -0.785461
C -0.552823 -0.710408 -1.579514
C -0.740994 0.617552 -1.990029
C -0.490960 1.648716 -1.077825
C -0.071526 1.368893 0.223731
C 0.128169 0.026173 0.663541
C -0.135322 -1.009655 -0.282861
C -5.893249 -2.892917 -0.543885
H -5.625561 -3.282752 -1.538698
H -6.981427 -2.748846 -0.527616
H -5.619654 -3.664280 0.190408
C -5.174095 -1.594227 -0.262110
C -5.876092 -0.403731 -0.538340
H -6.908869 -0.535018 -0.864128
C -5.410959 0.927064 -0.532631
C -6.387769 1.968383 -1.034799
H -6.191565 2.953754 -0.590500
H -7.417216 1.666889 -0.798342
H -6.324985 2.079834 -2.128775
C -3.167014 -2.772112 0.339097
C -2.631122 -3.127668 1.592178
C -1.713654 -4.173941 1.704361
H -1.312549 -4.431983 2.687267
C -1.309042 -4.880769 0.573816
H -0.580827 -5.688656 0.660226
C -1.835275 -4.536046 -0.675141
H -1.511995 -5.074238 -1.568995
C -2.750886 -3.496994 -0.795577
C -3.669938 2.561451 -0.334320
C -3.077145 3.259451 0.737620
C -2.378974 4.446958 0.522462
H -1.916356 4.958914 1.369250
C -2.272595 4.980103 -0.762351
H -1.728020 5.911466 -0.930556
C -2.869773 4.306906 -1.832322
H -2.786924 4.710767 -2.844881
C -3.549347 3.109627 -1.626940
C 5.422691 2.872450 -1.455920
H 4.919301 3.287021 -2.343180
H 6.470877 2.689242 -1.726891
H 5.380081 3.641678 -0.671499
C 4.757038 1.591158 -1.002047
C 5.314209 0.394399 -1.499648
H 6.226661 0.517983 -2.084321
C 4.772086 -0.907372 -1.507114
C 5.451964 -1.911099 -2.412144
H 5.495174 -2.902708 -1.937977
H 6.469292 -1.586106 -2.665245
H 4.894394 -2.032410 -3.353775
C 3.074473 -2.505302 -1.040575
C 2.349133 -2.712474 -2.228306
C 1.636908 -3.891579 -2.430291
H 1.081372 -4.033934 -3.361179
C 1.622174 -4.886480 -1.449506
H 1.067305 -5.812963 -1.612210
C 2.320444 -4.683699 -0.259194
H 2.313026 -5.451007 0.519001
C 3.038340 -3.506109 -0.054606
H 0.008706 -2.054461 0.003207
H -1.892557 0.175381 2.951202
H -0.734761 -1.534911 -2.272144
H -1.090847 0.844190 -2.998471
H -0.637364 2.690252 -1.366933
H 0.139137 2.197066 0.902784
C 3.013534 2.849972 0.015526
C 2.330421 3.485742 -1.037921
C 1.520077 4.591488 -0.797711
H 0.985161 5.058895 -1.628342
C 1.365373 5.088520 0.499213
H 0.721449 5.950428 0.686672
C 2.045172 4.473316 1.551253
H 1.939597 4.854665 2.570073
C 2.862603 3.368522 1.313196
H -3.174999 2.858548 1.752190
H 2.337570 -1.919297 -2.979161
H 2.409734 3.065891 -2.042987
H 3.394508 2.887974 2.136924
H 3.595585 -3.350959 0.872344
H -3.965592 2.561485 -2.474038
H -3.130820 -3.203434 -1.776378
H -2.948866 -2.575688 2.482429
C 0.915302 -1.644088 2.414079
C 0.380893 -0.277468 2.103204
C 1.071895 0.785474 2.914108
C 2.252169 0.539638 3.572253

C 2.110365 -1.818689 3.067207
C 2.918559 -0.724399 3.517234
H -0.875718 -0.133912 2.580416
H 0.572119 1.749227 3.041587
H 2.706452 1.353856 4.147446
H 2.461317 -2.841442 3.241209
H 3.814177 -0.904944 4.111917
H 0.325098 -2.518019 2.132689

96
A6
Ca 1.571182 0.386006 0.108199
Ca -1.571426 -0.385950 0.107592
N 1.450464 2.713947 -0.220502
N 3.833604 1.022113 0.333121
N -3.833604 -1.022784 0.331991
N -1.450274 -2.713788 -0.222852
C 1.188254 0.174818 -4.003262
C 1.166530 0.174807 -2.607000
C 0.000042 0.000633 -1.836658
C -1.166515 -0.172088 -2.607197
C -1.188391 -0.169520 -4.003475
C -0.000113 0.003322 -4.712185
C 1.967752 5.092618 -0.473654
H 1.708121 5.229757 -1.534627
H 2.774128 5.793547 -0.224789
H 1.069554 5.356224 0.105137
C 2.373119 3.662275 -0.200431
C 3.736563 3.431902 0.080708
H 4.362898 4.323597 0.105222
C 4.410823 2.220488 0.321132
C 5.896617 2.309627 0.581665
H 6.141259 1.884017 1.567249
H 6.253959 3.345768 0.546352
H 6.456263 1.716088 -0.157664
C 0.130520 2.968847 -0.622836
C -0.919757 2.857611 0.309595
C -2.253896 2.888973 -0.111419
H -3.062999 2.800262 0.619703
C -2.558187 3.057044 -1.465506
H -3.599128 3.070612 -1.790943
C -1.521799 3.216156 -2.388461
H -1.749783 3.341239 -3.449553
C -0.194447 3.170952 -1.975886
C 4.596138 -0.133724 0.579665
C 4.713985 -0.649674 1.883414
C 5.365041 -1.861025 2.114695
H 5.440293 -2.247596 3.134303
C 5.918567 -2.579051 1.052549
H 6.424407 -3.529867 1.233803
C 5.819288 -2.069461 -0.243742
H 6.252931 -2.621949 -1.081398
C 5.160119 -0.864337 -0.480508
C -5.896323 -2.310752 0.580441
H -6.141008 -1.885090 1.565982
H -6.253411 -3.346987 0.545254
H -6.456163 -1.717445 -0.158942
C -4.410592 -2.221259 0.319674
C -3.736172 -3.432394 0.078436
H -4.362316 -4.324231 0.102585
C -2.372727 -3.662304 -0.203185
C -1.967130 -5.092398 -0.477329
H -1.706770 -5.228660 -1.538229
H -2.773612 -5.793584 -0.229536
H -1.069297 -5.356426 0.101848
C -0.130286 -2.968299 -0.625385
C 0.919934 -2.857851 0.307192
C 2.254092 -2.888705 -0.113796
H 3.063160 -2.800573 0.617431
C 2.558452 -3.055496 -1.468029
H 3.599410 -3.068659 -1.793438
C 1.522115 -3.213907 -2.391163
H 1.750160 -3.338029 -3.452357
C 0.194738 -3.169203 -1.978592
H -0.000152 0.004355 -5.805201
H -2.133137 -0.304145 -4.538030
H 2.132932 0.310432 -4.537691
H 2.159249 0.337201 -2.146597
C -0.000304 -0.001092 2.059155
H -2.159180 -0.335498 -2.147021
C -4.596127 0.132834 0.579324
C -4.714389 0.647754 1.883450
C -5.365417 1.858973 2.115481
H -5.440965 2.244726 3.135379
C -5.918525 2.577909 1.053734
H -6.424345 3.528620 1.235591
C -5.818853 2.069350 -0.242933
H -6.252186 2.622536 -1.080287
C -5.159703 0.864373 -0.480446
H 4.283527 -0.079788 2.710276
H 0.666969 -2.735790 1.362150
H -4.284241 0.077191 2.710009
H -5.061482 0.471932 -1.495437
H -0.612787 -3.220514 -2.711073
H 5.062201 -0.471103 -1.495221
H 0.613104 3.222862 -2.708294
H -0.666859 2.734605 1.364462
C -1.088984 0.464345 2.823879
C -1.111107 0.460996 4.221546
C -0.000712 -0.002091 4.927692
C 1.088173 -0.467043 2.823862
C 1.109885 -0.464669 4.221556
H -1.989312 0.875315 2.335360
H -1.989257 0.829655 4.759770
H -0.000893 -0.002463 6.020676
H 1.988671 -0.877597 2.335303
H 1.987899 -0.833699 4.759749

96
A7*
Ca 2.281350 -0.116109 -0.564596
Ca -2.282016 -0.116817 0.565927
N 3.990681 -1.479044 0.281101
N 4.047558 1.398768 -0.280358
N -3.989771 -1.480372 -0.282347
N -4.048318 1.397376 0.279863

C -1.005962 1.097183 2.740989
C -0.153129 1.159818 1.642918
C 0.316374 -0.007931 0.947085
C -0.027270 -1.246173 1.593608
C -0.875266 -1.314878 2.696504
C -1.433333 -0.146296 3.254191
C 6.128320 -2.362363 1.109922
H 6.025299 -2.424586 2.204662
H 7.189208 -2.187790 0.887473
H 5.829414 -3.338778 0.701664
C 5.270480 -1.250895 0.547237
C 5.905984 -0.011542 0.333734
H 6.976995 0.007162 0.537987
C 5.331780 1.233219 -0.002946
C 6.245835 2.436321 0.005501
H 6.346492 2.862977 -1.004228
H 7.246547 2.181781 0.375453
H 5.822488 3.231317 0.638942
C 3.314307 -2.622726 0.715787
C 2.647503 -3.431268 -0.225724
C 1.830601 -4.484608 0.183923
H 1.320883 -5.091471 -0.567339
C 1.662597 -4.759681 1.541055
H 1.023121 -5.584450 1.863530
C 2.319153 -3.965170 2.485880
H 2.182118 -4.160705 3.552515
C 3.129163 -2.908198 2.083407
C 3.483292 2.651987 -0.560017
C 3.643477 3.285932 -1.805945
C 2.931449 4.444461 -2.108690
H 3.064859 4.919194 -3.084438
C 2.039168 4.992761 -1.183143
H 1.476915 5.896549 -1.429223
C 1.875985 4.375353 0.057778
H 1.180871 4.790424 0.791527
C 2.592275 3.220473 0.368754
C -6.126190 -2.364031 -1.114165
H -6.022421 -2.425756 -2.208864
H -7.187300 -2.189925 -0.892394
H -5.827238 -3.340498 -0.706084
C -5.269226 -1.252415 -0.550399
C -5.905275 -0.013136 -0.338058
H -6.975929 0.005386 -0.544201
C -5.331896 1.231728 -0.000370
C -6.245969 2.434790 -0.010841
H -6.348300 2.861836 0.998558
H -7.246047 2.180112 -0.382408
H -5.821547 3.229540 -0.643870
C -3.484583 2.650720 0.560082
C -2.592664 3.219359 -0.367739
C -1.876899 4.374350 -0.055965
H -1.181018 4.789527 -0.788944
C -2.041525 4.991703 1.184793
H -1.479657 5.895557 1.431506
C -2.934727 4.443258 2.109363
H -3.069281 4.917971 3.084964
C -3.646239 3.284611 1.805834
H -2.101340 -0.197877 4.115653
H -1.115000 -2.289905 3.132277
H -1.366274 2.024169 3.194746
H 0.104201 2.151400 1.255396
C -0.317053 -0.008297 -0.945810
H 0.350958 -2.189859 1.183555
C -3.312738 -2.624109 -0.715821
C -3.126458 -2.910452 -2.083104
C -2.315996 -3.967579 -2.484244
H -2.178113 -4.163808 -3.550645
C -1.660042 -4.761362 -1.538388
H -1.020231 -5.586275 -1.859823
C -1.829108 -4.485385 -0.181569
H -1.319869 -5.091669 0.570492
C -2.646511 -3.431914 0.226738
H 4.312159 2.836523 -2.543859
H -2.466284 2.728653 -1.335708
H -3.597485 -2.262818 -2.825272
H -2.790952 -3.223874 1.290097
H -4.315695 2.835093 2.542984
H 2.467093 2.729767 1.336877
H 3.600726 -2.260022 2.824768
H 2.791064 -3.223865 -1.289319
C 0.026929 -1.246669 -1.591897
C 0.874948 -1.315546 -2.694755
C 1.432584 -0.147001 -3.252941
C 0.152092 1.159344 -1.642084
C 1.004876 1.096533 -2.740196
H -0.350871 -2.190322 -1.181375
H 1.114994 -2.290669 -3.130135
H 2.100614 -0.198710 -4.114378
H -0.105487 2.150991 -1.254868
H 1.364913 2.023450 -3.194306

96
A8
Ca -2.444353 -0.201825 0.979086
Ca 2.443568 -0.202743 -0.978131
N -4.030089 1.413355 0.441250
N -4.034002 -1.454059 -0.183339
N 4.031452 1.410635 -0.440698
N 4.032597 -1.456990 0.182830
C -0.673353 -1.392955 2.739998
C -0.101458 -1.394525 1.486245
C 0.014594 -0.157879 0.709050
C -0.157852 1.067332 1.498127
C -0.731055 1.027141 2.752316
C -1.152185 -0.195111 3.351700
C -6.081500 2.578385 -0.188640
H -6.132893 3.131680 0.761534
H -7.100403 2.364188 -0.533537
H -5.598842 3.252582 -0.913575
C -5.264909 1.318637 -0.029857
C -5.863546 0.103149 -0.425702
H -6.902287 0.176468 -0.749938
C -5.264051 -1.160270 -0.593175
C -6.102723 -2.213728 -1.283018
H -5.959201 -2.184216 -2.374522
H -7.170035 -2.040424 -1.089651

H -5.834066 -3.224380 -0.944610
C -3.412945 2.656186 0.674790
C -2.624102 3.242830 -0.330453
C -1.868180 4.382853 -0.065659
H -1.247023 4.812092 -0.855215
C -1.888908 4.962915 1.204484
H -1.293647 5.854948 1.413224
C -2.678285 4.394397 2.206090
H -2.701072 4.841250 3.203452
C -3.433003 3.250986 1.947006
C -3.342607 -2.590118 -0.618164
C -3.086021 -2.834687 -1.981996
C -2.274360 -3.893449 -2.374970
H -2.082240 -4.056005 -3.438497
C -1.684064 -4.730070 -1.422583
H -1.042548 -5.556424 -1.736323
C -1.917974 -4.493124 -0.068354
H -1.458013 -5.132151 0.688521
C -2.738645 -3.438570 0.331418
C 6.084237 2.573338 0.189091
H 6.137519 3.125839 -0.761444
H 7.102460 2.358093 0.535338
H 5.601547 3.248729 0.912887
C 5.266241 1.314514 0.030202
C 5.863650 0.098302 0.425756
H 6.902460 0.170508 0.750013
C 5.262977 -1.164612 0.592696
C 6.100693 -2.219299 1.281818
H 5.956512 -2.191060 2.373264
H 7.168214 -2.046394 1.089289
H 5.831639 -3.229397 0.942045
C 3.340155 -2.592696 0.616791
C 2.735188 -3.439706 -0.333472
C 1.913535 -4.493822 0.065451
H 1.452819 -5.131685 -0.691945
C 1.679596 -4.731787 1.419494
H 1.037310 -5.557791 1.732576
C 2.270827 -3.896586 2.372547
H 2.078676 -4.059893 3.435951
C 3.083464 -2.838254 1.980435
H -1.571174 -0.209051 4.358117
H -0.903660 1.971060 3.276570
H -0.776887 -2.346235 3.267801
H 0.234395 -2.336631 1.050593
C -0.015200 -0.156539 -0.707582
H 0.103872 2.031744 1.060476
C 3.415800 2.654224 -0.674197
C 3.438161 3.250122 -1.945850
C 2.684882 4.394481 -2.204925
H 2.709415 4.842181 -3.201866
C 1.894688 4.962876 -1.203885
H 1.300534 5.855642 -1.412647
C 1.871720 4.381738 0.065723
H 1.249970 4.810893 0.854852
C 2.626188 3.240754 0.330508
H -3.500520 -2.152846 -2.726813
H 2.933459 -3.269786 -1.395805
H 4.039408 2.787540 -2.732378
H 2.595435 2.766785 1.314314
H 3.498666 -2.157481 2.725836
H -2.936872 -3.269381 1.393870
H -4.033718 2.788343 2.733909
H -2.595011 2.769639 -1.314680
C 0.158363 1.070032 -1.494281
C 0.731500 1.031861 -2.748546
C 1.151444 -0.189618 -3.350370
C 0.099630 -1.391751 -1.487246
C 0.671467 -1.388188 -2.741054
H -0.102720 2.033787 -1.054823
H 0.904738 1.976642 -3.271016
H 1.570364 -0.201979 -4.356836
H -0.237066 -2.334422 -1.053458
H 0.774058 -2.340497 -3.270797

98

B1

Ca -2.498288 -0.003142 0.259014
Ca 1.914662 0.027519 0.394402
N -4.059678 -1.735299 0.196644
N -4.511543 1.145256 0.057172
N 3.194890 1.813962 -0.449854
N 3.473589 -1.123940 -0.923704
C -0.262483 -0.769444 -0.846436
C -0.278504 0.738071 -0.881682
C -0.314342 1.443667 0.330959
C -0.371699 0.801149 1.582031
C -0.358454 -0.709885 1.616209
C -0.289765 -1.413716 0.401143
C -6.098943 -3.081624 0.168043
H -5.751965 -3.721088 -0.658507
H -7.186554 -2.961784 0.090643
H -5.868308 -3.626163 1.096803
C -5.383839 -1.751270 0.140424
C -6.181588 -0.589797 0.041772
H -7.257665 -0.762195 0.001068
C -5.776826 0.755333 -0.052201
C -6.864403 1.783469 -0.267861
H -6.638108 2.716340 0.269103
H -7.836839 1.401051 0.069172
H -6.961497 2.041541 -1.334367
C -3.303592 -2.919048 0.266031
C -2.957043 -3.481632 1.506469
C -2.082839 -4.566794 1.575241
H -1.822848 -4.990073 2.549003
C -1.532348 -5.103846 0.409760
H -0.842310 -5.948566 0.465081
C -1.868540 -4.551736 -0.827827
H -1.430729 -4.954602 -1.743502
C -2.745653 -3.470492 -0.900797
C -4.071419 2.442609 -0.221108
C -3.340425 3.146508 0.758703
C -2.733490 4.366273 0.459439
H -2.157766 4.883719 1.230460
C -2.855539 4.922780 -0.812802
H -2.380169 5.877676 -1.046344
C -3.590731 4.243703 -1.789313

H -3.686115 4.666911 -2.792829
C -4.184333 3.018336 -1.503857
C 5.138666 3.093272 -1.195967
H 4.654529 3.623425 -2.030889
H 6.194431 2.938307 -1.450853
H 5.071278 3.763120 -0.325056
C 4.430146 1.785980 -0.922862
C 5.145376 0.600475 -1.214128
H 6.176008 0.752141 -1.537694
C 4.672348 -0.723256 -1.328740
C 5.622720 -1.723282 -1.950986
H 5.538181 -2.704484 -1.460934
H 6.660861 -1.372587 -1.881853
H 5.394279 -1.878601 -3.016857
C 2.950332 -2.374306 -1.279849
C 2.649270 -2.698587 -2.616604
C 2.022700 -3.900169 -2.934355
H 1.793624 -4.127917 -3.978877
C 1.671163 -4.806788 -1.929717
H 1.177168 -5.747570 -2.182497
C 1.957277 -4.494265 -0.601220
H 1.685081 -5.189085 0.196508
C 2.591668 -3.295621 -0.278795
H -0.273076 -2.506648 0.422741
H -0.425685 -1.248094 2.560326
H -0.173959 -1.348938 -1.764030
H -0.215702 1.272576 -1.827909
H -0.310824 2.535976 0.297107
H -0.389873 1.380075 2.504622
C 2.470432 3.016953 -0.367150
C 1.765199 3.497256 -1.485099
C 0.921145 4.600035 -1.374967
H 0.362980 4.943686 -2.249137
C 0.770851 5.254715 -0.149201
H 0.106487 6.116986 -0.063687
C 1.474814 4.795726 0.963351
H 1.367210 5.300847 1.926731
C 2.314020 3.684727 0.857863
H -3.257045 2.717171 1.761077
H 2.888888 -1.974352 -3.398437
H 1.871093 2.965141 -2.432906
H 2.855458 3.317616 1.729653
H 2.819981 -3.054864 0.760634
H -4.720383 2.469679 -2.281468
H -3.000699 -3.021886 -1.863547
H -3.376045 -3.042867 2.415231
C 3.247290 -1.572060 2.977063
C 2.516427 -0.452311 3.395783
C 3.023022 0.835931 3.171316
C 4.264439 0.993934 2.533078
C 4.479642 -1.408838 2.337890
C 4.987486 -0.122528 2.115286
H 1.558052 -0.582312 3.900199
H 2.458267 1.707740 3.507314
H 4.661786 1.993455 2.347967
H 5.039339 -2.281768 1.996119
H 5.936090 0.009362 1.593362
H 2.846989 -2.573126 3.155135

98
B2*
Ca -2.143610 -1.134047 -0.003924
Ca 2.440323 0.339714 -0.400382
N -2.535605 -0.946902 2.330384
N -3.636586 0.713940 0.083449
N 2.644988 2.556599 0.369645
N 4.719950 0.524666 -0.094021
C -3.088446 -1.832883 -2.654578
C -1.820050 -1.845948 -3.199867
C -0.765363 -2.708676 -2.705228
C -1.188892 -3.631571 -1.667672
C -2.475629 -3.627716 -1.151220
C -3.458430 -2.700519 -1.588846
C -3.077781 -0.008580 4.556591
H -2.929957 -1.025638 4.943641
H -4.018378 0.391631 4.959181
H -2.264110 0.619535 4.952788
C -3.112307 0.016716 3.041256
C -3.820713 1.091469 2.474741
H -4.275704 1.772122 3.195979
C -4.093406 1.397086 1.122405
C -4.990871 2.593015 0.886860
H -4.651037 3.177367 0.019627
H -5.027555 3.242268 1.770992
H -6.019247 2.269790 0.660889
C -1.647604 -1.866570 2.882945
C -0.556366 -1.495892 3.699231
C 0.392516 -2.433195 4.094673
H 1.231205 -2.115284 4.720066
C 0.295453 -3.766952 3.683894
H 1.048276 -4.496565 3.989983
C -0.776720 -4.150564 2.878168
H -0.873189 -5.188782 2.550657
C -1.736506 -3.217896 2.488058
C -4.105008 1.016823 -1.209796
C -3.265533 1.639021 -2.147584
C -3.689751 1.833254 -3.462012
H -3.019077 2.315138 -4.178159
C -4.957597 1.413694 -3.865821
H -5.284811 1.558743 -4.897833
C -5.804288 0.802024 -2.936891
H -6.797488 0.463192 -3.242642
C -5.382956 0.603404 -1.624277
C 3.505420 4.652751 1.283645
H 3.043396 5.278475 0.504583
H 4.452224 5.112463 1.591673
H 2.810947 4.662979 2.137705
C 3.703537 3.242777 0.779372
C 5.028102 2.748167 0.773371
H 5.784221 3.439536 1.146013
C 5.511856 1.505576 0.330099
C 7.007618 1.295781 0.371127
H 7.252755 0.267143 0.674253
H 7.483888 1.998754 1.066517
H 7.457474 1.453221 -0.621777
C 5.194091 -0.655283 -0.679171

C 5.893398 -0.663105 -1.903806
C 6.215666 -1.863038 -2.530780
H 6.755896 -1.845189 -3.480915
C 5.838833 -3.085530 -1.966295
H 6.085613 -4.023656 -2.468000
C 5.140299 -3.093976 -0.759797
H 4.841619 -4.041940 -0.305279
C 4.823658 -1.895387 -0.118967
H -4.496076 -2.771693 -1.256987
H -2.739651 -4.378628 -0.399474
H -3.833056 -1.151376 -3.071604
H -1.612250 -1.186484 -4.047226
C 0.762679 -1.433158 -1.943861
H -0.484938 -4.387093 -1.309094
C 1.356972 3.136400 0.387155
C 0.839195 3.749447 -0.765442
C -0.497183 4.149056 -0.817692
H -0.888808 4.611020 -1.727454
C -1.340396 3.934860 0.274223
H -2.393726 4.214874 0.220259
C -0.829231 3.348199 1.434604
H -1.490370 3.156796 2.283179
C 0.510216 2.966952 1.495653
H -2.278118 1.972121 -1.828352
H 6.159103 0.290535 -2.365545
H 1.495280 3.881176 -1.629593
H 0.912412 2.484358 2.389132
H 4.297499 -1.904116 0.840350
H -6.028067 0.096253 -0.903007
H -2.589871 -3.528498 1.878990
H -0.439907 -0.450570 3.988859
C 1.150402 -2.024048 -0.665274
C 0.769328 -1.488435 0.545371
C 0.140977 -0.181042 0.621756
C 0.177531 -0.081411 -1.836678
C -0.148117 0.483184 -0.619712
H 1.668794 -2.986335 -0.673816
H 0.990953 -2.026010 1.470568
H -0.089542 0.287786 1.574460
H -0.073816 0.461910 -2.750682
H -0.617773 1.467510 -0.587564
H -0.047914 -3.094495 -3.437906
H 1.437803 -1.605482 -2.791097

98

B3

Ca 2.418492 0.892073 0.864612
Ca -2.476374 0.099142 -0.271315
N 3.092753 -1.315477 1.517272
N 3.545477 0.128769 -1.100156
N -2.520412 -2.042087 -1.220294
N -4.743294 -0.340051 -0.311033
C 3.074620 3.499931 0.556247
C 1.746279 3.554431 0.201007
C 0.613467 3.417714 1.201687
C 1.155591 2.754812 2.453879
C 2.494746 2.749248 2.776532
C 3.514993 3.070500 1.839258
C 4.309457 -3.464815 1.615721
H 4.232359 -3.378318 2.707904
H 5.340869 -3.733799 1.350048
H 3.667249 -4.304566 1.305581
C 3.915606 -2.174415 0.923681
C 4.495493 -1.973099 -0.342075
H 5.182646 -2.758336 -0.659847
C 4.293764 -0.946283 -1.292078
C 4.986403 -1.128821 -2.624804
H 4.258662 -1.056543 -3.447719
H 5.501014 -2.095533 -2.686000
H 5.721199 -0.327717 -2.797272
C 2.342312 -1.662370 2.643290
C 1.505791 -2.797501 2.688239
C 0.640813 -3.008593 3.757938
H -0.004225 -3.891488 3.759880
C 0.577848 -2.095951 4.815020
H -0.110202 -2.259552 5.647347
C 1.411171 -0.977024 4.793835
H 1.384484 -0.256898 5.615596
C 2.286285 -0.767936 3.729701
C 3.401681 1.071924 -2.138403
C 2.254493 1.065656 -2.946956
C 2.056345 2.054283 -3.910097
H 1.154797 2.031799 -4.528486
C 2.998948 3.068482 -4.085601
H 2.840359 3.846893 -4.835411
C 4.148645 3.077494 -3.292661
H 4.894425 3.866397 -3.420323
C 4.349051 2.090728 -2.329322
C -3.218084 -4.263437 -1.963480
H -2.760265 -4.163273 -2.959747
H -4.122389 -4.877660 -2.049620
H -2.483802 -4.795971 -1.340049
C -3.518517 -2.901290 -1.385264
C -4.867393 -2.625221 -1.070262
H -5.564223 -3.444496 -1.247044
C -5.446443 -1.426661 -0.617586
C -6.948891 -1.398404 -0.468624
H -7.234458 -0.937671 0.489299
H -7.374936 -2.407806 -0.523229
H -7.411649 -0.789338 -1.260490
C -5.352887 0.884944 0.006869
C -5.982115 1.673991 -0.975652
C -6.461549 2.944395 -0.666559
H -6.946390 3.541271 -1.443391
C -6.315270 3.463591 0.622648
H -6.685483 4.463537 0.858916
C -5.685908 2.696296 1.602413
H -5.564493 3.091395 2.614073
C -5.209164 1.420195 1.300719
H 4.568010 3.091984 2.120686
H 2.784239 2.390605 3.770675
H 3.819053 3.717233 -0.214748
H 1.496745 3.859431 -0.819110
C -0.590708 2.647169 0.524776
H 0.435941 2.442501 3.213305
C -1.209012 -2.375989 -1.625668

C -0.736777 -1.956720 -2.880356
C 0.600476 -2.141634 -3.235987
H 0.950886 -1.807780 -4.215675
C 1.491451 -2.730778 -2.339441
H 2.545176 -2.848533 -2.594377
C 1.028067 -3.159456 -1.094320
H 1.731630 -3.606686 -0.390336
C -0.309681 -2.996444 -0.741965
H 1.523753 0.268234 -2.805862
H -6.075079 1.276546 -1.989433
H -1.436016 -1.476632 -3.570162
H -0.670202 -3.307678 0.240734
H -4.728017 0.811542 2.071162
H 5.238448 2.103325 -1.694764
H 2.949401 0.100747 3.727997
H 1.512504 -3.494600 1.849968
C -1.485554 1.944445 1.513379
C -1.154043 0.674898 1.947673
C -0.307686 -0.183392 1.180782
C -0.124706 1.601159 -0.485032
C 0.102884 0.288637 -0.096373
H -2.222032 2.530634 2.070876
H -1.643064 0.260168 2.836662
H -0.088664 -1.196490 1.509339
H 0.121460 1.912707 -1.502048
H 0.548615 -0.419140 -0.804649
H 0.187904 4.416039 1.469123
H -1.164022 3.419002 -0.016196

98
B4*
Ca -2.193079 -0.818670 0.527793
Ca 2.333598 0.856730 0.824778
N -3.945125 0.473194 -0.357788
N -2.835186 -2.081912 -1.332932
N 4.168722 -0.302252 -0.026032
N 3.381502 2.498279 -0.456069
C -0.913234 -2.897968 3.235734
C -0.119144 -1.915350 3.737959
C -0.447935 -0.483668 3.416344
C -1.960720 -0.307627 3.388766
C -2.763155 -1.360480 2.999266
C -2.203020 -2.616485 2.643973
C -6.110907 1.034463 -1.372327
H -6.351324 1.594583 -0.456820
H -7.000892 0.483657 -1.703105
H -5.870783 1.777551 -2.148589
C -4.949057 0.093289 -1.143077
C -5.012606 -1.151968 -1.799460
H -5.937492 -1.346709 -2.343834
C -3.987743 -2.102144 -1.989228
C -4.254426 -3.187086 -3.008682
H -3.862027 -2.905663 -3.998483
H -5.333593 -3.357301 -3.119063
H -3.767017 -4.129967 -2.722093
C -3.794073 1.812734 0.030573
C -3.488452 2.815880 -0.909293
C -3.209479 4.115402 -0.497572
H -2.971692 4.876428 -1.244852
C -3.215510 4.445969 0.860707
H -2.987683 5.464522 1.182524
C -3.517426 3.461149 1.801076
H -3.532104 3.706341 2.866193
C -3.808090 2.159117 1.392467
C -1.734105 -2.847667 -1.735950
C -1.109650 -2.635713 -2.980264
C 0.073041 -3.295501 -3.302779
H 0.545879 -3.113670 -4.271560
C 0.665505 -4.173505 -2.390727
H 1.596604 -4.683117 -2.646391
C 0.058208 -4.387416 -1.152600
H 0.516460 -5.060447 -0.424960
C -1.130652 -3.736374 -0.826518
C 6.266209 -0.917934 -1.159521
H 6.413485 -1.656380 -0.359076
H 7.213630 -0.395181 -1.345919
H 6.012170 -1.475043 -2.075232
C 5.175424 0.068001 -0.804086
C 5.320662 1.371196 -1.331738
H 6.219081 1.531592 -1.929596
C 4.460617 2.480881 -1.229625
C 4.840111 3.705292 -2.032444
H 4.399033 3.669543 -3.041368
H 5.929912 3.761881 -2.156331
H 4.485303 4.627204 -1.550628
C 2.399893 3.485846 -0.490703
C 1.951244 4.037812 0.729128
C 0.849504 4.891625 0.769817
H 0.519671 5.300112 1.728089
C 0.174593 5.227814 -0.403022
H -0.685507 5.897764 -0.370685
C 0.608830 4.691391 -1.618920
H 0.078282 4.937936 -2.542475
C 1.699372 3.828603 -1.666734
H -2.872067 -3.448595 2.409496
H -3.853112 -1.224577 2.966586
H -0.617534 -3.946330 3.354757
H 0.788396 -2.142227 4.301205
C 0.094682 -0.068650 2.026573
H -2.396520 0.610308 3.789180
C 3.949540 -1.624172 0.383342
C 3.777045 -1.889372 1.756919
C 3.398643 -3.156363 2.199140
H 3.253638 -3.329249 3.267785
C 3.205072 -4.193067 1.287115
H 2.913299 -5.186864 1.633665
C 3.383203 -3.944213 -0.076493
H 3.231902 -4.748443 -0.799810
C 3.735306 -2.676356 -0.528786
H -1.552090 -1.918883 -3.675686
H 2.494870 3.795889 1.648766
H 3.932164 -1.082835 2.479112
H 3.820452 -2.478255 -1.598998
H 2.005187 3.378926 -2.613314
H -1.599257 -3.890764 0.148986
H -4.052646 1.389809 2.127354

H -3.445552 2.547813 -1.967532
C -0.416120 1.147075 1.446869
C -0.353873 1.405087 0.079662
C 0.125982 0.423309 -0.825738
C 0.460263 -1.079644 1.071335
C 0.506281 -0.827676 -0.307904
H -0.813441 1.917988 2.108292
H -0.691091 2.373831 -0.294407
H 0.142892 0.615847 -1.898141
H 0.763876 -2.054910 1.452484
H 0.826582 -1.626580 -0.979743
H 0.001669 0.205339 4.143368
H 1.810104 0.511538 2.793726

98

B5

Ca -2.603461 0.650363 1.258819
Ca 2.734080 -0.752285 0.879101
N -4.173421 -0.885626 0.415694
N -3.810910 1.998987 -0.244018
N 3.959659 1.000457 -0.081427
N 3.230433 -1.641763 -1.240147
C -0.883888 -0.717215 -1.161454
C -0.630592 0.629812 -1.443460
C -0.034356 1.442868 -0.476688
C 0.291546 0.906405 0.777986
C 0.051980 -0.451710 1.074408
C -0.532555 -1.257113 0.078268
C -6.240077 -1.831740 -0.543854
H -6.069002 -2.261406 -1.543714
H -7.279363 -1.476237 -0.519302
H -6.121051 -2.639233 0.191216
C -5.291595 -0.684105 -0.270726
C -5.694019 0.574893 -0.761781
H -6.680762 0.592234 -1.227767
C -4.985730 1.793165 -0.827049
C -5.665154 2.905923 -1.596655
H -5.323606 3.895598 -1.264545
H -6.754411 2.841975 -1.468291
H -5.461644 2.826853 -2.676417
C -3.641191 -2.145613 0.682034
C -3.162926 -2.419910 1.982250
C -2.456343 -3.591131 2.254363
H -2.088331 -3.771809 3.267099
C -2.219187 -4.524668 1.244543
H -1.665381 -5.441330 1.458309
C -2.701375 -4.272352 -0.043906
H -2.517701 -4.992556 -0.844710
C -3.396426 -3.101029 -0.326821
C -3.000076 3.102863 -0.508030
C -2.406805 3.786514 0.574944
C -1.461512 4.788346 0.358849
H -1.013062 5.294246 1.216724
C -1.093548 5.146024 -0.938482
H -0.361882 5.938554 -1.107036
C -1.676878 4.480311 -2.020971
H -1.391017 4.745980 -3.042333
C -2.610810 3.469603 -1.813141
C 5.901546 2.018308 -1.202330
H 5.554150 2.628872 -2.050409
H 6.903245 1.646453 -1.457666
H 5.973187 2.680096 -0.327872
C 4.962471 0.860978 -0.942527
C 5.219615 -0.324722 -1.663307
H 6.142507 -0.323644 -2.245527
C 4.347972 -1.403802 -1.922438
C 4.741179 -2.316249 -3.064023
H 4.377681 -3.340555 -2.900567
H 5.833250 -2.334126 -3.180302
H 4.315830 -1.966025 -4.017409
C 2.227611 -2.473856 -1.759063
C 1.576695 -2.167409 -2.968858
C 0.503144 -2.933636 -3.414590
H 0.011530 -2.675882 -4.356564
C 0.039026 -4.012596 -2.657104
H -0.809851 -4.605694 -3.004216
C 0.674415 -4.324334 -1.454626
H 0.324906 -5.165185 -0.851308
C 1.759622 -3.569290 -1.012214
H -0.721302 -2.312717 0.273319
H -2.307055 1.074129 3.244072
H -1.354840 -1.360728 -1.905026
H -0.907605 1.051151 -2.411101
H 0.155947 2.498784 -0.676128
H 0.744683 1.550512 1.535774
C 3.503389 2.262460 0.321582
C 3.010445 3.195633 -0.610442
C 2.424920 4.384412 -0.185560
H 2.042167 5.089227 -0.926908
C 2.302396 4.668271 1.177412
H 1.831325 5.596401 1.507838
C 2.789672 3.753125 2.111265
H 2.704714 3.962651 3.180381
C 3.389517 2.566327 1.690025
H -2.701055 3.526695 1.598215
H 1.909001 -1.296413 -3.537908
H 3.061433 2.953630 -1.674282
H 3.778507 1.855823 2.422749
H 2.259085 -3.821096 -0.074872
H -3.030418 2.926950 -2.662279
H -3.724255 -2.891194 -1.346468
H -3.358829 -1.701206 2.786237
C 1.040515 -2.445590 2.307526
C 0.424450 -1.061091 2.451553
C 1.350740 -0.169720 3.257433
C 2.634254 -0.524965 3.585426
C 2.335527 -2.729704 2.663394
C 3.242998 -1.752731 3.175257
H -0.555546 -1.129353 2.973565
H 0.925138 0.754997 3.653892
H 3.223873 0.179736 4.181702
H 2.692114 -3.756104 2.521728
H 4.224542 -2.043122 3.550031
H 0.392834 -3.251633 1.958454

98

B6*
Ca -2.497616 0.048970 0.911482
Ca 2.716594 -0.139304 1.001416
N -3.945814 -1.615097 0.235682
N -4.205606 1.289891 -0.104782
N 3.708935 1.647680 -0.187253
N 3.722085 -1.286066 -0.785460
C -0.552822 -0.710408 -1.579514
C -0.740994 0.617552 -1.990029
C -0.490960 1.648715 -1.077825
C -0.071527 1.368893 0.223731
C 0.128169 0.026174 0.663541
C -0.135322 -1.009655 -0.282861
C -5.893248 -2.892917 -0.543886
H -5.625562 -3.282751 -1.538697
H -6.981427 -2.748846 -0.527616
H -5.619655 -3.664280 0.190409
C -5.174094 -1.594227 -0.262110
C -5.876092 -0.403731 -0.538340
H -6.908869 -0.535018 -0.864127
C -5.410958 0.927064 -0.532631
C -6.387769 1.968383 -1.034798
H -6.191564 2.953754 -0.590500
H -7.417215 1.666889 -0.798342
H -6.324985 2.079834 -2.128775
C -3.167014 -2.772112 0.339097
C -2.631122 -3.127668 1.592178
C -1.713655 -4.173940 1.704361
H -1.312549 -4.431982 2.687267
C -1.309043 -4.880769 0.573816
H -0.580828 -5.688656 0.660226
C -1.835274 -4.536046 -0.675140
H -1.511995 -5.074238 -1.568995
C -2.750886 -3.496994 -0.795577
C -3.669938 2.561451 -0.334320
C -3.077145 3.259452 0.737620
C -2.378974 4.446958 0.522462
H -1.916356 4.958914 1.369249
C -2.272595 4.980102 -0.762351
H -1.728019 5.911466 -0.930555
C -2.869773 4.306906 -1.832322
H -2.786924 4.710767 -2.844881
C -3.549347 3.109627 -1.626940
C 5.422691 2.872451 -1.455920
H 4.919302 3.287021 -2.343181
H 6.470877 2.689242 -1.726891
H 5.380081 3.641678 -0.671499
C 4.757039 1.591158 -1.002046
C 5.314209 0.394398 -1.499648
H 6.226660 0.517983 -2.084322
C 4.772085 -0.907373 -1.507113
C 5.451964 -1.911099 -2.412144
H 5.495175 -2.902708 -1.937977
H 6.469292 -1.586107 -2.665245
H 4.894394 -2.032410 -3.353775
C 3.074472 -2.505302 -1.040575
C 2.349133 -2.712474 -2.228306
C 1.636908 -3.891579 -2.430291
H 1.081372 -4.033935 -3.361178
C 1.622173 -4.886480 -1.449506
H 1.067304 -5.812963 -1.612209
C 2.320444 -4.683699 -0.259193
H 2.313026 -5.451008 0.519001
C 3.038340 -3.506109 -0.054605
H 0.008706 -2.054462 0.003207
H -1.892557 0.175381 2.951201
H -0.734762 -1.534912 -2.272143
H -1.090847 0.844189 -2.998471
H -0.637365 2.690251 -1.366933
H 0.139137 2.197066 0.902783
C 3.013534 2.849971 0.015526
C 2.330421 3.485742 -1.037922
C 1.520078 4.591489 -0.797711
H 0.985161 5.058895 -1.628343
C 1.365373 5.088520 0.499213
H 0.721449 5.950428 0.686672
C 2.045173 4.473316 1.551252
H 1.939596 4.854666 2.570073
C 2.862603 3.368523 1.313196
H -3.174998 2.858548 1.752191
H 2.337570 -1.919298 -2.979161
H 2.409734 3.065891 -2.042987
H 3.394508 2.887974 2.136924
H 3.595585 -3.350959 0.872345
H -3.965592 2.561485 -2.474038
H -3.130820 -3.203434 -1.776378
H -2.948866 -2.575688 2.482429
C 0.915302 -1.644088 2.414079
C 0.380893 -0.277468 2.103204
C 1.071895 0.785474 2.914108
C 2.252168 0.539639 3.572252
C 2.110366 -1.818688 3.067207
C 2.918559 -0.724399 3.517234
H -0.875719 -0.133911 2.580415
H 0.572119 1.749227 3.041587
H 2.706453 1.353856 4.147446
H 2.461317 -2.841441 3.241210
H 3.814177 -0.904944 4.111917
H 0.325098 -2.518019 2.132689

98
B8
Ca -1.982961 -0.011754 -0.199254
Ca 2.897500 -0.814637 0.891822
N -2.708465 1.614979 -1.653867
N -4.249880 -0.600663 -0.449067
N 2.597539 -2.374724 -0.949797
N 5.089221 -1.003356 0.022913
C -3.052926 1.131306 2.718230
C -2.974870 -0.130954 3.307786
C -1.759444 -0.823298 3.293197
C -0.629566 -0.279911 2.676399
C -0.708141 0.980215 2.049374
C -1.926261 1.683984 2.096790
C -3.720570 2.511705 -3.691689
H -4.122369 3.451112 -3.280881

H -4.377760 2.183139 -4.506347
H -2.725665 2.749539 -4.096812
C -3.621408 1.471108 -2.603514
C -4.547807 0.403517 -2.638829
H -5.179044 0.361952 -3.526987
C -4.932434 -0.449699 -1.585415
C -6.223250 -1.213937 -1.777064
H -6.139091 -2.237073 -1.381775
H -6.502047 -1.256415 -2.837661
H -7.048422 -0.730924 -1.231295
C -1.989195 2.820842 -1.502146
C -0.657713 2.921708 -1.928393
C 0.095287 4.057575 -1.633039
H 1.140987 4.100677 -1.943672
C -0.473618 5.116735 -0.924680
H 0.121662 6.000828 -0.686189
C -1.805378 5.033121 -0.512412
H -2.259281 5.856873 0.044548
C -2.557291 3.893230 -0.793358
C -4.872787 -1.185913 0.669090
C -4.434996 -2.417164 1.182048
C -4.988432 -2.945906 2.348668
H -4.631345 -3.907326 2.726159
C -5.993816 -2.257533 3.027358
H -6.426987 -2.671037 3.940724
C -6.440713 -1.031429 2.525248
H -7.224685 -0.479607 3.050406
C -5.885686 -0.499946 1.364316
C 3.055444 -3.896773 -2.830921
H 2.224568 -4.516011 -2.463473
H 3.889270 -4.553387 -3.111028
H 2.708879 -3.397280 -3.749547
C 3.495823 -2.889949 -1.786390
C 4.871568 -2.601746 -1.767188
H 5.464521 -3.156525 -2.495900
C 5.608349 -1.686823 -0.979218
C 7.074320 -1.547949 -1.329866
H 7.218959 -0.808262 -2.133526
H 7.471658 -2.504979 -1.694840
H 7.667321 -1.219367 -0.465555
C 5.747617 0.033541 0.683541
C 5.769869 0.034434 2.093757
C 6.258787 1.132881 2.800589
H 6.261014 1.109881 3.893203
C 6.741729 2.252048 2.122067
H 7.119088 3.114432 2.675893
C 6.733056 2.259045 0.723633
H 7.100775 3.134063 0.181107
C 6.241378 1.169938 0.011273
H -1.988421 2.664927 1.616696
H -3.997288 1.679654 2.721982
H -3.861063 -0.580497 3.759237
H -1.690211 -1.809606 3.759036
H 0.320521 -0.844025 2.664338
C 1.233329 -2.550924 -1.122461
C 0.457776 -3.024979 -0.037631
C -0.932228 -3.082160 -0.121804
H -1.498394 -3.467819 0.730357
C -1.598710 -2.661871 -1.284919
H -2.685146 -2.739546 -1.371864
C -0.841668 -2.177043 -2.363823
H -1.348252 -1.840913 -3.272858
C 0.547746 -2.123103 -2.283867
H -3.661374 -2.969559 0.647665
H 5.400922 -0.846861 2.628856
H 0.973787 -3.313357 0.882794
H 1.124956 -1.717978 -3.116902
H 6.196396 1.199245 -1.078884
H -6.214234 0.470200 0.983967
H -3.592085 3.805989 -0.452545
H -0.208561 2.085875 -2.465232
C 1.759901 1.704972 1.815560
C 0.410647 1.490188 1.177547
C 0.624786 0.530652 -0.001548
C 1.693209 0.844451 -0.865040
C 2.796586 1.989737 0.965266
C 2.702809 1.713321 -0.443475
H 0.080141 2.462672 0.757238
H 0.320110 -0.525405 0.080310
H 1.809610 0.304018 -1.810176
H 3.764370 2.307054 1.368977
H 3.523659 1.985570 -1.107684
H 1.874306 1.722653 2.902579
H 2.107906 -1.789923 2.564643

98
B9*
Ca -2.978114 0.433065 -0.485137
Ca 3.027801 0.406646 0.661647
N -4.973926 1.576196 -0.354242
N -4.509775 -1.134362 -1.204965
N 4.092521 -1.700516 0.934042
N 5.264706 0.901943 0.063818
C -1.859132 1.056459 2.560742
C -2.515449 -0.161666 2.763284
C -2.172111 -1.262546 1.970358
C -1.229599 -1.125440 0.946605
C -0.605496 0.112517 0.692537
C -0.898110 1.185273 1.553849
C -7.347495 2.237350 -0.262283
H -7.768888 2.057939 0.739851
H -8.169954 2.128296 -0.982325
H -6.982170 3.273299 -0.289819
C -6.244029 1.252384 -0.569583
C -6.626740 0.011651 -1.111188
H -7.690198 -0.104878 -1.324751
C -5.824613 -1.123676 -1.366585
C -6.535075 -2.363914 -1.855080
H -5.896040 -2.945394 -2.534702
H -7.467612 -2.102292 -2.371975
H -6.798534 -3.022515 -1.012144
C -4.514476 2.649711 0.398905
C -3.399036 3.367744 -0.090898
C -2.738002 4.306442 0.702046
H -1.875397 4.841095 0.297129
C -3.181570 4.567624 1.996974

H -2.665083 5.299519 2.621158
C -4.296416 3.879471 2.488390
H -4.649040 4.074487 3.504656
C -4.950929 2.929595 1.711733
C -3.709367 -2.281573 -1.285791
C -2.596760 -2.276509 -2.152285
C -1.653856 -3.304754 -2.110257
H -0.791276 -3.268538 -2.778925
C -1.806926 -4.363783 -1.216893
H -1.065372 -5.163216 -1.179338
C -2.916030 -4.389125 -0.365344
H -3.041743 -5.214282 0.340536
C -3.854441 -3.361722 -0.392813
C 5.780389 -3.484393 1.141446
H 5.110493 -3.948551 1.878749
H 6.808853 -3.511611 1.525578
H 5.753329 -4.109014 0.234356
C 5.365788 -2.059060 0.838116
C 6.411433 -1.179060 0.492635
H 7.409856 -1.618512 0.520606
C 6.368335 0.171422 0.083736
C 7.697232 0.779618 -0.314374
H 7.907690 0.610344 -1.382492
H 8.514700 0.317071 0.255222
H 7.710780 1.864885 -0.143420
C 5.185638 2.171307 -0.507538
C 4.583073 3.212318 0.229979
C 4.330687 4.451475 -0.357796
H 3.859638 5.238791 0.236471
C 4.674611 4.688880 -1.689214
H 4.474601 5.659286 -2.149236
C 5.278119 3.666790 -2.428999
H 5.545247 3.836294 -3.475668
C 5.528626 2.425762 -1.851744
H -0.375345 2.132346 1.435676
H -2.097060 1.920357 3.183763
H -3.275897 -0.255638 3.541472
H -2.652319 -2.230815 2.123836
H -1.013889 -1.986769 0.313674
C 3.051177 -2.608224 1.116148
C 2.111891 -2.368811 2.141755
C 0.973412 -3.162285 2.268731
H 0.263101 -2.950501 3.071364
C 0.735568 -4.211463 1.380078
H -0.160168 -4.828064 1.478281
C 1.657843 -4.458560 0.358353
H 1.483254 -5.273061 -0.350159
C 2.797830 -3.671459 0.224111
H -2.475323 -1.450999 -2.858918
H 4.305475 3.029989 1.273475
H 2.287216 -1.541002 2.836666
H 3.493751 -3.848114 -0.598250
H 5.962107 1.620260 -2.447514
H -4.695215 -3.362247 0.304230
H -5.785952 2.362122 2.126000
H -3.076338 3.204946 -1.126524
C 0.899406 1.569176 -0.765099
C 0.228571 0.300516 -0.548263
C 1.015045 -0.841434 -0.967640
C 2.082977 -0.704431 -1.846746
C 1.952041 1.688648 -1.659179
C 2.553738 0.556344 -2.241696
H -1.044533 0.397838 -1.461416
H 0.728282 -1.843822 -0.648589
H 2.608333 -1.606131 -2.172126
H 2.373694 2.678801 -1.852622
H 3.409790 0.660320 -2.907311
H 0.484208 2.478083 -0.328416
H 2.183904 1.355028 2.313787

98

B10

Ca -3.308440 -0.001137 1.530998
Ca 3.405488 0.000386 -1.203266
N -3.759030 1.484816 -0.236196
N -3.759379 -1.486840 -0.236025
N 3.156032 1.521151 0.582974
N 3.156448 -1.520412 0.582781
C -0.536392 -1.205144 2.727275
C -0.271103 -1.205517 1.356637
C -0.119349 0.000723 0.641917
C -0.272232 1.206967 1.356356
C -0.537521 1.206687 2.727006
C -0.664335 0.000795 3.425580
C -4.910089 2.436369 -2.203099
H -4.910291 3.382293 -1.645168
H -5.921034 2.251433 -2.592254
H -4.247124 2.558252 -3.074236
C -4.472483 1.274411 -1.337672
C -4.890088 -0.001035 -1.768528
H -5.549017 -0.001027 -2.638644
C -4.472360 -1.276482 -1.337761
C -4.909377 -2.438444 -2.203473
H -4.245848 -2.560359 -3.074175
H -5.920079 -2.253572 -2.593284
H -4.909868 -3.384343 -1.645488
C -3.086941 2.676567 0.024372
C -2.214168 3.274578 -0.908420
C -1.432805 4.369441 -0.553526
H -0.756441 4.806558 -1.291664
C -1.488439 4.895366 0.741357
H -0.870668 5.752175 1.018706
C -2.355189 4.321288 1.672616
H -2.423924 4.730851 2.683645
C -3.151000 3.231835 1.320539
C -3.086543 -2.678126 0.024686
C -2.213436 -3.275746 -0.908045
C -1.431271 -4.369960 -0.552915
H -0.754701 -4.806822 -1.291015
C -1.486360 -4.895533 0.742133
H -0.867967 -5.751843 1.019642
C -2.353325 -4.321734 1.673367
H -2.421562 -4.730977 2.684559
C -3.149978 -3.232964 1.321087
C 3.018400 2.428208 2.882583
H 3.541833 3.315707 2.502535

H 3.483760 2.117180 3.826963
H 1.982535 2.723087 3.113122
C 3.048656 1.288943 1.886962
C 3.009921 0.000177 2.459392
H 2.966881 0.000123 3.549179
C 3.049068 -1.288497 1.886819
C 3.019124 -2.427966 2.882182
H 1.983354 -2.723469 3.112354
H 3.484053 -2.116926 3.826766
H 3.543101 -3.315117 2.502060
C 3.087205 -2.784689 -0.004179
C 3.958590 -3.053247 -1.085159
C 3.863416 -4.234026 -1.818571
H 4.554092 -4.402382 -2.648497
C 2.911328 -5.198162 -1.485744
H 2.843080 -6.130846 -2.049825
C 2.051737 -4.954669 -0.412443
H 1.306651 -5.702924 -0.133073
C 2.123406 -3.766964 0.311105
H -0.889534 0.000777 4.493248
H -0.669550 2.158718 3.244695
H -0.667454 -2.157141 3.245274
H -0.204910 -2.163133 0.842659
C 0.225474 0.000717 -0.801723
H -0.207027 2.164503 0.842116
C 3.086179 2.785512 -0.003753
C 3.957163 3.054487 -1.084972
C 3.861296 4.235233 -1.818318
H 4.551661 4.403912 -2.648439
C 2.908872 5.198959 -1.485207
H 2.840046 6.131616 -2.049265
C 2.049727 4.955094 -0.411648
H 1.304411 5.703033 -0.132043
C 2.122121 3.767409 0.311886
H -2.128810 -2.840813 -1.904985
H 4.741292 -2.329394 -1.345939
H 4.740037 2.330906 -1.345982
H 1.414673 3.594254 1.121610
H 1.415633 -3.594099 1.120615
H -3.845342 -2.805084 2.052727
H -3.846280 2.803832 2.052175
H -2.129210 2.839448 -1.905248
C 0.439137 1.207166 -1.501380
C 0.868135 1.207946 -2.830955
C 1.091865 0.000657 -3.504946
C 0.439043 -1.205754 -1.501365
C 0.868042 -1.206605 -2.830947
H 0.311770 2.164783 -1.000811
H 1.053175 2.161175 -3.330970
H 1.450277 0.000655 -4.536233
H 0.311596 -2.163366 -1.000789
H 1.052987 -2.159869 -3.330932
H -4.296185 -0.004351 3.320891
H 4.987740 0.000971 -2.502608

76

(^{Ph}BDICaH)$_2$

Ca 1.639265 -0.223571 0.001972
Ca -1.639218 0.223624 -0.003156
N 1.206214 -2.564796 -0.001549
N 3.849755 -1.129915 0.001327
N -3.849749 1.129939 0.000789
N -1.206267 2.565018 -0.003264
C 1.424756 -4.999188 -0.007351
H 0.779030 -5.145206 0.872349
H 2.200957 -5.773971 -0.007558
H 0.781894 -5.142040 -0.889669
C 2.019271 -3.609130 -0.003913
C 3.427333 -3.524242 -0.003403
H 3.945738 -4.482816 -0.005195
C 4.266479 -2.395563 -0.001268
C 5.753736 -2.670571 -0.001784
H 6.233080 -2.213750 -0.881267
H 5.966018 -3.746355 -0.003427
H 6.233067 -2.216385 0.879088
C -0.185366 -2.752015 -0.003318
C -0.904932 -2.778083 -1.213987
C -2.301793 -2.822329 -1.213021
H -2.845324 -2.828319 -2.161124
C -3.010757 -2.841955 -0.006536
H -4.100949 -2.862543 -0.007677
C -2.304472 -2.824282 1.201543
H -2.850179 -2.831703 2.148362
C -0.907642 -2.780014 1.205698
C 4.807732 -0.097022 0.002825
C 5.266835 0.459892 -1.203464
C 6.165028 1.526284 -1.200619
H 6.511646 1.943549 -2.149600
C 6.619426 2.063954 0.005953
H 7.320994 2.901108 0.007177
C 6.163202 1.524333 1.210941
H 6.508385 1.940066 2.161119
C 5.264991 0.457919 1.210689
C -5.753849 2.670456 -0.002196
H -6.233721 2.215676 -0.882453
H -5.966203 3.746227 -0.001314
H -6.232594 2.214159 0.877898
C -4.266571 2.395547 -0.003081
C -3.427503 3.524270 -0.007159
H -3.945938 4.482825 -0.009753
C -2.019430 3.609242 -0.006455
C -1.425047 4.999375 -0.008884
H -0.781049 5.145459 0.872069
H -2.201323 5.774083 -0.010562
H -0.780516 5.142293 -0.889968
C 0.185284 2.752177 -0.001152
C 0.908272 2.779372 -1.209788
C 2.305089 2.823563 -1.204855
H 2.851355 2.830400 -2.151361
C 3.010692 2.841901 0.003636
H 4.100882 2.862452 0.005419
C 2.301045 2.823042 1.209717
H 2.844053 2.829594 2.158108
C 0.904168 2.778884 1.209911
C -4.807541 0.096930 0.004680
C -5.272938 -0.457512 -1.200358

C -6.171176 -1.523847 -1.195041
H -6.522752 -1.939117 -2.143073
C -6.619225 -2.064094 0.012764
H -7.320819 -2.901223 0.015876
C -6.156583 -1.527083 1.216460
H -6.496769 -1.944767 2.167581
C -5.258443 -0.460611 1.213736
H 4.905570 0.036966 -2.144247
H 0.353535 2.727312 -2.147735
H -4.916480 -0.032806 -2.142176
H -4.891090 -0.037899 2.152255
H 0.346242 2.726626 2.145950
H 4.902456 0.033364 2.150249
H -0.352377 -2.728573 2.143364
H -0.347600 -2.725059 -2.150339
H -0.002417 -0.000611 1.407217
H 0.002144 0.000589 -1.408528

2

H_2

H 0.000000 0.000000 0.379948
H 0.000000 0.000000 -0.379948

12

C_6H_6

C 1.296735 -0.517294 0.000476
C 1.096552 0.864110 -0.000382
C -0.200423 1.381597 -0.000069
C -1.296722 0.517431 0.000170
C 0.200284 -1.381614 0.000020
C -1.096445 -0.864227 -0.000258
H 1.954588 1.541072 -0.000179
H -0.356563 2.463302 0.000359
H -2.311990 0.922170 0.000639
H 0.357014 -2.463247 -0.000311
H -1.954788 -1.540789 -0.000228
H 2.311855 -0.922526 -0.000020

7. References

[S1] S. Meiries, G. Le Duc, A. Chartoire, A. Collado, K. Speck, K. S. A. Arachchige, A. M. Z. Slawin, S. P. Nolan, *Chem. Eur. J.* **2013**, *19*, 17358–17368.

[S2] T. X. Gentner, B. Rösch, G. Ballmann, J. Langer, H. Elsen, S. Harder, *Angew. Chem. Int. Ed.* **2019**, *58*, 607–611.

[S3] B. Rösch, T. X. Gentner, J. Langer, C. Färber, J. Eyselein, L. Zhao, C. Ding, G. Frenking, S. Harder, *Science* **2021**, *371*, 1125–1128.

[S4] S. Pillai Sarish, A. Jana, H. W. Roesky, T. Schulz, M. John, D. Stalke, *Inorg. Chem.* **2010**, *49*, 3816–3820.

[S5] S. M. Nabavizadeh, F. N. Hosseini, C. Park, G. Wu, M. M. Abu-Omar, *Dalton Trans.* **2020**, *49*, 2477–2486.

[S6] K. G. Pearce, C. Dinoi, M. S. Hill, M. F. Mahon, L. Maron, R. S. Schwamm, A. S. S. Wilson, *Angew. Chem. Int. Ed.* **2022**, *61*, e202200305.

[S7] (a) Rigaku Oxford Diffraction, **2019**, CrysAlisPro Software system, version 1.171.40.53, Rigaku Corporation, Oxford, UK $[(^{DIPeP}BDI)SrI]_2$ (**3**); (b) Rigaku Oxford Diffraction, **2020**, CrysAlisPro Software system, version 1.171.40.84a, Rigaku Corporation, Oxford, UK $[(^{DIPP}BDI)Ca(THF)]_2$(biphenyl) (**2**-THF); (c) Rigaku Oxford Diffraction, **2021**, CrysAlisPro Software system, version 1.171.41.113a, Rigaku Corporation, Oxford, UK (all other compounds).

[S8] O. V. Dolomanov, L. J. Bourhis, R.J. Gildea, J. A. K. Howard, H. Puschmann, *J. Appl. Cryst.* **2009**, *42*, 339–341.

[S9] G. M. Sheldrick, *Acta Cryst. A* **2015**, *71*, 3–8.

[S10] G. M. Sheldrick, *Acta Cryst. C* **2015**, *71*, 3-8.

[S11] A. Thorn, B. Dittrich and G. M. Sheldrick, *Acta Cryst. A* **2012**, *68*, 448–451.

[S12] P. van der Sluis, A. L. Spek, *Acta Cryst. A* **1990**, *46*, 194–201.

[S13] J.-S. Jiang, A. T. Brünger, *J. Molec. Biol.* **1994**, *243*, 100–115.

[S14] M. J. Frisch, G. W. Trucks, H. B. Schlegel, G. E. Scuseria, M. A. Robb, J. R. Cheeseman, G. Scalmani, V. Barone, G. A. Petersson, H. Nakatsuji, X. Li, M. Caricato, A. V. Marenich, J. Bloino, B. G. Janesko, R. Gomperts, B. Mennucci, H. P. Hratchian, J. V. Ortiz, A. F. Izmaylov, J. L. Sonnenberg,

D. Williams-Young, F. Ding, F. Lipparini, F. Egidi, J. Goings, B. Peng, A. Petrone, T. Henderson, D. Ranasinghe, V. G. Zakrzewski, J. Gao, N. Rega, G. Zheng, W. Liang, M. Hada, M. Ehara, K. Toyota, R. Fukuda, J. Hasegawa, M. Ishida, T. Nakajima, Y. Honda, O. Kitao, H. Nakai, T. Vreven, K. Throssell, J. A. Montgomery, J. E. Peralta, F. Ogliaro, M. J. Bearpark, J. J. Heyd, E. N. Brothers, K. N. Kudin, V. N. Staroverov, T. A. Keith, R. Kobayashi, J. Normand, K. Raghavachari, A. P. Rendell, J. C. Burant, S. S. Iyengar, J. Tomasi, M. Cossi, J. M. Millam, M. Klene, C. Adamo, R. Cammi, J. W. Ochterski, R. L. Martin, K. Morokuma, O. Farkas, J. B. Foresman, D. J. Fox, *Gaussian 16 Rev. A.03*, Wallingford CT, **2016**.

[S15] A. D. Becke, *J. Chem. Phys.* **1993**, *98*, 5648–5652.

[S16] J. P. Perdew, J. A. Chevary, S. H. Vosko, K. A. Jackson, M. R. Pederson, D. J. Singh, C. Fiolhais, *Phys. Rev. B* **1993**, *48*, 4978–4978.

[S17] F. Weigend, *Phys. Chem. Chem. Phys.* **2006**, *8*, 1057–1065.

[S18] F. Weigend, R. Ahlrichs, *Phys. Chem. Chem. Phys.* **2005**, *7*, 3297–3305.

[S19] S. Grimme, S. Ehrlich, L. Goerigk, *J. Comput. Chem.* **2011**, *32*, 1456–1465.

[S20] E. D. Glendening, J. K. Badenhoop, A. E. Reed, J. E. Carpenter, J. A. Bohmann, C. M. Morales, P. Karafiloglou, C. R. Landis, F. Weinhold, *NBO 7.0*, Theoretical Chemistry Institute, University of Wisconsin, Madison, **2018**.

[S21] N. J. R. van Eikema Hommes, *Molecule*, Erlangen, **2018**.
